# Supplementary material for: Deficiency of neuronal LGR4 increases energy expenditure and inhibits food intake via hypothalamic leptin signaling
Source: EMBO Rep. 2025 Mar 11;26(8):2098–120. doi: 10.1038/s44319-025-00398-5 (PMC12018946; doi:10.1038/s44319-025-00398-5)
Supplement: Supplementary file 1 — Appendix [file 44319_2025_398_MOESM1_ESM.pdf]

Appendix Figures

Table of Contents

Appendix Figure S1…………………………………………………………………………….2

Appendix Figure S2…………………………………………………………………………….4

Appendix Figure S3…………………………………………………………………………….6

Appendix Figure S4…………………………………………………………………………….8

Appendix Figure S5…………………………………………………………………………….9

Appendix Figure S6…………………………………………………………………………...11

Appendix Figure S1

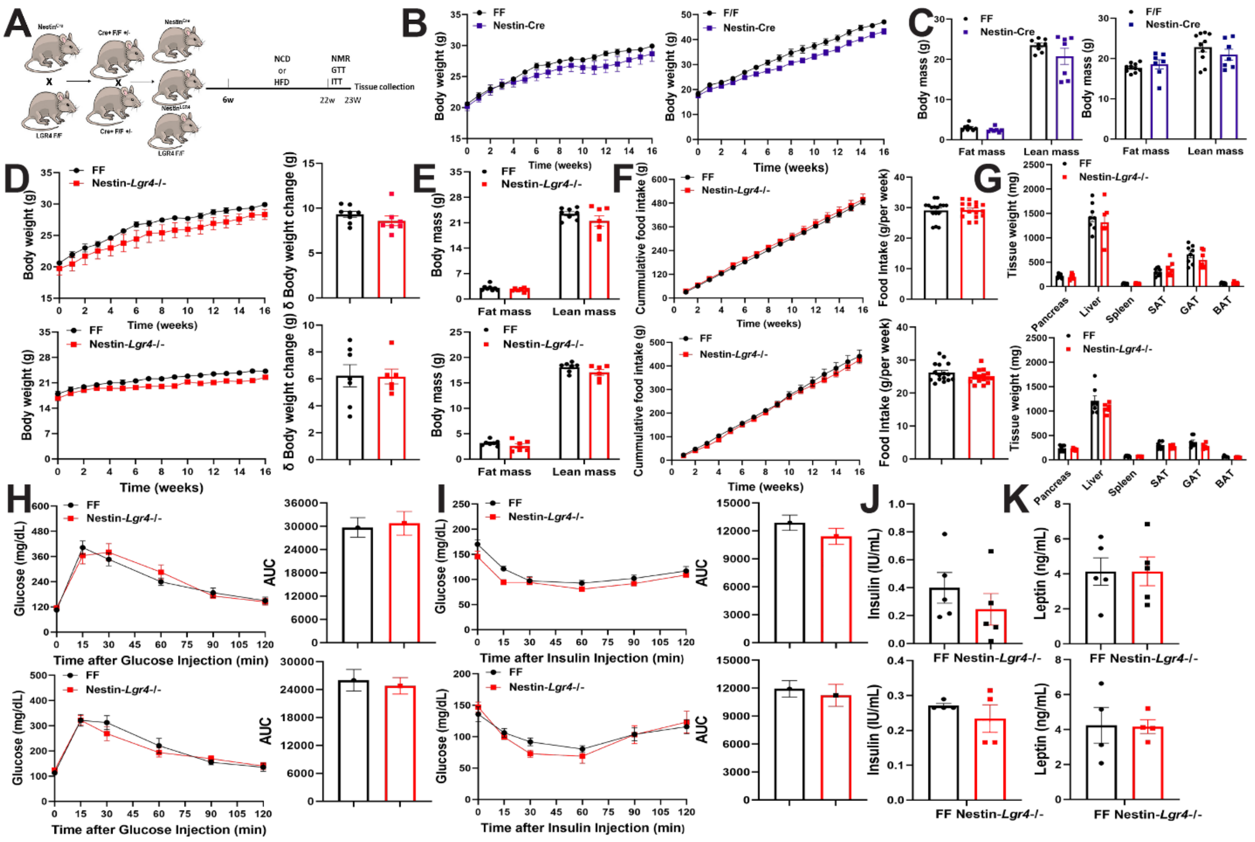

Appendix Figure S1. Metabolic effects of *Lgr 4* knockdown in *nestin* neurons in mice fed NCD

Mice were fed on NCD or HFD at 6 weeks old for 16 weeks. All data were shown as mean±SEM, *P<0.05.

(A-C) No change in body weight and body composition between FF and *Nestin-Cre* mice fed either NCD or HFD.

Mice were fed on NCD or HFD at 6 weeks old for 16 weeks. (**A**) Workflow chart. (**B**) Body weight curves of FF and *Nestin-Cre* mice under NCD (Left, FF n=8, *Nestin*-Cre n=7) and HFD (Right, FF n=10, *Nestin*-Cre n=7). (**C**) Body composition of FF and *Nestin-Cre* mice under NCD (Left, FF n=8, *Nestin*-Cre n=7) and HFD (Right, FF n=10, *Nestin*-Cre n=7).

(**D-K**) **Metabolic effects of *Lgr4* deficiency in *nestin* neurons of male or female mice fed NCD.** (**D**) Body weight curves (Left) and body weight changes (Right), male (Upper, FF n=8, *Nestin-Lgr4*$^{-/-}$ n=7), female (Lower, FF n=7, *Nestin-Lgr4*$^{-/-}$ n=6). (**E**) Body mass, male (Upper, FF n=8, *Nestin-Lgr4*$^{-/-}$ n=7), female (Lower, FF n=7, *Nestin-Lgr4*$^{-/-}$ n=6). (**F**) Cumulative food intake (Left) and weekly food intake (Right), male (Upper, FF n=8, *Nestin-Lgr4*$^{-/-}$ n=7), female (Lower, FF n=7, *Nestin-Lgr4*$^{-/-}$ n=6). (**G**) Tissue weights, male (Upper, FF n=8, *Nestin-Lgr4*$^{-/-}$ n=7), female (Lower, FF n=7, *Nestin-Lgr4*$^{-/-}$ n=6). (**H-I)** IPGTT (**H**) and IPITT (**I**), male (Upper, FF n=8, *Nestin-Lgr4*$^{-/-}$ n=7), female (Lower, FF n=7, *Nestin-Lgr4*$^{-/-}$ n=6). (**J-K**) Plasma Insulin (**J**) and Leptin (**K**) of mice under NCD, male (Upper, FF n=5, *Nestin-Lgr4*$^{-/-}$ n=5), female (Lower, FF n=4, Nestin-Lgr4-/- n=4).

Appendix Figure S2

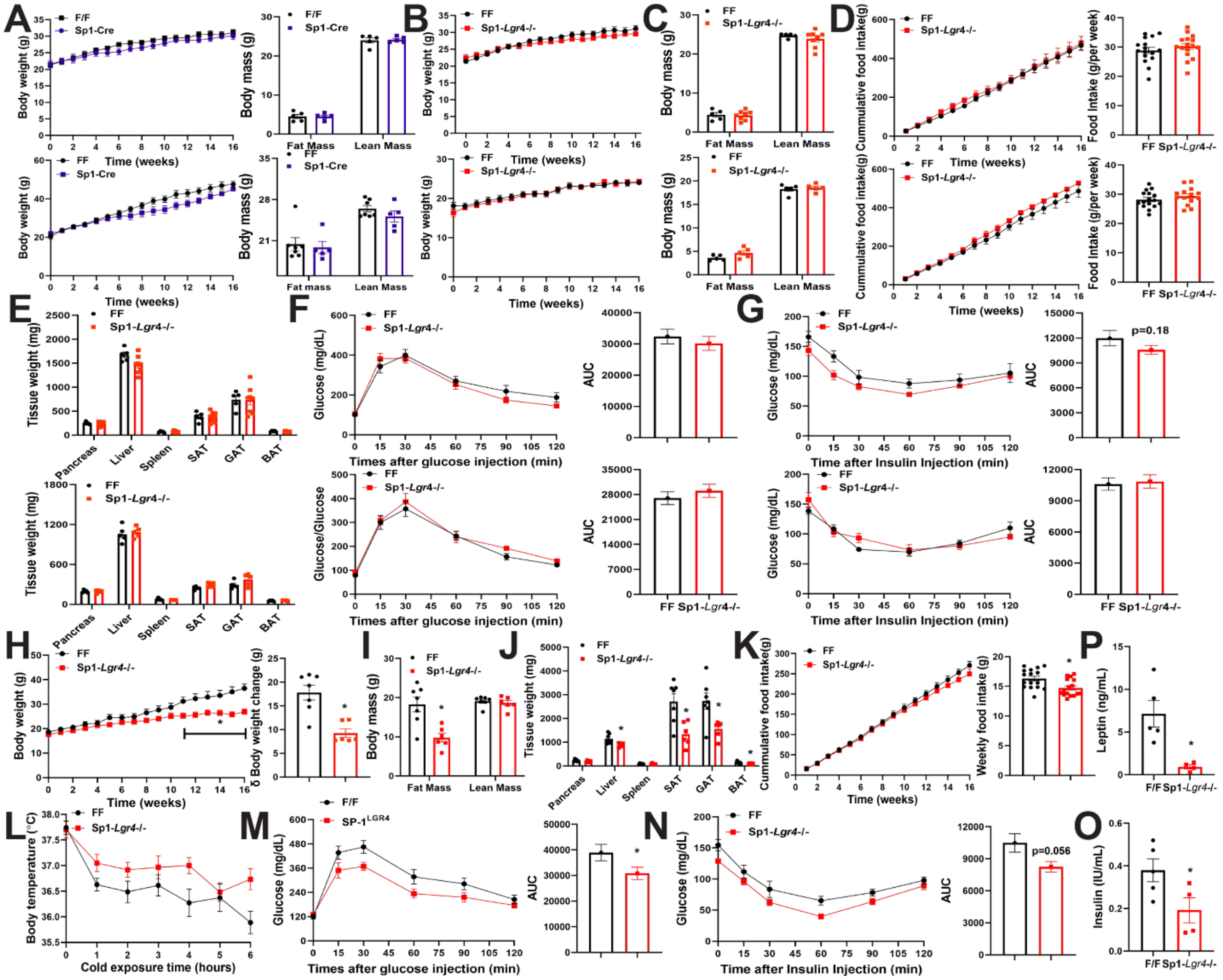

Appendix Figure S2. Metabolic effects of *Lgr 4* knockdown in *Sp1* neurons of male mice fed NCD or female mice fed NCD or HFD

Mice were fed NCD or HFD at 6 weeks old for 16 weeks. All data were shown as mean±SEM, *P<0.05.

(**A**) Body weight curves (Left) and body composition (Right) of FF and *Sp1*-Cre mice under NCD (Upper, FF n=5, *Sp1*-Cre n=5) and HFD (Lower, FF n=7, *Sp1*-Cre n=5).

(**B-G**) **Metabolic effects of *Lgr4* deficiency in *Sp1* neurons of male or female mice fed NCD.** (**B**) Body weight curves, male (Upper, FF n=5, *Sp1-Lgr4*$^{-/-}$ n=8), female (Lower, FF n=5, *Sp1-Lgr4*$^{-/-}$ n=5). (**C**) Body mass, male (Upper, FF n=5, *Sp1-Lgr4*$^{-/-}$ n=8), female (Lower, FF n=5, *Sp1-Lgr4*$^{-/-}$ n=5). (**D**) Cumulative food intake (Left) and weekly food intake (Right), male (Upper, FF n=5, *Sp1-Lgr4*$^{-/-}$ n=8), female (Lower, FF n=5, *Sp1-Lgr4*$^{-/-}$ n=5). (**E**) Tissue weights, male (Upper, FF n=5, *Sp1-Lgr4*$^{-/-}$ n=8), female (Lower, FF n=5, *Sp1-Lgr4*$^{-/-}$ n=5). (**F-G**) IPGTT (**F**) and IPITT (**G**), male (Upper, FF n=5, *Sp1-Lgr4*$^{-/-}$ n=8), female (Lower, FF n=5, *Sp1-Lgr4*$^{-/-}$ n=5).

(**H-Z**) **Metabolic effects of *Lgr4* deficiency in *Sp1* neurons of female mice fed HFD.** (**H**) Body weight and body weight change, FF n=7, *Sp1-Lgr4*$^{-/-}$ n=6. (**I**) Body mass, FF n=7, *Sp1-Lgr4*$^{-/-}$ n=6. (**J**) Tissue weights, FF n=7, *Sp1-Lgr4*$^{-/-}$ n=6. (**K**) Cumulative food intake (Left) and weekly food intake (Right), FF n=7, *Sp1-Lgr4*$^{-/-}$ n=6. (**L**) Rectal body temperature under 4 ^{o}C, FF n=7, *Sp1-Lgr4*$^{-/-}$ n=6. (**M-N**) IPGTT (**M**) and IPITT (**N**), FF n=7, *Sp1-Lgr4*$^{-/-}$ n=6. (**O**) Plasma Insulin, FF n=5, *Sp1-Lgr4*$^{-/-}$ n=4. (**P**) Plasma Leptin, FF n=5, *Sp1-Lgr4*$^{-/-}$ n=4.

Appendix Figure S3

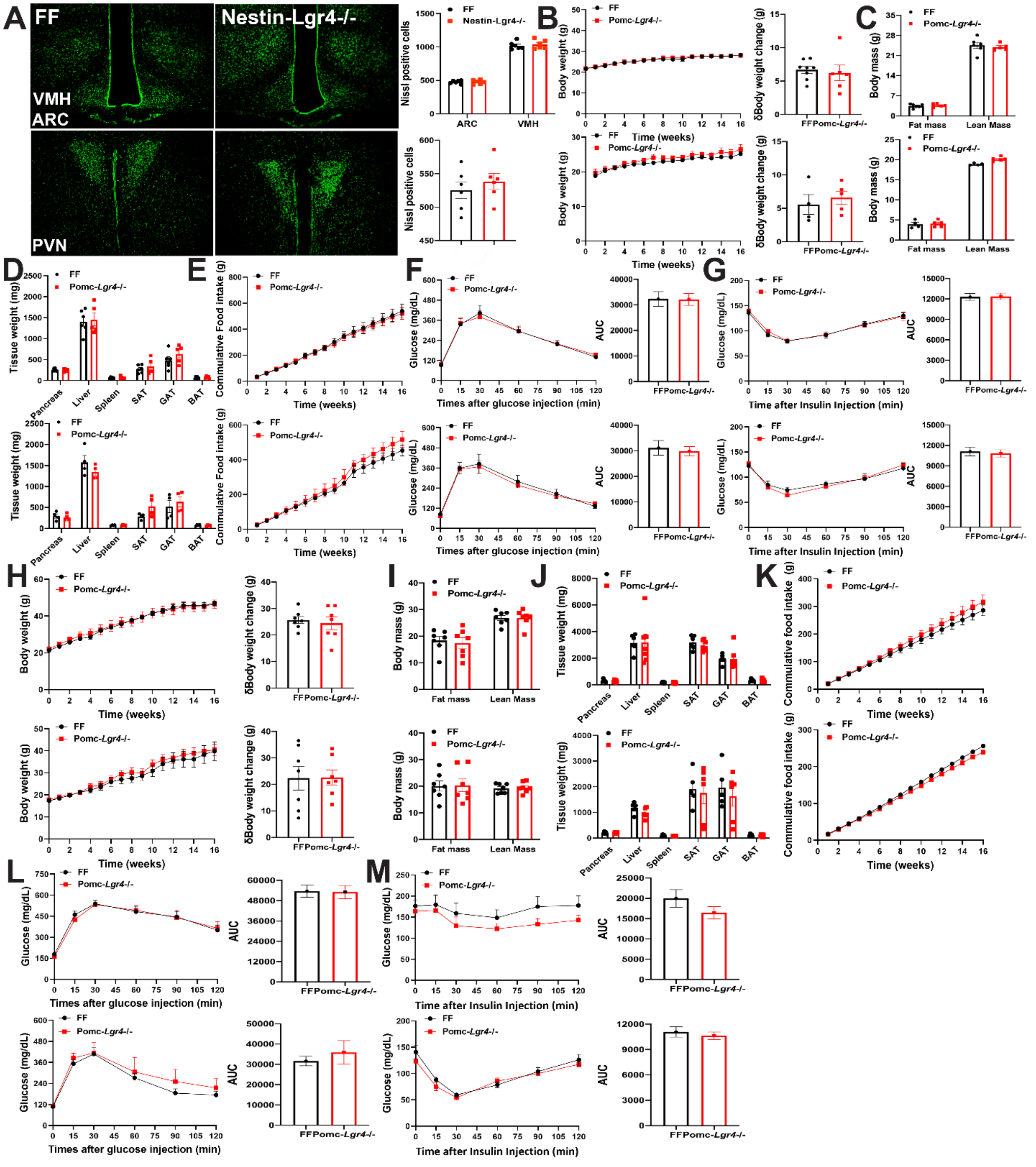

Appendix Figure S3. No effect of Lgr4 knockdown in *Pomc* specific neurons

Mice were fed on NCD or HFD at 6 weeks old for 16 weeks. All data were shown as mean±SEM, *P<0.05.

(**A**) Representative Nissl staining of hypothalamus slides, FF n=3, *Nestin-Lgr4*$^{-/-}$ n=3. (**B-G**) Male and female mice fed with NCD. (**B**)Body weight curves (Left) and body weight changes (Right), male (Upper, FF n=8, *Pomc-Lgr4*$^{-/-}$ n=6), female (Lower, FF n=4, *Pomc-Lgr4*$^{-/-}$ n=5). (**C**) Body mass of mice with NCD, male (Upper, FF n=6, *Pomc-Lgr4*$^{-/-}$ n=5), female (Lower, FF n=4, *Pomc-Lgr4*$^{-/-}$ n=5). (**D**) Tissue weights, male (Upper, FF n=6, *Pomc-Lgr4*$^{-/-}$ n=5), female (Lower, FF n=4, *Pomc-Lgr4*$^{-/-}$ n=4). (**E**) Cumulative food intake (Left) and weekly food intake (Right), male (Upper, FF n=6, *Pomc-Lgr4*$^{-/-}$ n=5), female (Lower, FF n=4, *Pomc-Lgr4*$^{-/-}$ n=5). (**F-G**)**,** IPGTT (**F**) and IPITT (**G**), male (Upper, FF n=6, *Pomc-Lgr4*$^{-/-}$ n=6), female (Lower, FF n=4, *Pomc-Lgr4*$^{-/-}$ n=5). (**H-M**) Male and female mice fed with HFD. (**H**) Body weight (Left) and body weight change (Right), male (Upper, FF n=7, *Pomc-Lgr4*$^{-/-}$ n=7), female (Lower, FF n=7, *Pomc-Lgr4*$^{-/-}$ n=7). (**I**) Body mass, male (Upper, FF n=7, *Pomc-Lgr4*$^{-/-}$ n=7), female (Lower, FF n=7, *Pomc-Lgr4*$^{-/-}$ n=7). (**J**) Tissue weights, male (Upper, FF n=6, *Pomc-Lgr4*$^{-/-}$ n=7), female (Lower, FF n=5, *Pomc-Lgr4*$^{-/-}$ n=6). (**K**) Cumulative food intake, male (Upper, FF n=7, *Pomc-Lgr4*$^{-/-}$ n=7), female (Lower, FF n=7, *Pomc-Lgr4*$^{-/-}$ n=7). (**L-M**) IPGTT (**L**) and IPITT (**M**), male (upper, FF n=6, *Pomc-Lgr4*$^{-/-}$ n=7), female (lower, FF n=5, *Pomc-Lgr4*$^{-/-}$ n=5).

Appendix Figure S4

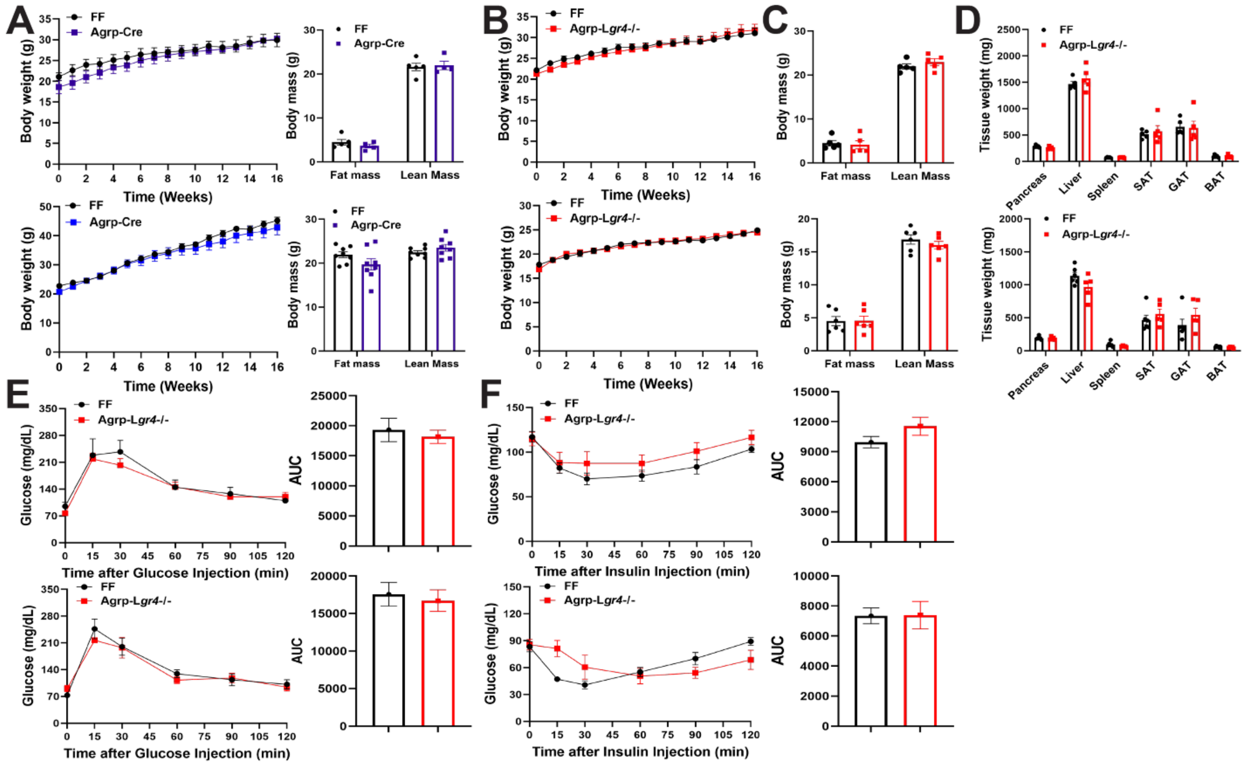

Appendix Figure S4. Metabolic effects of Lgr 4 knockdown in *AgRP* neurons in mice fed NCD

Mice were fed on NCD or HFD at 6 weeks old for 16 weeks. All data were shown as mean±SEM, *P<0.05.

(**A**) Body weight curves (Left) and body composition (Right) of FF and *Agrp*-Cre mice under NCD (Upper, FF n=5, *Agrp*-Cre n=4) and HFD (Lower, FF n=8, *Agrp*-Cre n=8).

(**B-E**) **Metabolic effects of *Lgr4* deficiency in *AgRP* neurons of male or female mice fed NCD.** (**B**) Body weight curves (Left) and body weight changes (Right), male (Upper, FF n=5, *Agrp-Lgr4*-/- n=5), female (Lower, FF n=6, *Agrp-Lgr4*$^{-/-}$ n=6). (**C**) Body composition, male (Upper, FF n=5, *Agrp-Lgr4*$^{-/-}$ n=5), female (Lower, FF n=6, *Agrp-Lgr4*$^{-/-}$ n=6). (**D**) Tissue weights,

male (Upper, FF n=5, *Agrp-Lgr4*$^{-/-}$ n=5), female (Lower, FF n=6, *Agrp-Lgr4*$^{-/-}$ n=6). (**E-F**) IPGTT (**E**) and IPITT (**F**) L, male (Upper, FF n=5, *Agrp-Lgr4*$^{-/-}$ n=5), female (Lower, FF n=6, *Agrp-Lgr4*$^{-/-}$ n=6).

Appendix Figure S5

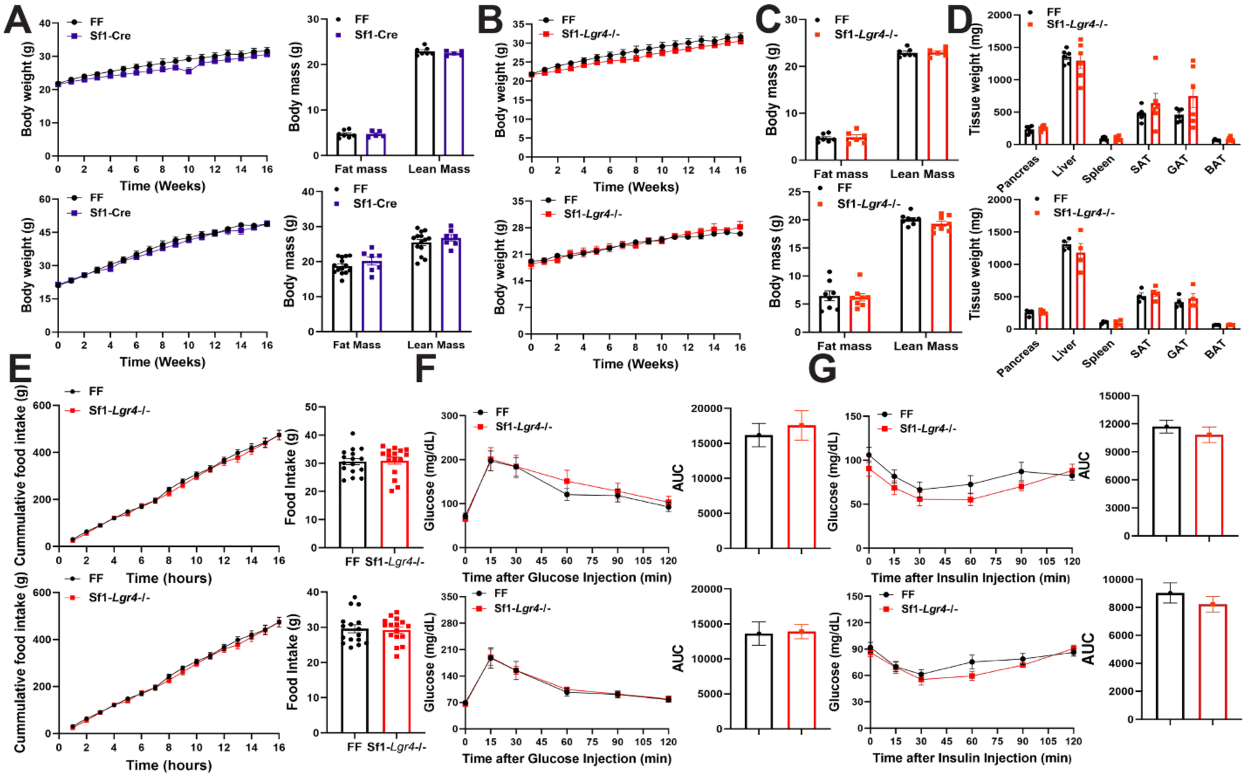

Appendix Figure S5. Metabolic effects of Lgr 4 knockdown in *Sf1* neurons in mice fed NCD

Mice were fed on NCD or HFD at 6 weeks old for 16 weeks. All data were shown as mean±SEM, *P<0.05.

(**A**) Body weight curves (Left) and body composition (Right) of FF and *Sf1*-Cre mice under NCD (upper, FF n=7, *Sf1*-Cre n=5) and HFD (lower, FF n=14, *Sf1*-Cre n=7).

(**B-G**) **Metabolic effects of *Lgr 4* knockdown in *Sf1* neurons of male and female mice fed NCD.** (**B**)Body weight curves, male (Upper, FF n=7, *Sf1-Lgr4*$^{-/-}$ n=6), female (Lower, FF n=8,

Sf1-*Lgr4*$^{-/-}$ n=8). (**C**) Body mass, male (Upper, FF n=7, *Sf1*-*Lgr4*$^{-/-}$ n=6), female (Lower, FF n=8, *Sf1*-*Lgr4*$^{-/-}$ n=8). (**D**) Tissue weights, male (Upper, FF n=6, *Sf1*-*Lgr4*$^{-/-}$ n=6), female (Lower, FF n=4, *Sf1*-*Lgr4*$^{-/-}$ n=4). (**E**) Cumulative food intake (Left) and weekly food intake (Right), male (Upper, FF n=7, *Sf1*-*Lgr4*$^{-/-}$ n=6), female (Lower, FF n=8, *Sf1*-*Lgr4*$^{-/-}$ n=8). (**F**-**G**) IPGTT (**F**) and IPITT (**G**), male (Upper, FF n=7, *Sf1*-*Lgr4*$^{-/-}$ n=6), female (Lower, FF n=8, *Sf1*-*Lgr4*$^{-/-}$ n=8).

Appendix Figure S6

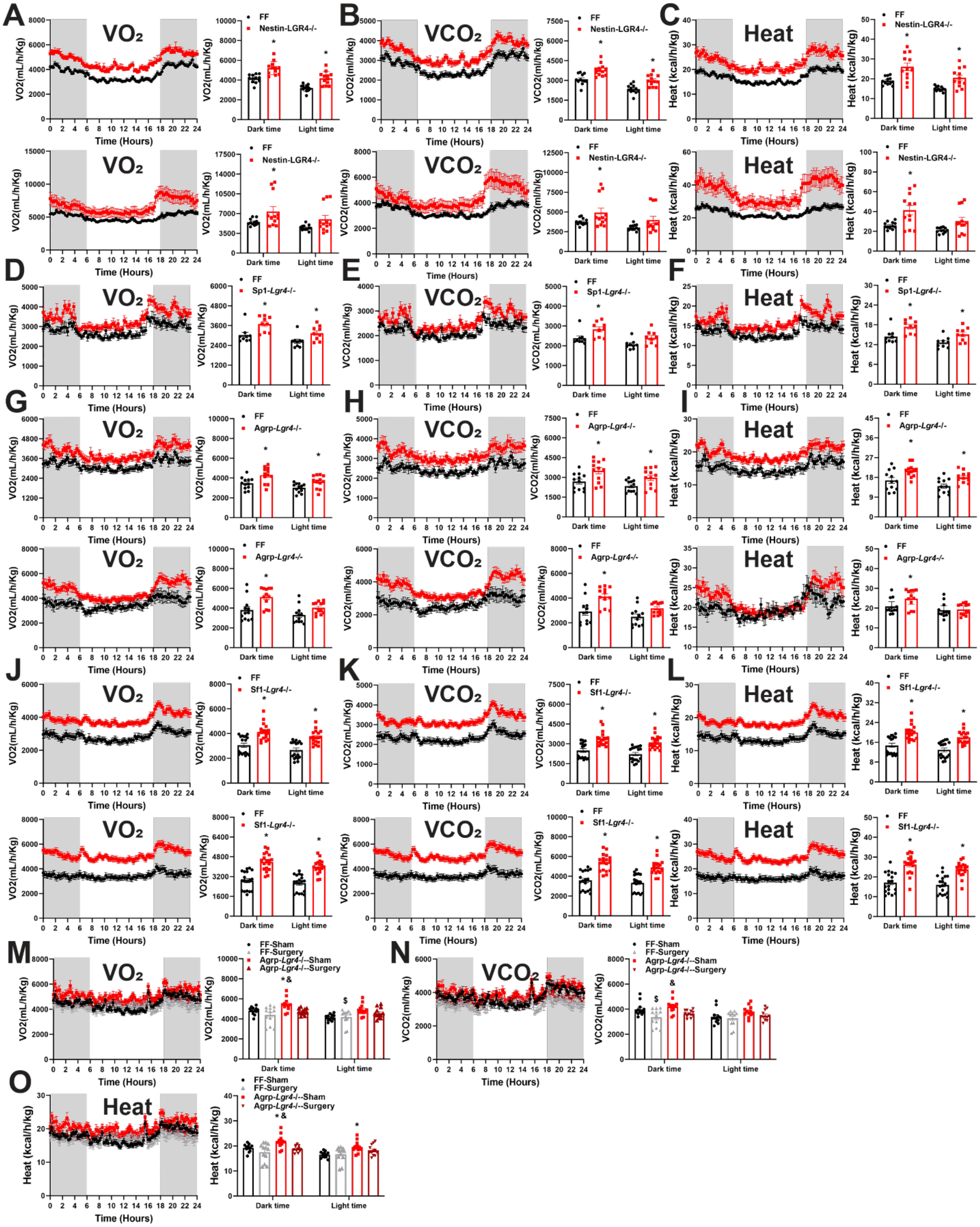

Appendix Figure S6.TSE phenotype data of mice with knockdown of LGR4 in estin/Sp-1/AgRP/Sf-1 specific neurons.

(**A-L**) Mice fed HFD were placed in TSE chamber for 7 days, data for the last 3 days were recorded and analyzed. All data were shown as mean±SEM, *P<0.05. (**A-C**) FF n=4, Nestin-Lgr4-/- n=4. (**A**) O2 consumption (Upper: male; Lower: female). (**B**) CO2 production (Upper: male; Lower: female). (**C**) Energy expenditure (Upper: male; Lower: female). (**D-F**) FF n=3, Sp1-Lgr4-/- n=3. (D) O2 consumption. (E) CO2 production. F Energy expenditure. (**G-I**) FF n=4, Agrp-Lgr4-/- n=4. (**G**) O2 consumption (Upper: male; Lower: female). (**H**) CO2 production (Upper: male; Lower: female). (**I**) Energy expenditure (Upper: male; Lower: female). (**J-L**) FF n=6, Sf1-Lgr4-/- n=6. (**J**) O2 consumption (Upper: male; Lower: female). (**K**) CO2 production (Upper: male; Lower: female). (**L**) Energy expenditure (Upper: male; Lower: female).

(**M-O**) Mice fed HFD with or without Intrascapular BAT Bilateral Sympathectomy were placed in TSE chamber for 7 days, data for the last 3 days were recorded and analyzed. All data were shown as mean±SEM, *P<0.05, * FF-sham vs Agrp-Lgr4-/--sham, $ FF-sham vs FF-Surgery, & Agrp-Lgr4-/--sham vs Agrp-Lgr4-/--Surgery, # FF- Surgery vs Agrp-Lgr4-/--Surgery, n=4. (**M**) O2 consumption. (**N**) CO2 production. (**O**) Energy expenditure.
