# Supplementary material for: Deficiency of neuronal LGR4 increases energy expenditure and inhibits food intake via hypothalamic leptin signaling
Source: EMBO Rep. 2025 Mar 11;26(8):2098–120. doi: 10.1038/s44319-025-00398-5 (PMC12018946; doi:10.1038/s44319-025-00398-5)

Figure 1 A

Graph in figures

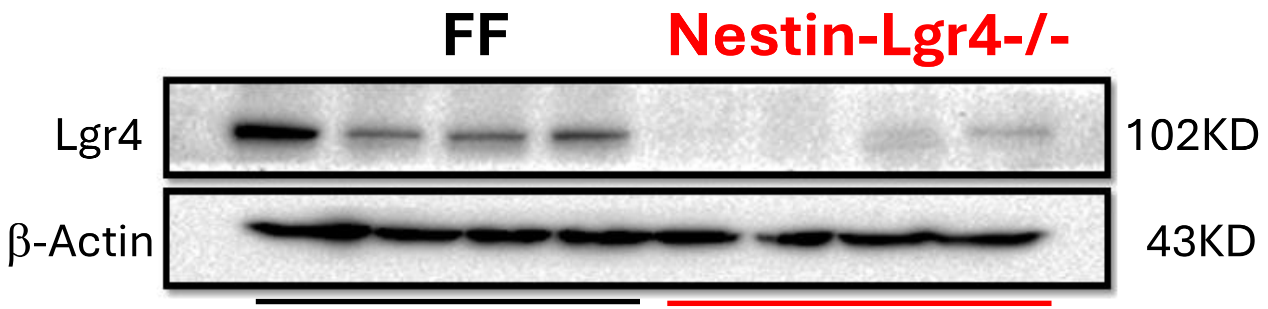

Corresponding uncropped images

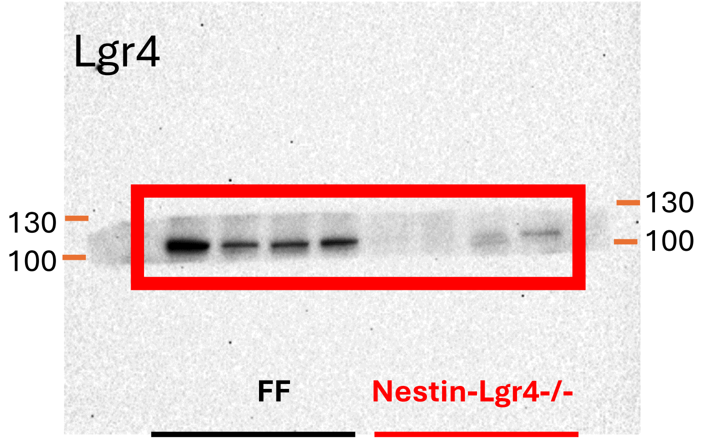

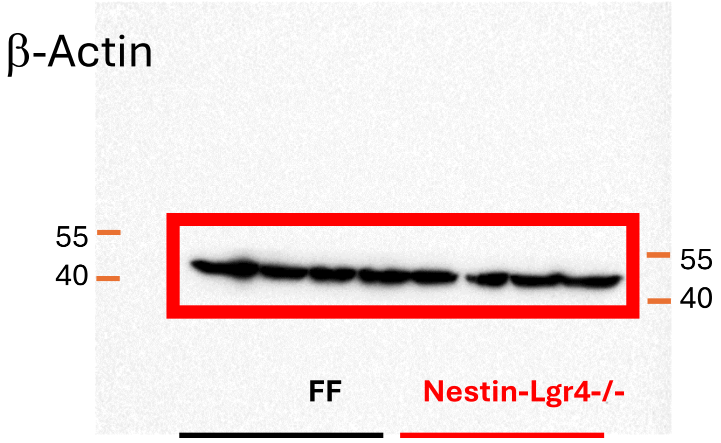

Figure 1 T

Graph in figures

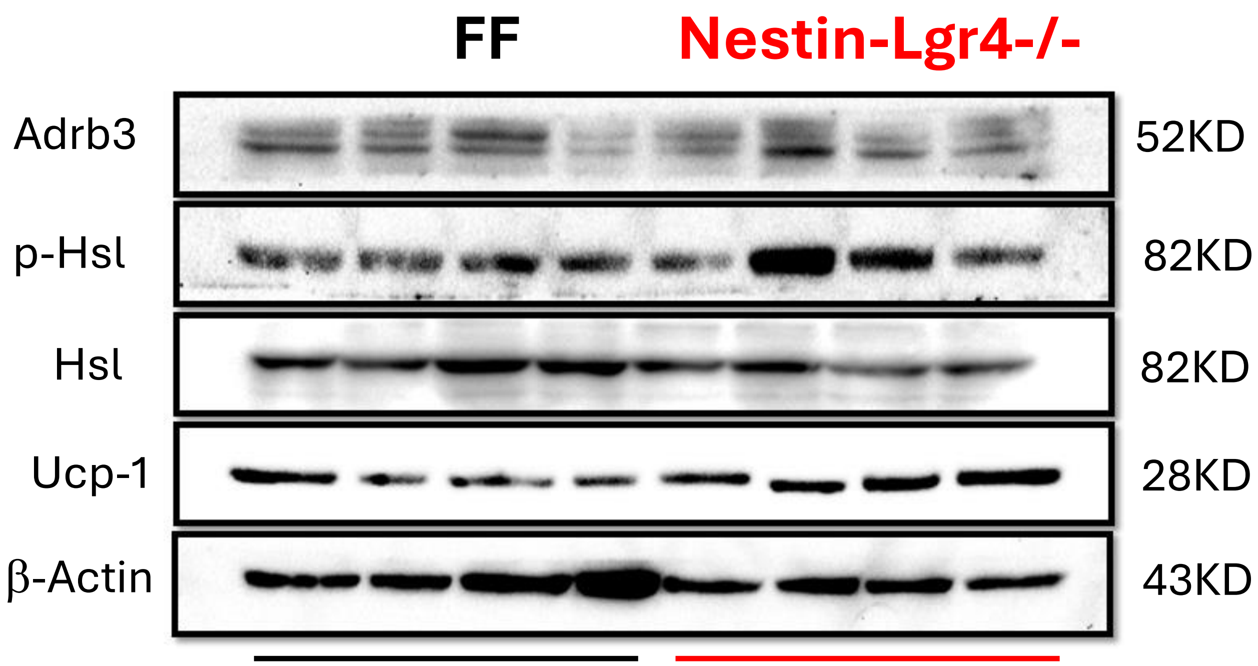

# Corresponding uncropped images

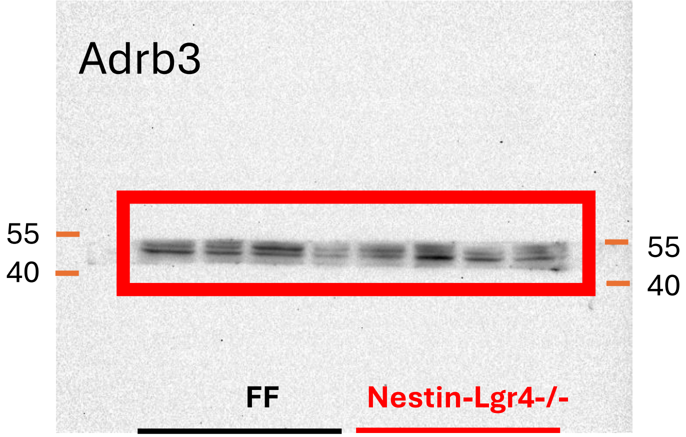

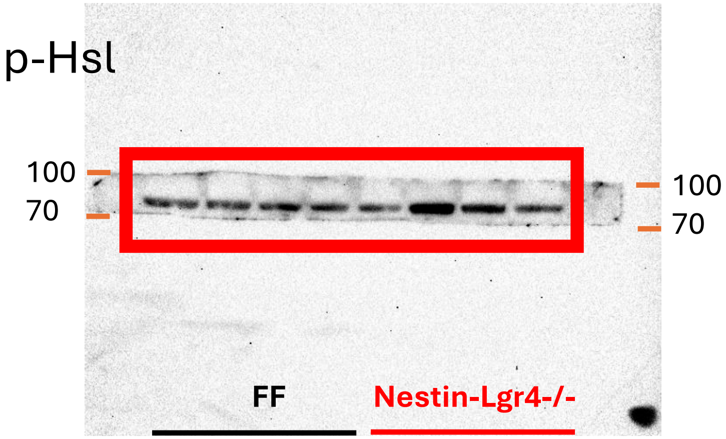

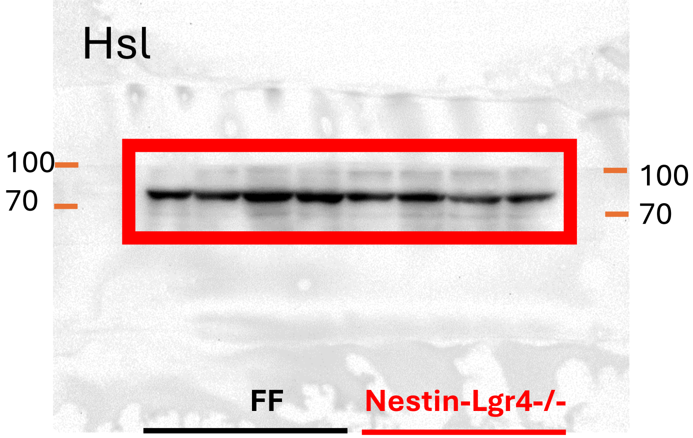

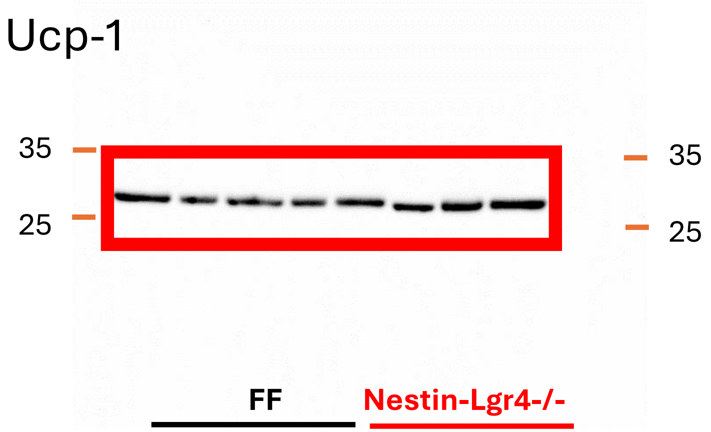

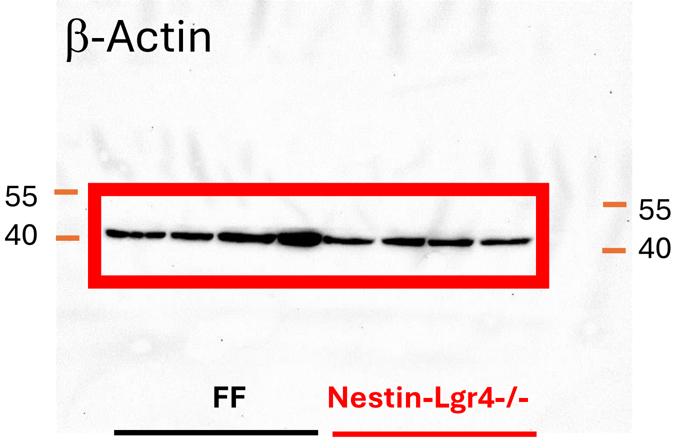

Figure 1 W

Graph in figures

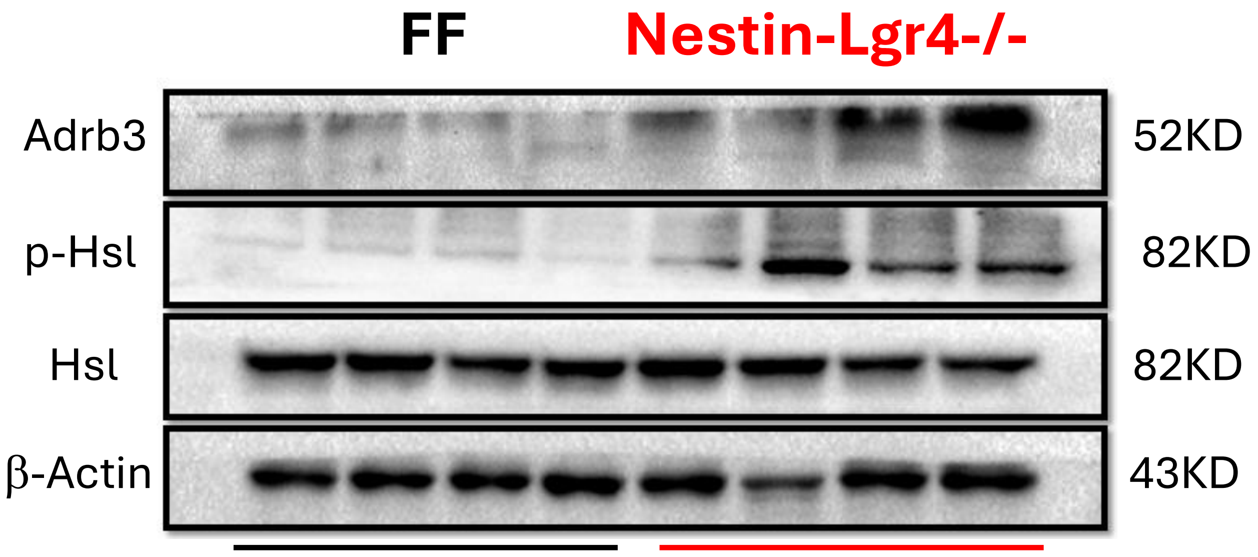

Corresponding uncropped images

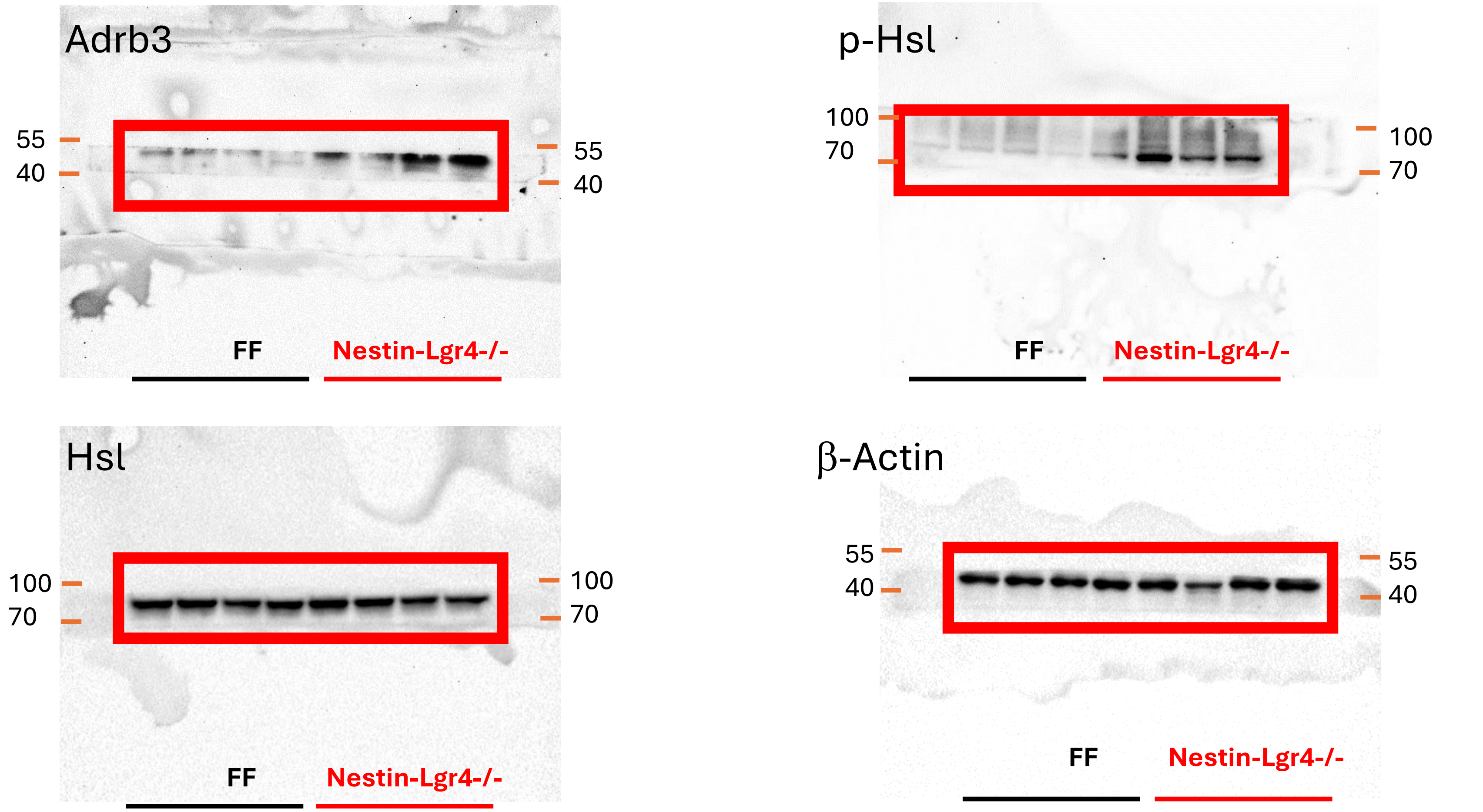

Supplement: Supplementary file 3 — Source data Fig. 1 [file 44319_2025_398_MOESM3_ESM.zip › Figure 1/Uncropped western blots of Figure 1.pdf]
