# Supplementary figures and images for: Deficiency of neuronal LGR4 increases energy expenditure and inhibits food intake via hypothalamic leptin signaling (part 1 of 2)
Source: EMBO Rep. 2025 Mar 11;26(8):2098–120. doi: 10.1038/s44319-025-00398-5 (PMC12018946; doi:10.1038/s44319-025-00398-5)

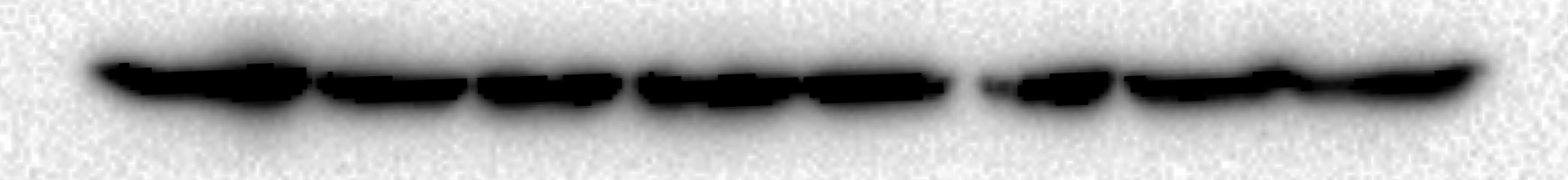

Supplement: Supplementary file 3 — Source data Fig. 1 [file 44319_2025_398_MOESM3_ESM.zip › Figure 1/Figure 1A/Western-actin.tif]

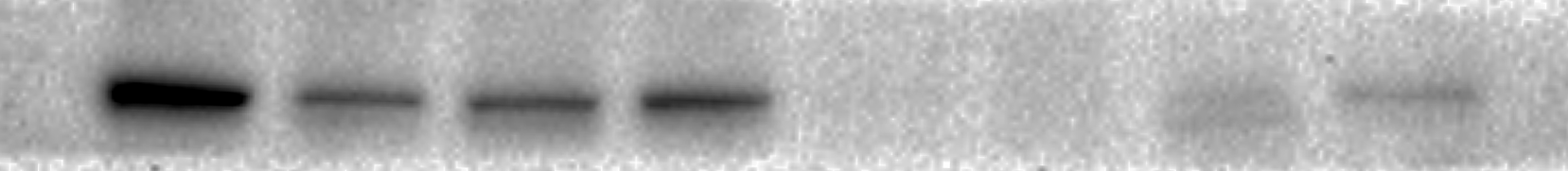

Supplement: Supplementary file 3 — Source data Fig. 1 [file 44319_2025_398_MOESM3_ESM.zip › Figure 1/Figure 1A/Western-Lgr4.tif]

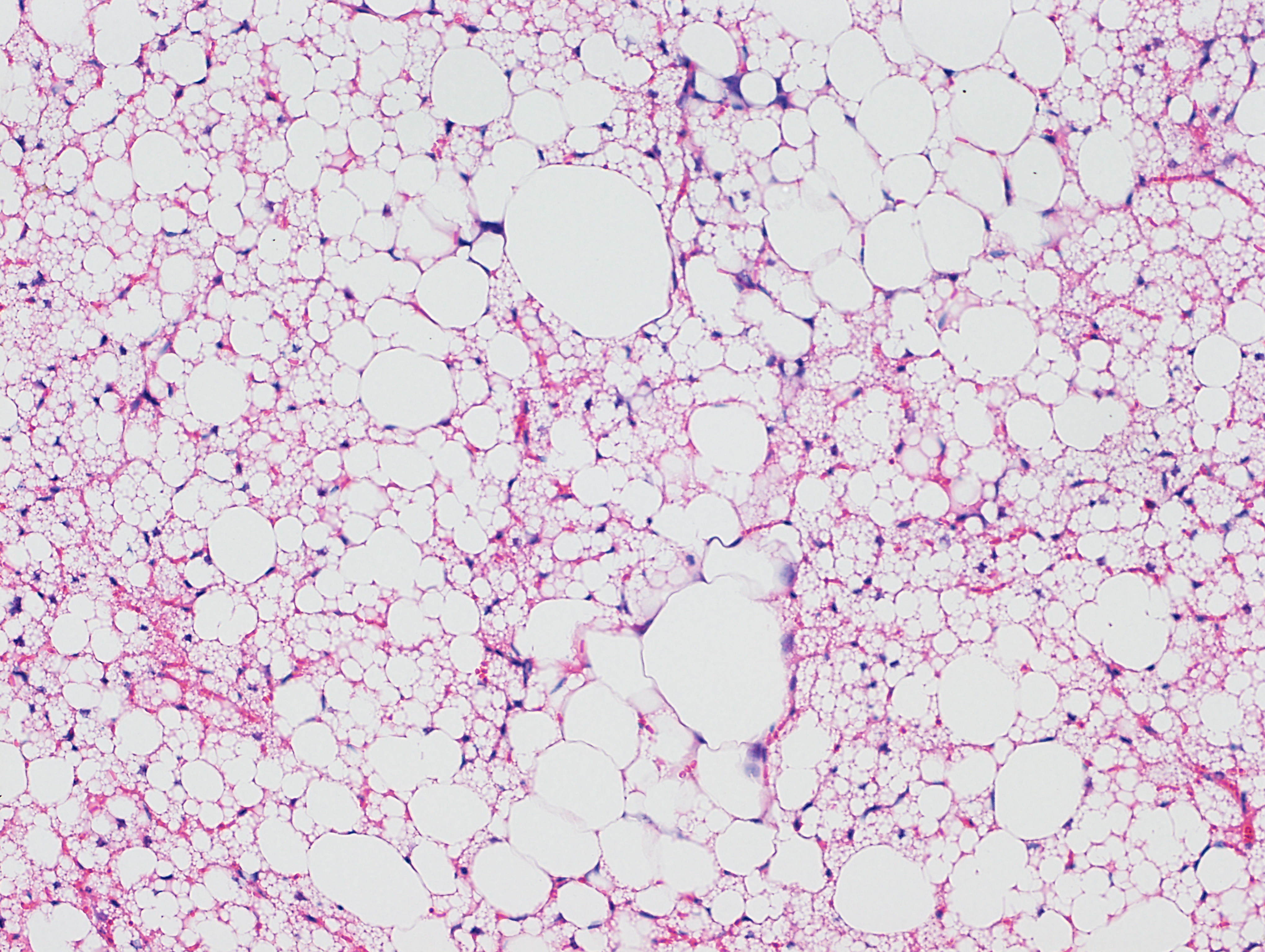

Supplement: Supplementary file 3 — Source data Fig. 1 [file 44319_2025_398_MOESM3_ESM.zip › Figure 1/Figure 1P/BAT-FF.tif]

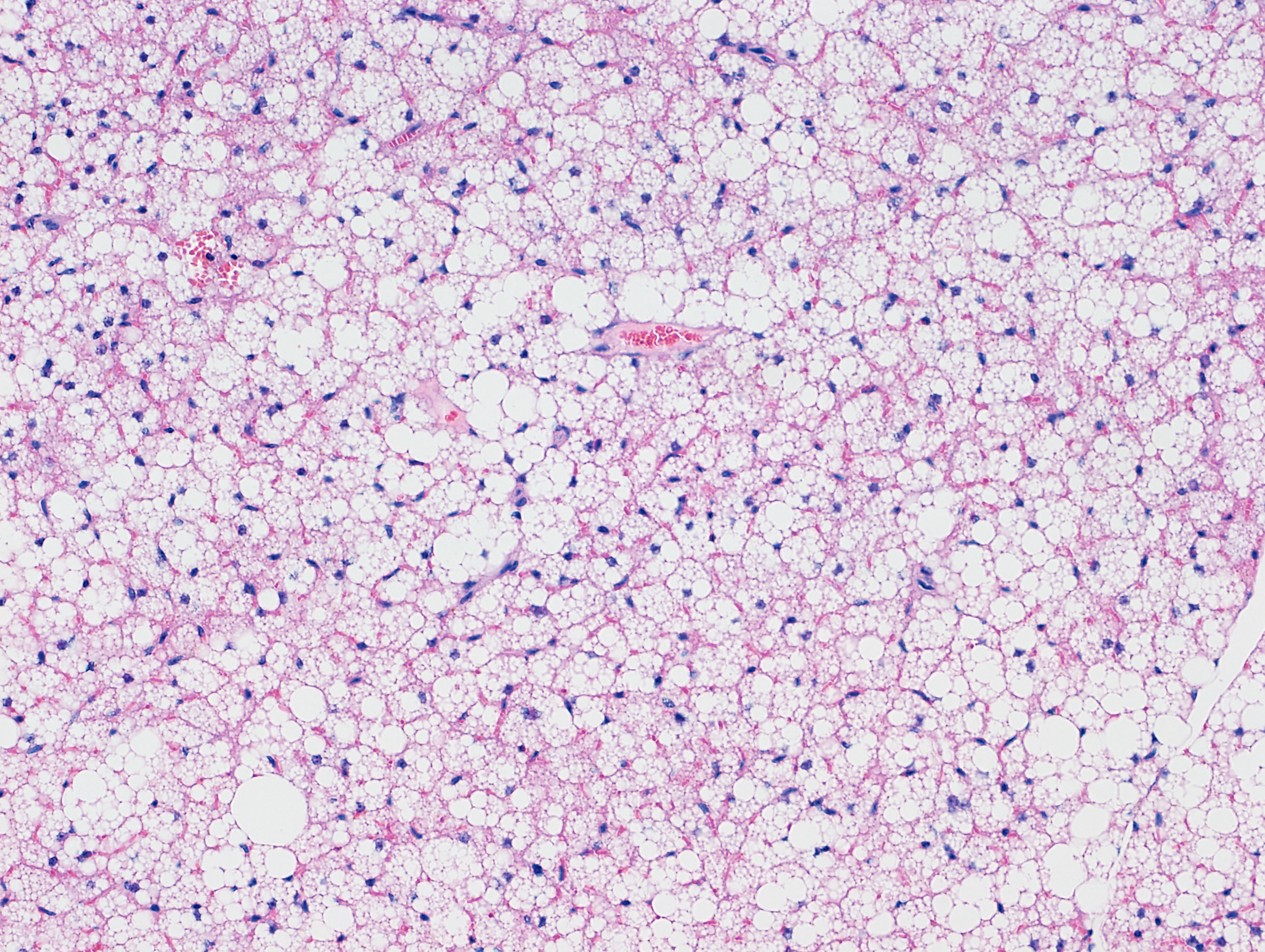

Supplement: Supplementary file 3 — Source data Fig. 1 [file 44319_2025_398_MOESM3_ESM.zip › Figure 1/Figure 1P/BAT-KO.tif]

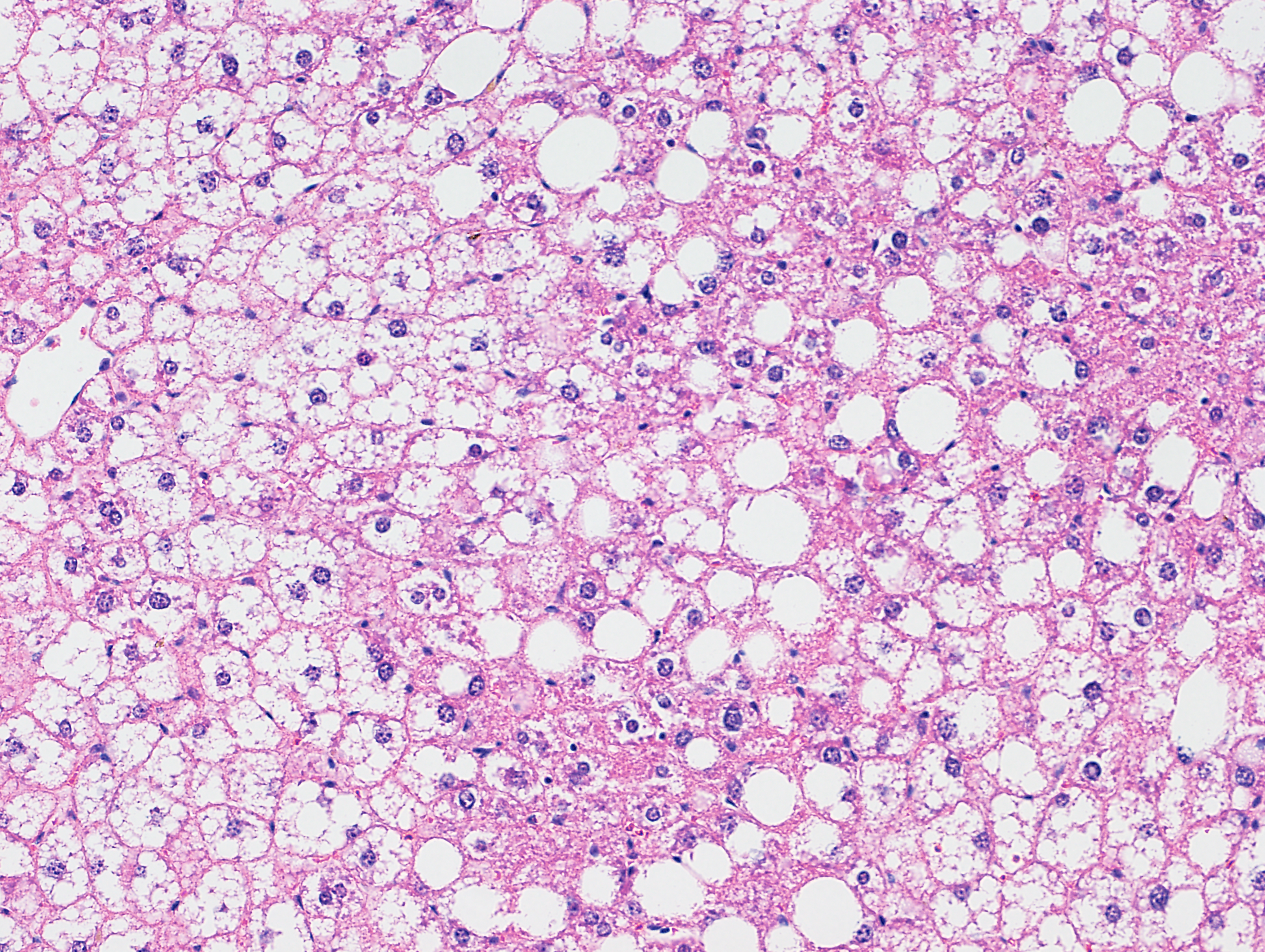

Supplement: Supplementary file 3 — Source data Fig. 1 [file 44319_2025_398_MOESM3_ESM.zip › Figure 1/Figure 1P/Liver-FF.tif]

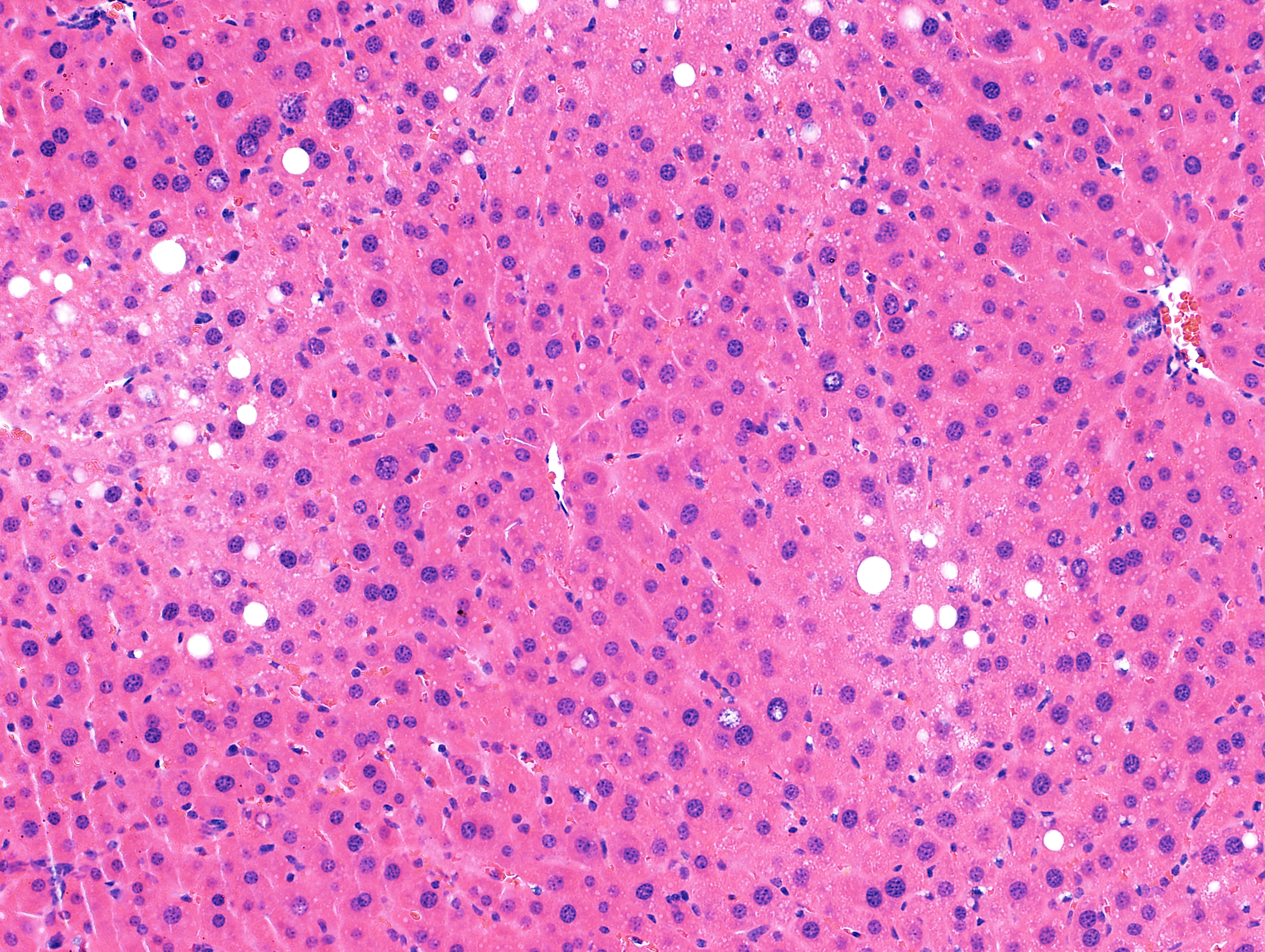

Supplement: Supplementary file 3 — Source data Fig. 1 [file 44319_2025_398_MOESM3_ESM.zip › Figure 1/Figure 1P/Liver-KO.tif]

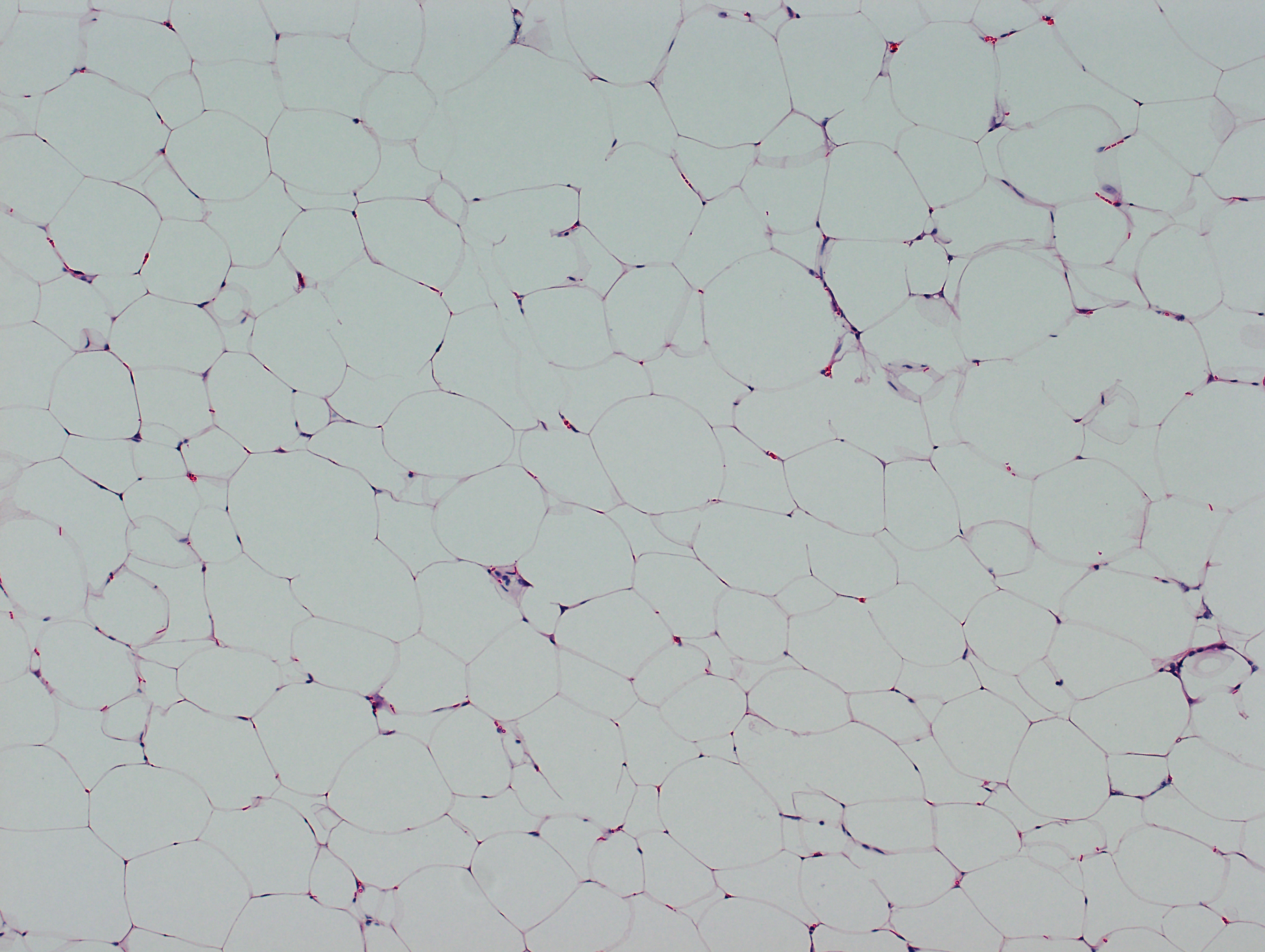

Supplement: Supplementary file 3 — Source data Fig. 1 [file 44319_2025_398_MOESM3_ESM.zip › Figure 1/Figure 1P/SAT-FF.tif]

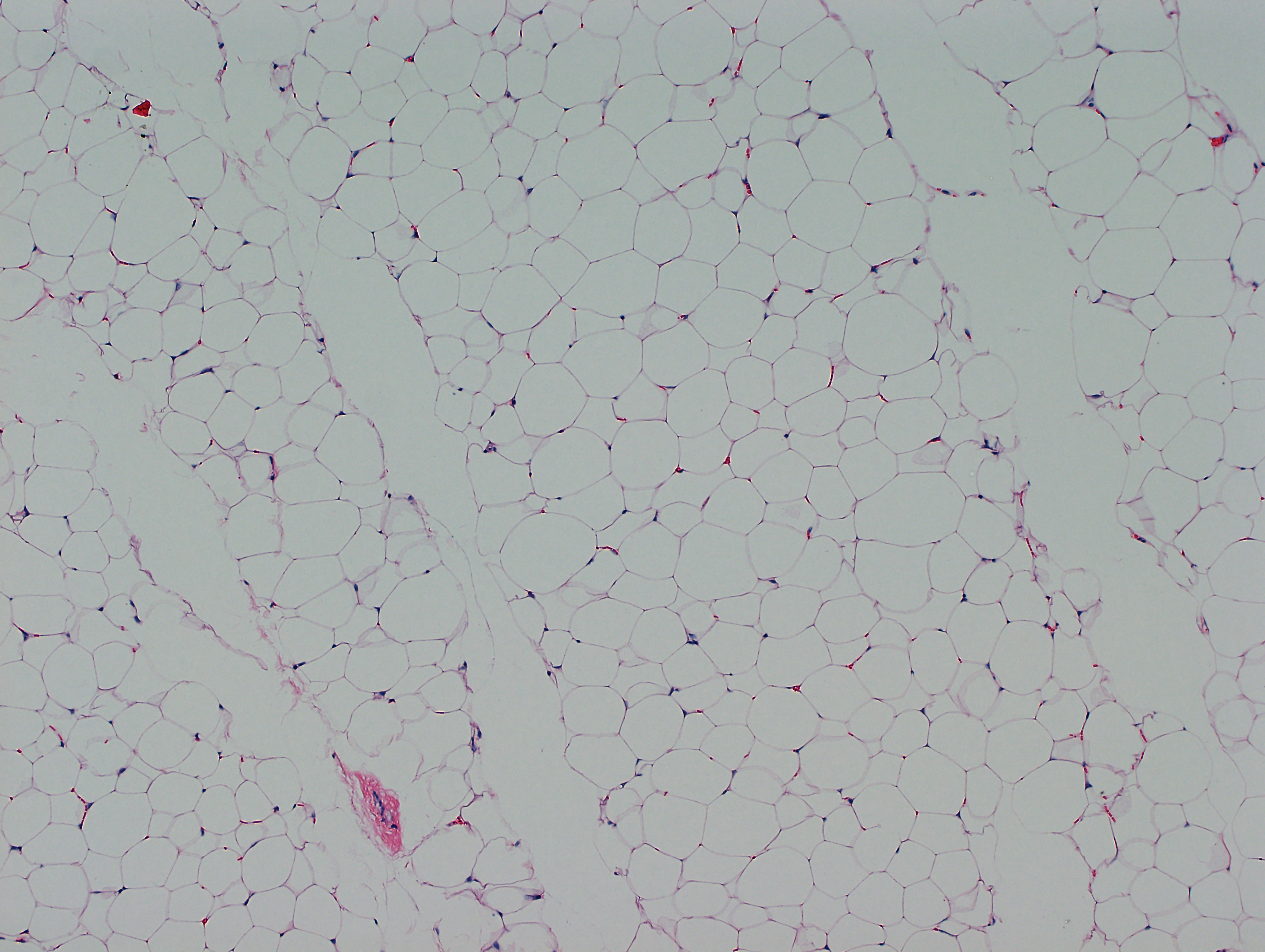

Supplement: Supplementary file 3 — Source data Fig. 1 [file 44319_2025_398_MOESM3_ESM.zip › Figure 1/Figure 1P/SAT-KO.tif]

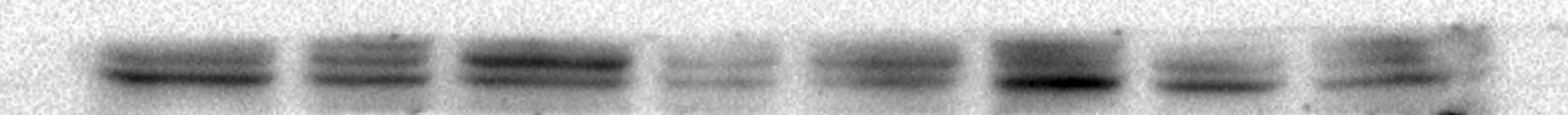

Supplement: Supplementary file 3 — Source data Fig. 1 [file 44319_2025_398_MOESM3_ESM.zip › Figure 1/Figure 1T/Western blot-adrb3.tif]

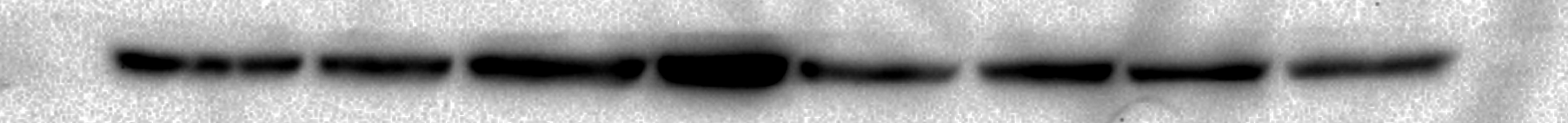

Supplement: Supplementary file 3 — Source data Fig. 1 [file 44319_2025_398_MOESM3_ESM.zip › Figure 1/Figure 1T/Western blot-beta-actin.tif]

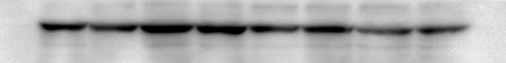

Supplement: Supplementary file 3 — Source data Fig. 1 [file 44319_2025_398_MOESM3_ESM.zip › Figure 1/Figure 1T/Western blot-HSL.tif]

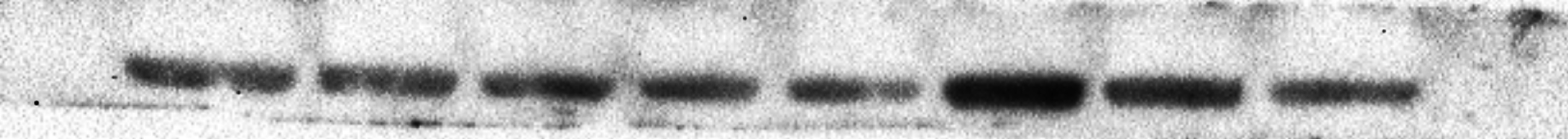

Supplement: Supplementary file 3 — Source data Fig. 1 [file 44319_2025_398_MOESM3_ESM.zip › Figure 1/Figure 1T/Western blot-p-hsl.tif]

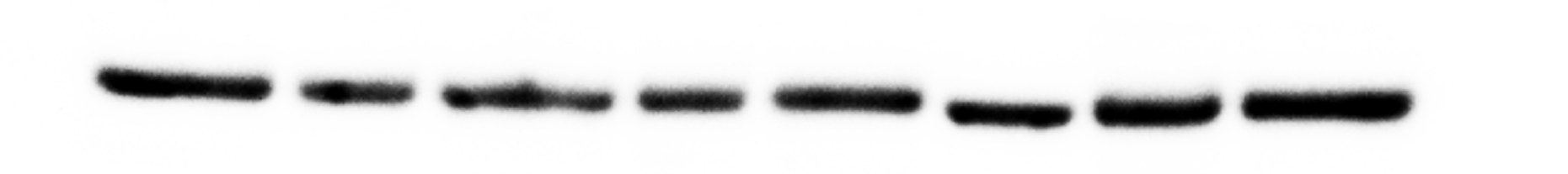

Supplement: Supplementary file 3 — Source data Fig. 1 [file 44319_2025_398_MOESM3_ESM.zip › Figure 1/Figure 1T/Western blot-Ucp-1 1.tif]

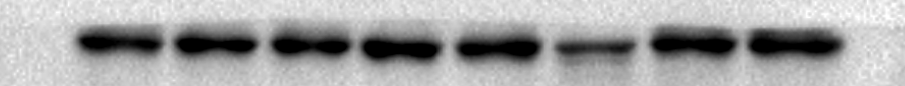

Supplement: Supplementary file 3 — Source data Fig. 1 [file 44319_2025_398_MOESM3_ESM.zip › Figure 1/Figure 1W/Western blot-actin.tif]

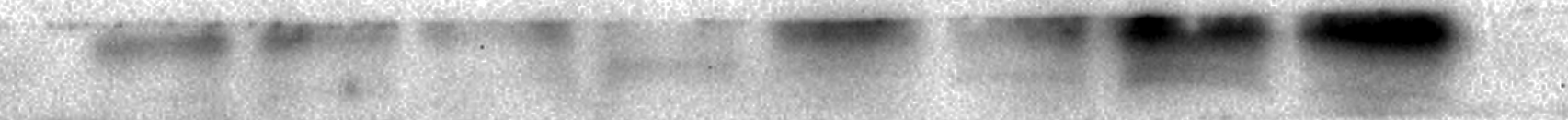

Supplement: Supplementary file 3 — Source data Fig. 1 [file 44319_2025_398_MOESM3_ESM.zip › Figure 1/Figure 1W/Western blot-adrb3.tif]

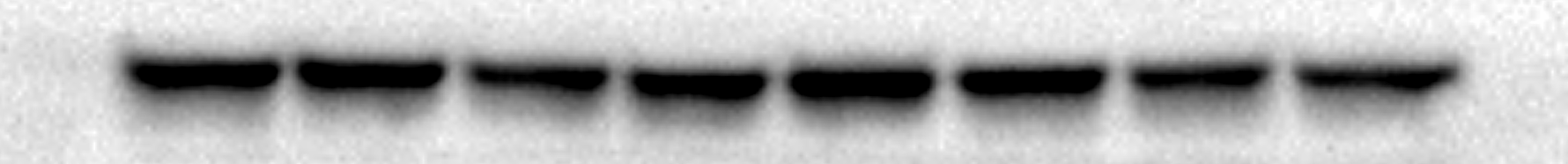

Supplement: Supplementary file 3 — Source data Fig. 1 [file 44319_2025_398_MOESM3_ESM.zip › Figure 1/Figure 1W/Western blot-hsl.tif]

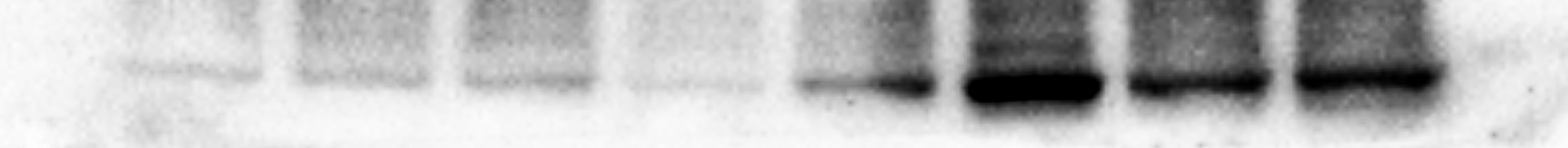

Supplement: Supplementary file 3 — Source data Fig. 1 [file 44319_2025_398_MOESM3_ESM.zip › Figure 1/Figure 1W/Western blot-p-hsl.tif]

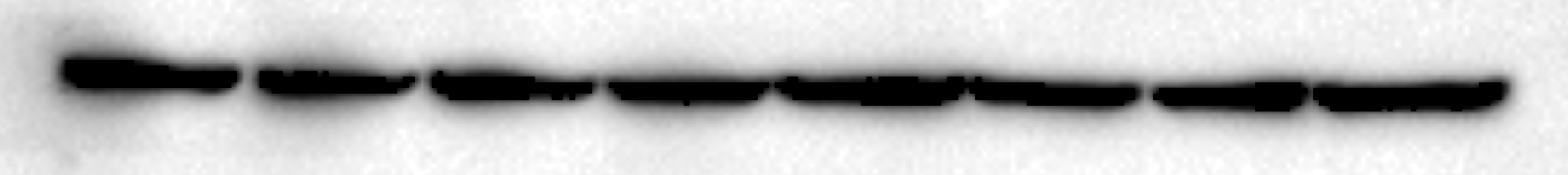

Supplement: Supplementary file 4 — Source data Fig. 2 [file 44319_2025_398_MOESM4_ESM.zip › Figure 2/Figure 2 A/Western blot-actin.tif]

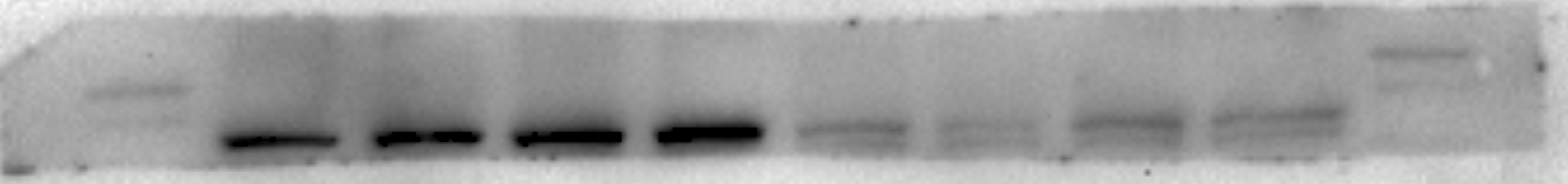

Supplement: Supplementary file 4 — Source data Fig. 2 [file 44319_2025_398_MOESM4_ESM.zip › Figure 2/Figure 2 A/Western blot-lgr4.tif]

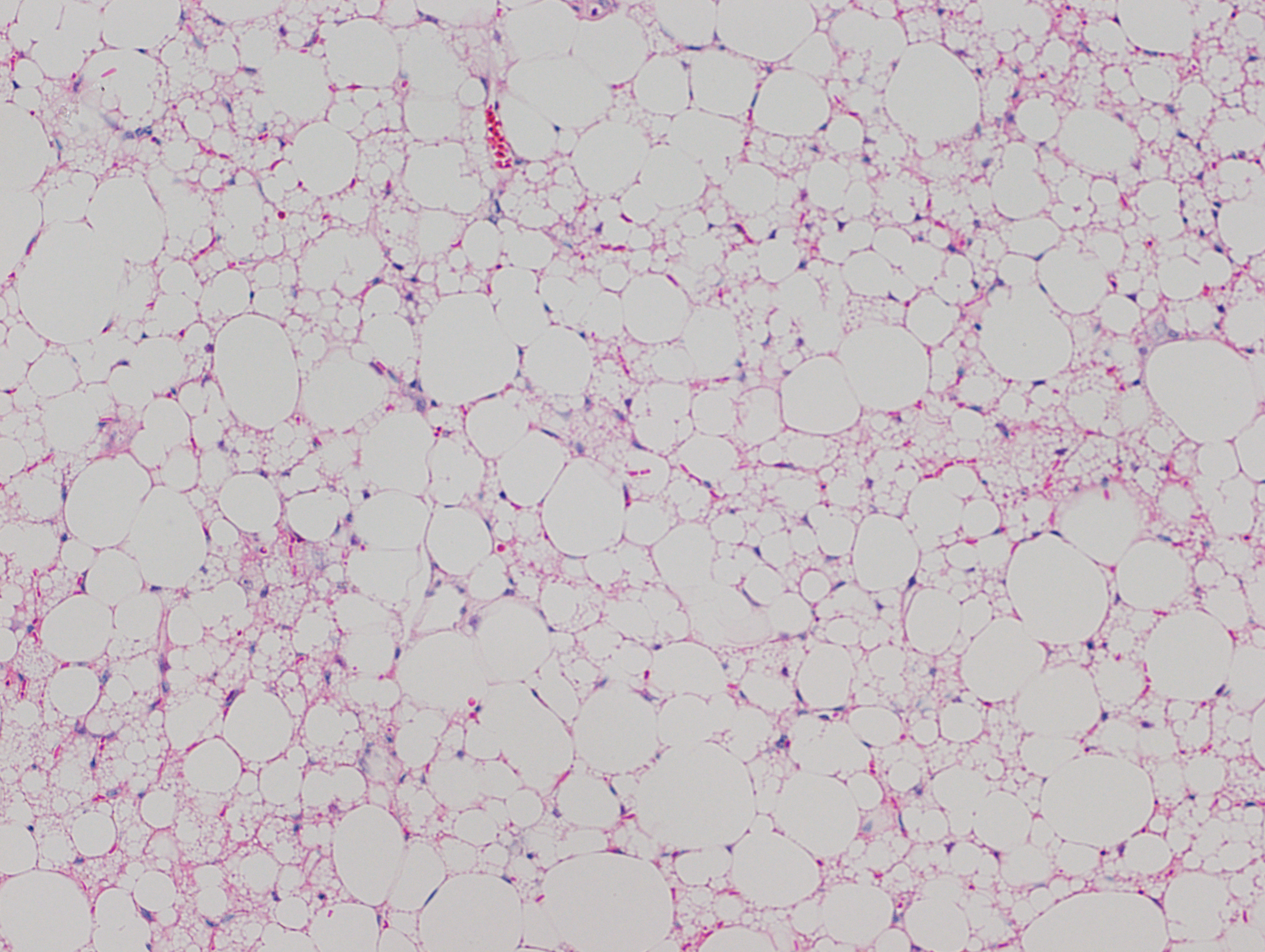

Supplement: Supplementary file 4 — Source data Fig. 2 [file 44319_2025_398_MOESM4_ESM.zip › Figure 2/Figure 2 O/BAT-FF.tif]

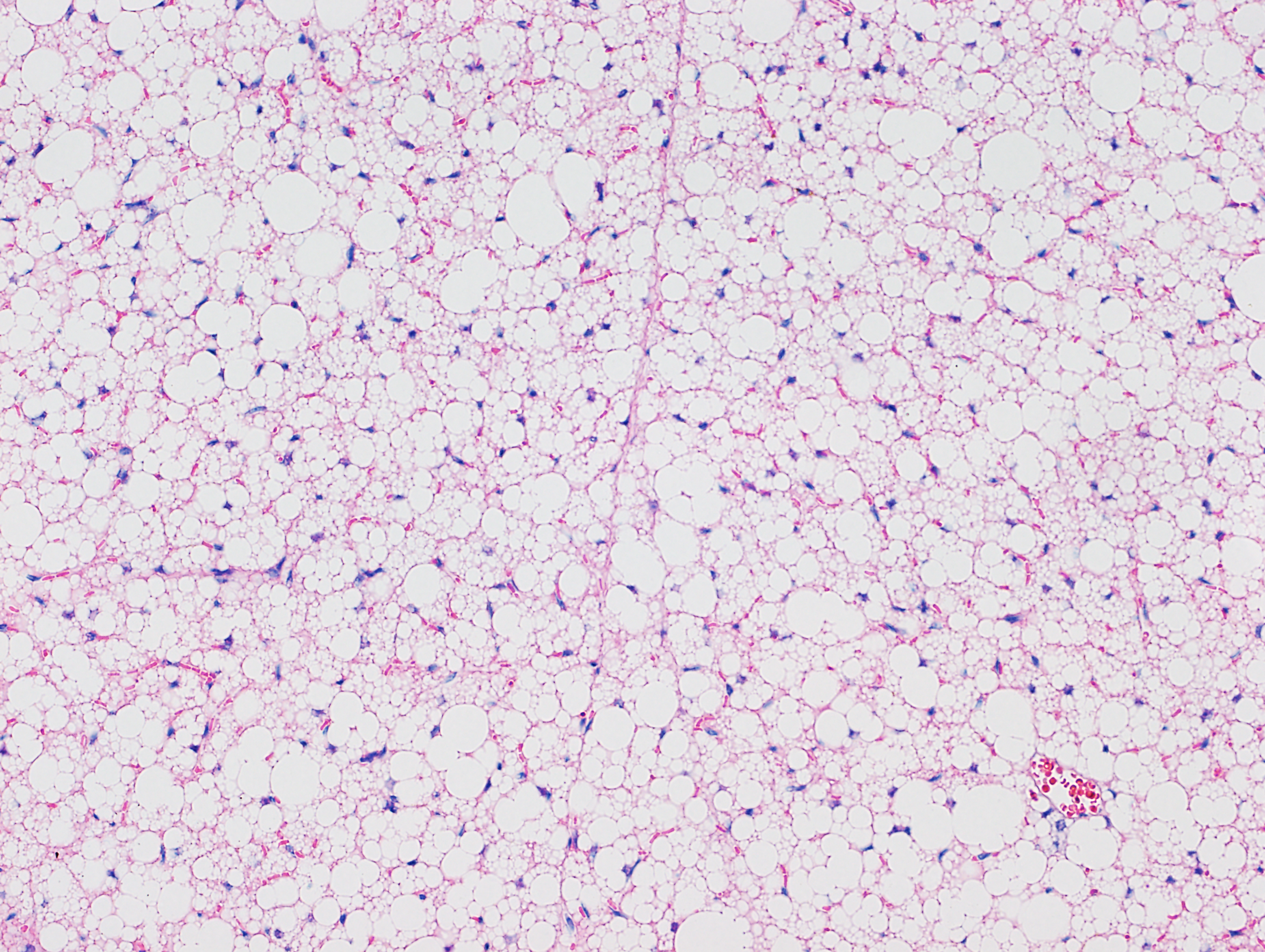

Supplement: Supplementary file 4 — Source data Fig. 2 [file 44319_2025_398_MOESM4_ESM.zip › Figure 2/Figure 2 O/BAT-KO.tif]

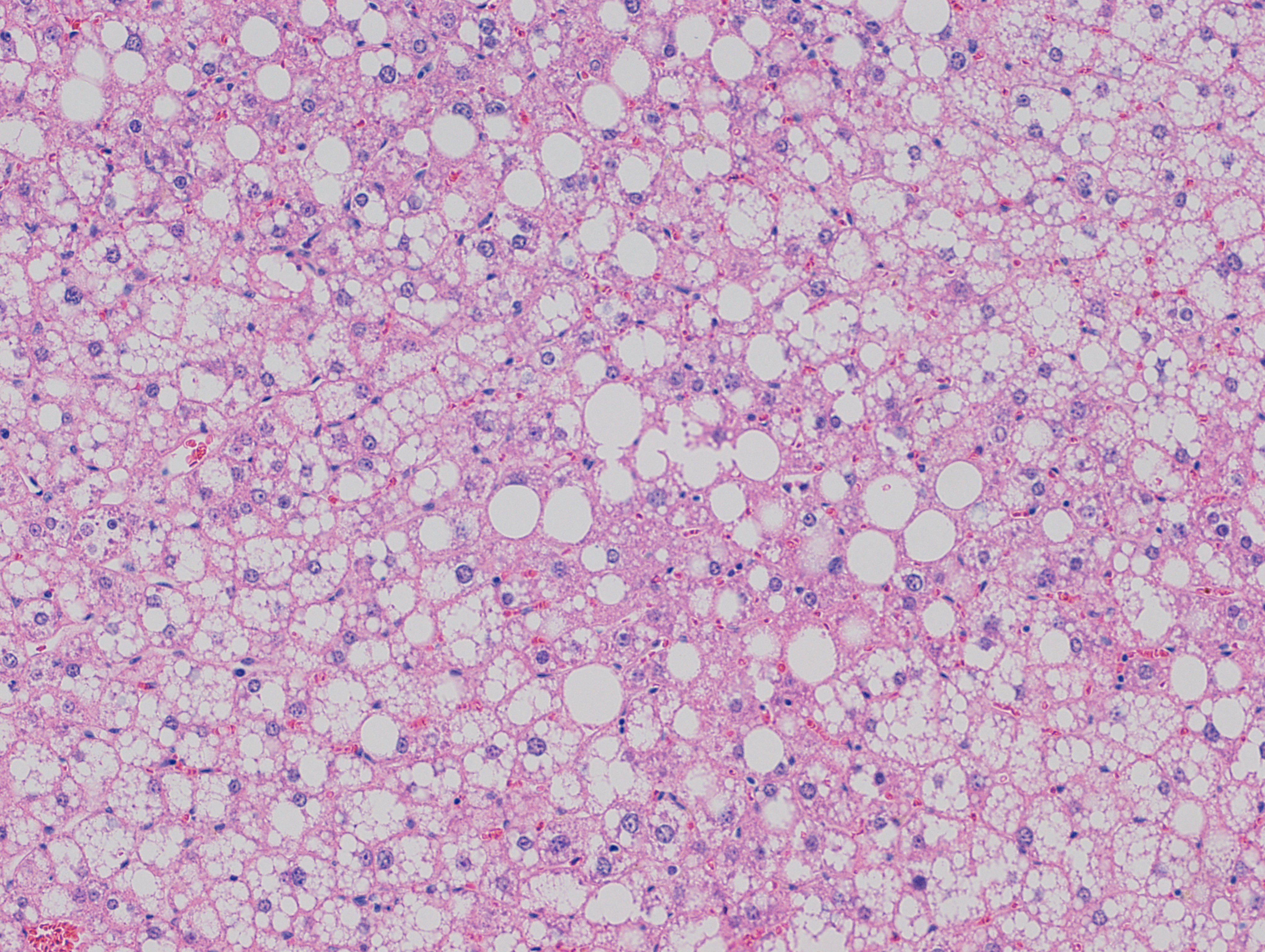

Supplement: Supplementary file 4 — Source data Fig. 2 [file 44319_2025_398_MOESM4_ESM.zip › Figure 2/Figure 2 O/Liver-FF.tif]

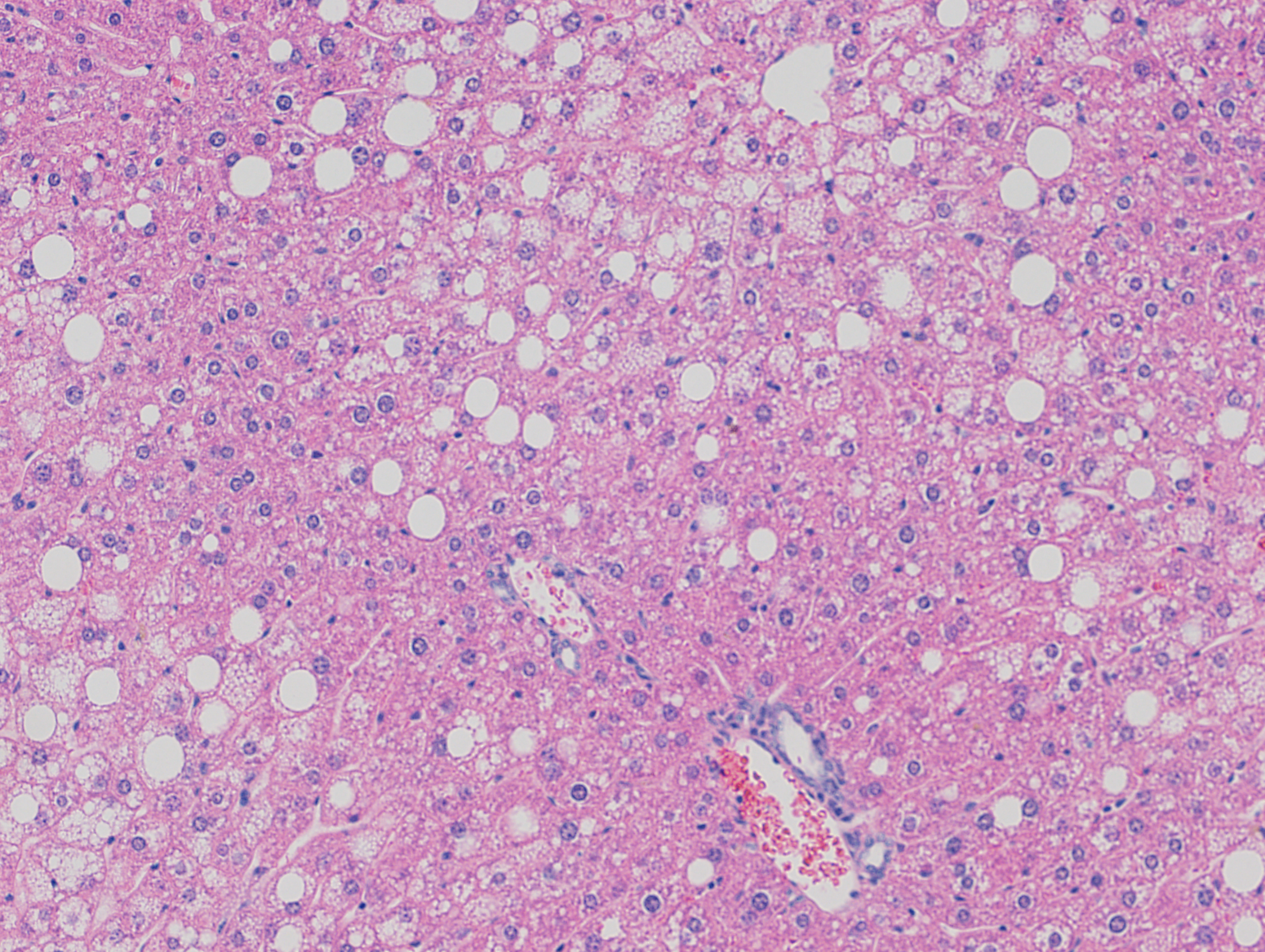

Supplement: Supplementary file 4 — Source data Fig. 2 [file 44319_2025_398_MOESM4_ESM.zip › Figure 2/Figure 2 O/Liver-KO.tif]

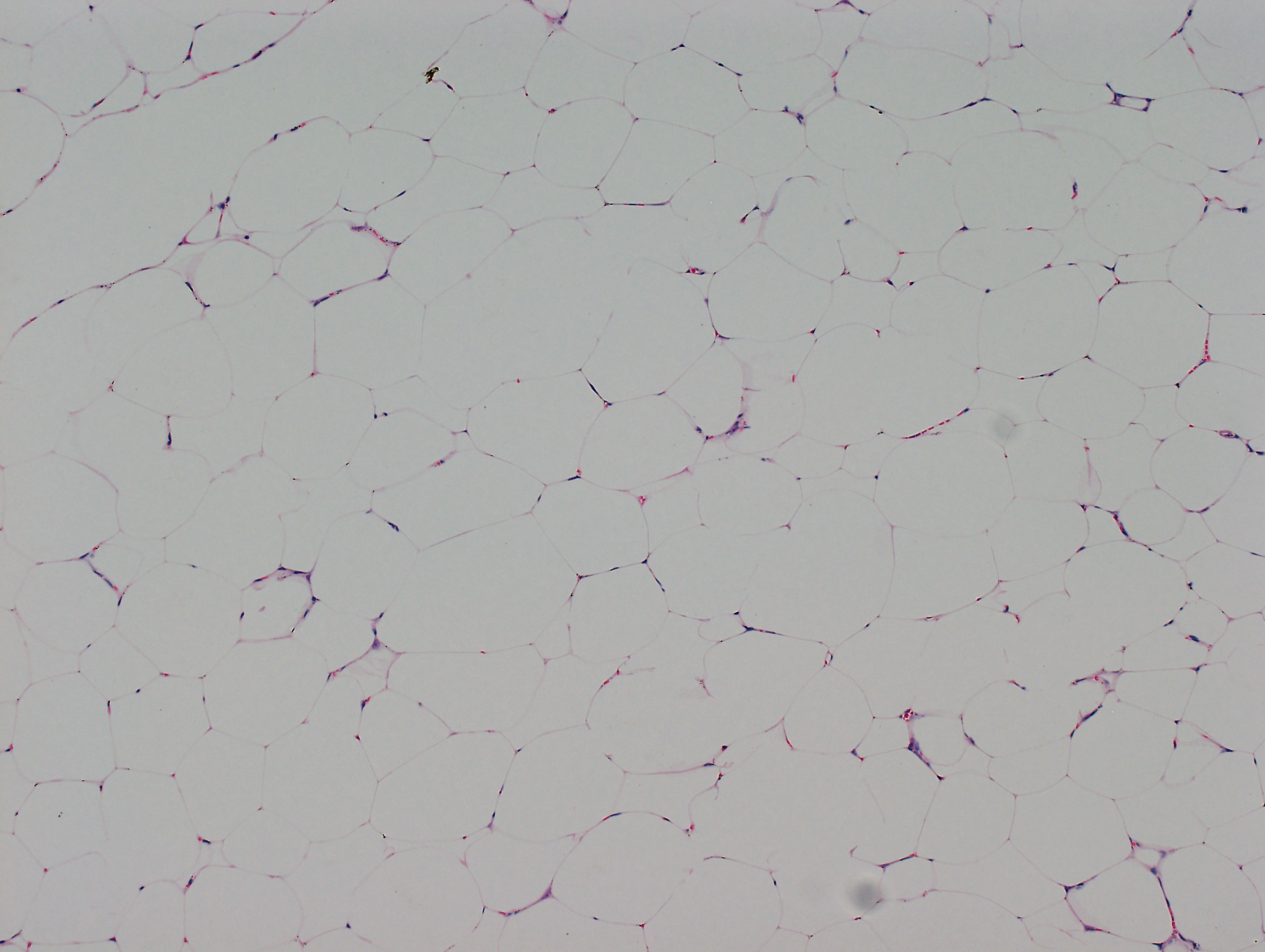

Supplement: Supplementary file 4 — Source data Fig. 2 [file 44319_2025_398_MOESM4_ESM.zip › Figure 2/Figure 2 O/SAT-FF.tif]

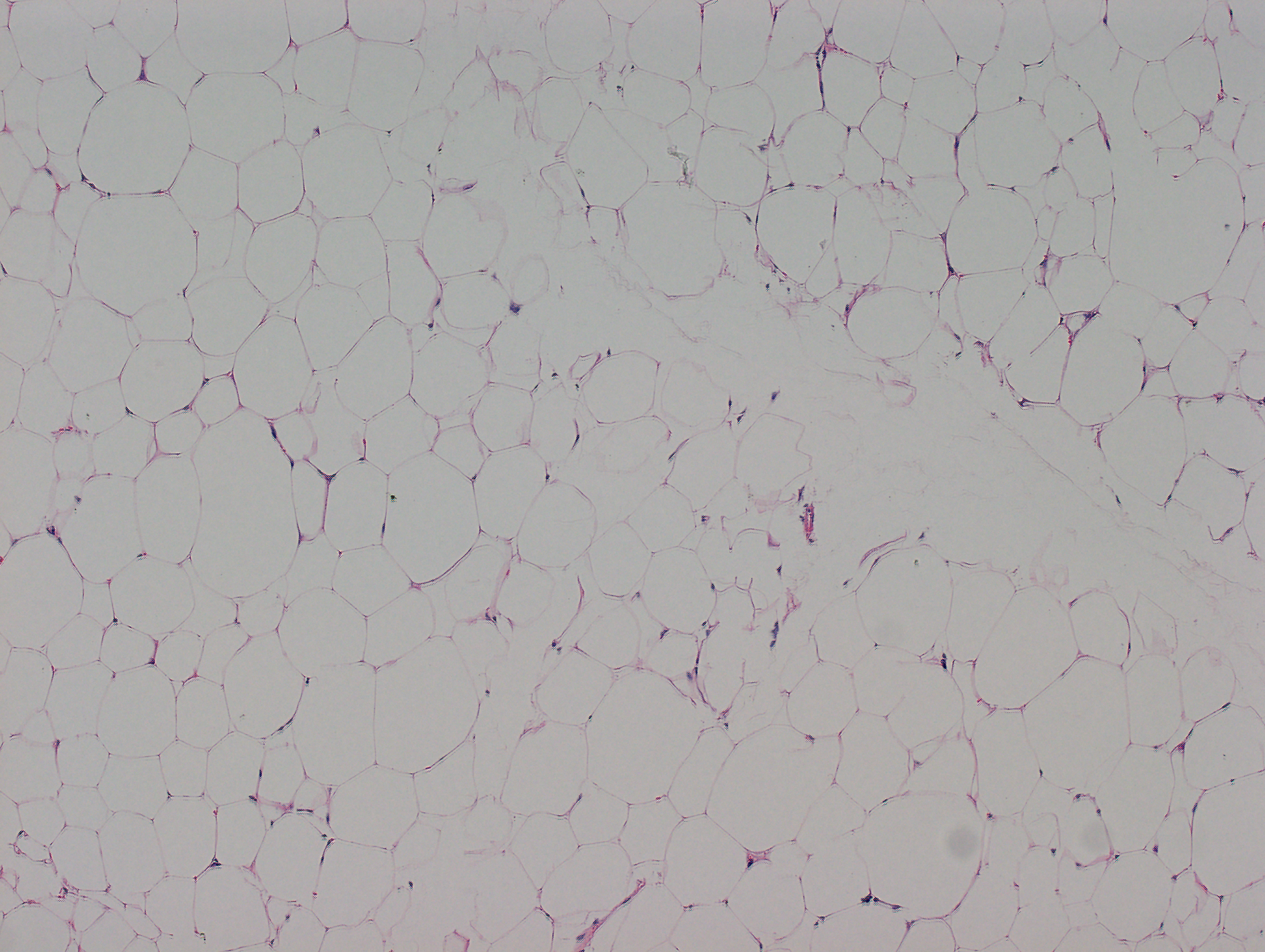

Supplement: Supplementary file 4 — Source data Fig. 2 [file 44319_2025_398_MOESM4_ESM.zip › Figure 2/Figure 2 O/SAT-KO.tif]

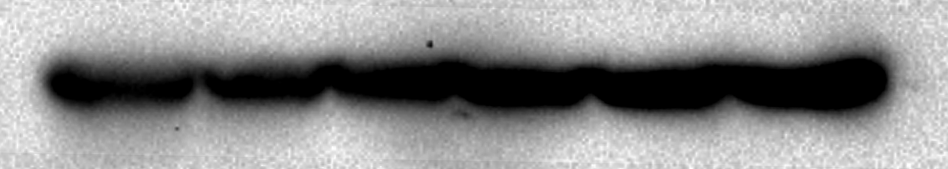

Supplement: Supplementary file 4 — Source data Fig. 2 [file 44319_2025_398_MOESM4_ESM.zip › Figure 2/Figure 2 S/Western blot-Actin.tif]

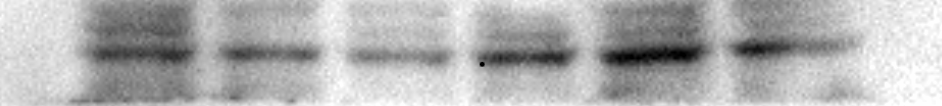

Supplement: Supplementary file 4 — Source data Fig. 2 [file 44319_2025_398_MOESM4_ESM.zip › Figure 2/Figure 2 S/Western blot-Adrb3.tif]

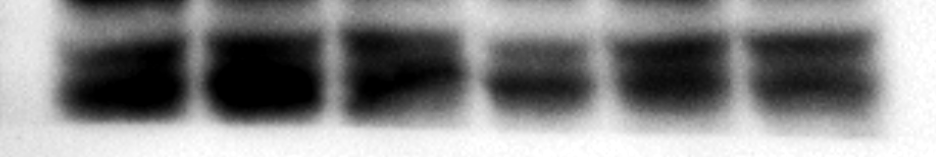

Supplement: Supplementary file 4 — Source data Fig. 2 [file 44319_2025_398_MOESM4_ESM.zip › Figure 2/Figure 2 S/Western blot-Hsl.tif]

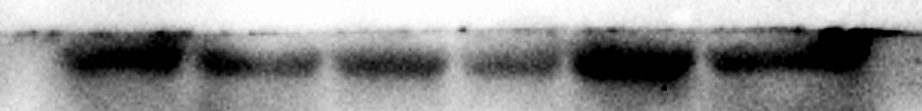

Supplement: Supplementary file 4 — Source data Fig. 2 [file 44319_2025_398_MOESM4_ESM.zip › Figure 2/Figure 2 S/Western blot-pHsl.tif]

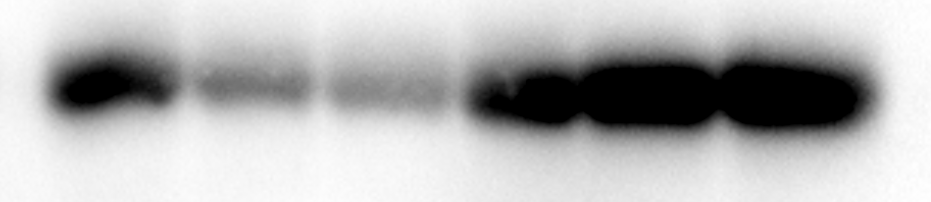

Supplement: Supplementary file 4 — Source data Fig. 2 [file 44319_2025_398_MOESM4_ESM.zip › Figure 2/Figure 2 S/Western blot-Ucp-1.tif]

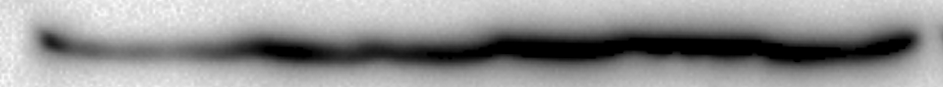

Supplement: Supplementary file 5 — Source data Fig. 3 [file 44319_2025_398_MOESM5_ESM.zip › Figure 3/Figure 3A/Western blot-actin.tif]

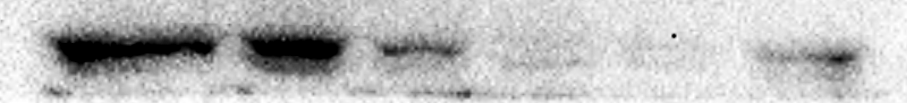

Supplement: Supplementary file 5 — Source data Fig. 3 [file 44319_2025_398_MOESM5_ESM.zip › Figure 3/Figure 3A/Western blot-Lgr4.tif]

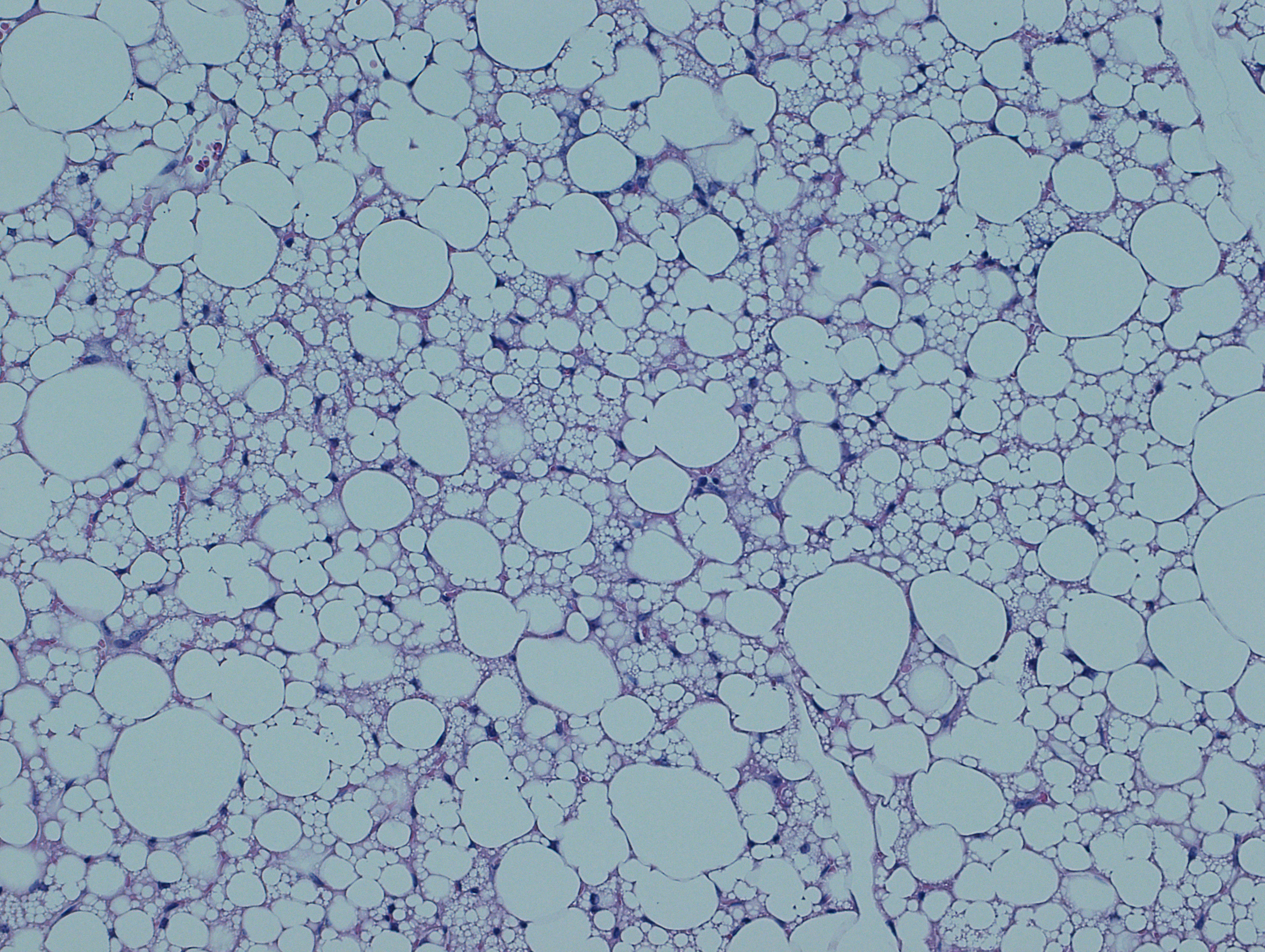

Supplement: Supplementary file 5 — Source data Fig. 3 [file 44319_2025_398_MOESM5_ESM.zip › Figure 3/Figure 3O/BAT-FF.tif]

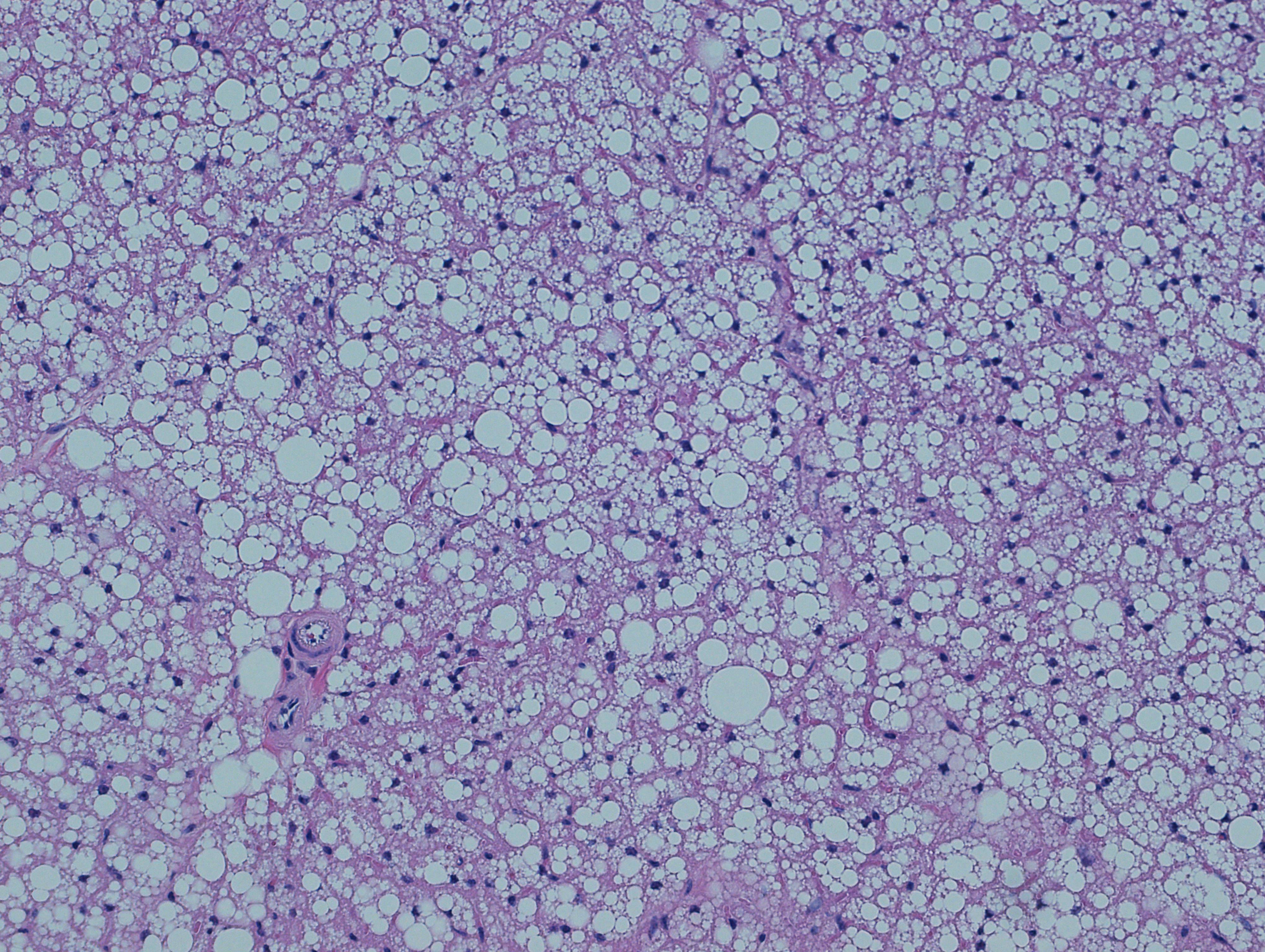

Supplement: Supplementary file 5 — Source data Fig. 3 [file 44319_2025_398_MOESM5_ESM.zip › Figure 3/Figure 3O/BAT-KO.tif]

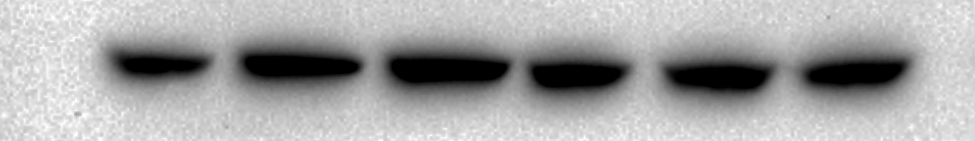

Supplement: Supplementary file 5 — Source data Fig. 3 [file 44319_2025_398_MOESM5_ESM.zip › Figure 3/Figure 3P/Western blot-Actin.tif]

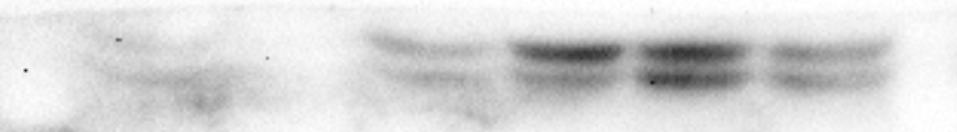

Supplement: Supplementary file 5 — Source data Fig. 3 [file 44319_2025_398_MOESM5_ESM.zip › Figure 3/Figure 3P/Western blot-Adrb3.tif]

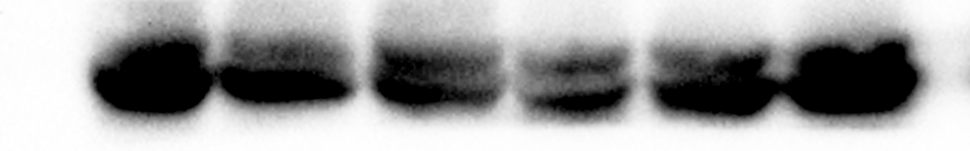

Supplement: Supplementary file 5 — Source data Fig. 3 [file 44319_2025_398_MOESM5_ESM.zip › Figure 3/Figure 3P/Western blot-Hsl.tif]

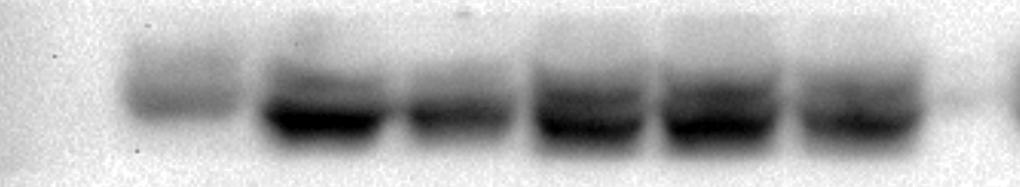

Supplement: Supplementary file 5 — Source data Fig. 3 [file 44319_2025_398_MOESM5_ESM.zip › Figure 3/Figure 3P/Western blot-p-Hsl.tif]

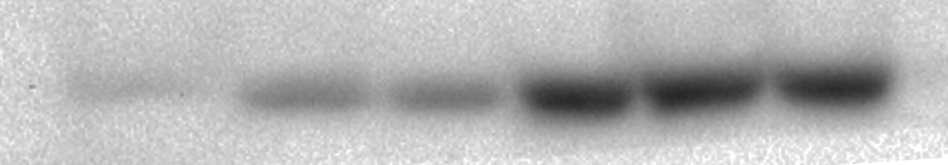

Supplement: Supplementary file 5 — Source data Fig. 3 [file 44319_2025_398_MOESM5_ESM.zip › Figure 3/Figure 3P/Western blot-Ucp-1.tif]

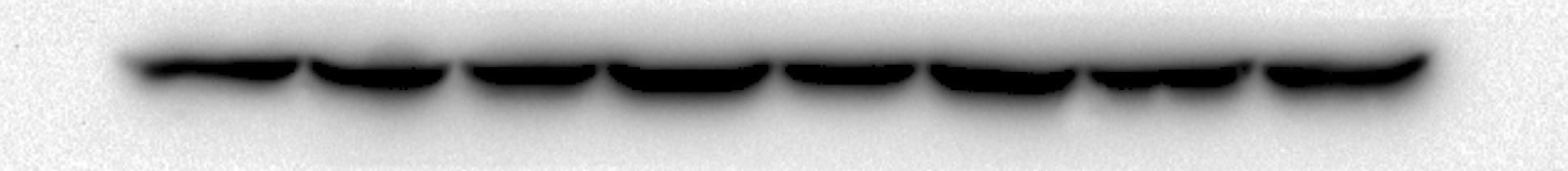

Supplement: Supplementary file 6 — Source data Fig. 4 [file 44319_2025_398_MOESM6_ESM.zip › Figure 4/Figure 4 A/Western blot-Actin.tif]

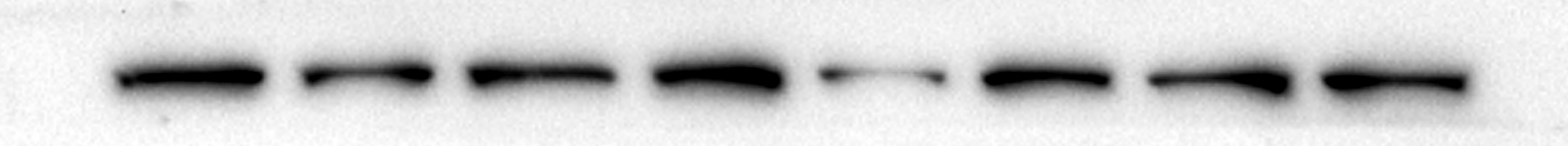

Supplement: Supplementary file 6 — Source data Fig. 4 [file 44319_2025_398_MOESM6_ESM.zip › Figure 4/Figure 4 A/Western blot-Lgr4.tif]

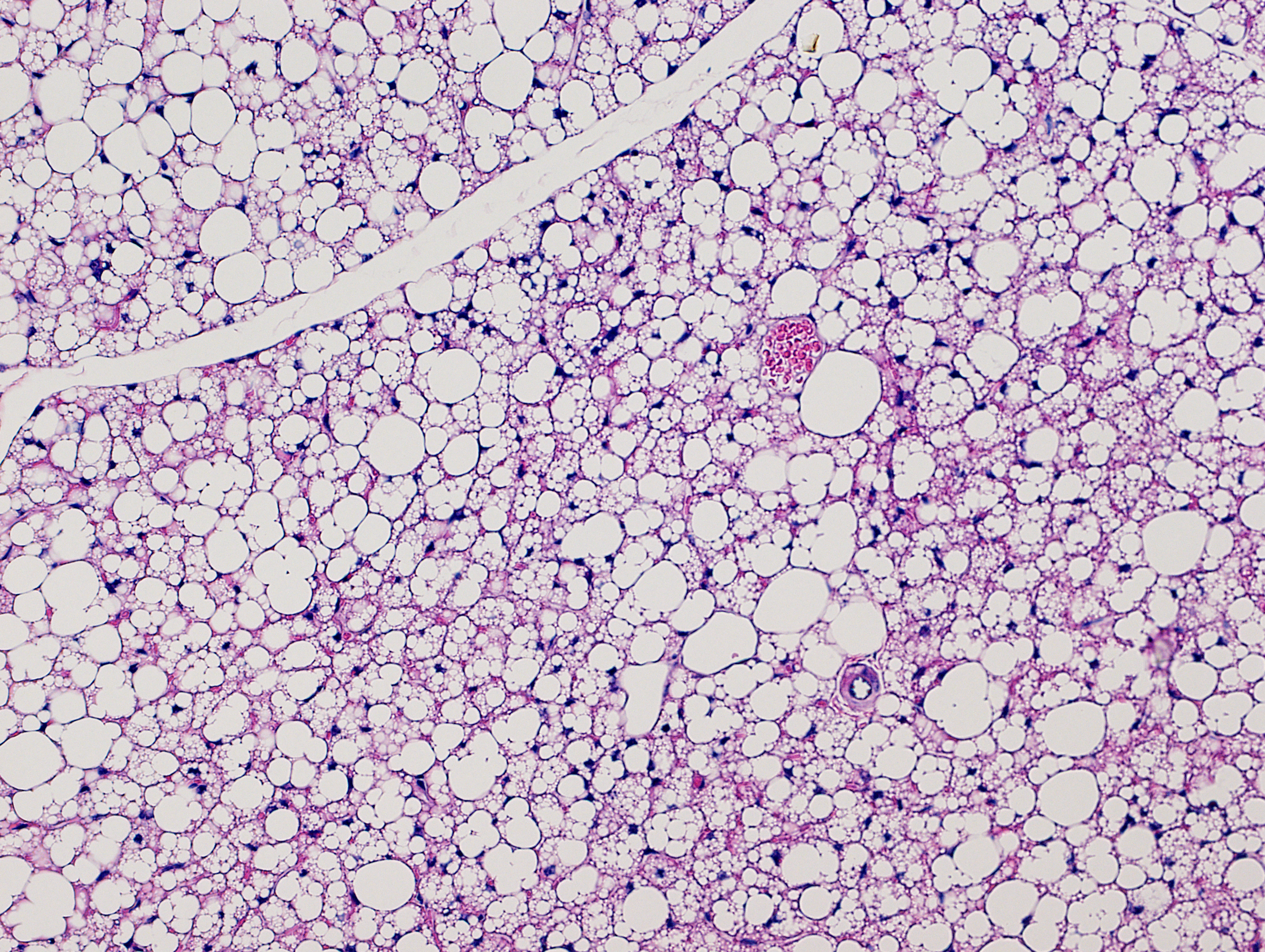

Supplement: Supplementary file 6 — Source data Fig. 4 [file 44319_2025_398_MOESM6_ESM.zip › Figure 4/Figure 4 O/BAT-FF.tif]

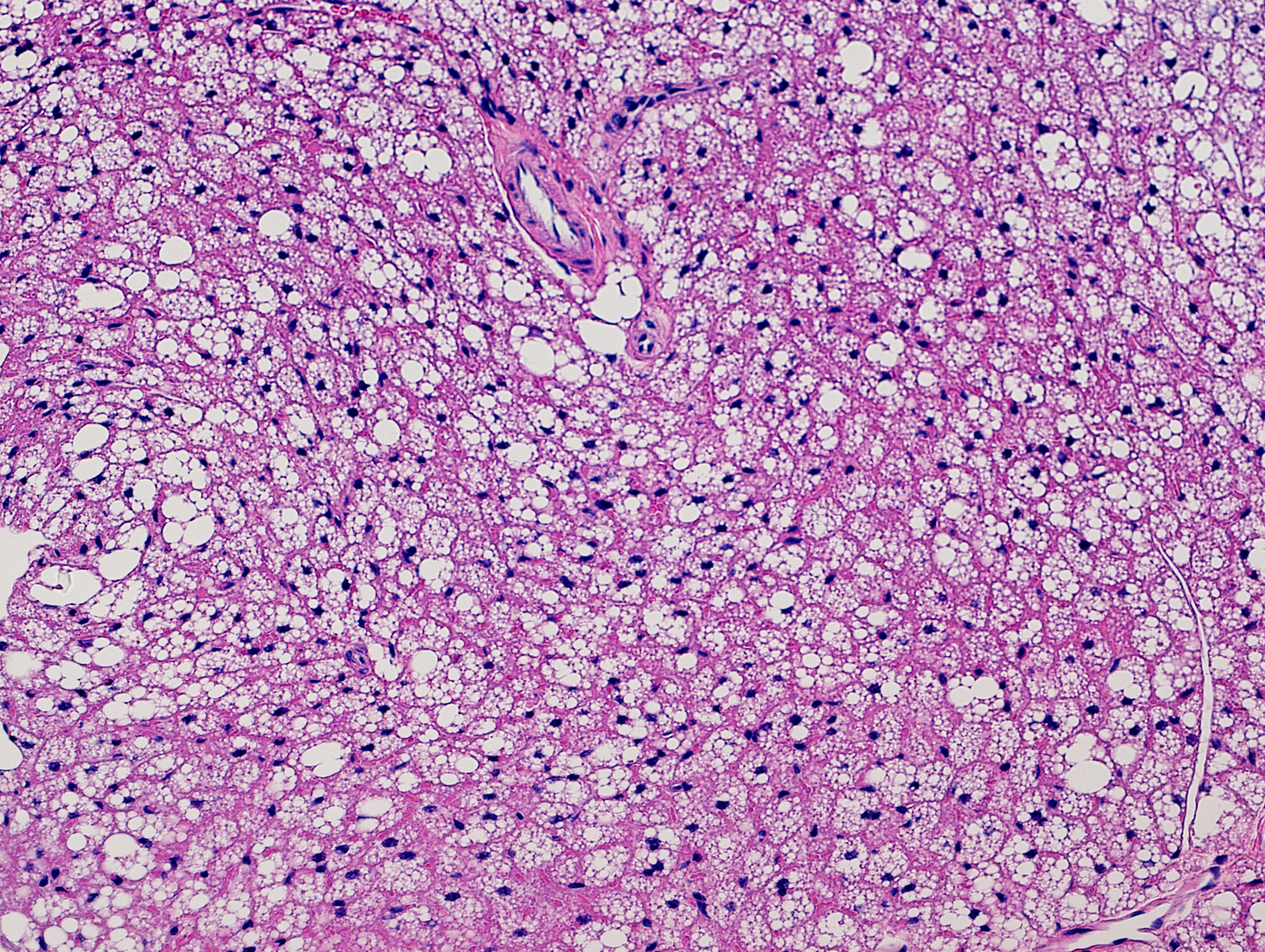

Supplement: Supplementary file 6 — Source data Fig. 4 [file 44319_2025_398_MOESM6_ESM.zip › Figure 4/Figure 4 O/BAT-KO.tif]

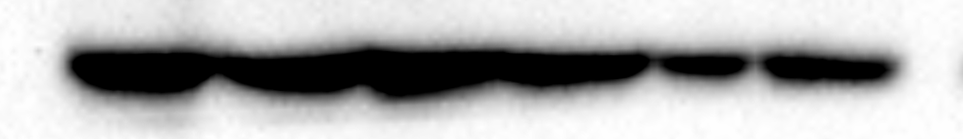

Supplement: Supplementary file 6 — Source data Fig. 4 [file 44319_2025_398_MOESM6_ESM.zip › Figure 4/Figure 4 P/Western blot-Actin.tif]

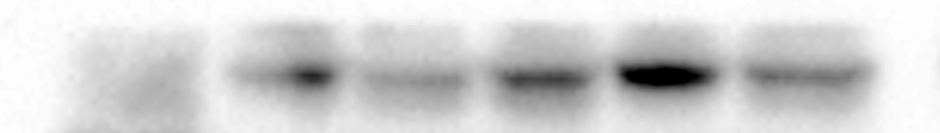

Supplement: Supplementary file 6 — Source data Fig. 4 [file 44319_2025_398_MOESM6_ESM.zip › Figure 4/Figure 4 P/Western blot-Adrb3.tif]

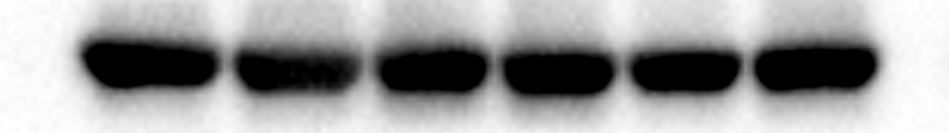

Supplement: Supplementary file 6 — Source data Fig. 4 [file 44319_2025_398_MOESM6_ESM.zip › Figure 4/Figure 4 P/Western blot-Hsl.tif]

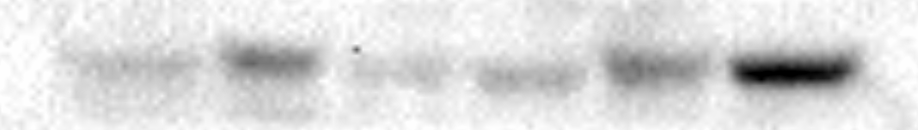

Supplement: Supplementary file 6 — Source data Fig. 4 [file 44319_2025_398_MOESM6_ESM.zip › Figure 4/Figure 4 P/Western blot-p-Hsl.tif]

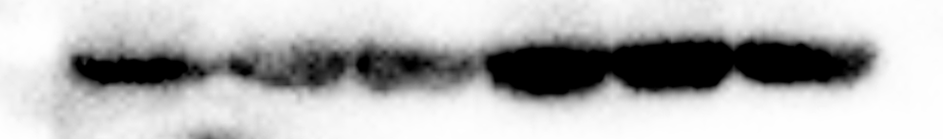

Supplement: Supplementary file 6 — Source data Fig. 4 [file 44319_2025_398_MOESM6_ESM.zip › Figure 4/Figure 4 P/Western blot-Ucp-1.tif]

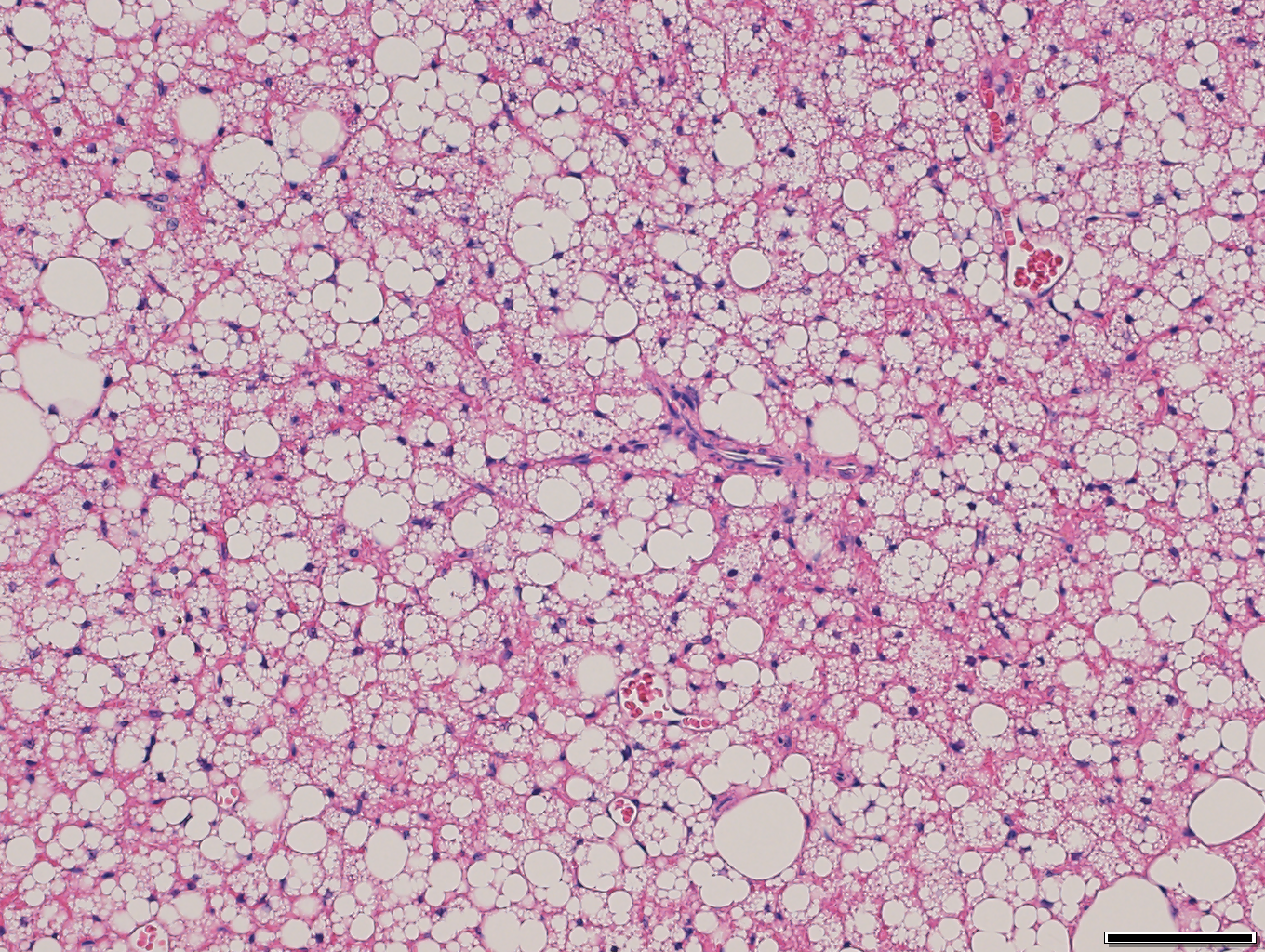

Supplement: Supplementary file 7 — Source data Fig. 5 [file 44319_2025_398_MOESM7_ESM.zip › Figure 5/Figure 5 M/FF-Sham.tif]

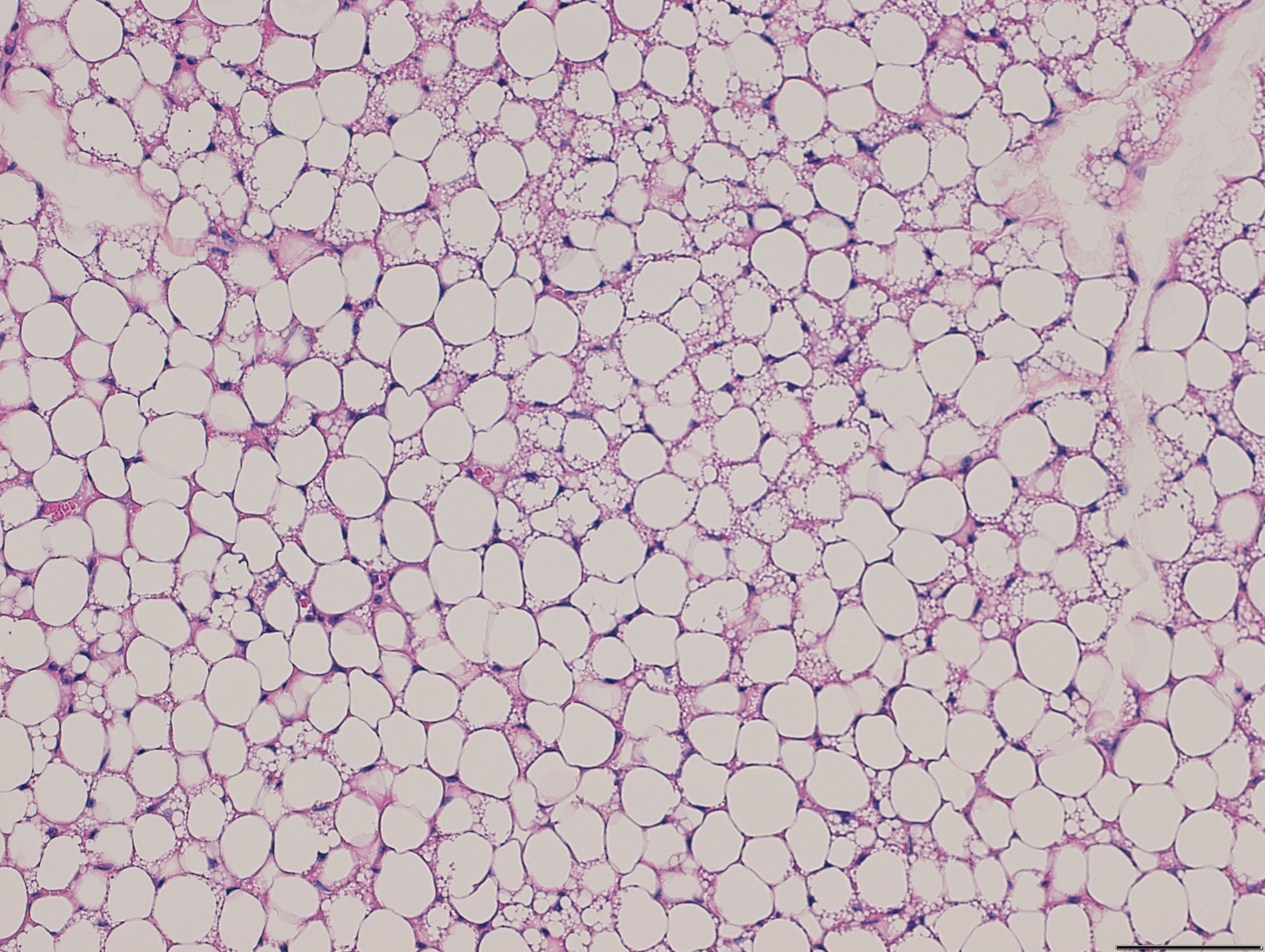

Supplement: Supplementary file 7 — Source data Fig. 5 [file 44319_2025_398_MOESM7_ESM.zip › Figure 5/Figure 5 M/FF-Surgery.tif]

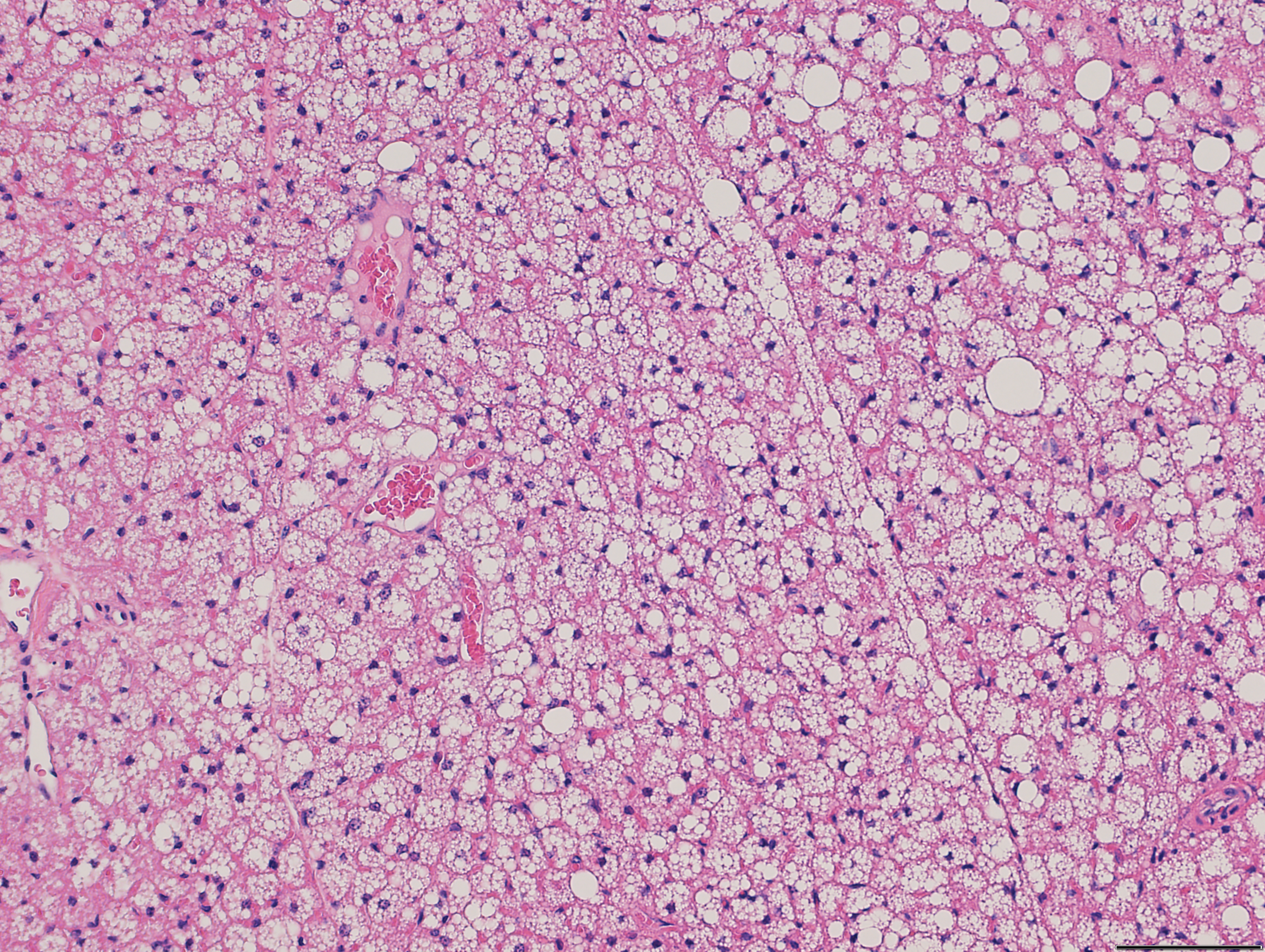

Supplement: Supplementary file 7 — Source data Fig. 5 [file 44319_2025_398_MOESM7_ESM.zip › Figure 5/Figure 5 M/KO-Sham.tif]

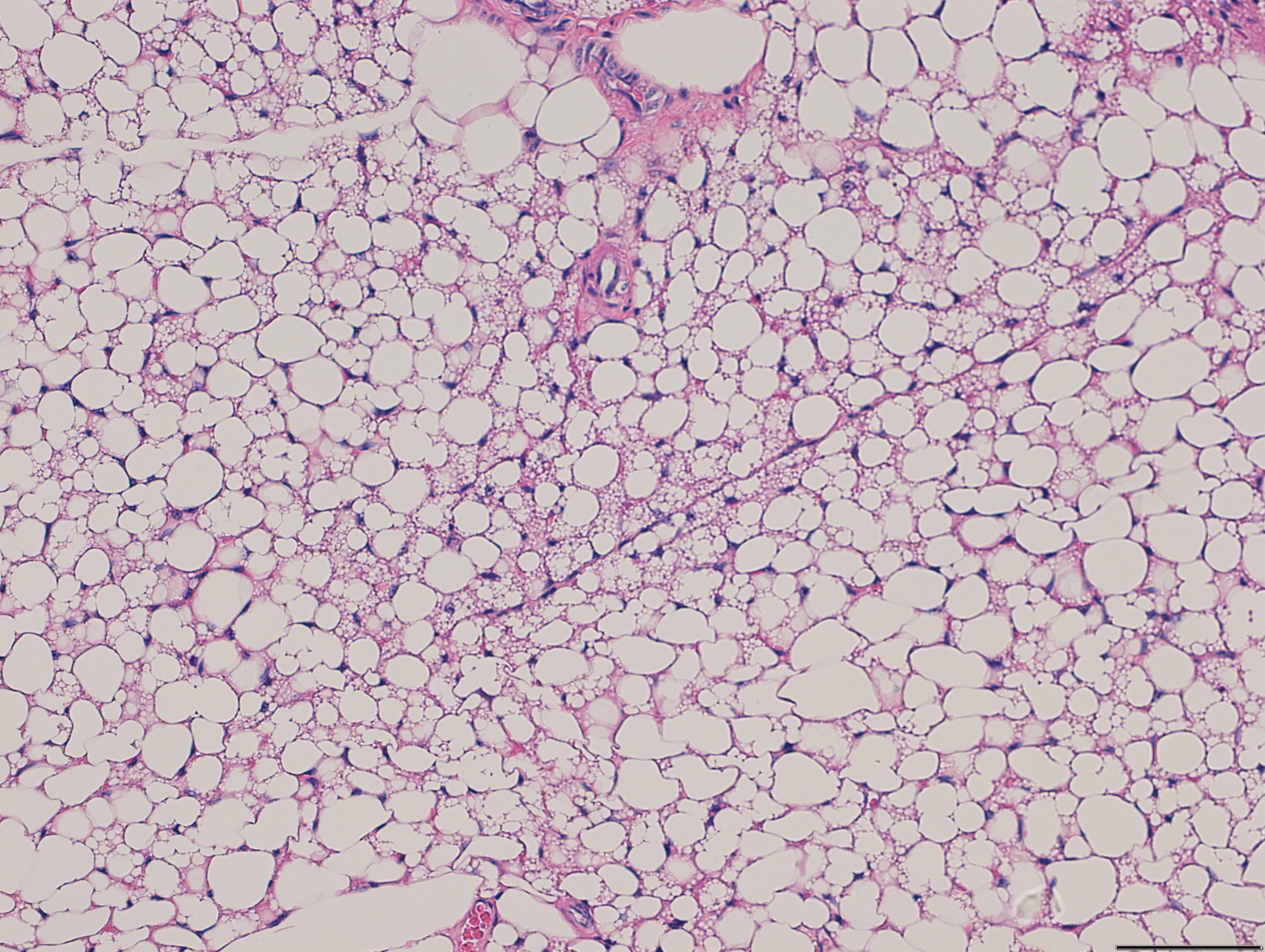

Supplement: Supplementary file 7 — Source data Fig. 5 [file 44319_2025_398_MOESM7_ESM.zip › Figure 5/Figure 5 M/KO-Surgery.tif]

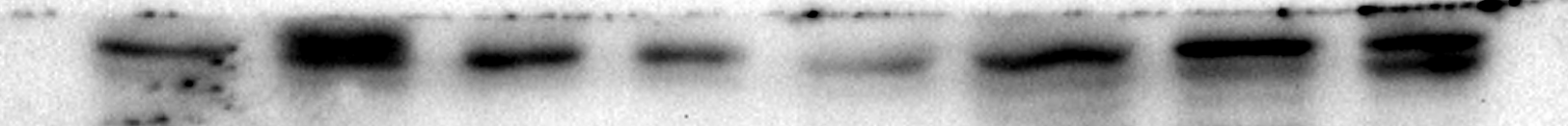

Supplement: Supplementary file 7 — Source data Fig. 5 [file 44319_2025_398_MOESM7_ESM.zip › Figure 5/Figure 5 N/Western blot-Actin.tif]

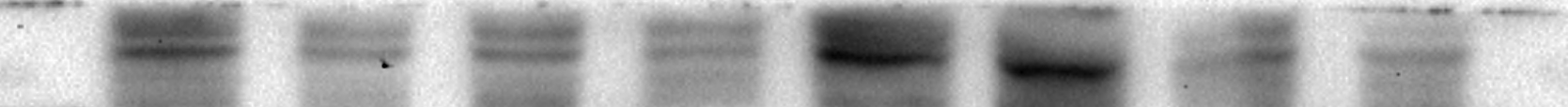

Supplement: Supplementary file 7 — Source data Fig. 5 [file 44319_2025_398_MOESM7_ESM.zip › Figure 5/Figure 5 N/Western blot-Adrb3.tif]

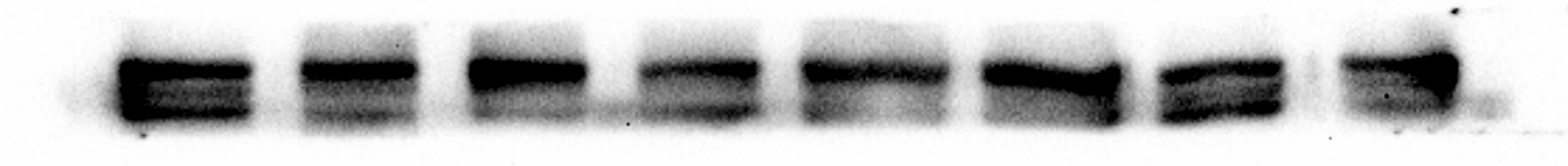

Supplement: Supplementary file 7 — Source data Fig. 5 [file 44319_2025_398_MOESM7_ESM.zip › Figure 5/Figure 5 N/Western blot-Hsl.tif]

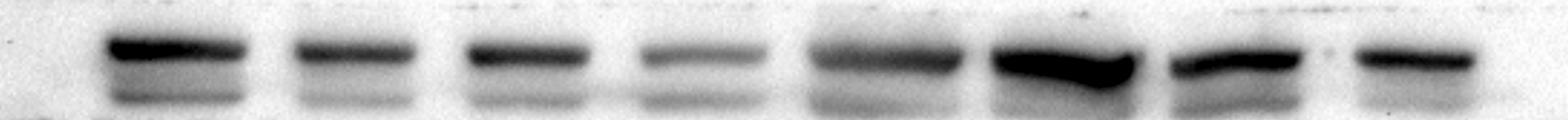

Supplement: Supplementary file 7 — Source data Fig. 5 [file 44319_2025_398_MOESM7_ESM.zip › Figure 5/Figure 5 N/Western blot-pHsl.tif]

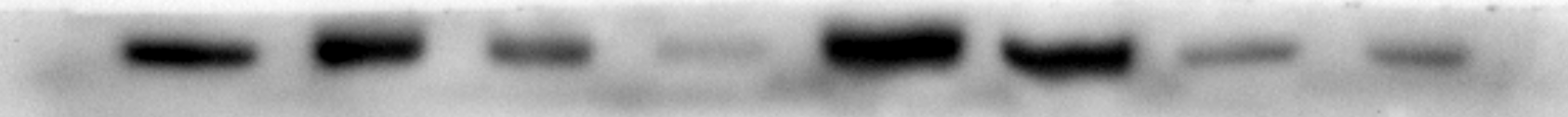

Supplement: Supplementary file 7 — Source data Fig. 5 [file 44319_2025_398_MOESM7_ESM.zip › Figure 5/Figure 5 N/Western blot-Ucp-1.tif]

Corresponding uncropped images

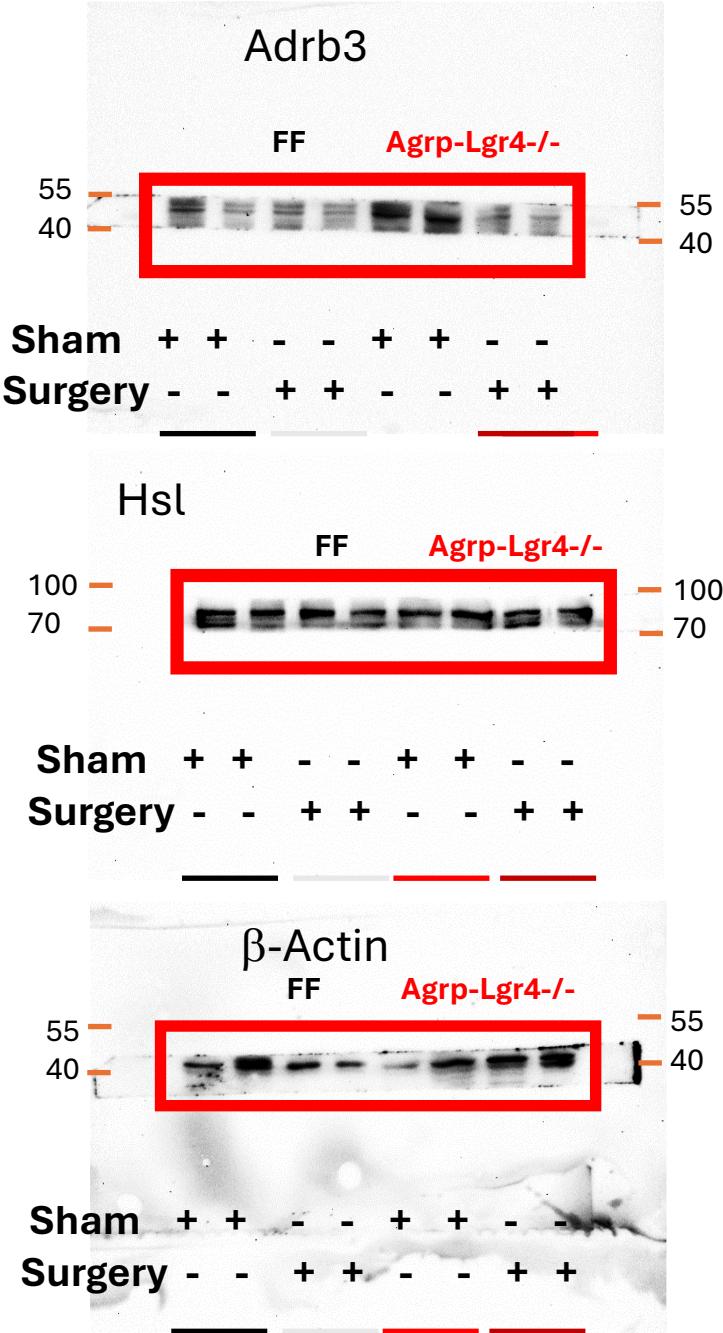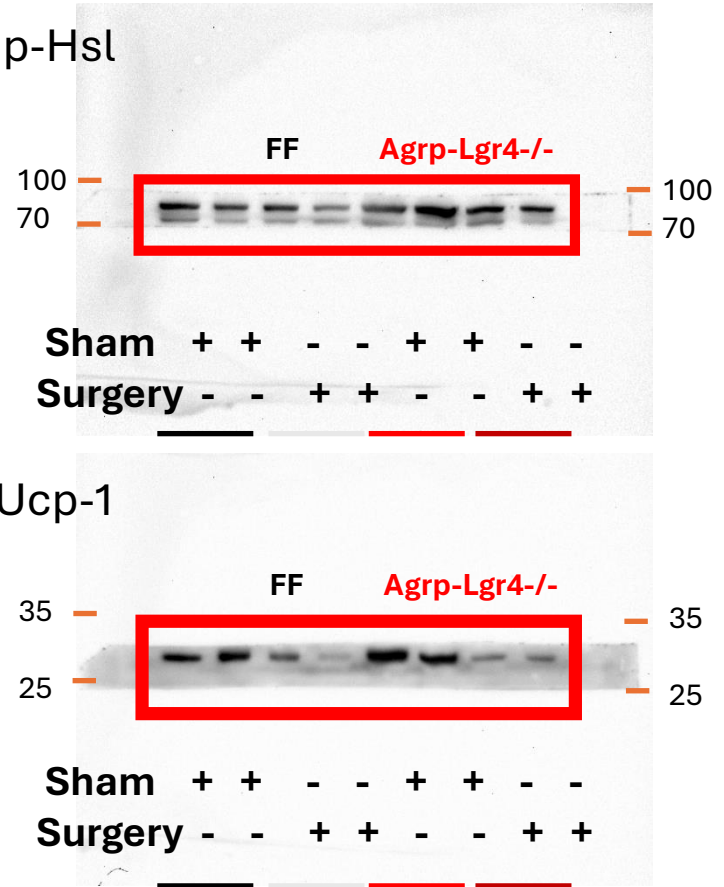

Supplement: Supplementary file 7 — Source data Fig. 5 [file 44319_2025_398_MOESM7_ESM.zip › Figure 5/Uncropped Western Blots of Figure 5.pdf]

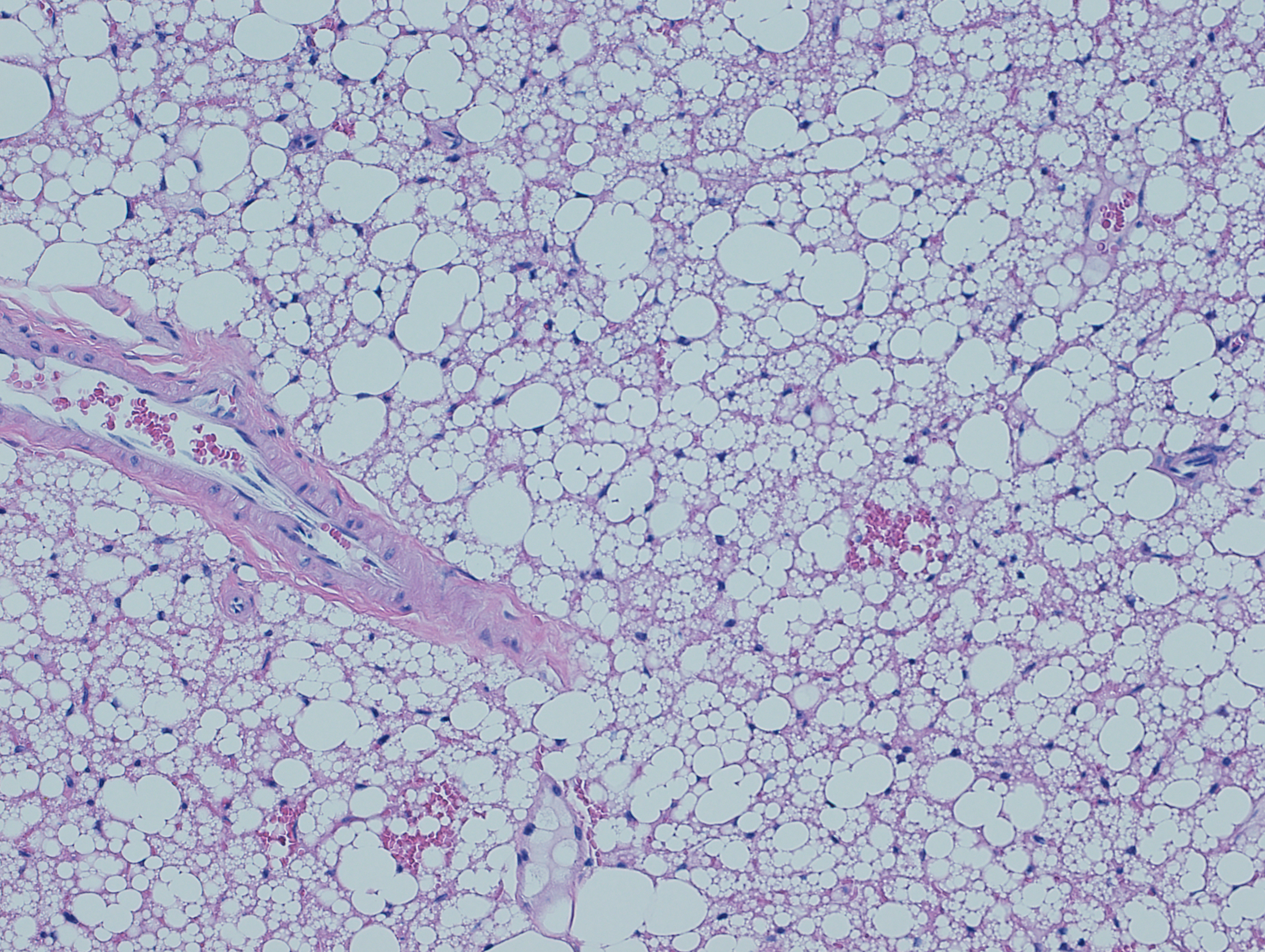

Supplement: Supplementary file 8 — Source data Fig. 6 [file 44319_2025_398_MOESM8_ESM.zip › Figure 6/Figure 6 H/FF-Sham.tif]

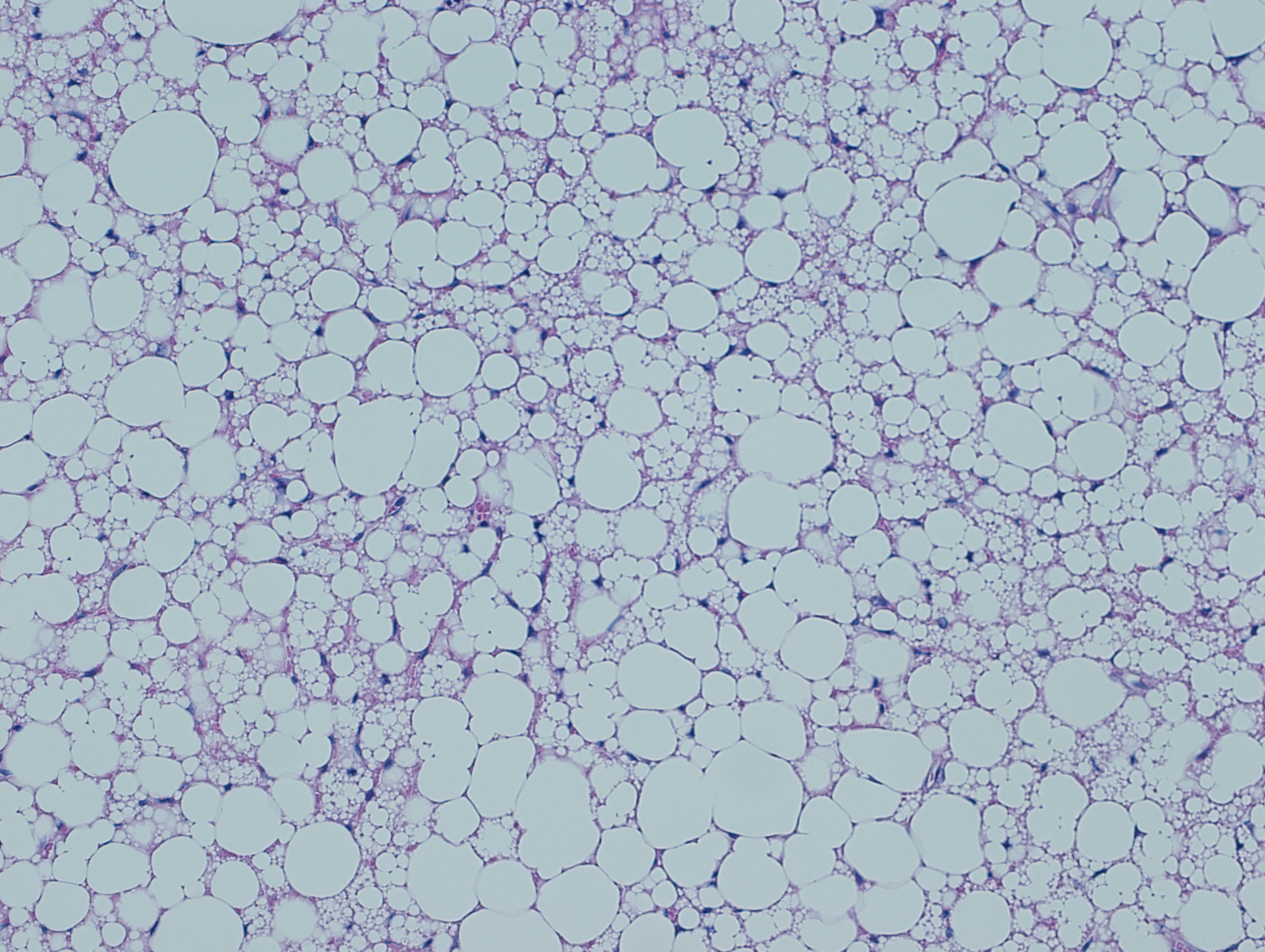

Supplement: Supplementary file 8 — Source data Fig. 6 [file 44319_2025_398_MOESM8_ESM.zip › Figure 6/Figure 6 H/FF-Surgery.tif]

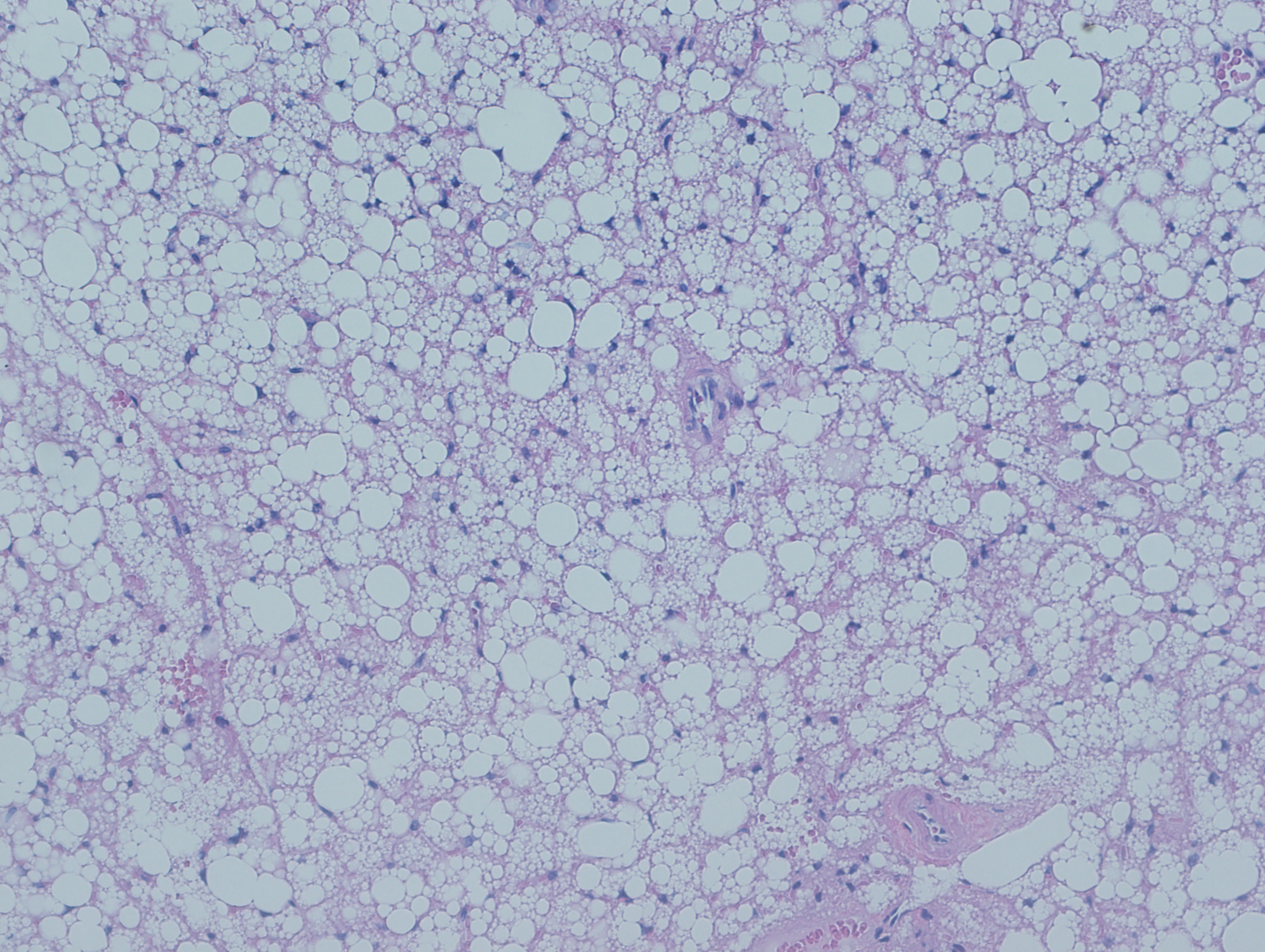

Supplement: Supplementary file 8 — Source data Fig. 6 [file 44319_2025_398_MOESM8_ESM.zip › Figure 6/Figure 6 H/KO-Sham.tif]

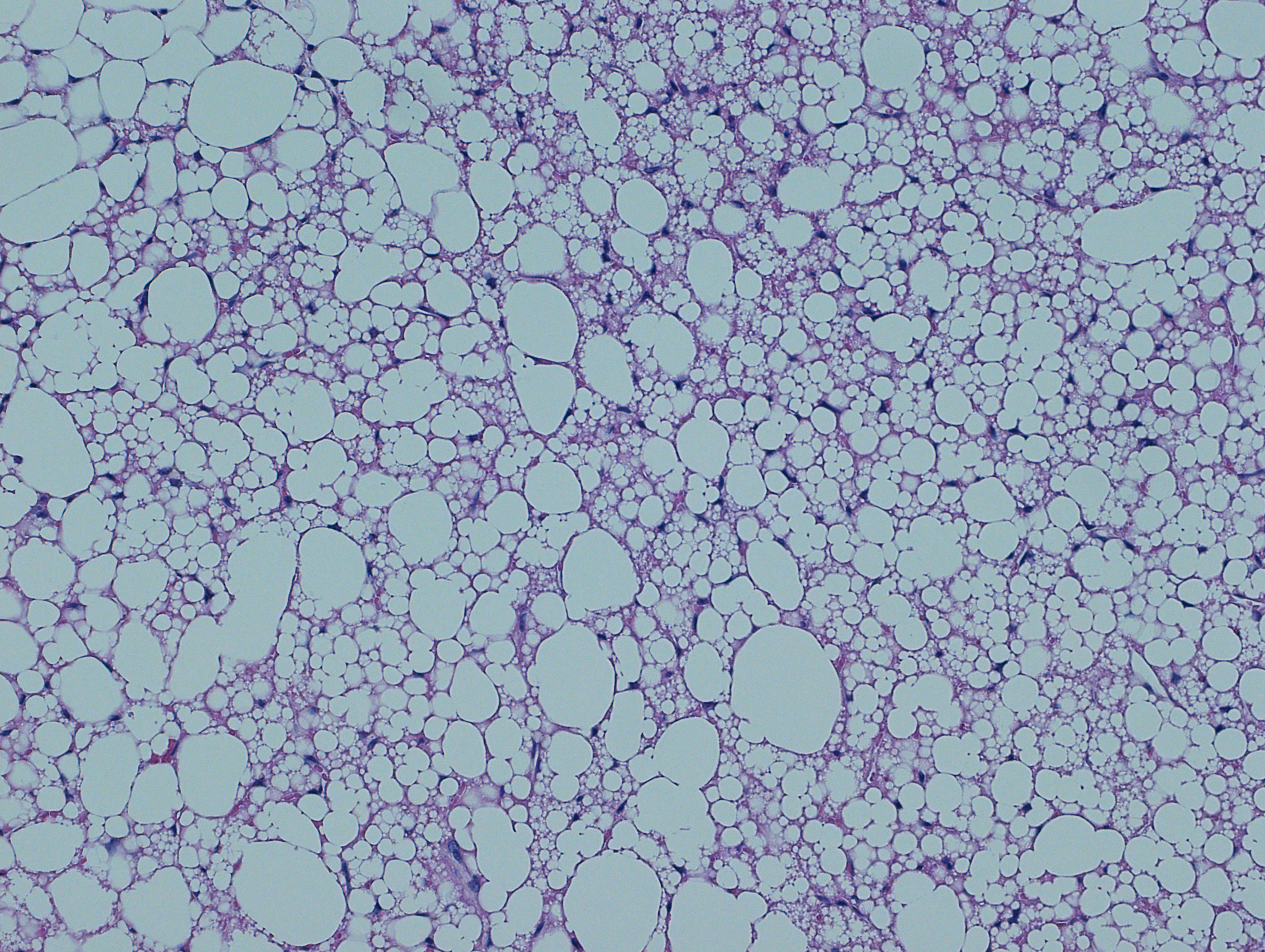

Supplement: Supplementary file 8 — Source data Fig. 6 [file 44319_2025_398_MOESM8_ESM.zip › Figure 6/Figure 6 H/KO-Surgery.tif]

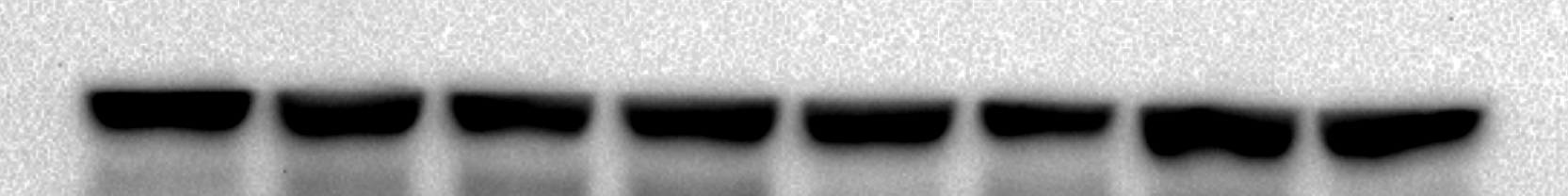

Supplement: Supplementary file 8 — Source data Fig. 6 [file 44319_2025_398_MOESM8_ESM.zip › Figure 6/Figure 6 I/Western blot-Actin.tif]

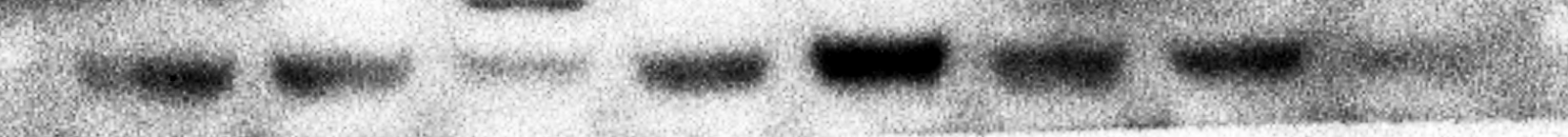

Supplement: Supplementary file 8 — Source data Fig. 6 [file 44319_2025_398_MOESM8_ESM.zip › Figure 6/Figure 6 I/Western blot-Adrb3.tif]

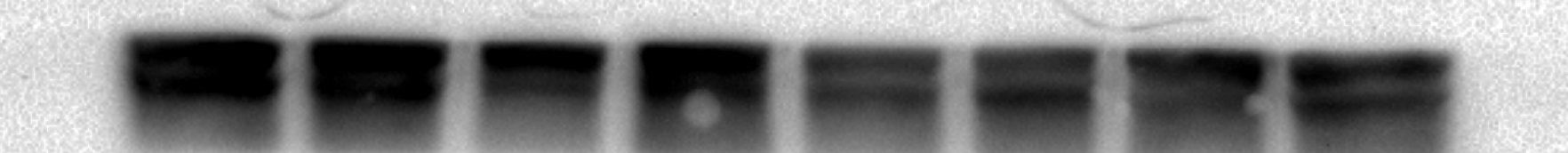

Supplement: Supplementary file 8 — Source data Fig. 6 [file 44319_2025_398_MOESM8_ESM.zip › Figure 6/Figure 6 I/Western blot-HSL.tif]

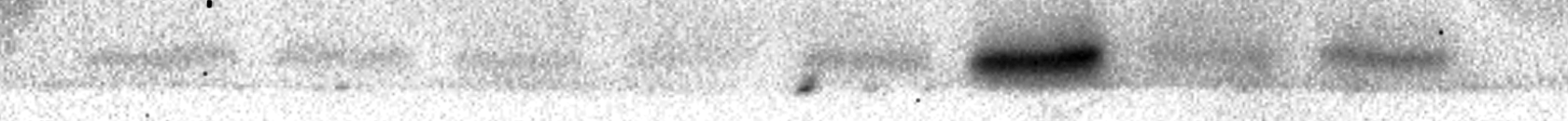

Supplement: Supplementary file 8 — Source data Fig. 6 [file 44319_2025_398_MOESM8_ESM.zip › Figure 6/Figure 6 I/Western blot-p-HSL.tif]

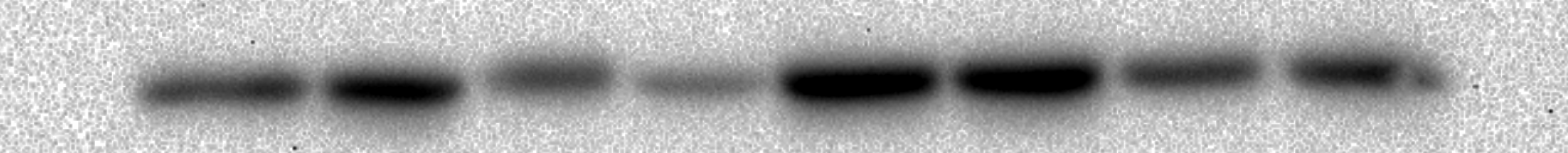

Supplement: Supplementary file 8 — Source data Fig. 6 [file 44319_2025_398_MOESM8_ESM.zip › Figure 6/Figure 6 I/Western blot-Ucp-1.tif]

Figure 6 I

Graph in figures

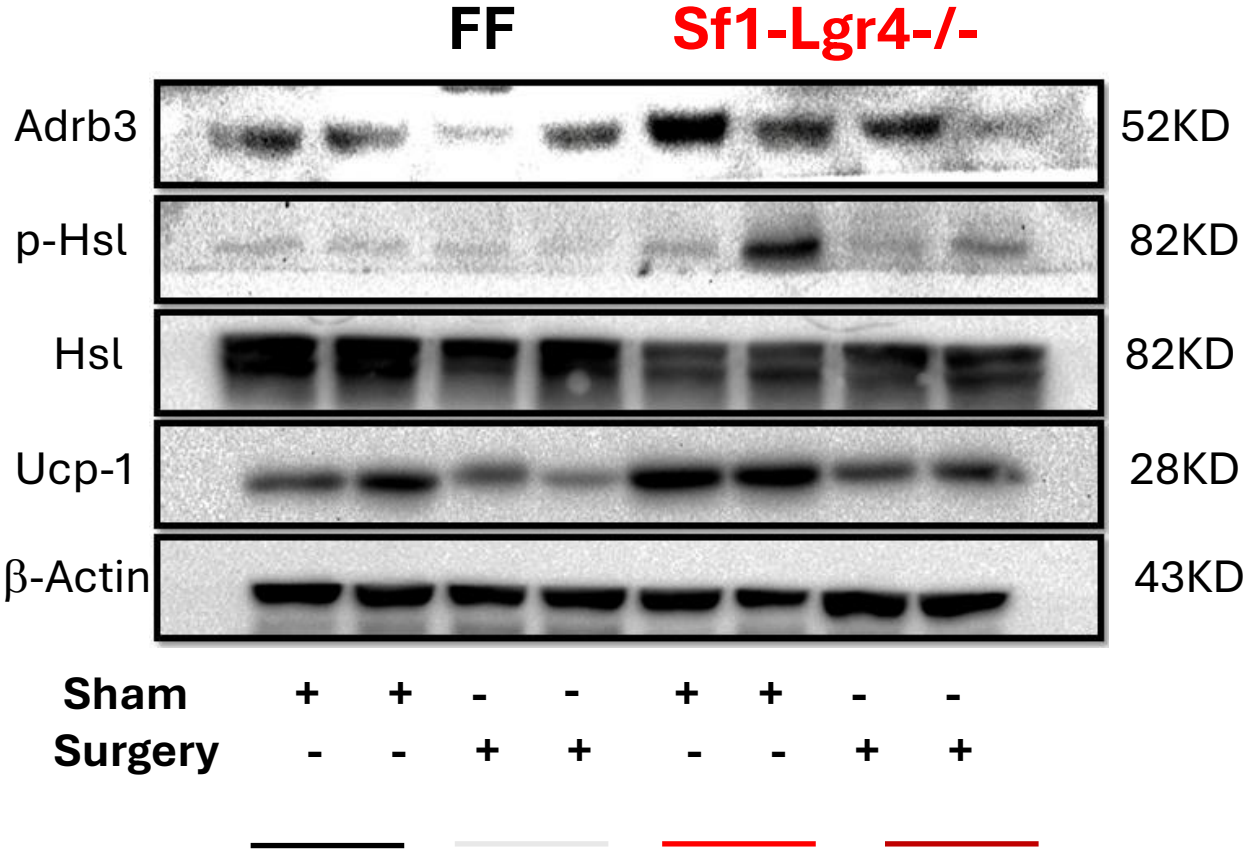

Corresponding uncropped images

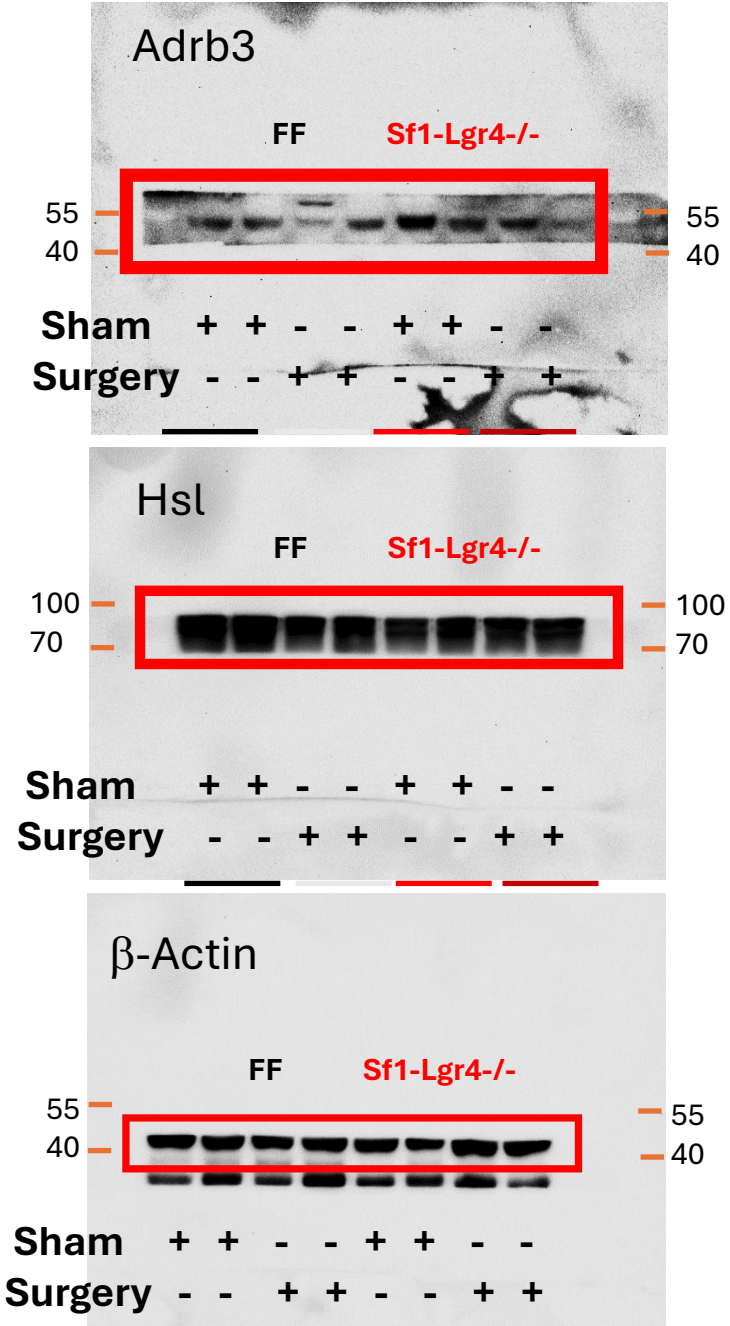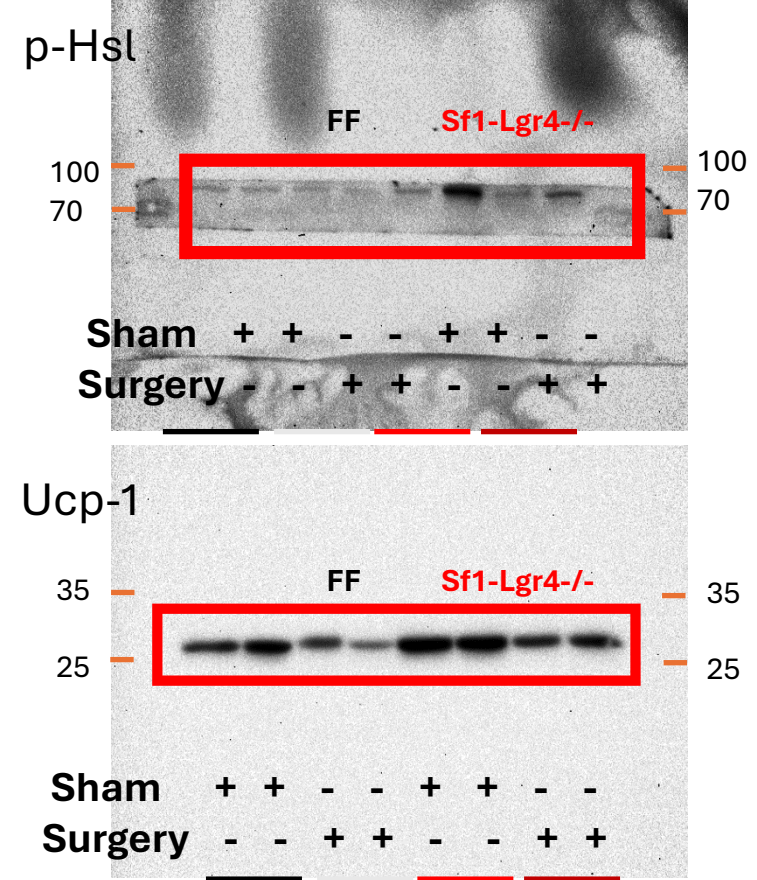

Supplement: Supplementary file 8 — Source data Fig. 6 [file 44319_2025_398_MOESM8_ESM.zip › Figure 6/Uncropped Western Blots of Figure 6.pdf]

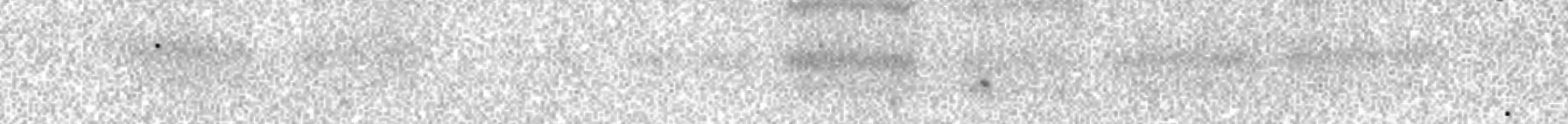

Supplement: Supplementary file 9 — Source data Fig. 7 [file 44319_2025_398_MOESM9_ESM.zip › Figure 7/Figure 7 B/Figure 7 B Left/Western blot-p-Stat3.tif]

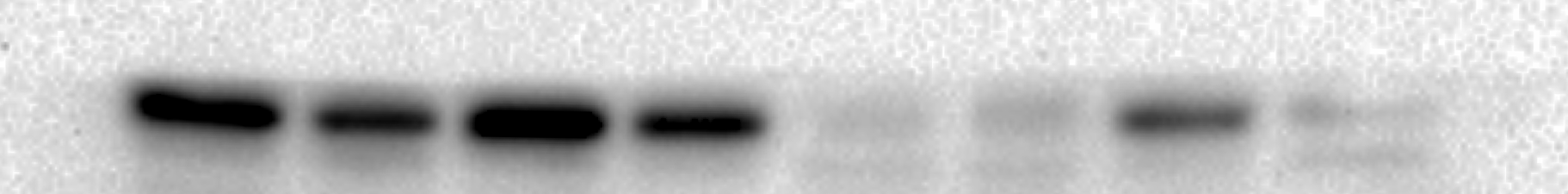

Supplement: Supplementary file 9 — Source data Fig. 7 [file 44319_2025_398_MOESM9_ESM.zip › Figure 7/Figure 7 B/Figure 7 B Left/Western blot-Socs-3.tif]

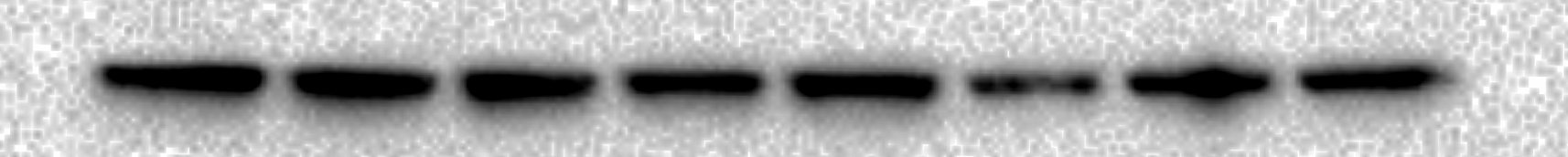

Supplement: Supplementary file 9 — Source data Fig. 7 [file 44319_2025_398_MOESM9_ESM.zip › Figure 7/Figure 7 B/Figure 7 B Left/Western blot-Stat3.tif]

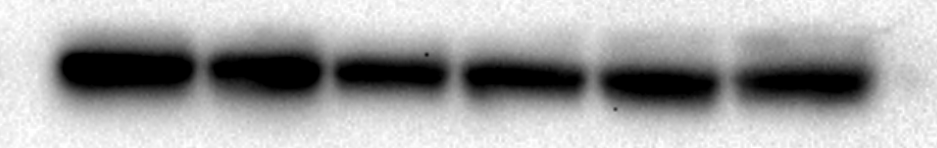

Supplement: Supplementary file 9 — Source data Fig. 7 [file 44319_2025_398_MOESM9_ESM.zip › Figure 7/Figure 7 B/Figure 7 B Middle/Wb-Stat3.tif]

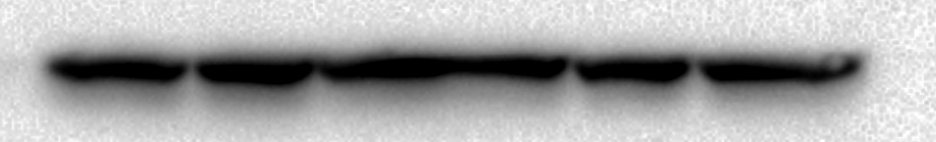

Supplement: Supplementary file 9 — Source data Fig. 7 [file 44319_2025_398_MOESM9_ESM.zip › Figure 7/Figure 7 B/Figure 7 B Middle/Western blot-Actin.tif]

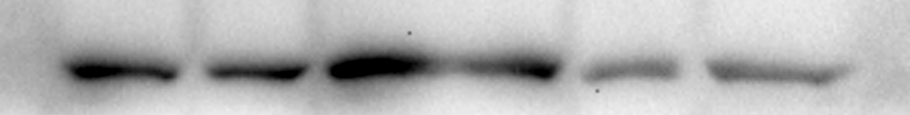

Supplement: Supplementary file 9 — Source data Fig. 7 [file 44319_2025_398_MOESM9_ESM.zip › Figure 7/Figure 7 B/Figure 7 B Middle/Western blot-Lgr4.tif]

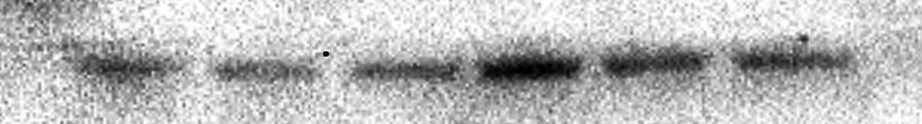

Supplement: Supplementary file 9 — Source data Fig. 7 [file 44319_2025_398_MOESM9_ESM.zip › Figure 7/Figure 7 B/Figure 7 B Middle/Western blot-p-Stat3.tif]

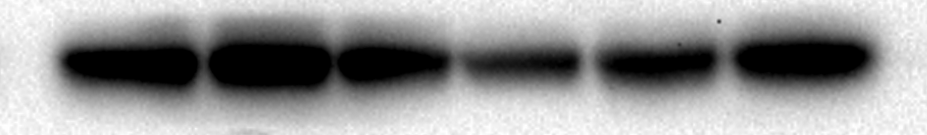

Supplement: Supplementary file 9 — Source data Fig. 7 [file 44319_2025_398_MOESM9_ESM.zip › Figure 7/Figure 7 B/Figure 7 B Middle/Western blot-Socs-3.tif]

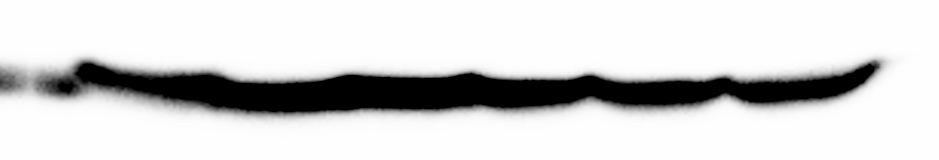

Supplement: Supplementary file 9 — Source data Fig. 7 [file 44319_2025_398_MOESM9_ESM.zip › Figure 7/Figure 7 B/Figure 7 B Right/Western blot-Actin.tif]

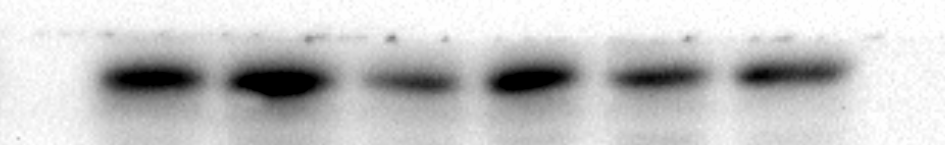

Supplement: Supplementary file 9 — Source data Fig. 7 [file 44319_2025_398_MOESM9_ESM.zip › Figure 7/Figure 7 B/Figure 7 B Right/Western blot-Socs-3.tif]

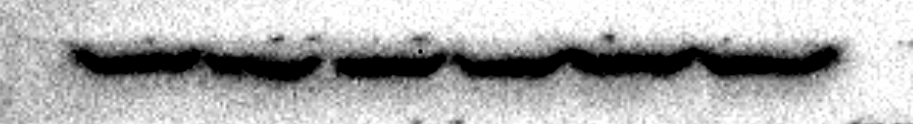

Supplement: Supplementary file 9 — Source data Fig. 7 [file 44319_2025_398_MOESM9_ESM.zip › Figure 7/Figure 7 B/Figure 7 B Right/Western blot-Stat3.tif]

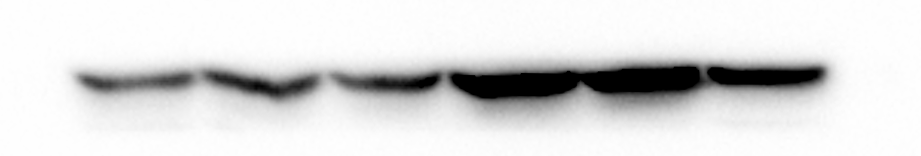

Supplement: Supplementary file 9 — Source data Fig. 7 [file 44319_2025_398_MOESM9_ESM.zip › Figure 7/Figure 7 B/Figure 7 B Right/Western blot=p-Stat3.tif]

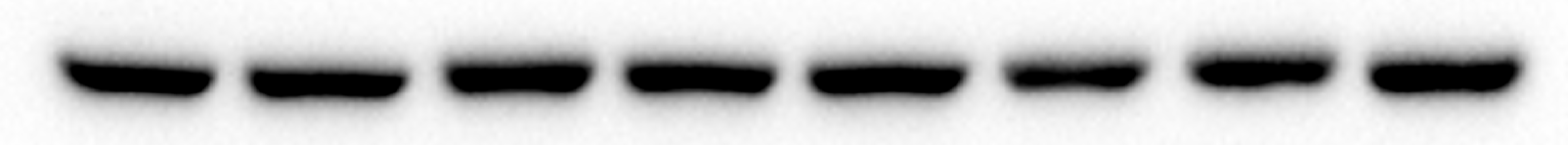

Supplement: Supplementary file 9 — Source data Fig. 7 [file 44319_2025_398_MOESM9_ESM.zip › Figure 7/Figure 7 C/Figure 7 C Left/Western blot-Actin.tif]

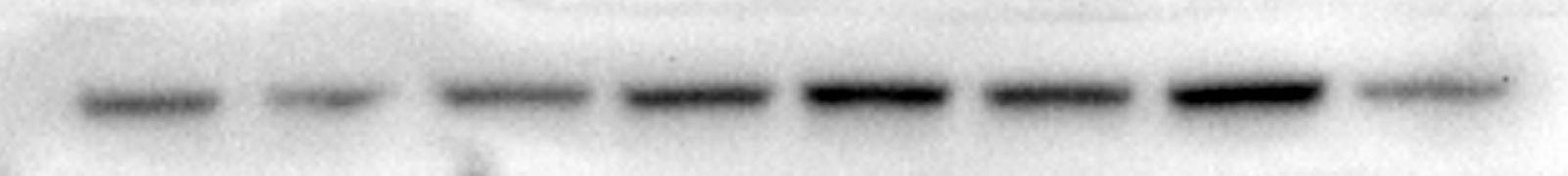

Supplement: Supplementary file 9 — Source data Fig. 7 [file 44319_2025_398_MOESM9_ESM.zip › Figure 7/Figure 7 C/Figure 7 C Left/Western blot-Th.tif]

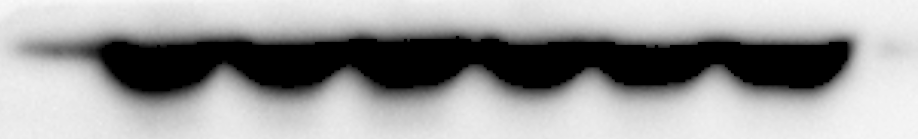

Supplement: Supplementary file 9 — Source data Fig. 7 [file 44319_2025_398_MOESM9_ESM.zip › Figure 7/Figure 7 C/Figure 7 C Middle/Western blot-Actin.tif]

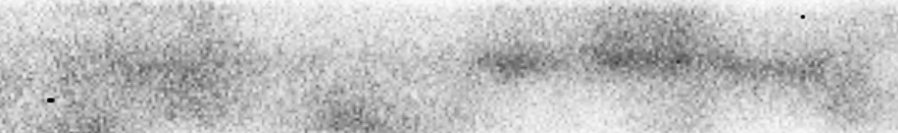

Supplement: Supplementary file 9 — Source data Fig. 7 [file 44319_2025_398_MOESM9_ESM.zip › Figure 7/Figure 7 C/Figure 7 C Middle/Western blot-Th-1.tif]

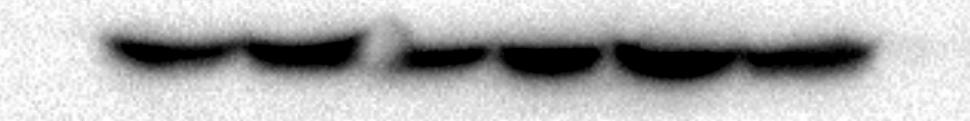

Supplement: Supplementary file 9 — Source data Fig. 7 [file 44319_2025_398_MOESM9_ESM.zip › Figure 7/Figure 7 C/Figure 7 C Right/Western blot-Actin.tif]

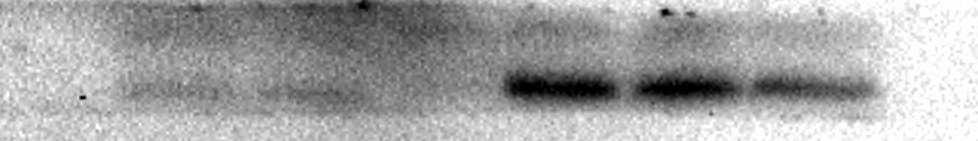

Supplement: Supplementary file 9 — Source data Fig. 7 [file 44319_2025_398_MOESM9_ESM.zip › Figure 7/Figure 7 C/Figure 7 C Right/Western blot-Th-1.tif]

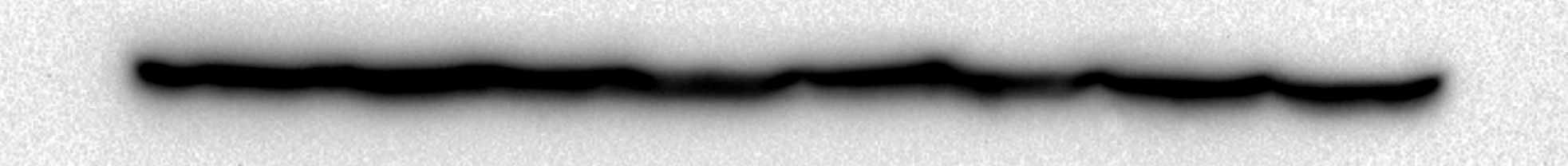

Supplement: Supplementary file 9 — Source data Fig. 7 [file 44319_2025_398_MOESM9_ESM.zip › Figure 7/Figure 7 D/Western blot-Actin.tif]

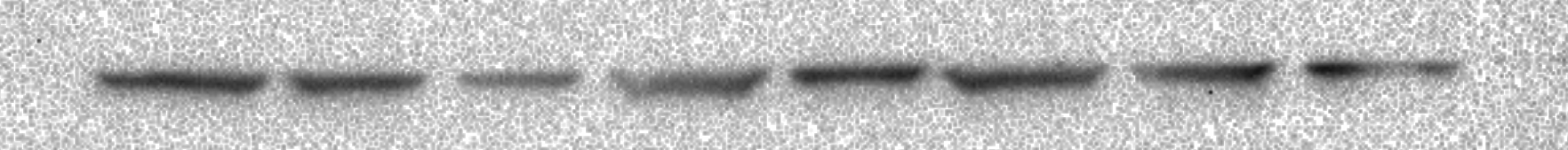

Supplement: Supplementary file 9 — Source data Fig. 7 [file 44319_2025_398_MOESM9_ESM.zip › Figure 7/Figure 7 D/Western blot-p-Stat3.tif]

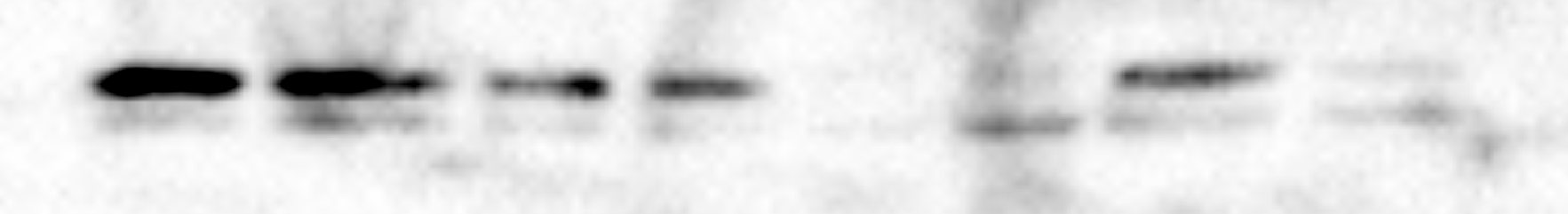

Supplement: Supplementary file 9 — Source data Fig. 7 [file 44319_2025_398_MOESM9_ESM.zip › Figure 7/Figure 7 D/Western blot-Socs-3.tif]

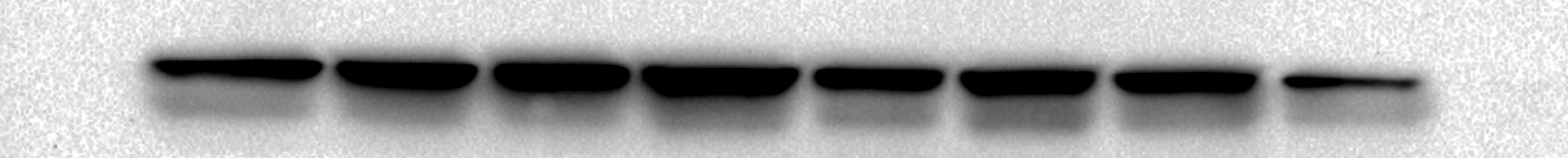

Supplement: Supplementary file 9 — Source data Fig. 7 [file 44319_2025_398_MOESM9_ESM.zip › Figure 7/Figure 7 D/Western blot-Stat3.tif]

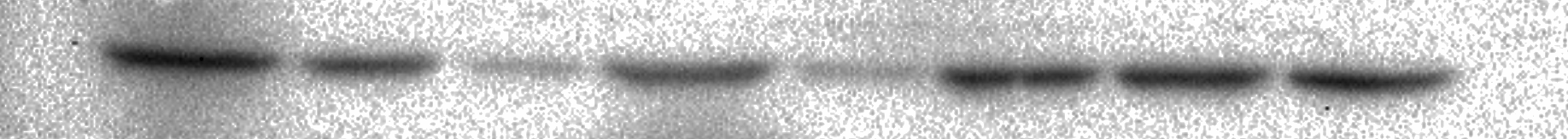

Supplement: Supplementary file 9 — Source data Fig. 7 [file 44319_2025_398_MOESM9_ESM.zip › Figure 7/Figure 7 D/Western blot-Th-1.tif]

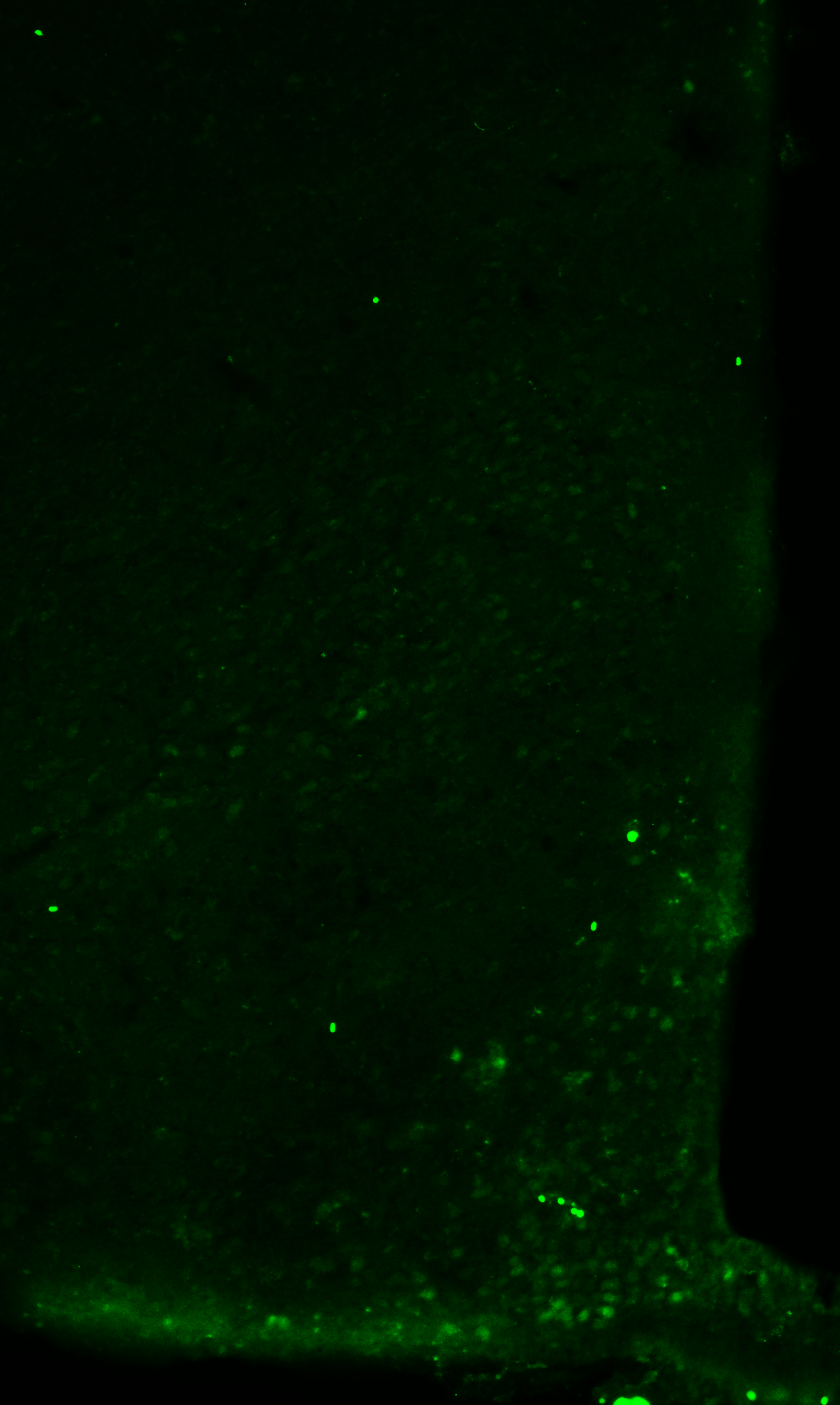

Supplement: Supplementary file 9 — Source data Fig. 7 [file 44319_2025_398_MOESM9_ESM.zip › Figure 7/Figure 7 E/FF-ARC VMH.tif]

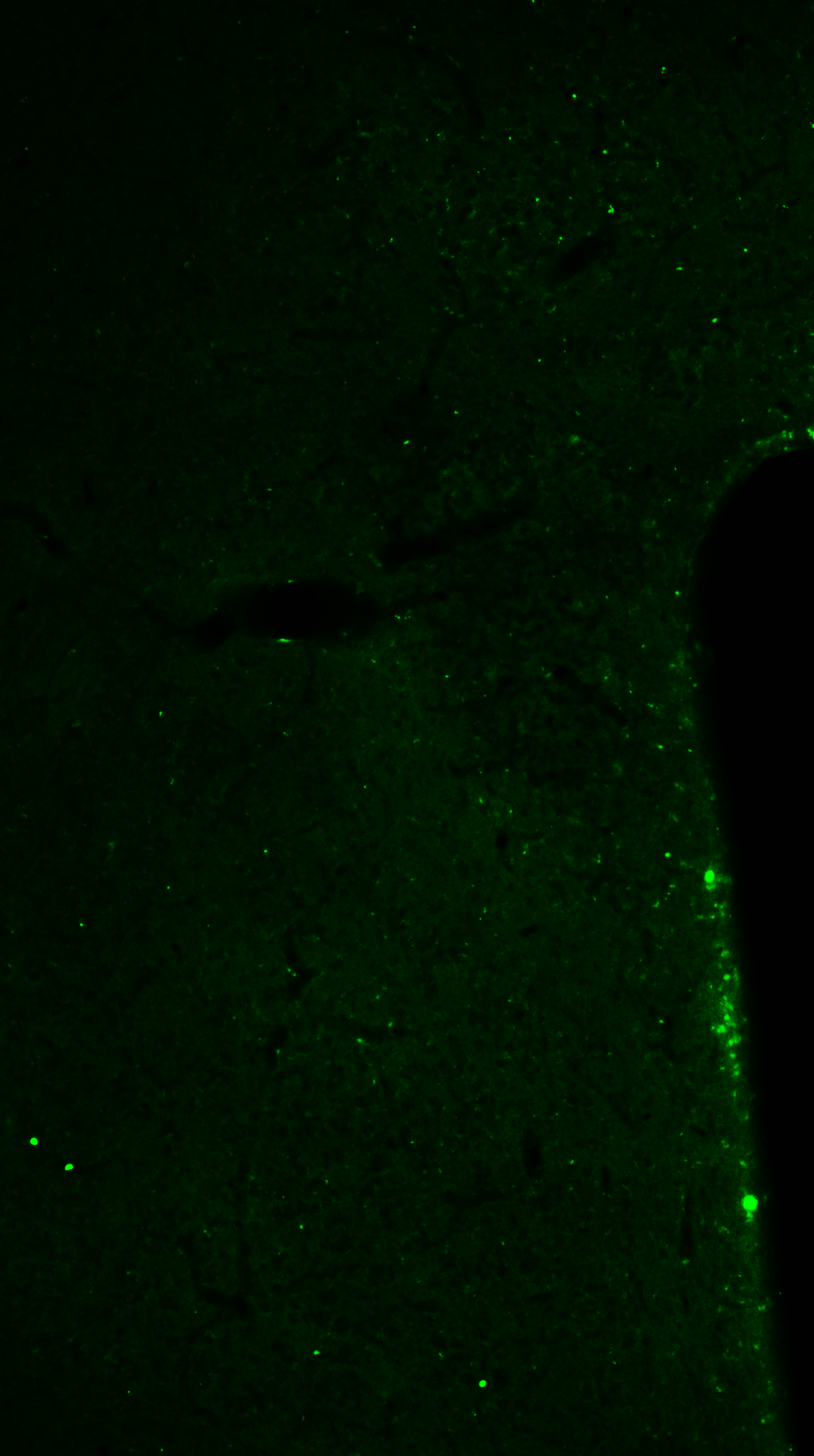

Supplement: Supplementary file 9 — Source data Fig. 7 [file 44319_2025_398_MOESM9_ESM.zip › Figure 7/Figure 7 E/FF-PVN.tif]

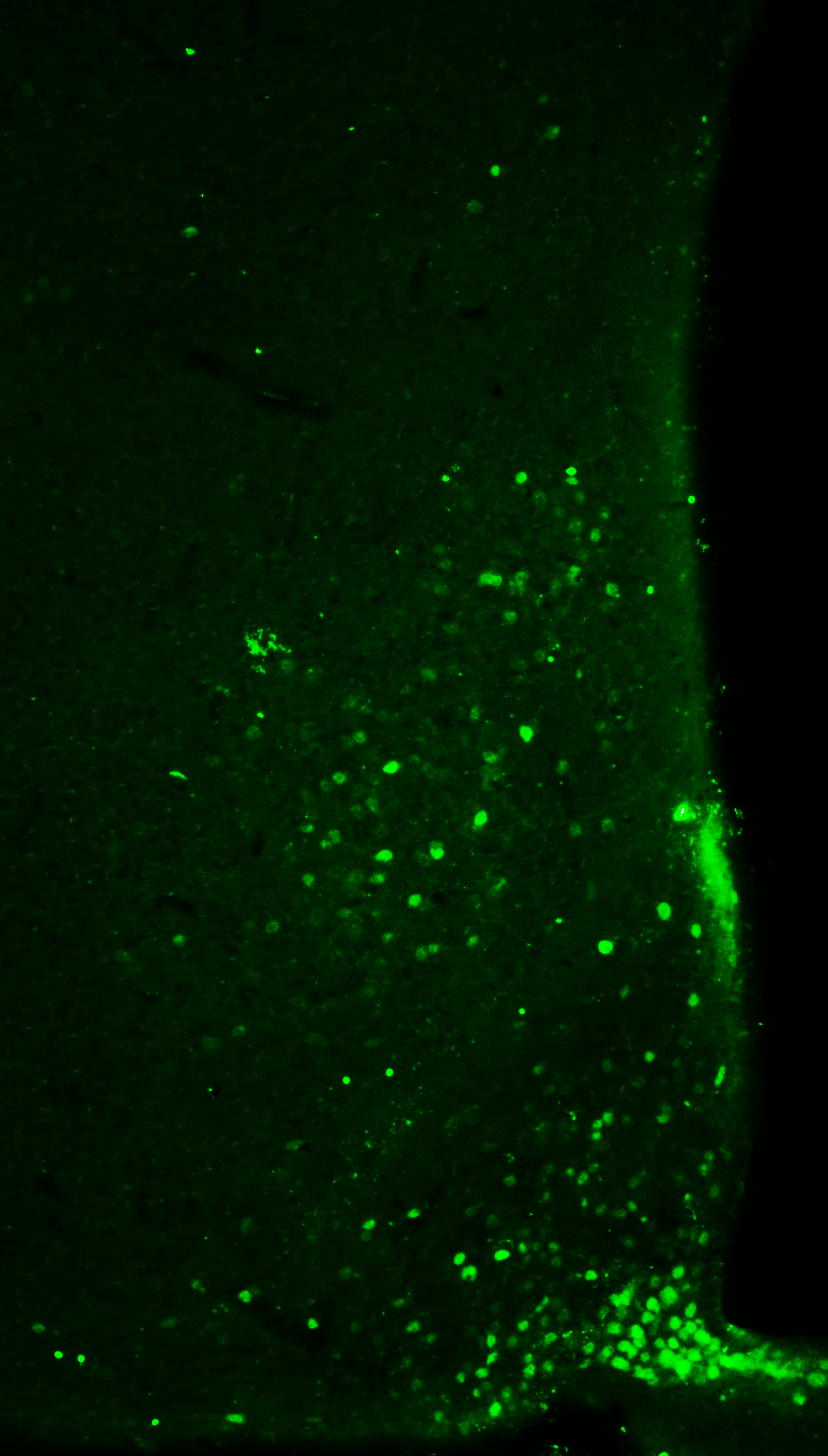

Supplement: Supplementary file 9 — Source data Fig. 7 [file 44319_2025_398_MOESM9_ESM.zip › Figure 7/Figure 7 E/KO-ARC VMH.tif]

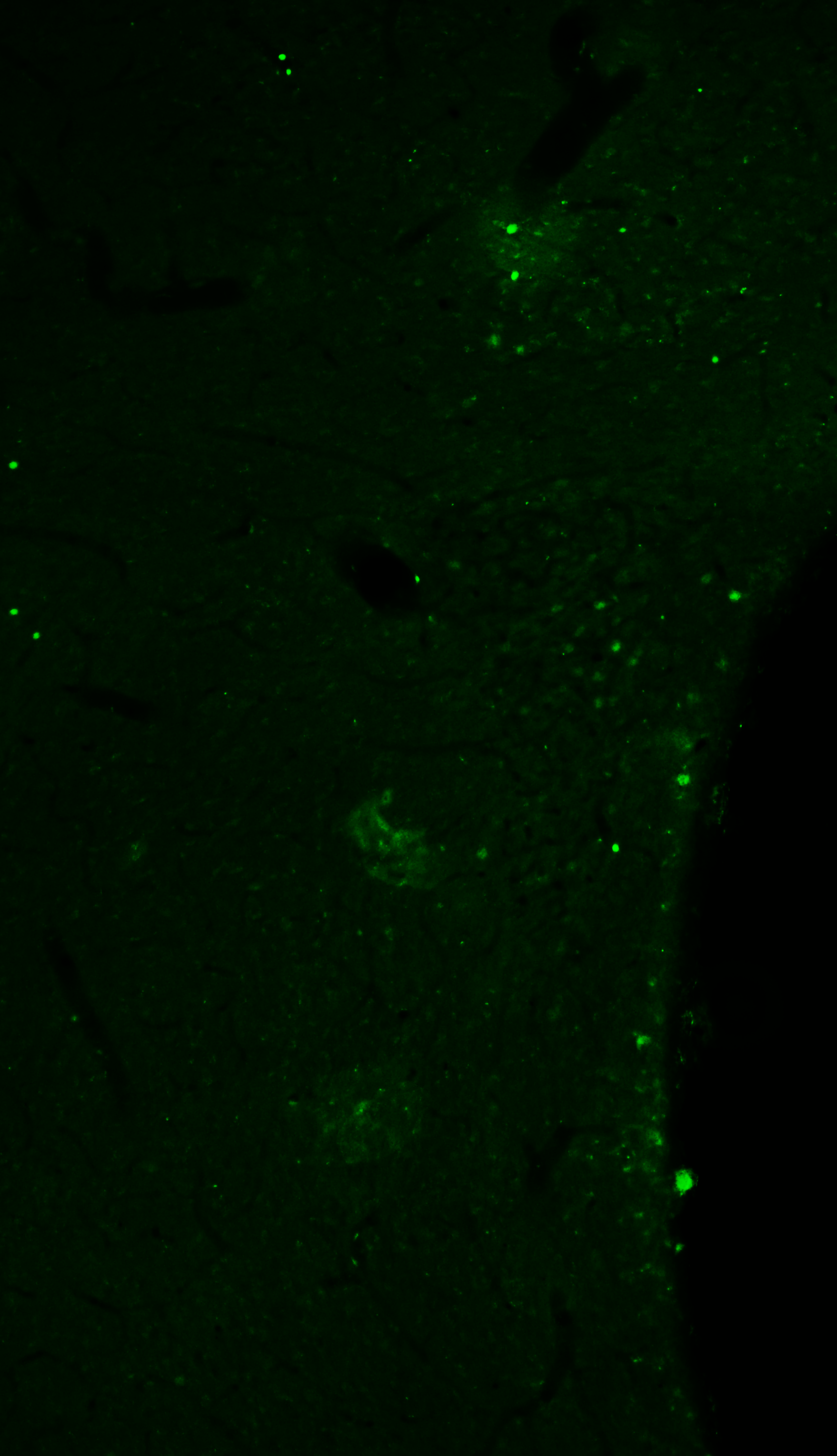

Supplement: Supplementary file 9 — Source data Fig. 7 [file 44319_2025_398_MOESM9_ESM.zip › Figure 7/Figure 7 E/KO-PVN.tif]

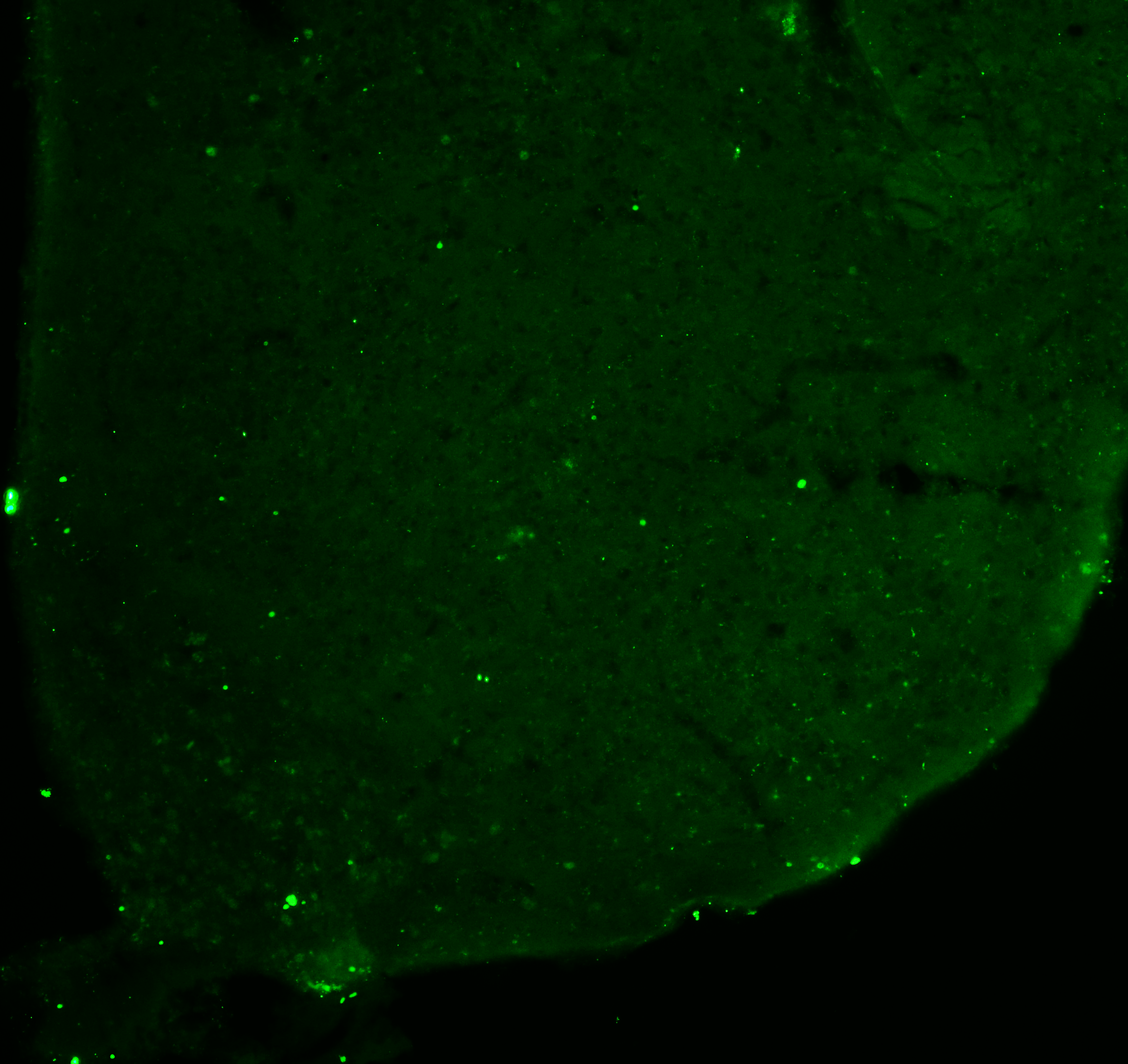

Supplement: Supplementary file 9 — Source data Fig. 7 [file 44319_2025_398_MOESM9_ESM.zip › Figure 7/Figure 7 F/FF-ARC VMH.tif]

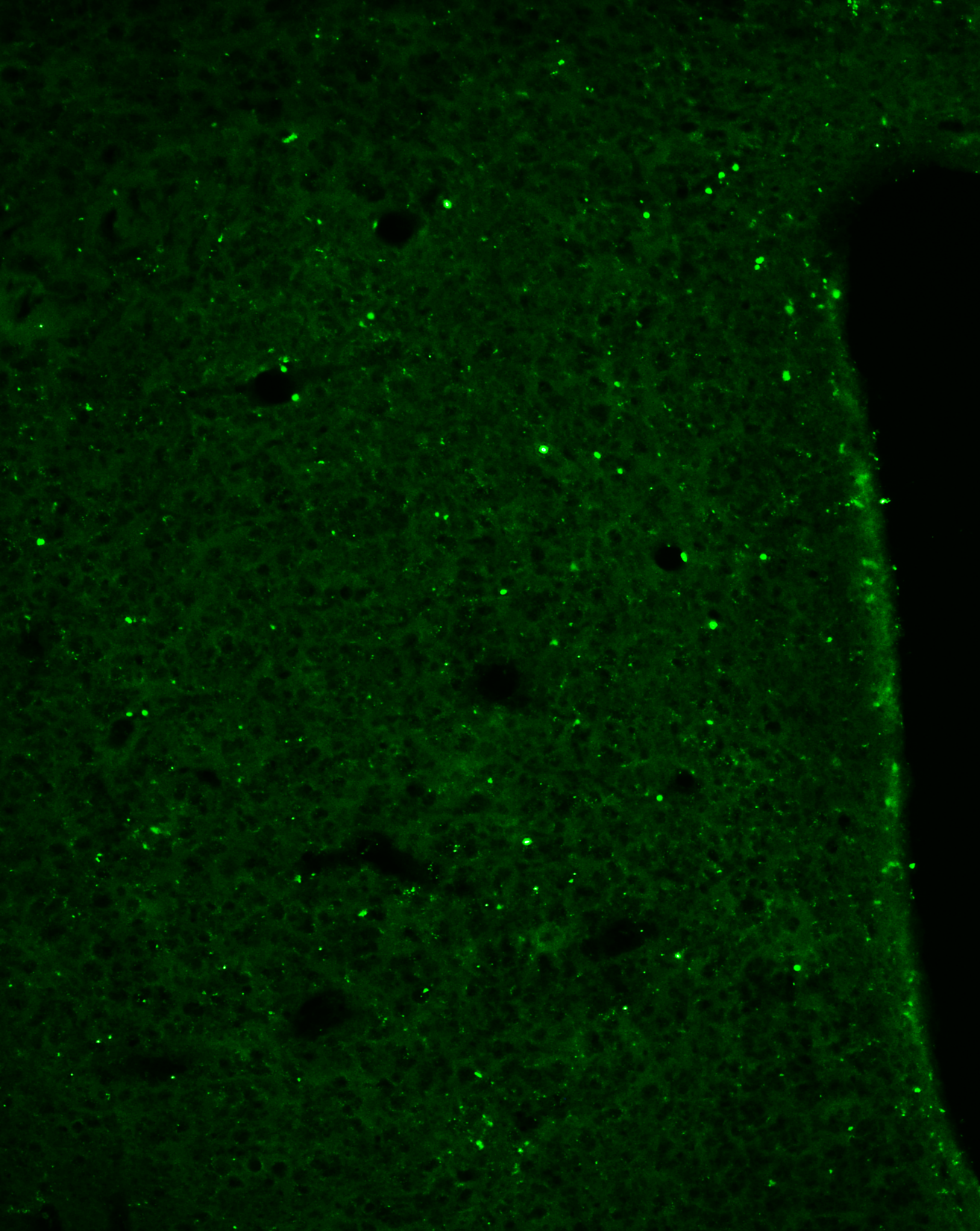

Supplement: Supplementary file 9 — Source data Fig. 7 [file 44319_2025_398_MOESM9_ESM.zip › Figure 7/Figure 7 F/FF-PVN.tif]

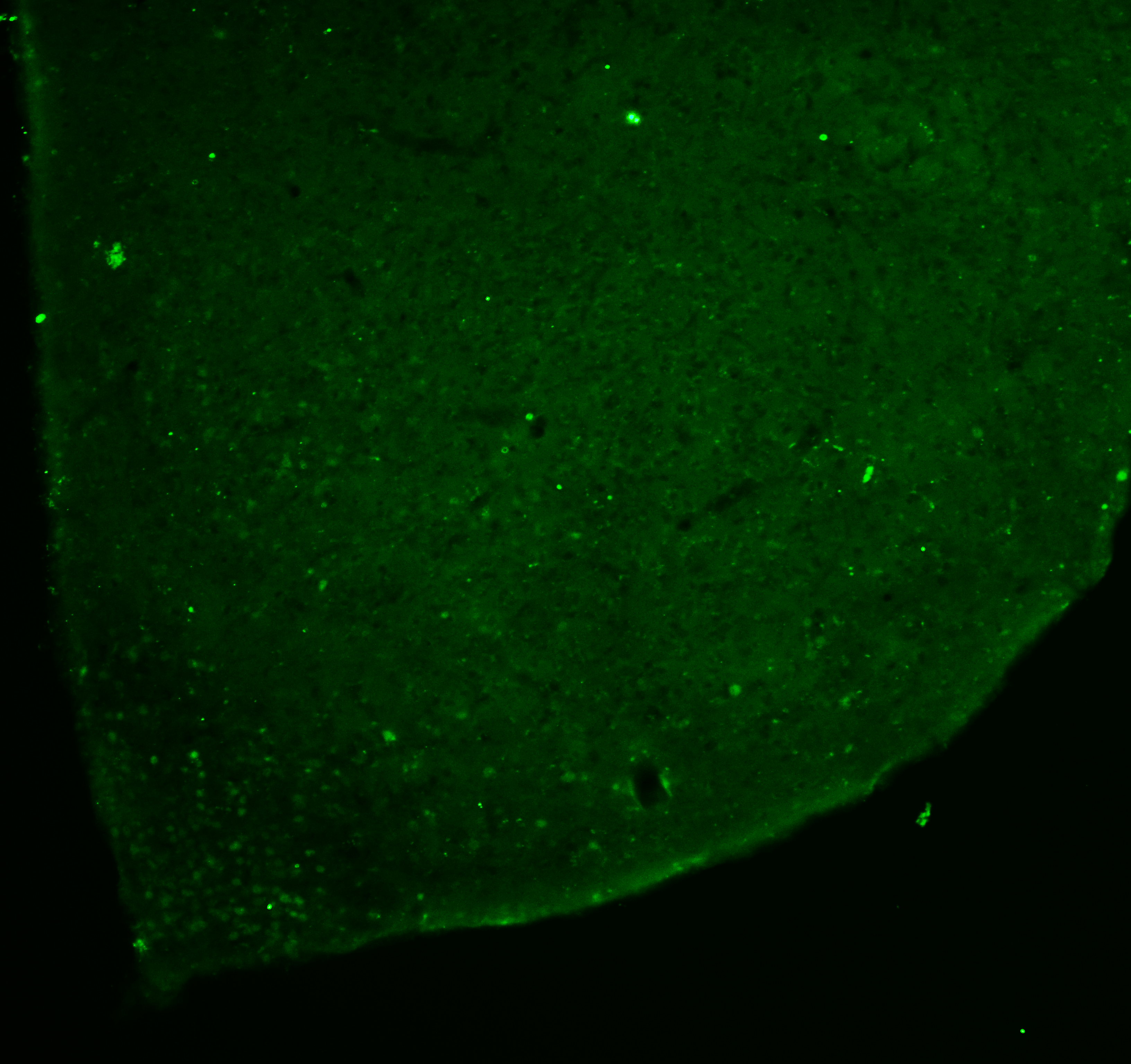

Supplement: Supplementary file 9 — Source data Fig. 7 [file 44319_2025_398_MOESM9_ESM.zip › Figure 7/Figure 7 F/KO-ARC VMH.tif]

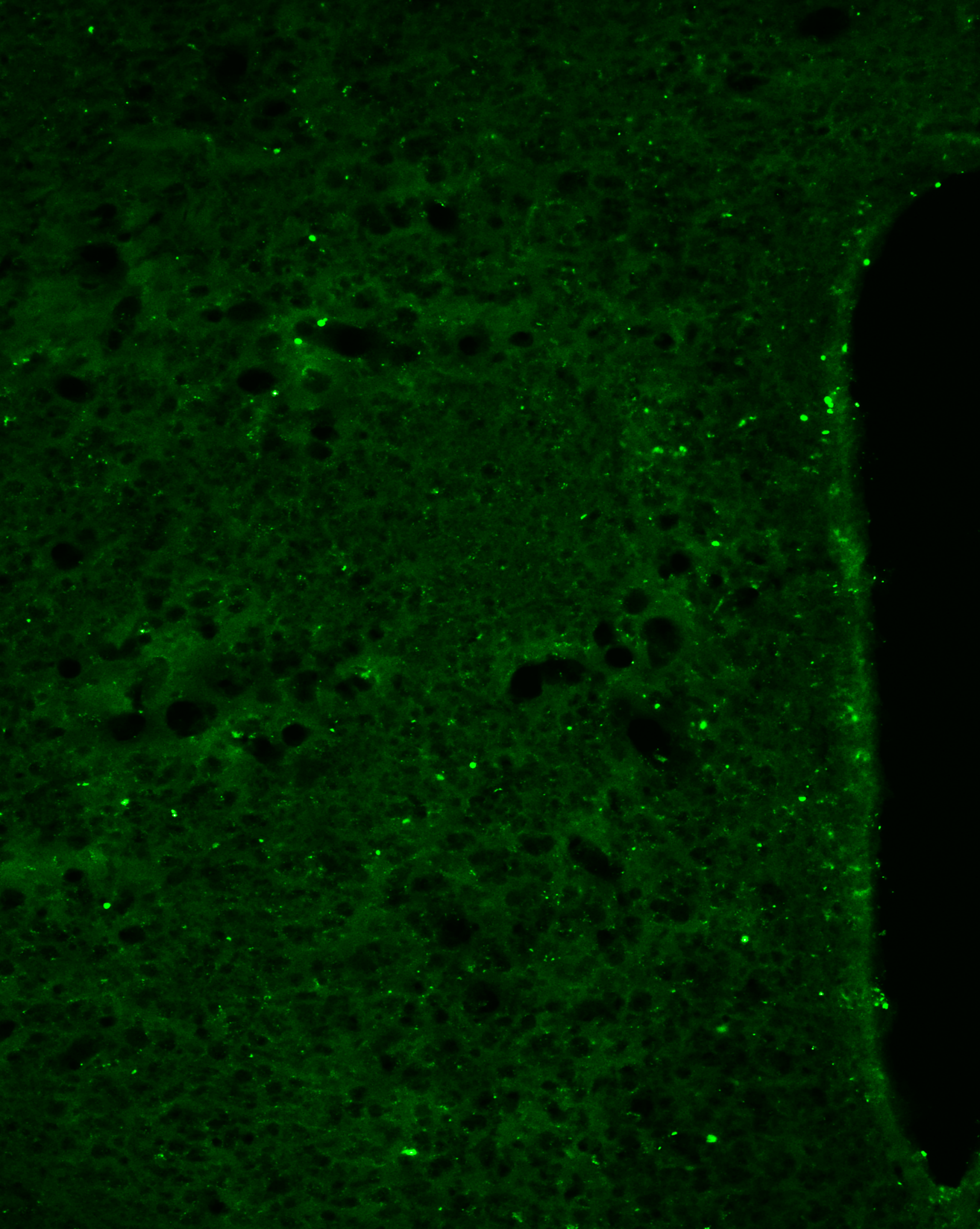

Supplement: Supplementary file 9 — Source data Fig. 7 [file 44319_2025_398_MOESM9_ESM.zip › Figure 7/Figure 7 F/KO-PVN.tif]

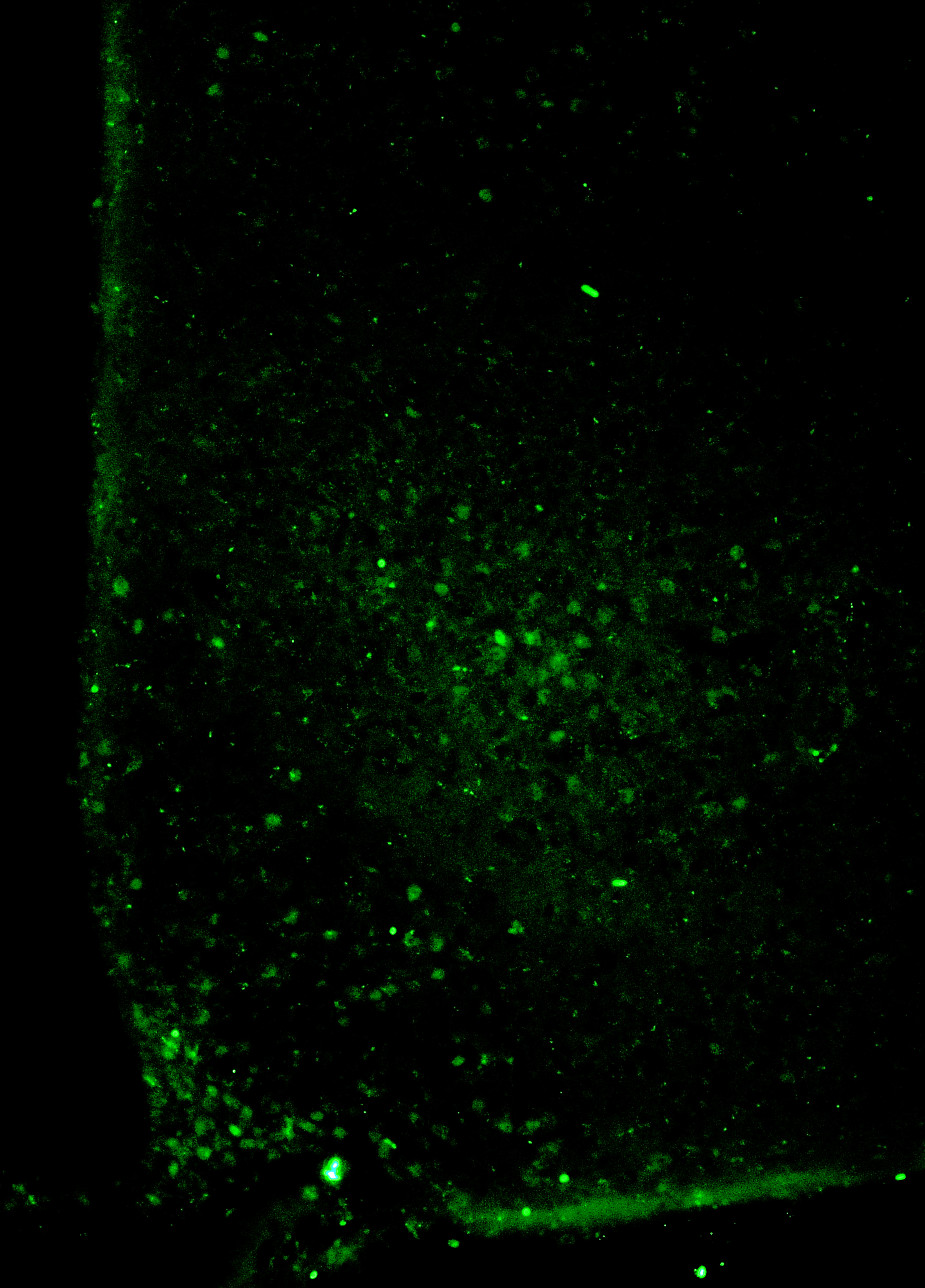

Supplement: Supplementary file 9 — Source data Fig. 7 [file 44319_2025_398_MOESM9_ESM.zip › Figure 7/Figure 7 G/FF-ARC VMH.tif]
