# Supplementary material for: Deficiency of neuronal LGR4 increases energy expenditure and inhibits food intake via hypothalamic leptin signaling
Source: EMBO Rep. 2025 Mar 11;26(8):2098–120. doi: 10.1038/s44319-025-00398-5 (PMC12018946; doi:10.1038/s44319-025-00398-5)

Figure 3 A

Graph in figures

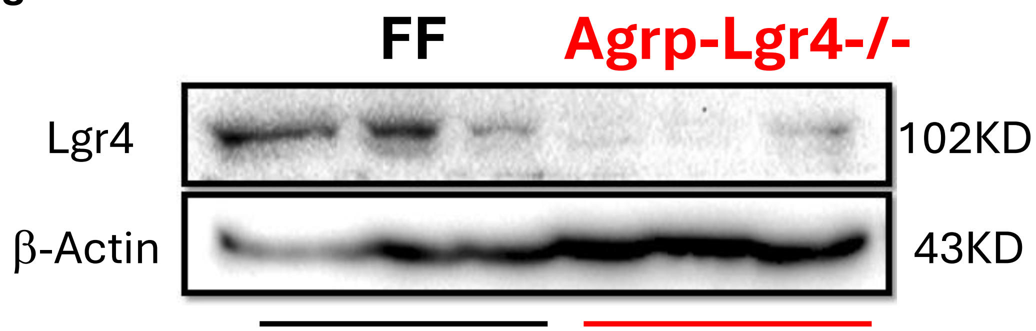

Corresponding uncropped images

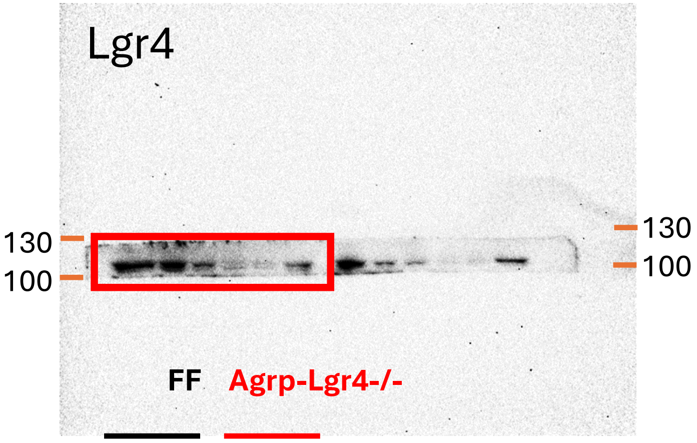

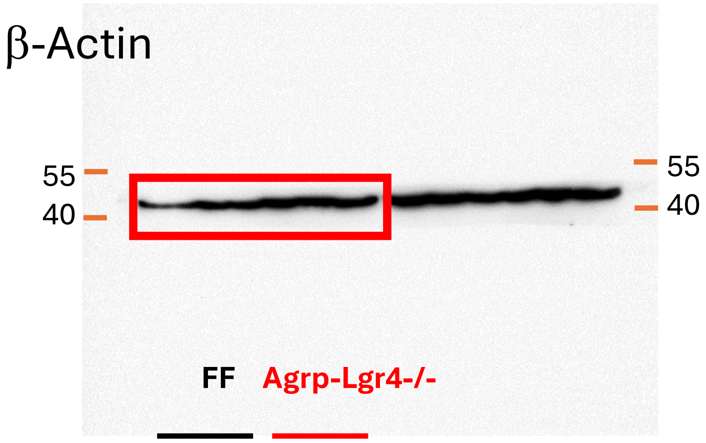

Figure 3 P

Graph in figures

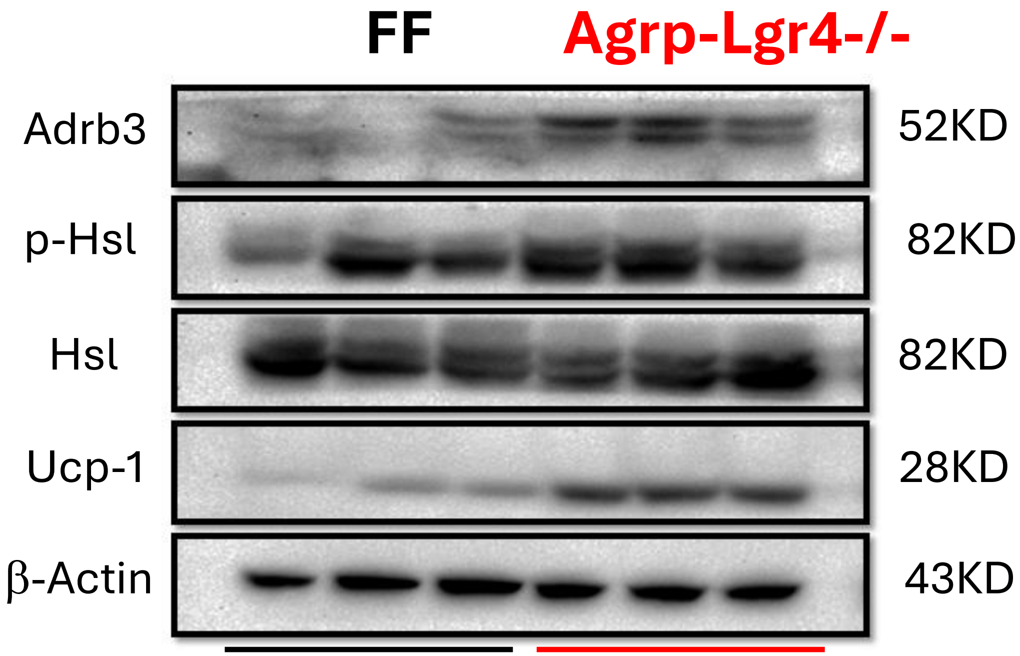

Corresponding uncropped images

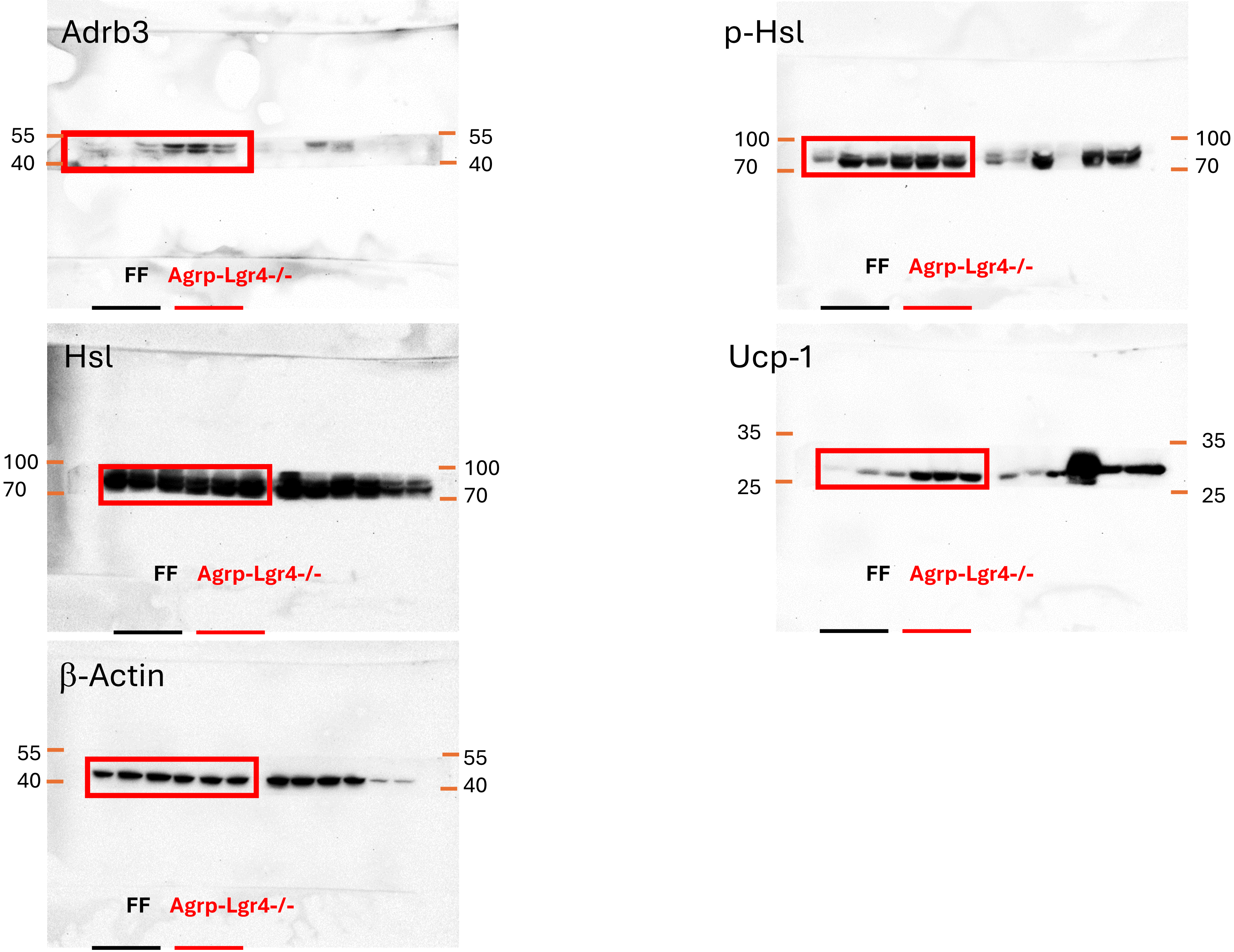

Supplement: Supplementary file 5 — Source data Fig. 3 [file 44319_2025_398_MOESM5_ESM.zip › Figure 3/Uncropped Western Blots of Figure 3.pdf]
