# Supplementary material for: Deficiency of neuronal LGR4 increases energy expenditure and inhibits food intake via hypothalamic leptin signaling
Source: EMBO Rep. 2025 Mar 11;26(8):2098–120. doi: 10.1038/s44319-025-00398-5 (PMC12018946; doi:10.1038/s44319-025-00398-5)

Figure 7 B Left

Graph in figures

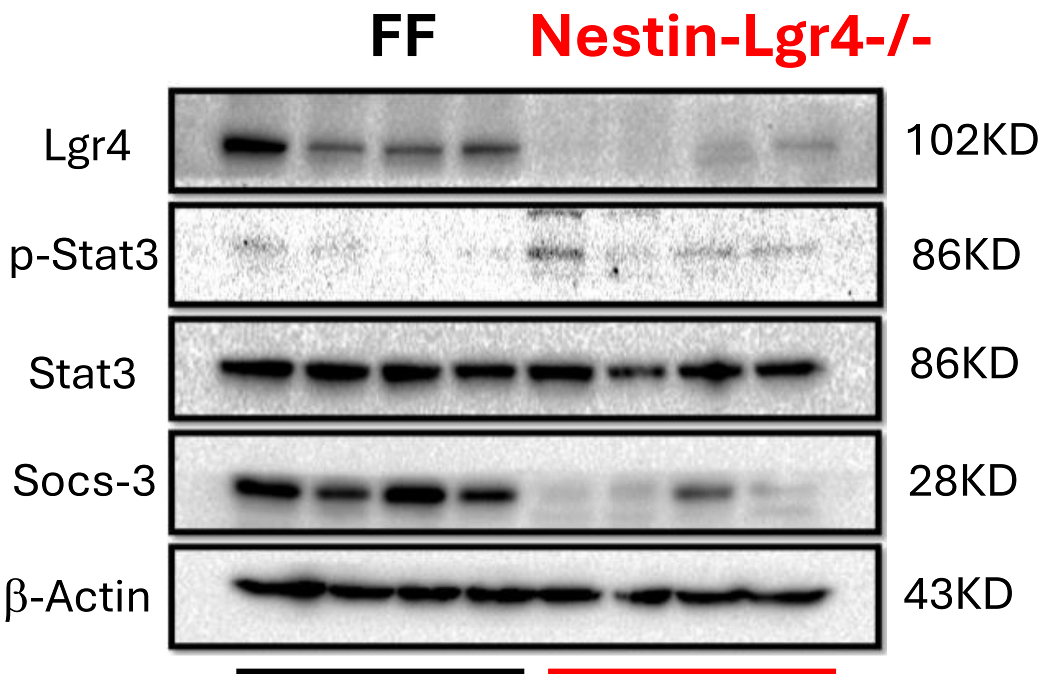

Corresponding uncropped images

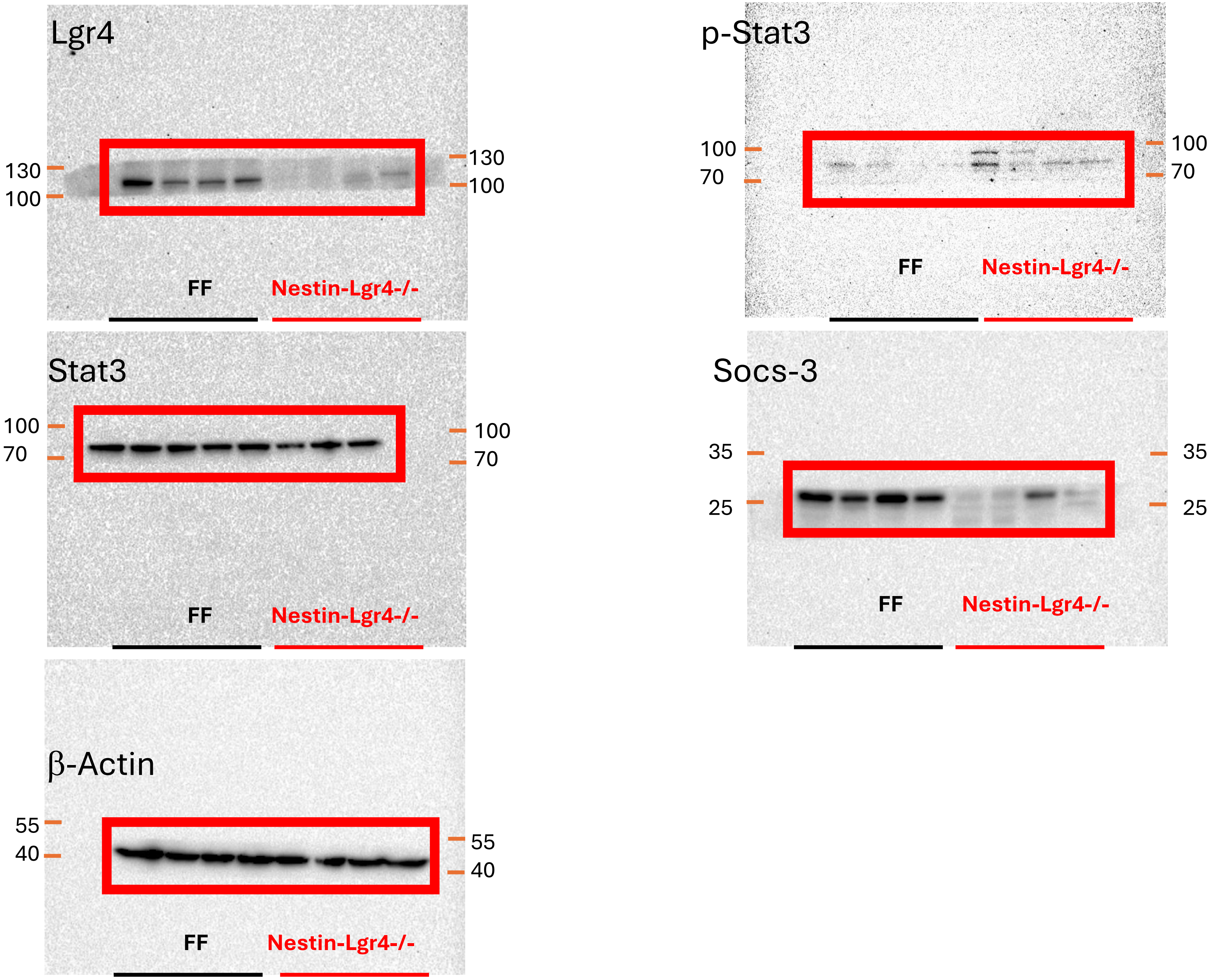

Figure 7 B Middle

Graph in figures

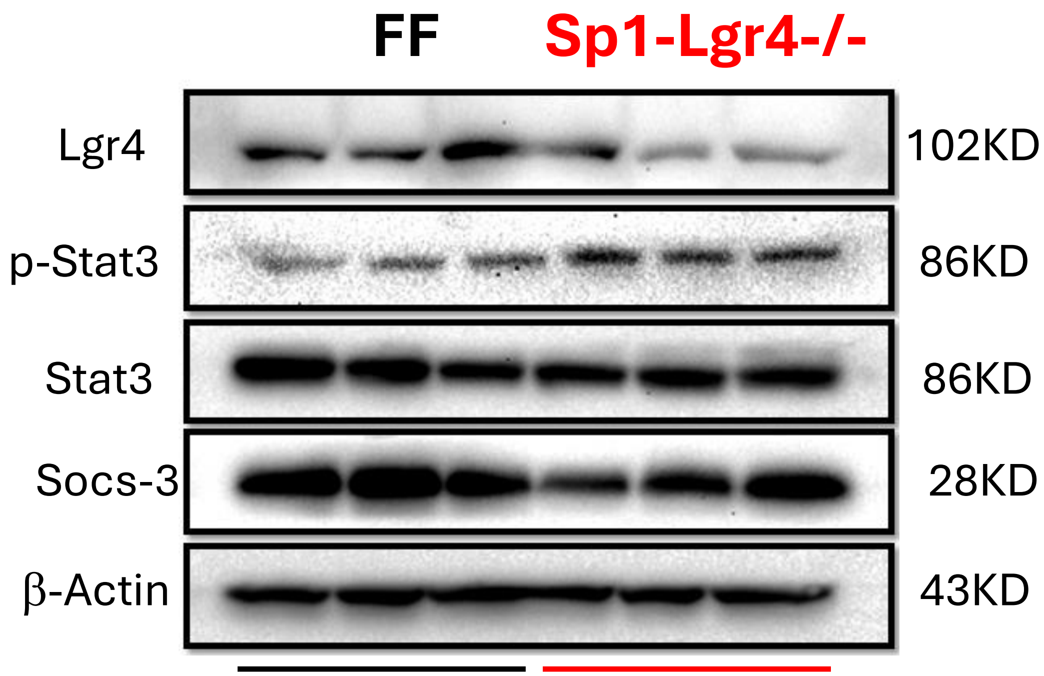

Corresponding uncropped images

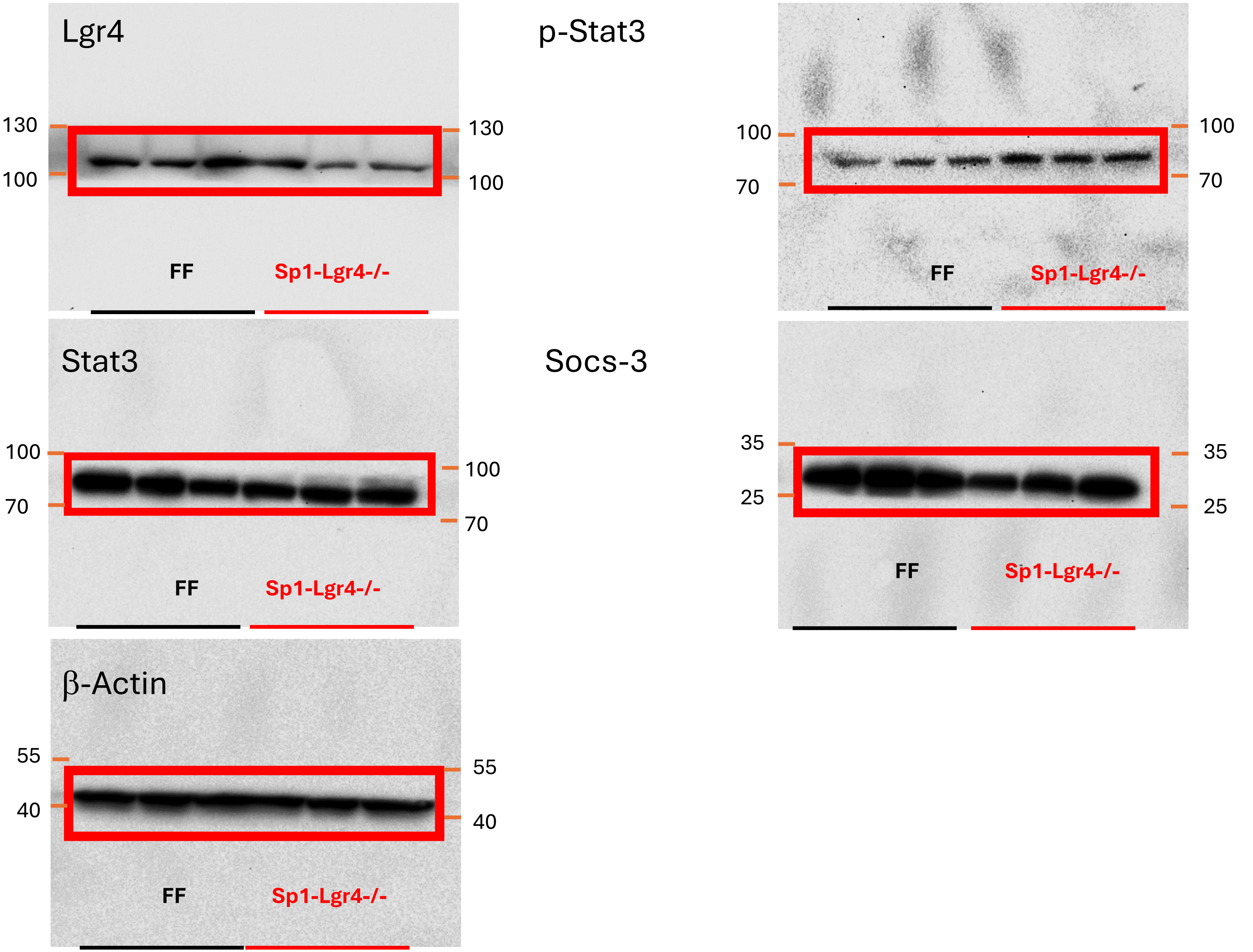

Figure 7 B Right

Graph in figures

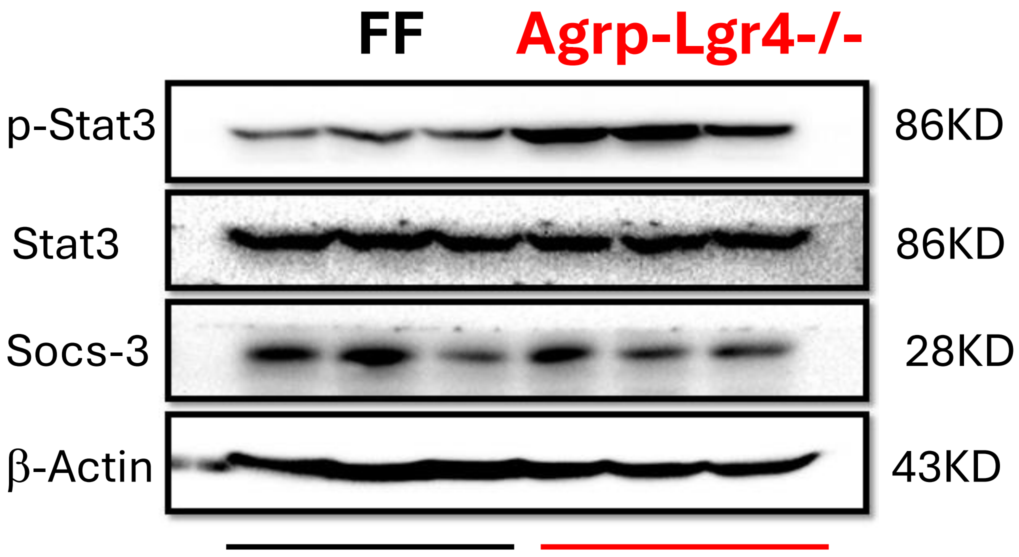

# Corresponding uncropped images

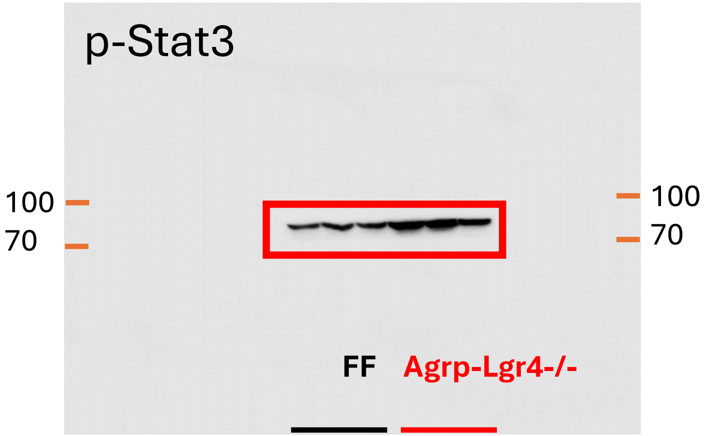

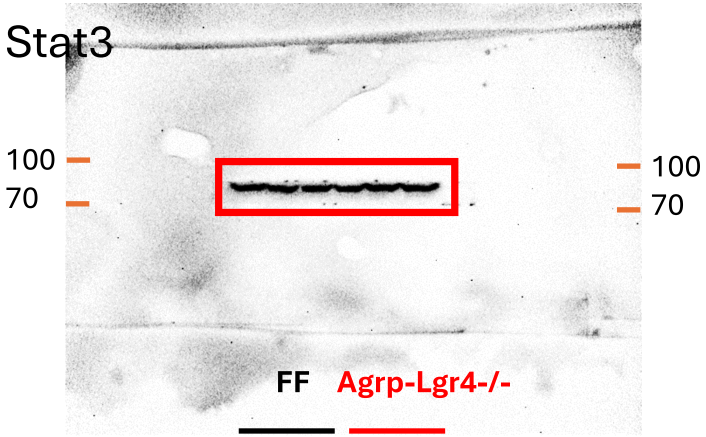

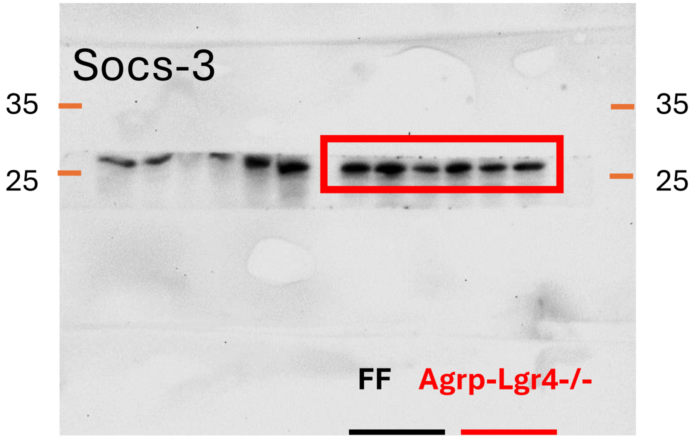

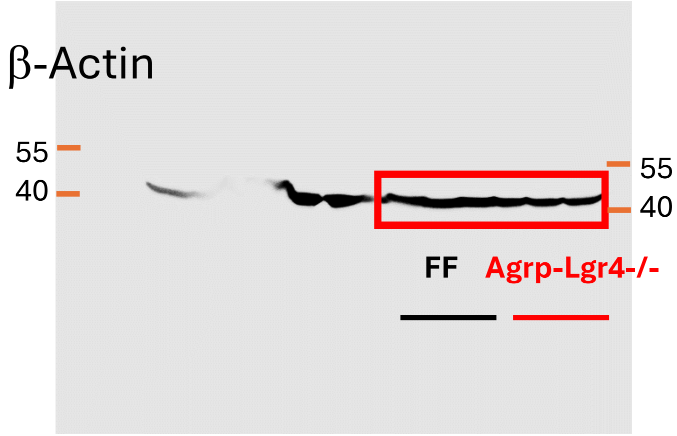

Figure 7 C Left

Graph in figures

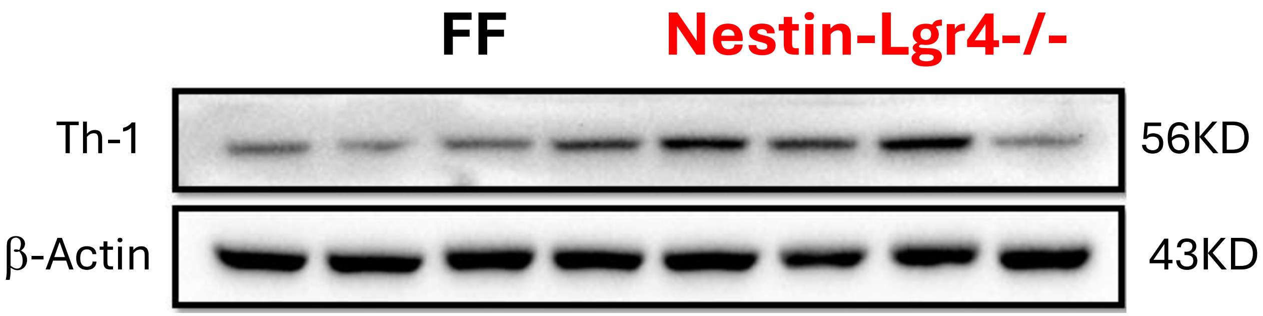

Corresponding uncropped images

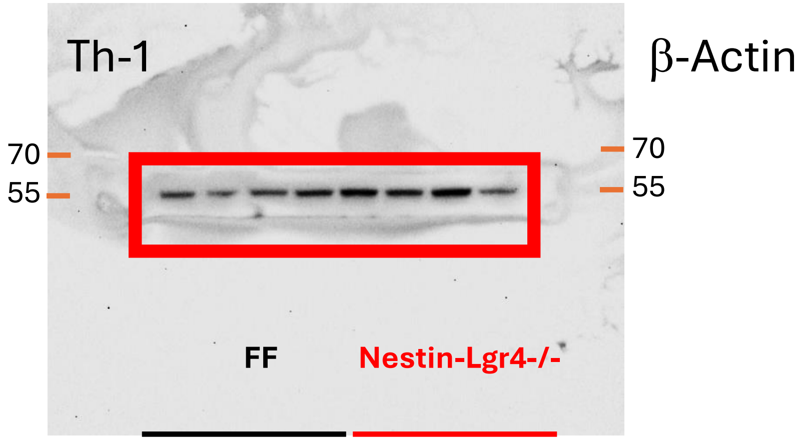

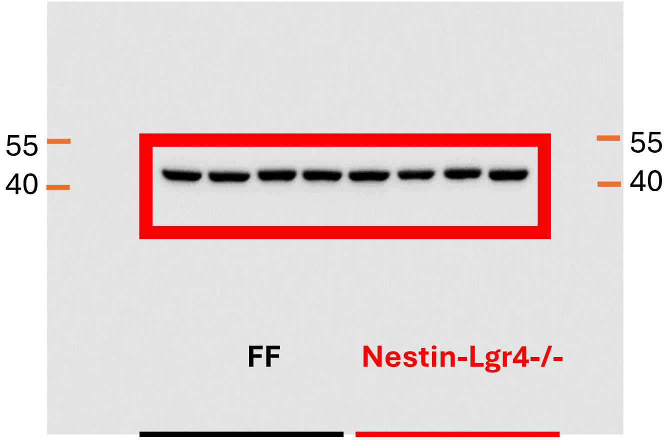

Figure 7 C Middle

Graph in figures

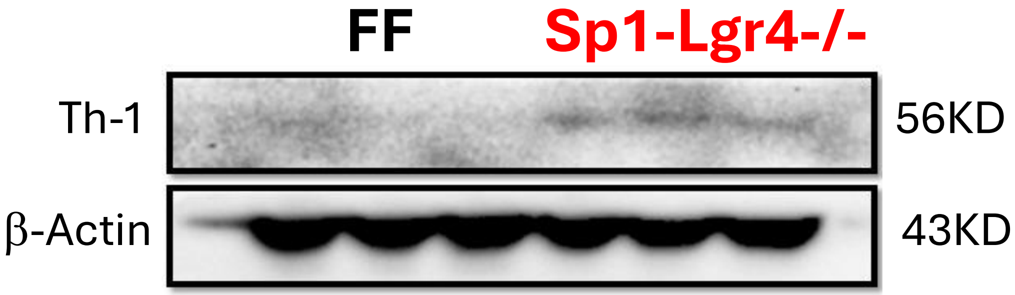

Corresponding uncropped images

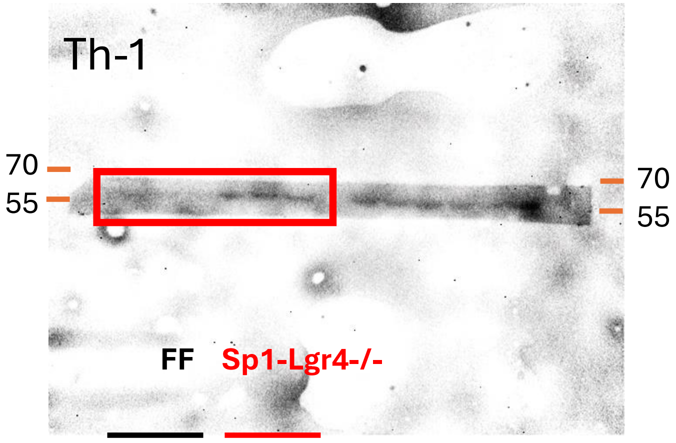

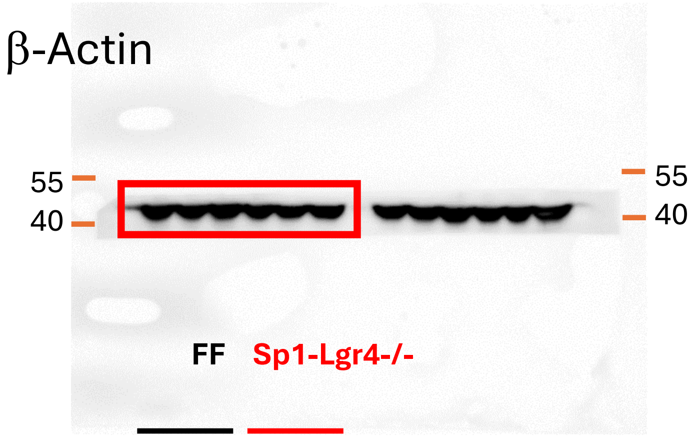

Figure 7 C Right

Graph in figures

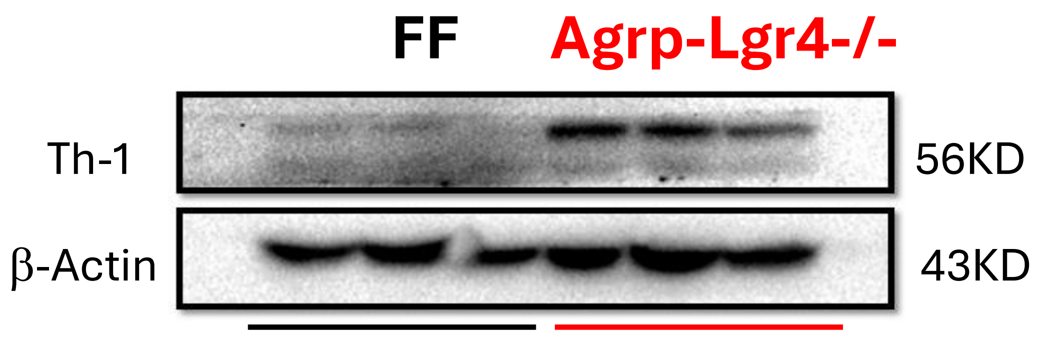

Corresponding uncropped images

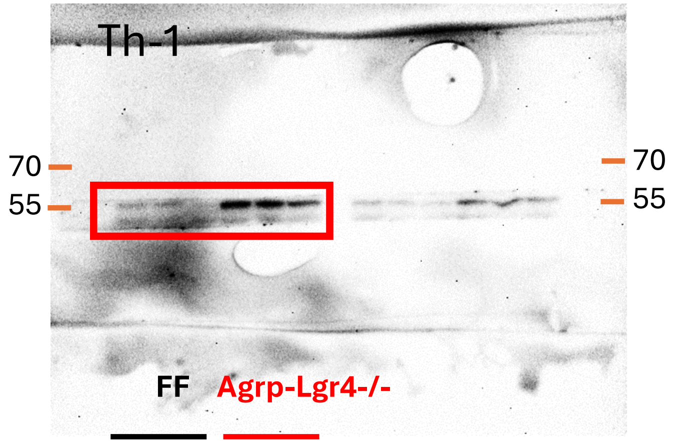

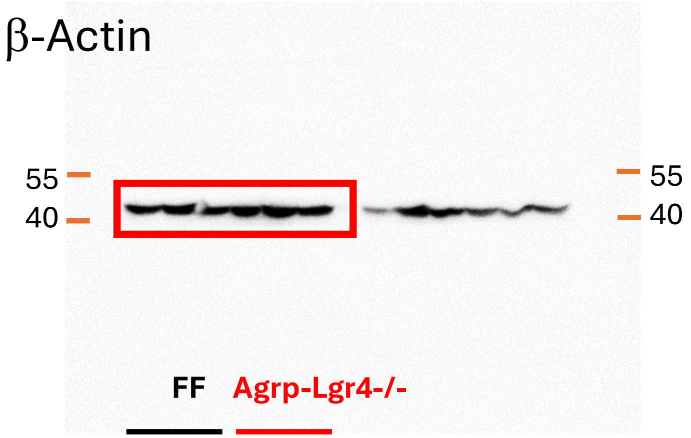

Figure 7 D

Graph in figures

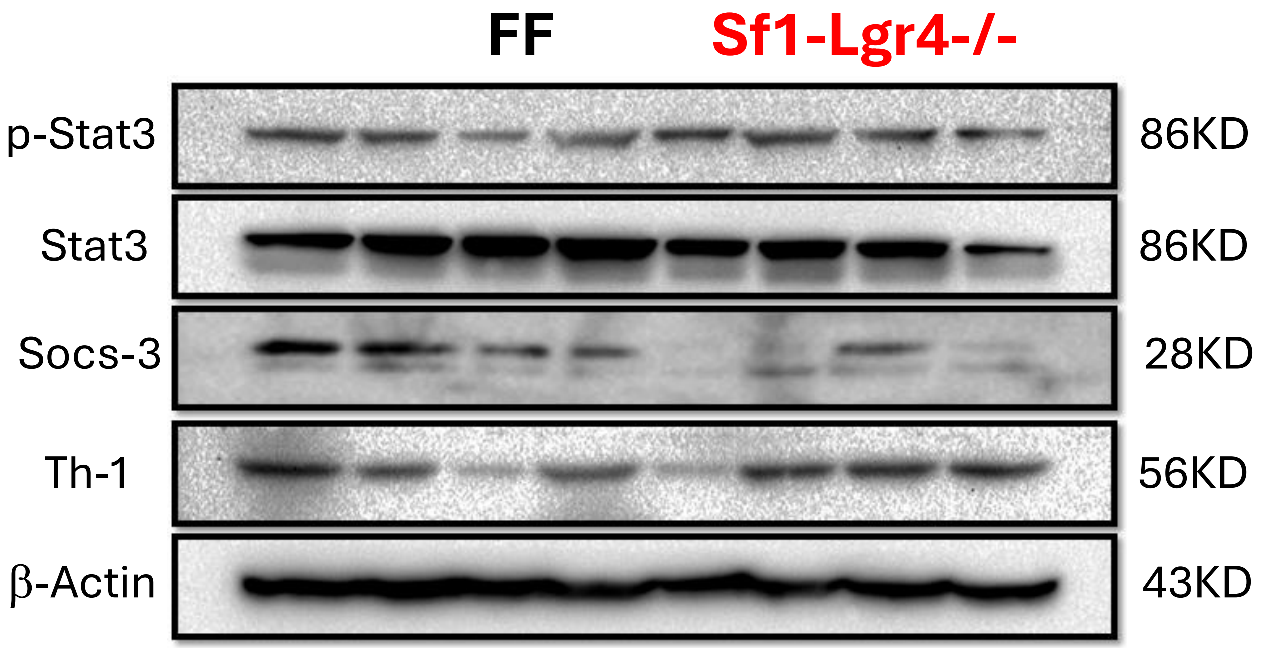

Corresponding uncropped images

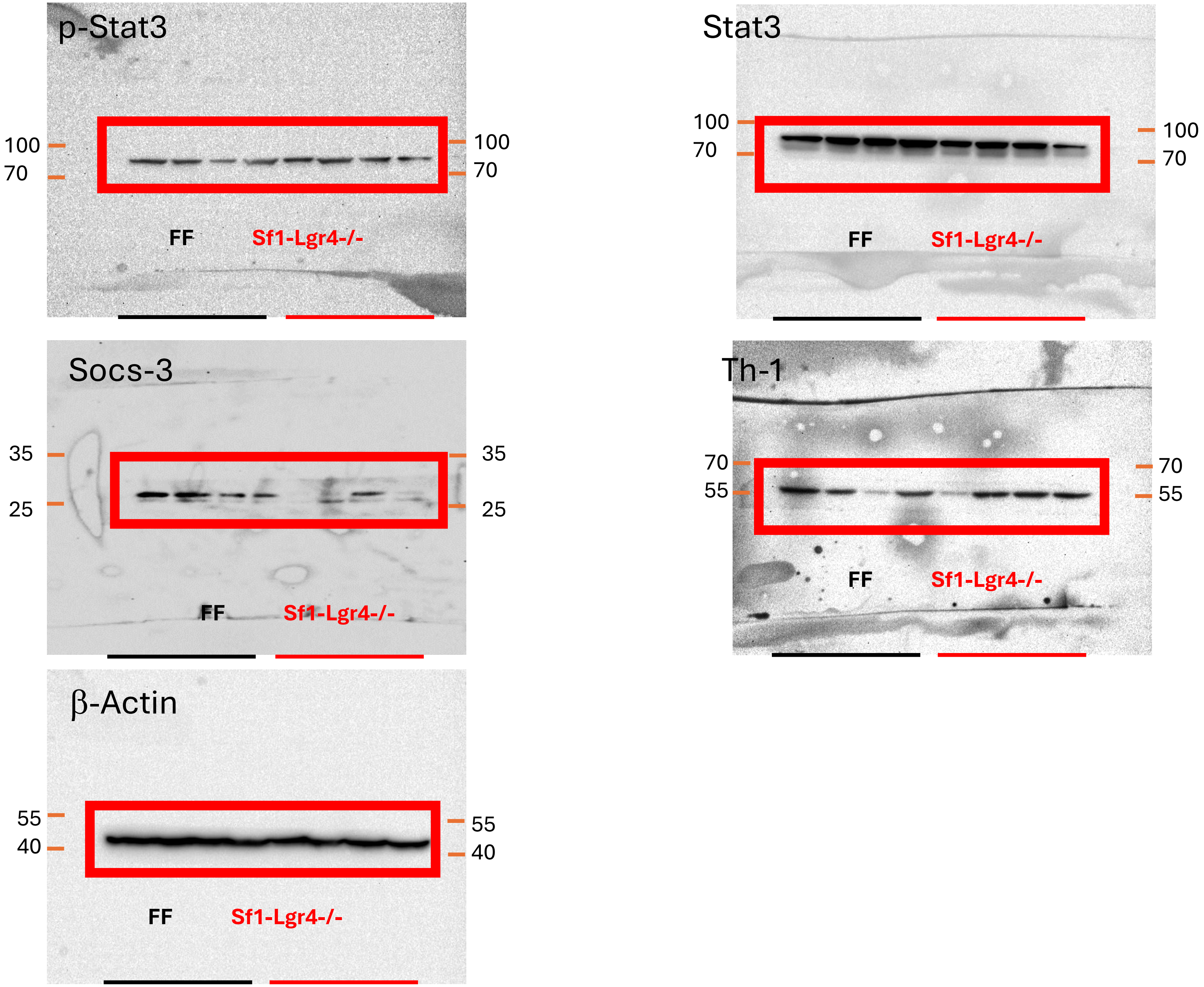

Figure 7 J Left Upper

Graph in figures

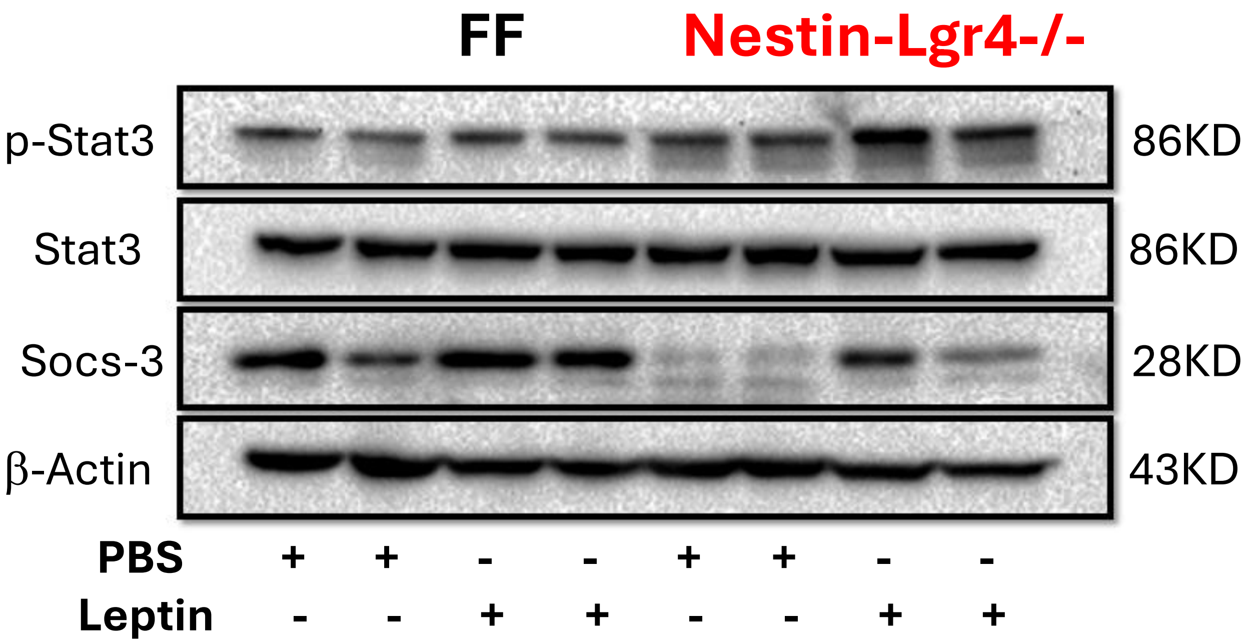

Corresponding uncropped images

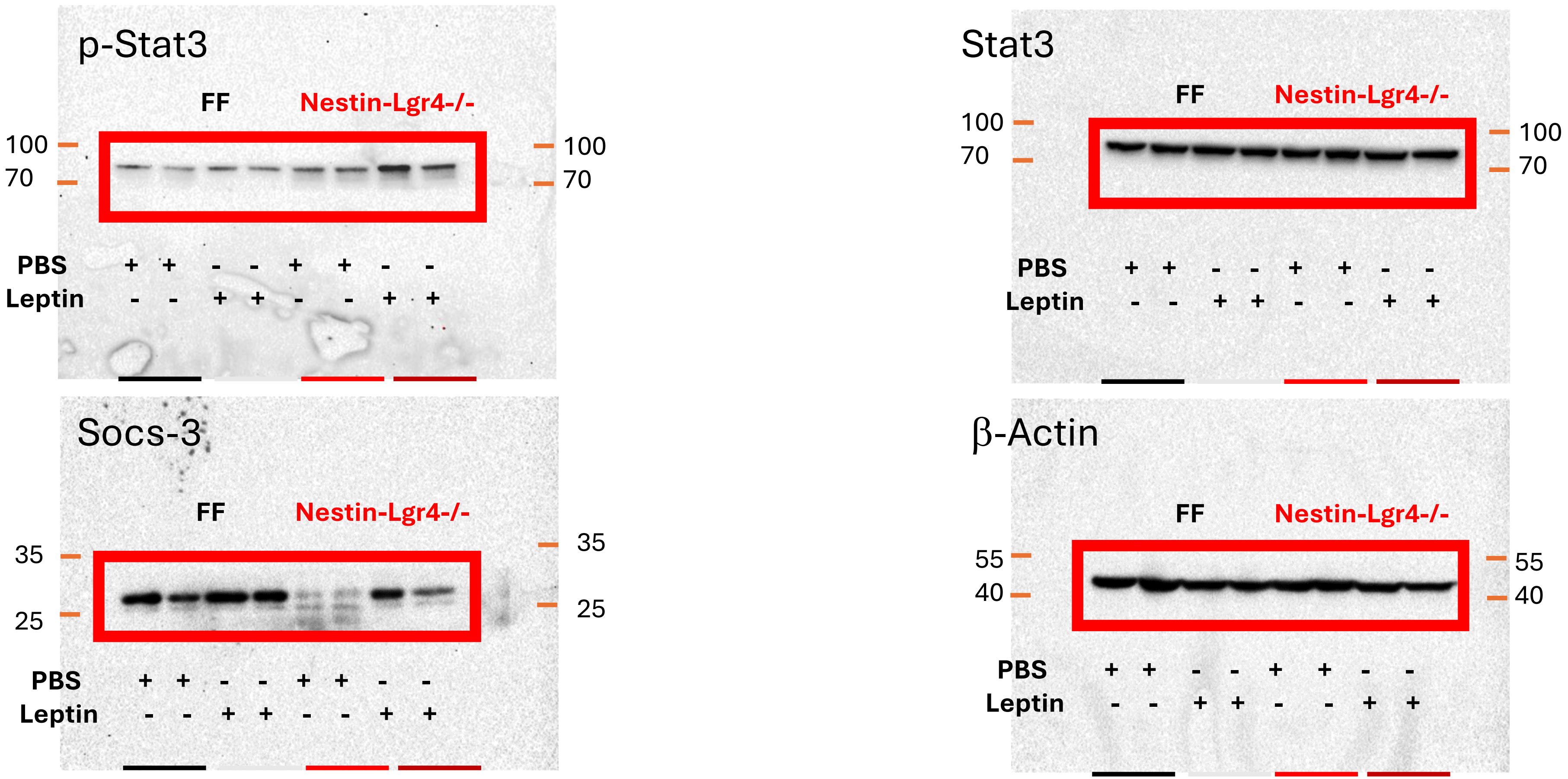

Figure 7 J Left Lower

Graph in figures

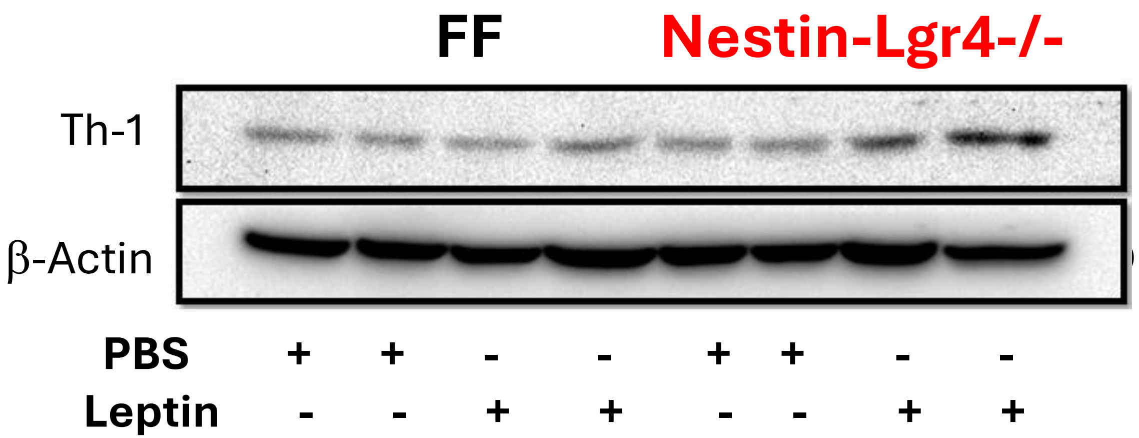

Corresponding uncropped images

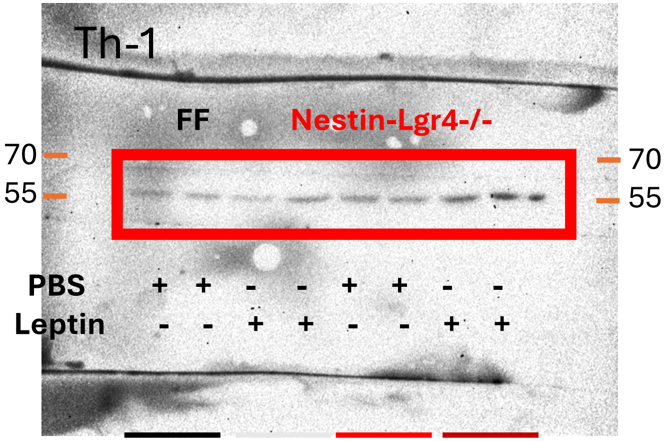

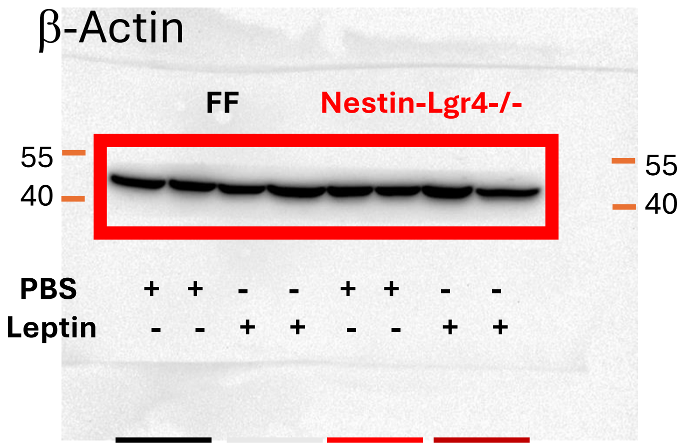

Figure 7 J Right Upper

Graph in figures

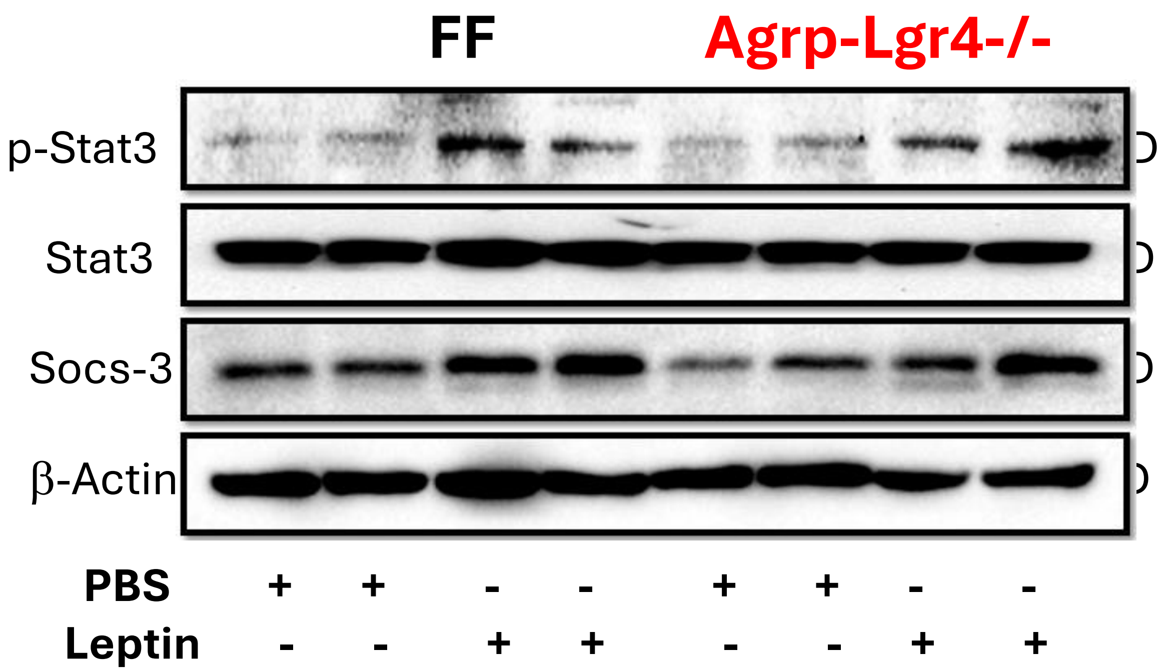

Corresponding uncropped images

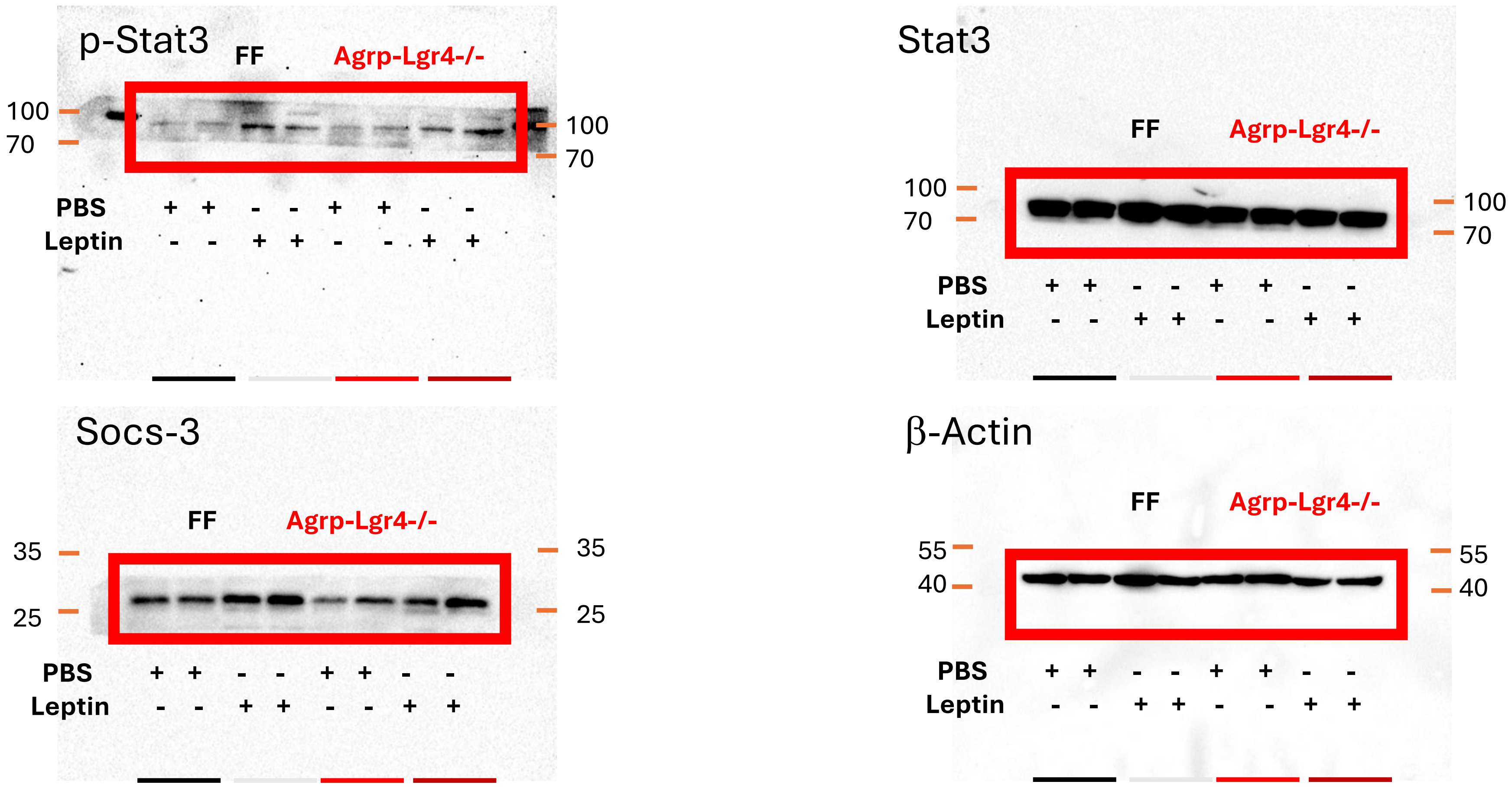

Figure 7 J Right Lower

Graph in figures

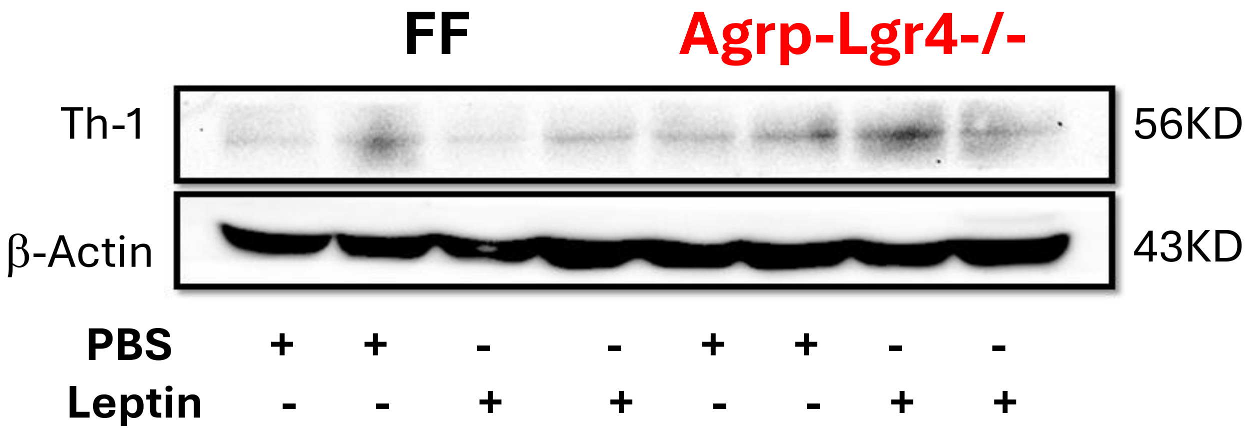

Corresponding uncropped images

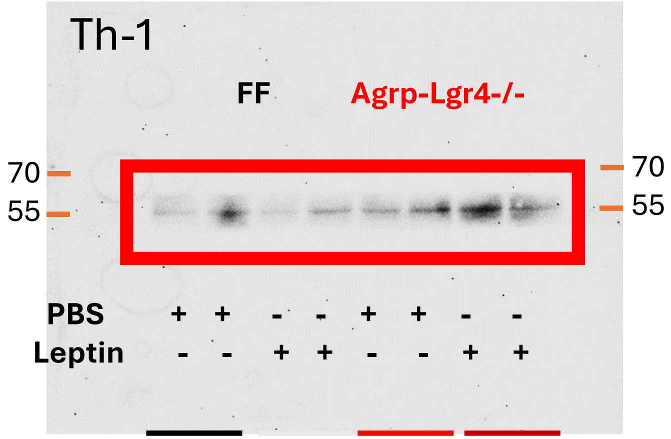

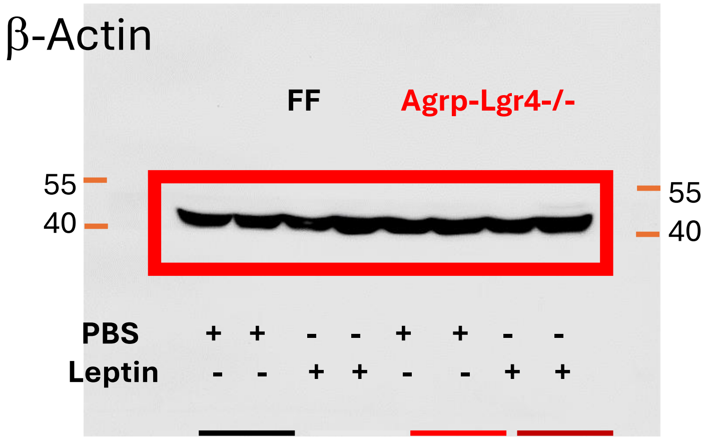

Figure 7 K Upper

Graph in figures

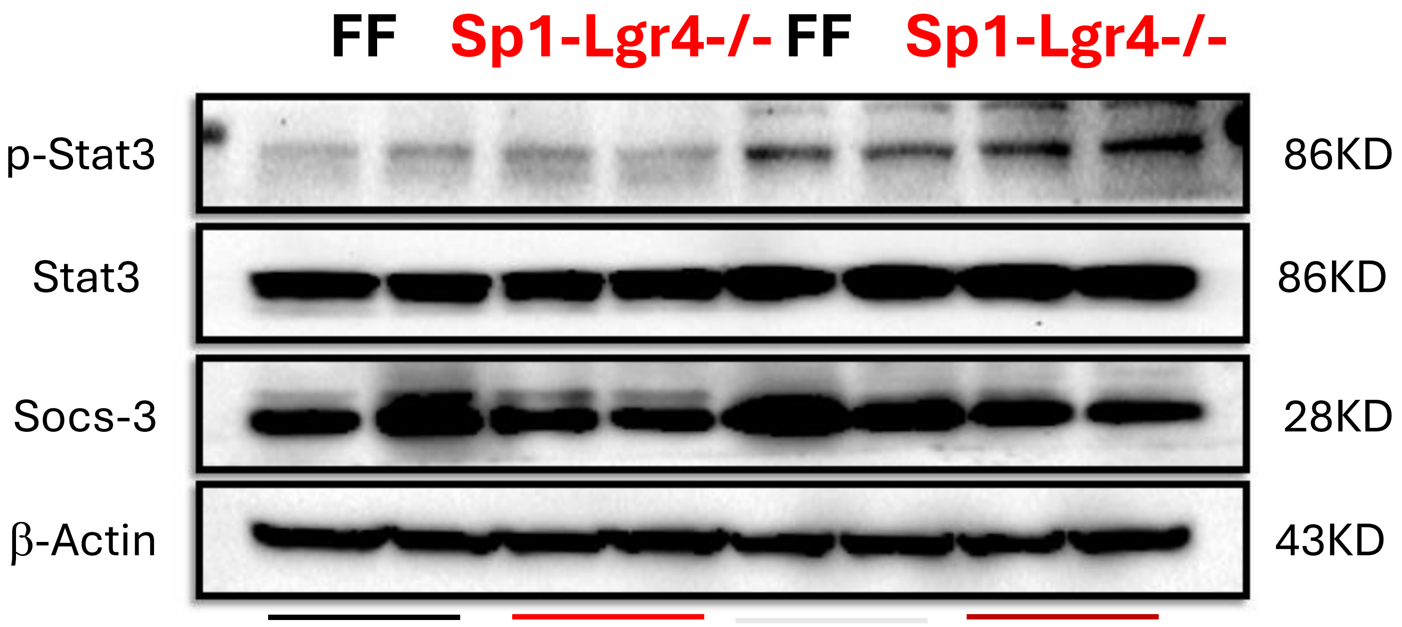

# Corresponding uncropped images

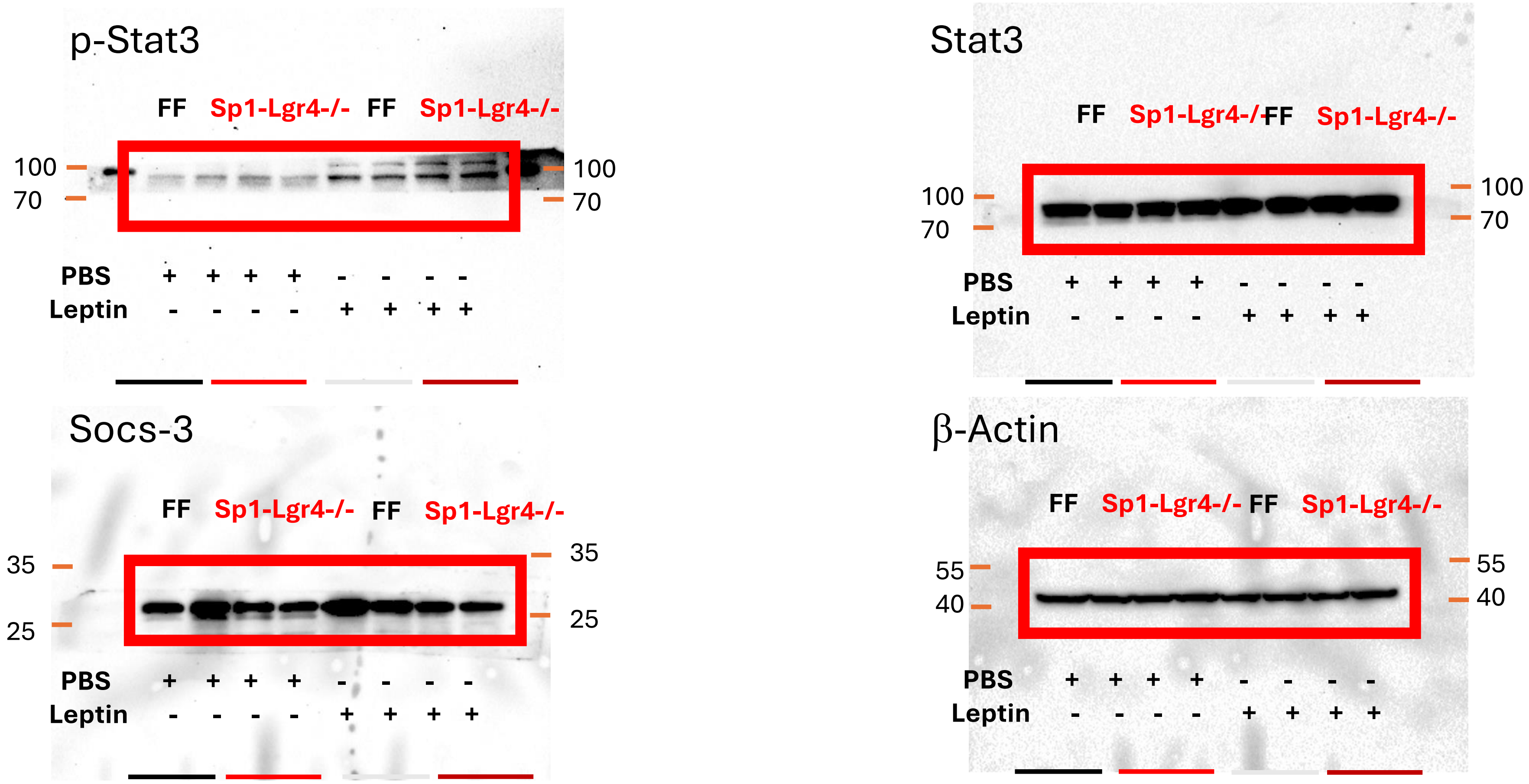

Figure 7 K Lower

Graph in figures

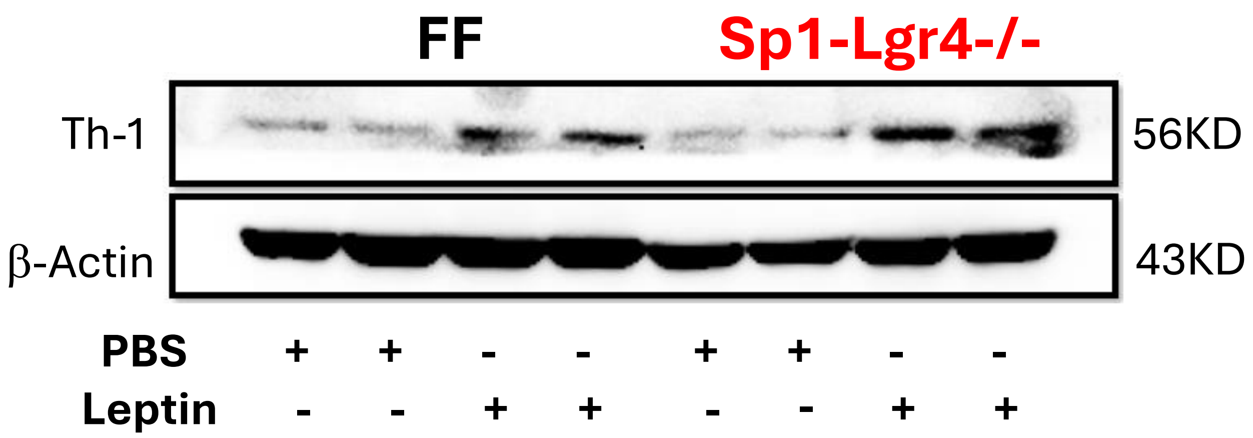

Corresponding uncropped images

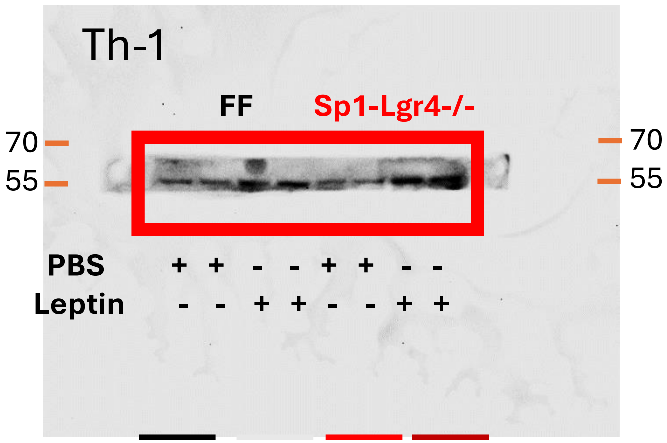

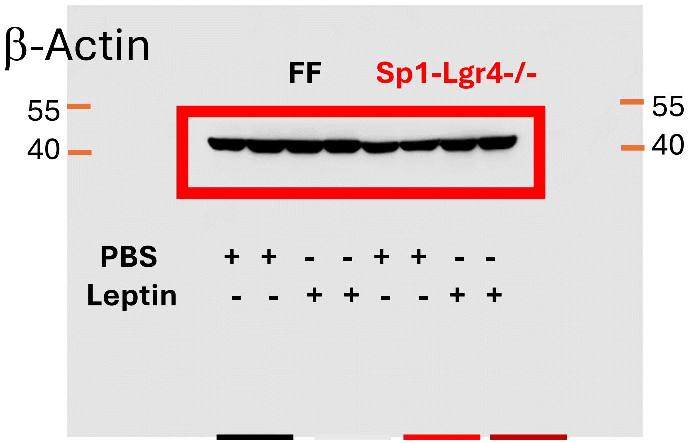

Figure 7 L

Graph in figures

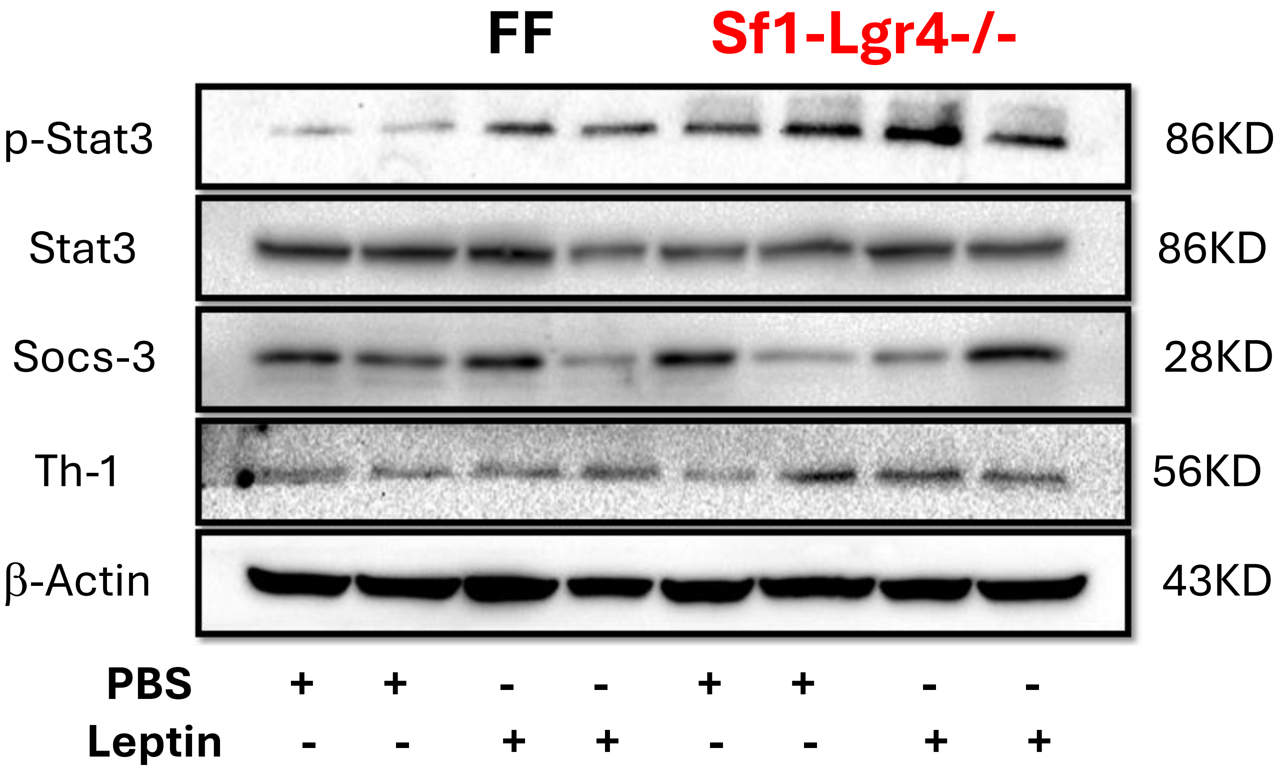

# Corresponding uncropped images

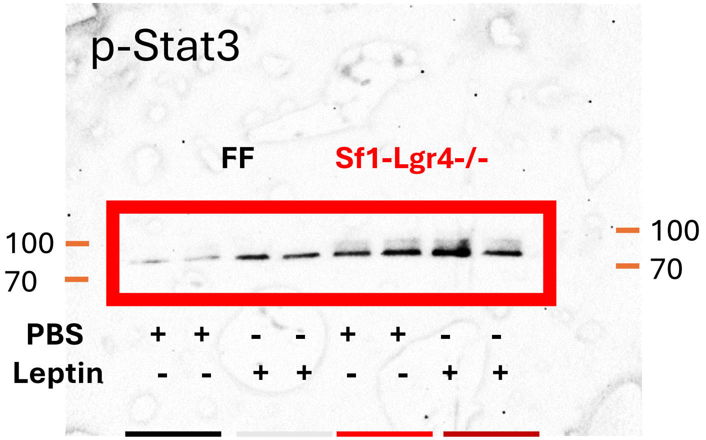

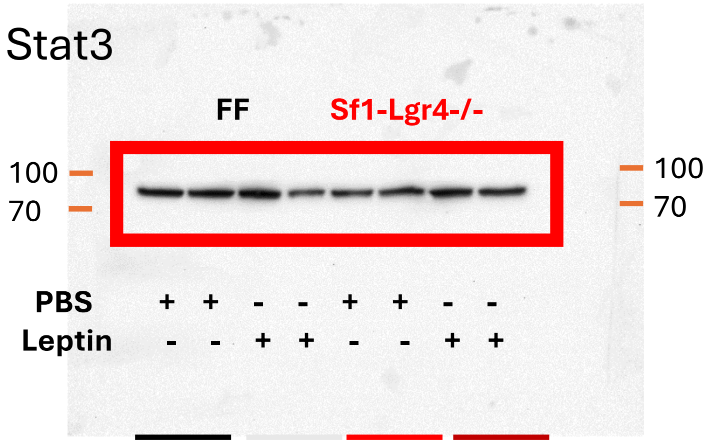

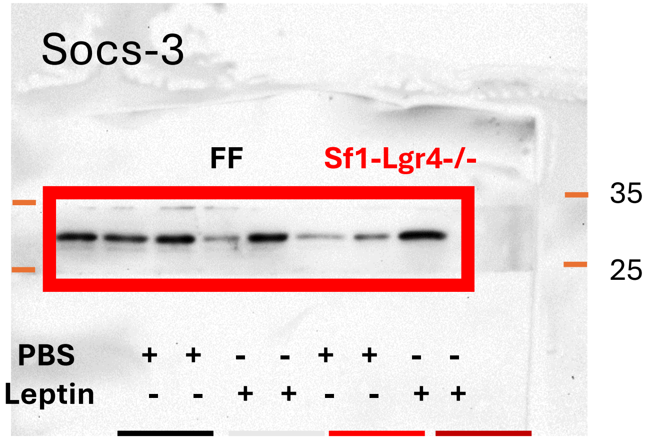

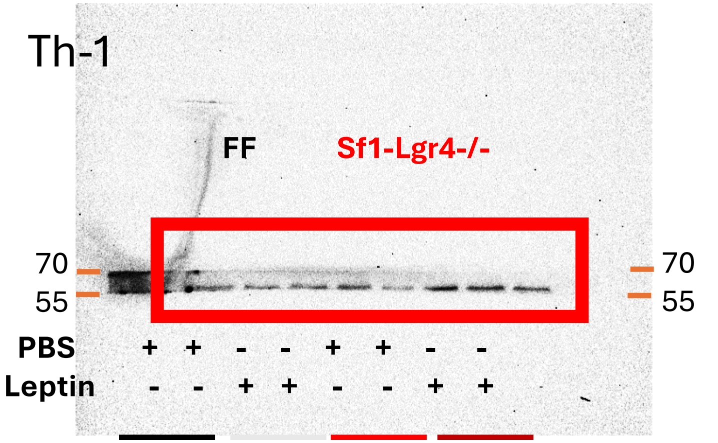

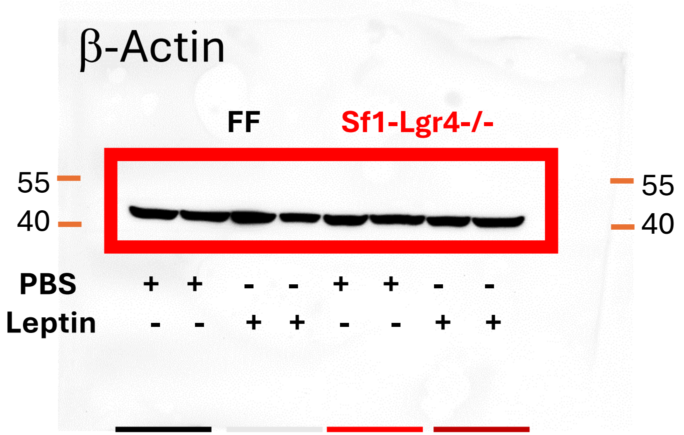

Supplement: Supplementary file 9 — Source data Fig. 7 [file 44319_2025_398_MOESM9_ESM.zip › Figure 7/Uncropped Western Blots of Figure 7.pdf]
