# Supplementary figures and images for: Deficiency of neuronal LGR4 increases energy expenditure and inhibits food intake via hypothalamic leptin signaling (part 2 of 2)
Source: EMBO Rep. 2025 Mar 11;26(8):2098–120. doi: 10.1038/s44319-025-00398-5 (PMC12018946; doi:10.1038/s44319-025-00398-5)

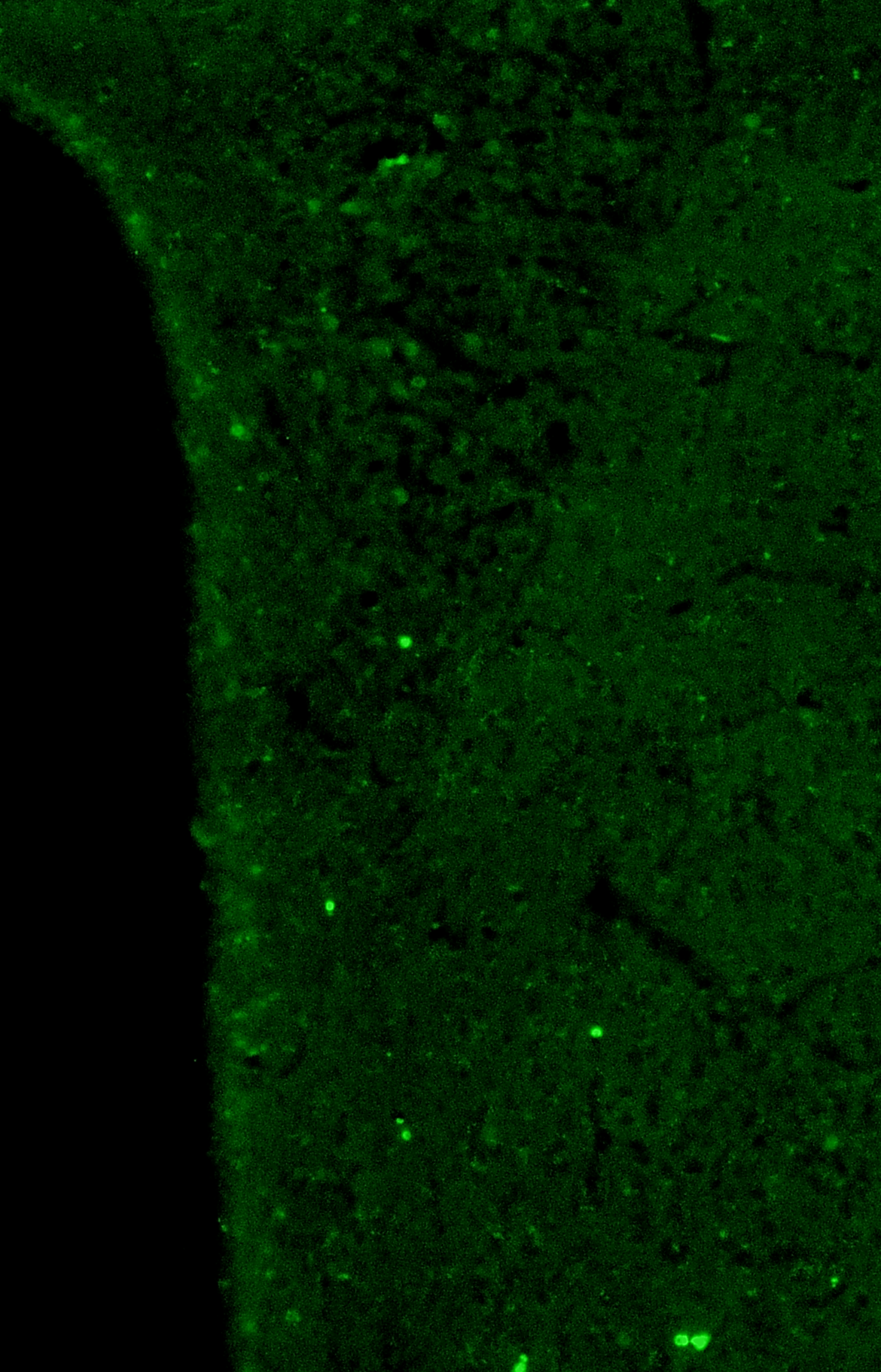

Supplement: Supplementary file 9 — Source data Fig. 7 [file 44319_2025_398_MOESM9_ESM.zip › Figure 7/Figure 7 G/FF-PVN.tif]

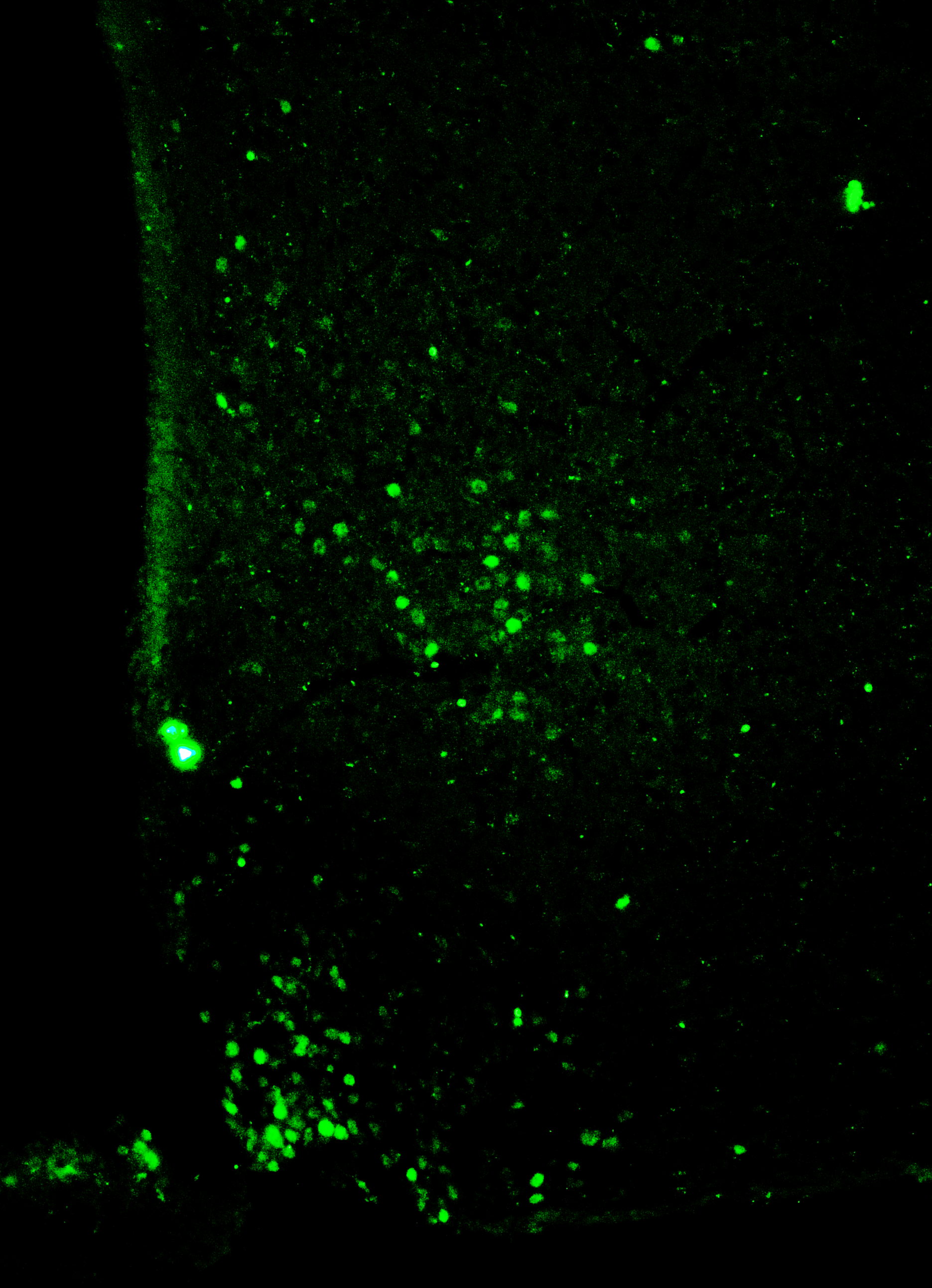

Supplement: Supplementary file 9 — Source data Fig. 7 [file 44319_2025_398_MOESM9_ESM.zip › Figure 7/Figure 7 G/KO-ARC VMH.tif]

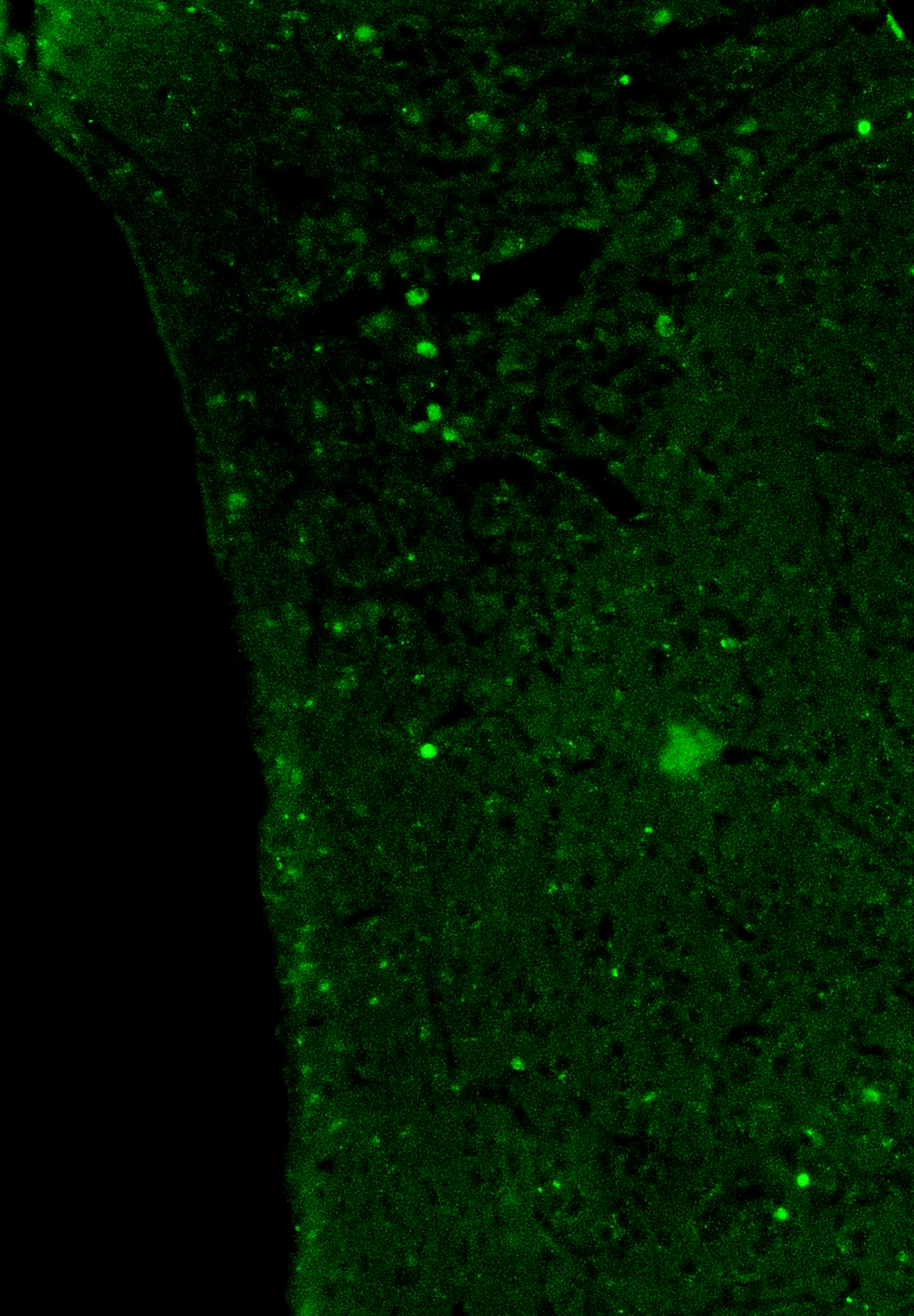

Supplement: Supplementary file 9 — Source data Fig. 7 [file 44319_2025_398_MOESM9_ESM.zip › Figure 7/Figure 7 G/KO-PVN.tif]

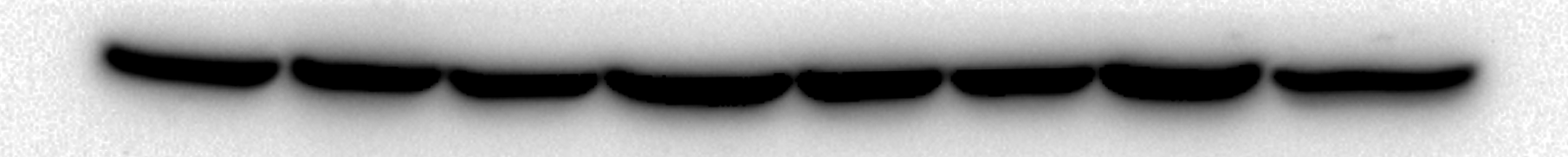

Supplement: Supplementary file 9 — Source data Fig. 7 [file 44319_2025_398_MOESM9_ESM.zip › Figure 7/Figure 7 J/Figure 7 J Left Lower/Western blot-actin.tif]

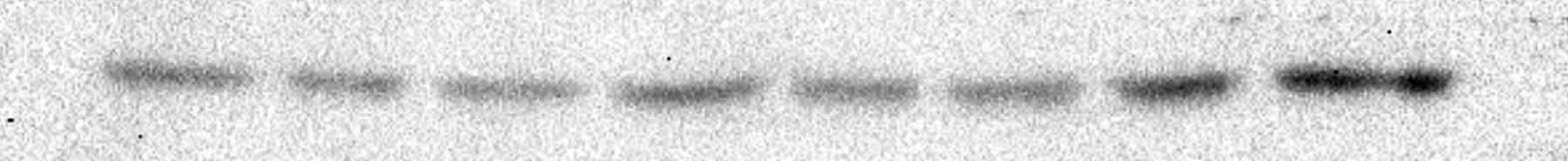

Supplement: Supplementary file 9 — Source data Fig. 7 [file 44319_2025_398_MOESM9_ESM.zip › Figure 7/Figure 7 J/Figure 7 J Left Lower/Western blot-th.tif]

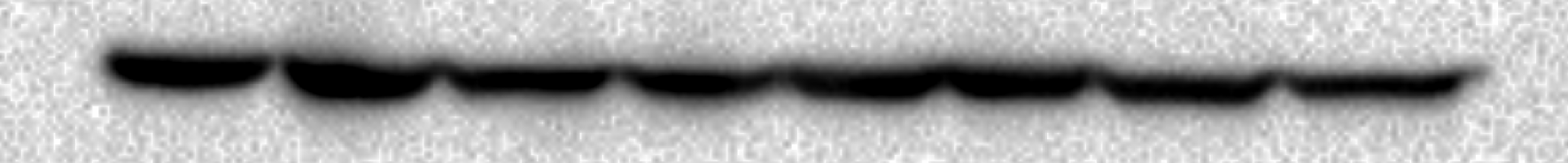

Supplement: Supplementary file 9 — Source data Fig. 7 [file 44319_2025_398_MOESM9_ESM.zip › Figure 7/Figure 7 J/Figure 7 J Left Upper/Western blot Actin.tif]

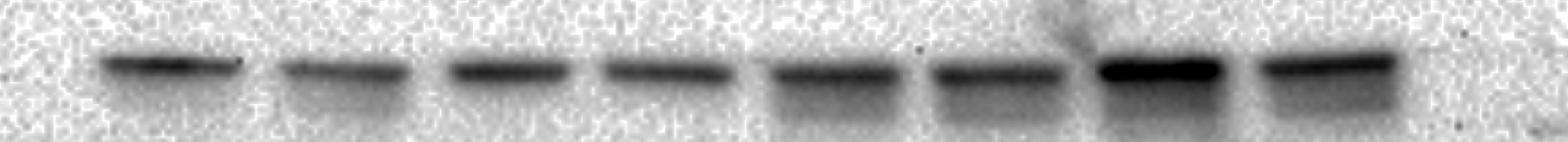

Supplement: Supplementary file 9 — Source data Fig. 7 [file 44319_2025_398_MOESM9_ESM.zip › Figure 7/Figure 7 J/Figure 7 J Left Upper/Western blot-p-Stat3.tif]

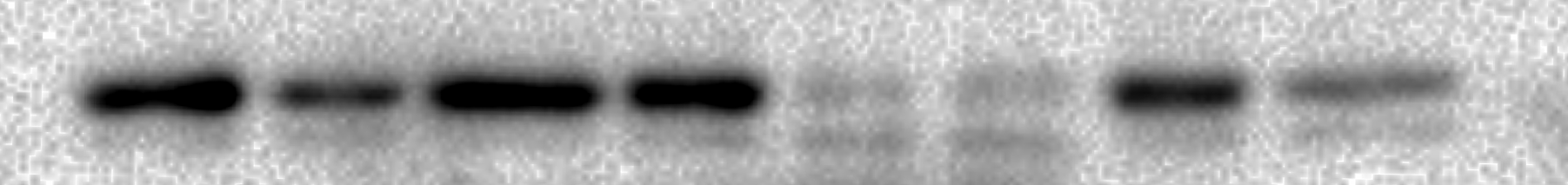

Supplement: Supplementary file 9 — Source data Fig. 7 [file 44319_2025_398_MOESM9_ESM.zip › Figure 7/Figure 7 J/Figure 7 J Left Upper/Western blot-socs-3.tif]

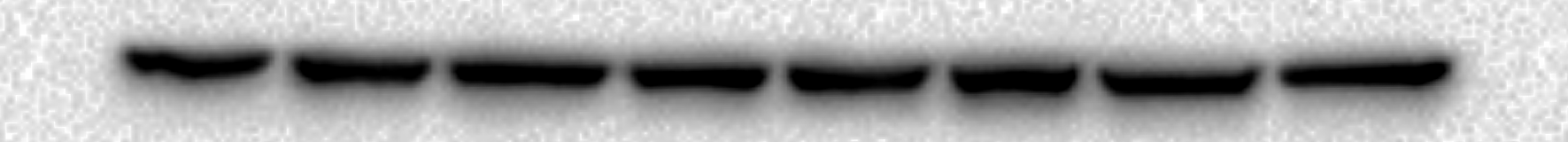

Supplement: Supplementary file 9 — Source data Fig. 7 [file 44319_2025_398_MOESM9_ESM.zip › Figure 7/Figure 7 J/Figure 7 J Left Upper/Western blot-Stat3.tif]

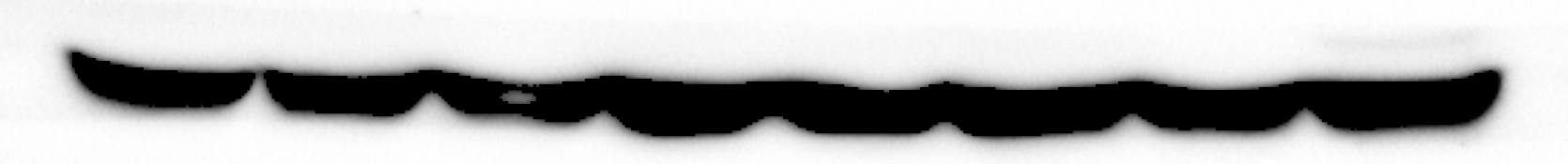

Supplement: Supplementary file 9 — Source data Fig. 7 [file 44319_2025_398_MOESM9_ESM.zip › Figure 7/Figure 7 J/Figure 7 J Right Lower/Western blot-Actin.tif]

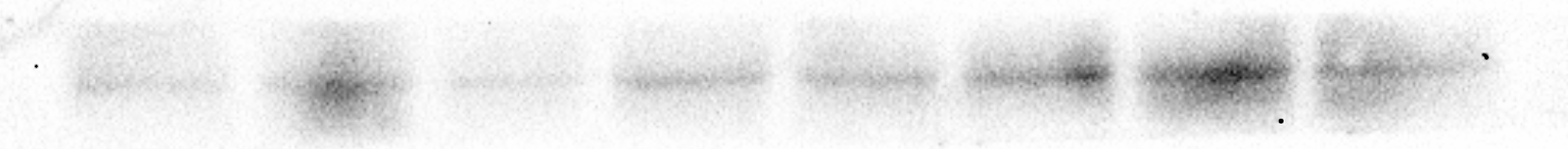

Supplement: Supplementary file 9 — Source data Fig. 7 [file 44319_2025_398_MOESM9_ESM.zip › Figure 7/Figure 7 J/Figure 7 J Right Lower/Western blot-Th-1.tif]

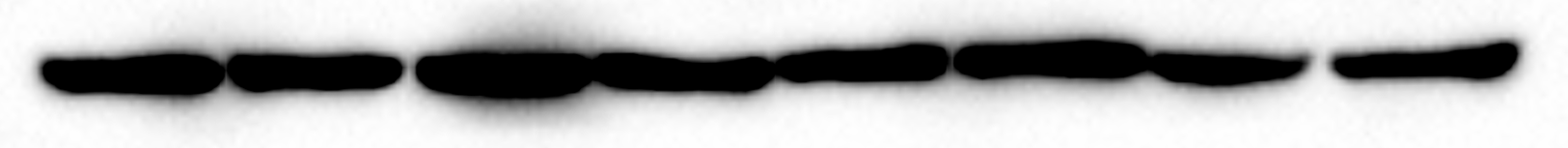

Supplement: Supplementary file 9 — Source data Fig. 7 [file 44319_2025_398_MOESM9_ESM.zip › Figure 7/Figure 7 J/Figure 7 J Right Upper/Western blot-Actin.tif]

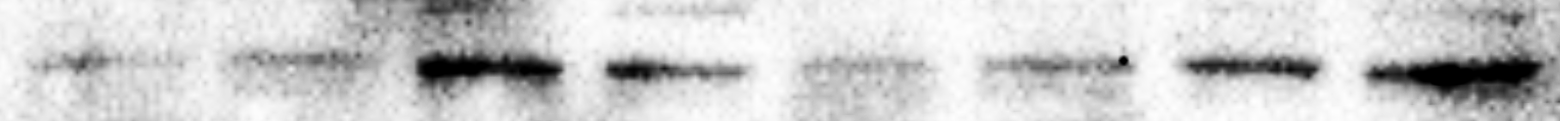

Supplement: Supplementary file 9 — Source data Fig. 7 [file 44319_2025_398_MOESM9_ESM.zip › Figure 7/Figure 7 J/Figure 7 J Right Upper/Western blot-p-Stat3.tif]

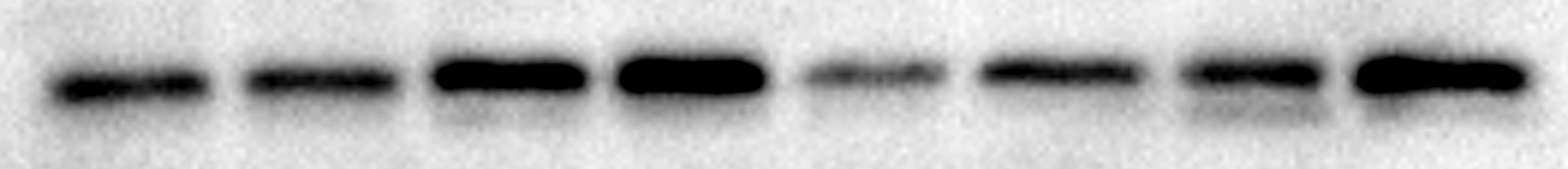

Supplement: Supplementary file 9 — Source data Fig. 7 [file 44319_2025_398_MOESM9_ESM.zip › Figure 7/Figure 7 J/Figure 7 J Right Upper/Western blot-Socs-3.tif]

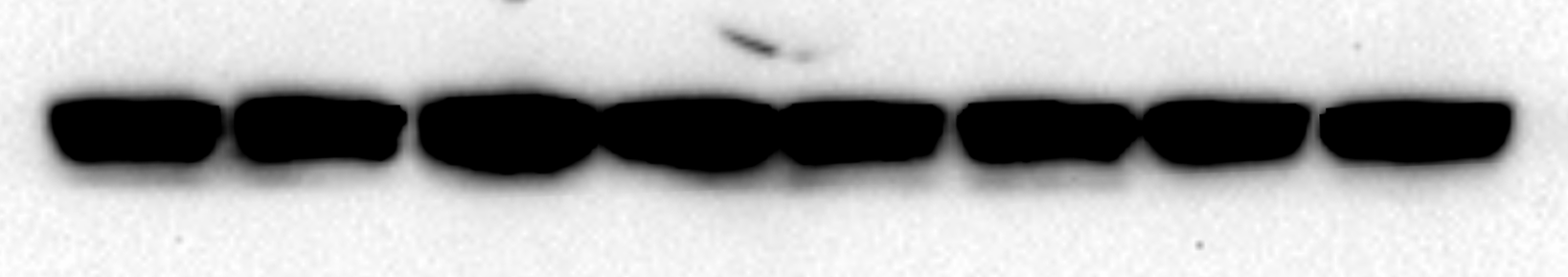

Supplement: Supplementary file 9 — Source data Fig. 7 [file 44319_2025_398_MOESM9_ESM.zip › Figure 7/Figure 7 J/Figure 7 J Right Upper/Western blot-Stat3.tif]

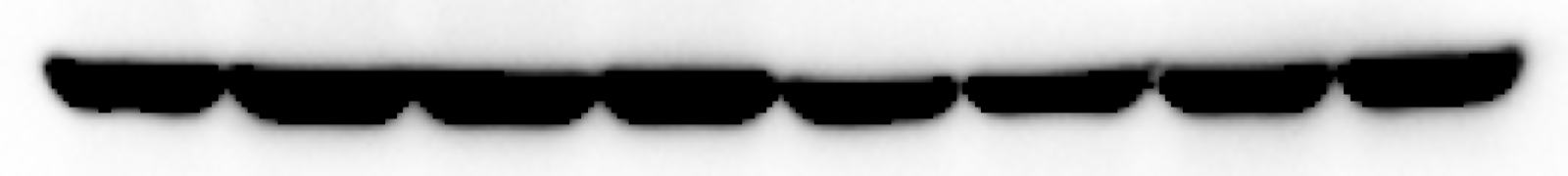

Supplement: Supplementary file 9 — Source data Fig. 7 [file 44319_2025_398_MOESM9_ESM.zip › Figure 7/Figure 7 K/Figure 7 K Lower/Western blot-Actin.tif]

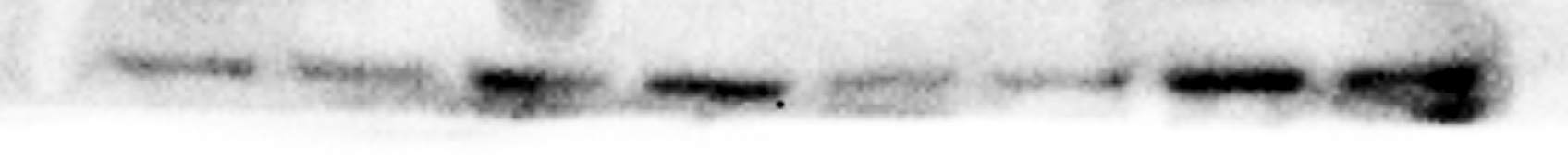

Supplement: Supplementary file 9 — Source data Fig. 7 [file 44319_2025_398_MOESM9_ESM.zip › Figure 7/Figure 7 K/Figure 7 K Lower/Western blot-Th-1.tif]

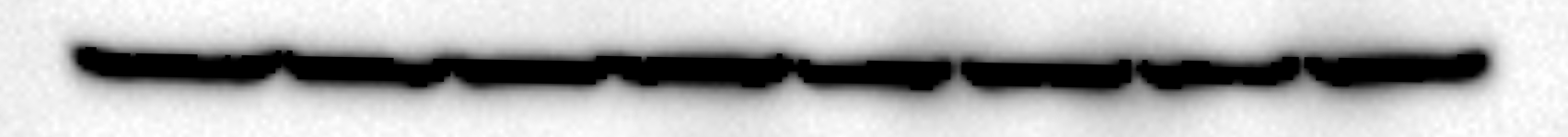

Supplement: Supplementary file 9 — Source data Fig. 7 [file 44319_2025_398_MOESM9_ESM.zip › Figure 7/Figure 7 K/Figure 7 K Upper/Western blot-Actin.tif]

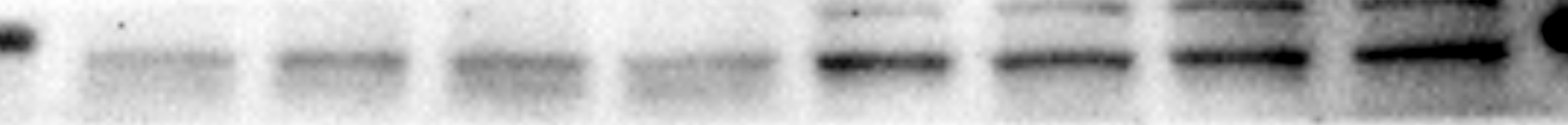

Supplement: Supplementary file 9 — Source data Fig. 7 [file 44319_2025_398_MOESM9_ESM.zip › Figure 7/Figure 7 K/Figure 7 K Upper/Western blot-p-Stat3.tif]

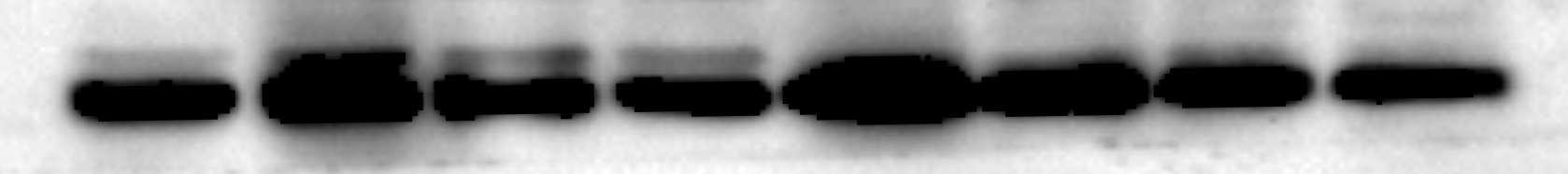

Supplement: Supplementary file 9 — Source data Fig. 7 [file 44319_2025_398_MOESM9_ESM.zip › Figure 7/Figure 7 K/Figure 7 K Upper/Western blot-Socs3.tif]

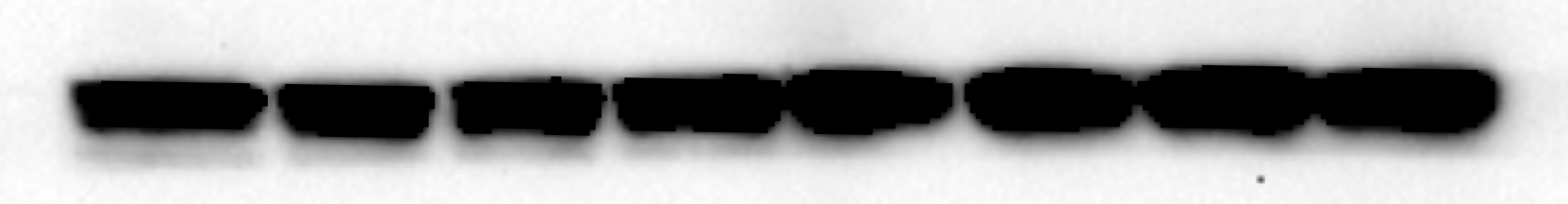

Supplement: Supplementary file 9 — Source data Fig. 7 [file 44319_2025_398_MOESM9_ESM.zip › Figure 7/Figure 7 K/Figure 7 K Upper/Western blot-Stat3.tif]

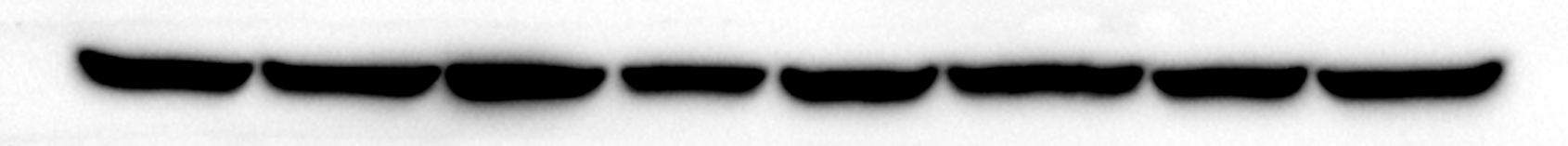

Supplement: Supplementary file 9 — Source data Fig. 7 [file 44319_2025_398_MOESM9_ESM.zip › Figure 7/Figure 7 L/Western blot-Actin.tif]

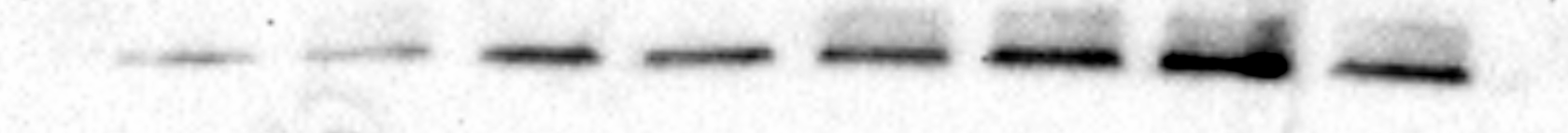

Supplement: Supplementary file 9 — Source data Fig. 7 [file 44319_2025_398_MOESM9_ESM.zip › Figure 7/Figure 7 L/Western blot-p-Stat3.tif]

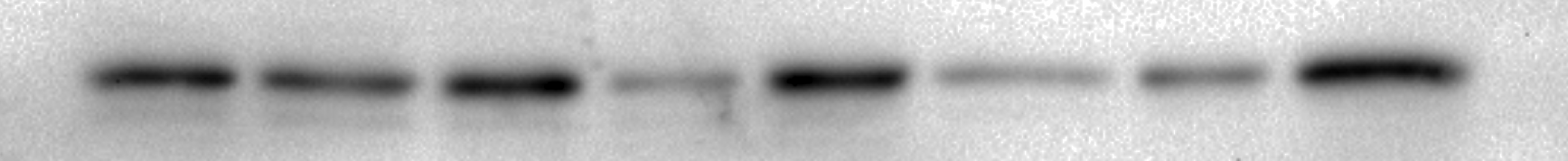

Supplement: Supplementary file 9 — Source data Fig. 7 [file 44319_2025_398_MOESM9_ESM.zip › Figure 7/Figure 7 L/Western blot-Socs-3.tif]

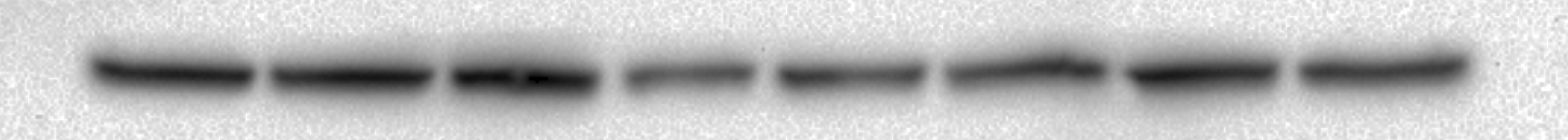

Supplement: Supplementary file 9 — Source data Fig. 7 [file 44319_2025_398_MOESM9_ESM.zip › Figure 7/Figure 7 L/Western blot-Stat3.tif]

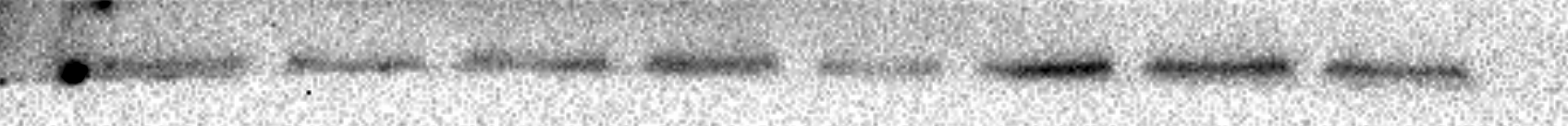

Supplement: Supplementary file 9 — Source data Fig. 7 [file 44319_2025_398_MOESM9_ESM.zip › Figure 7/Figure 7 L/Western blot-Th-1.tif]

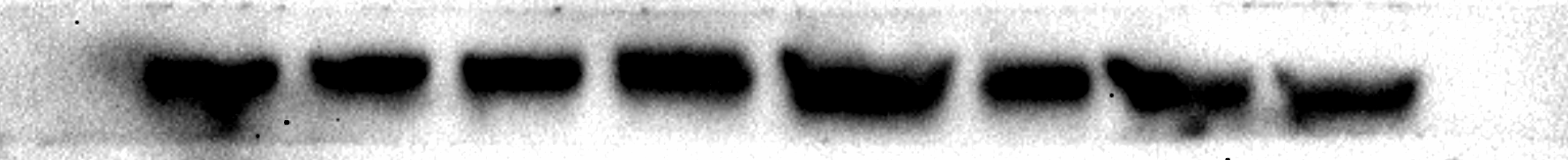

Supplement: Supplementary file 10 — Source data Fig. 8 [file 44319_2025_398_MOESM10_ESM.zip › Figure 8/Figure 8 A/Western blot-Actin.tif]

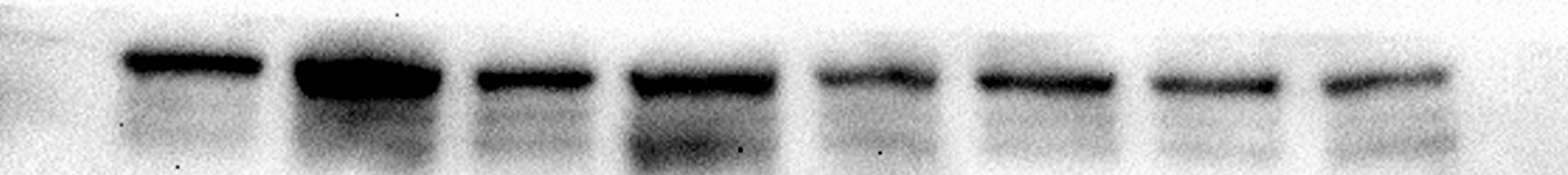

Supplement: Supplementary file 10 — Source data Fig. 8 [file 44319_2025_398_MOESM10_ESM.zip › Figure 8/Figure 8 A/Western blot-bcatenin.tif]

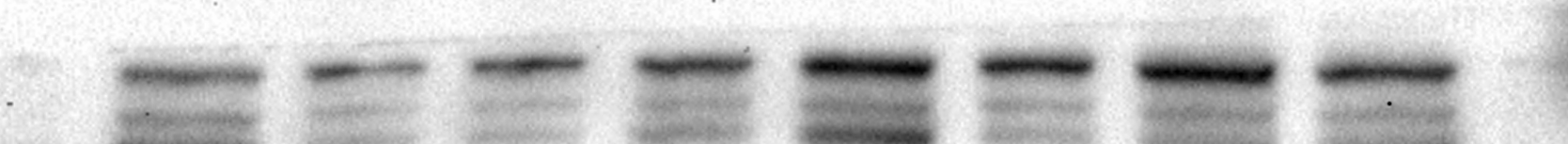

Supplement: Supplementary file 10 — Source data Fig. 8 [file 44319_2025_398_MOESM10_ESM.zip › Figure 8/Figure 8 A/Western blot-p-bcatenin.tif]

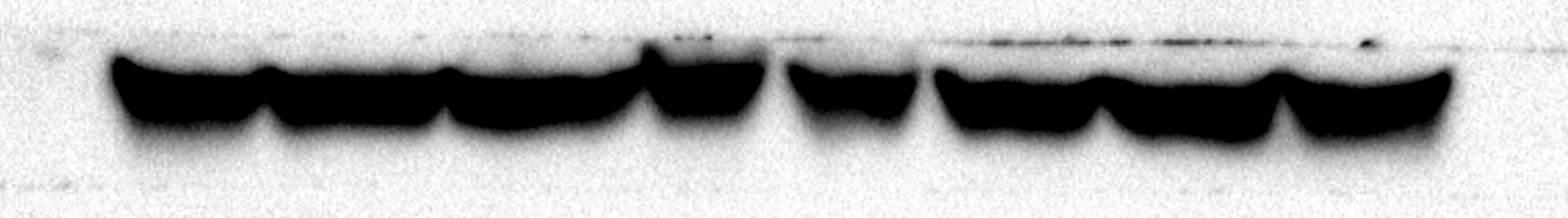

Supplement: Supplementary file 10 — Source data Fig. 8 [file 44319_2025_398_MOESM10_ESM.zip › Figure 8/Figure 8 B/Western blot-Actin.tif]

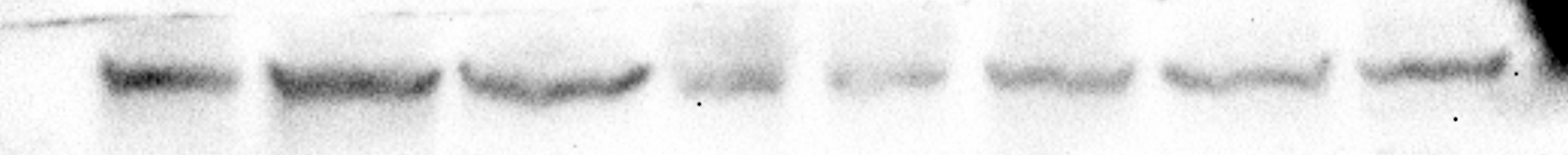

Supplement: Supplementary file 10 — Source data Fig. 8 [file 44319_2025_398_MOESM10_ESM.zip › Figure 8/Figure 8 B/Western blot-b-catenin.tif]

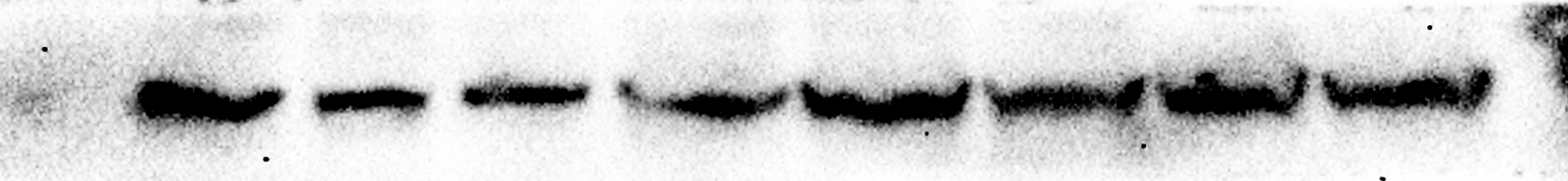

Supplement: Supplementary file 10 — Source data Fig. 8 [file 44319_2025_398_MOESM10_ESM.zip › Figure 8/Figure 8 B/Western blot-p-b-Catenin.tif]

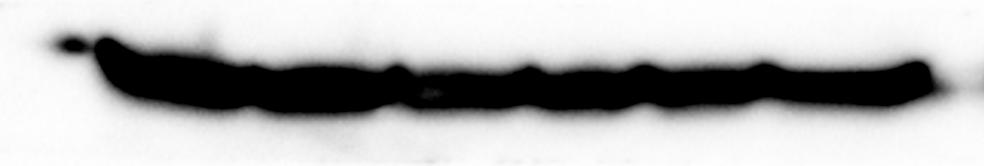

Supplement: Supplementary file 10 — Source data Fig. 8 [file 44319_2025_398_MOESM10_ESM.zip › Figure 8/Figure 8 C/Western blot-Actin.tif]

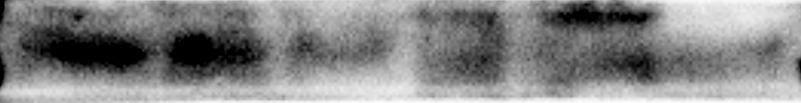

Supplement: Supplementary file 10 — Source data Fig. 8 [file 44319_2025_398_MOESM10_ESM.zip › Figure 8/Figure 8 C/Western blot-bcatenin.tif]

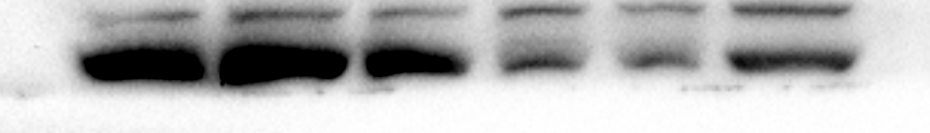

Supplement: Supplementary file 10 — Source data Fig. 8 [file 44319_2025_398_MOESM10_ESM.zip › Figure 8/Figure 8 C/Western blot-Lgr4.tif]

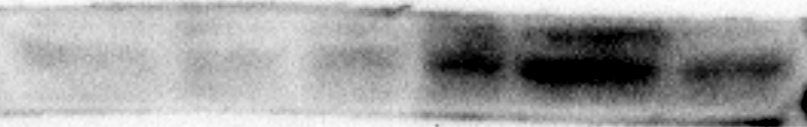

Supplement: Supplementary file 10 — Source data Fig. 8 [file 44319_2025_398_MOESM10_ESM.zip › Figure 8/Figure 8 C/Western blot-p-bcatenin.tif]

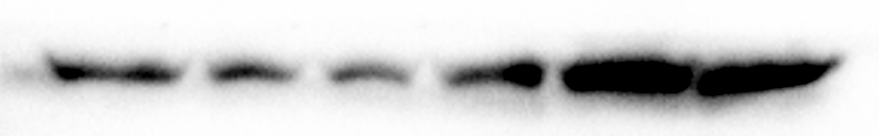

Supplement: Supplementary file 10 — Source data Fig. 8 [file 44319_2025_398_MOESM10_ESM.zip › Figure 8/Figure 8 C/Western blot-p-stat3.tif]

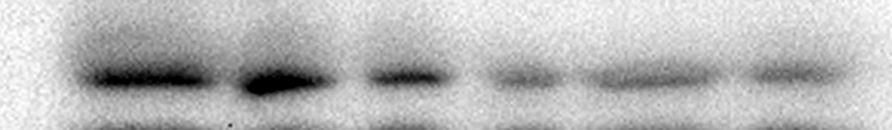

Supplement: Supplementary file 10 — Source data Fig. 8 [file 44319_2025_398_MOESM10_ESM.zip › Figure 8/Figure 8 C/Western blot-socs-3.tif]

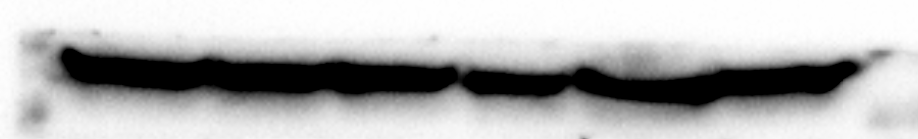

Supplement: Supplementary file 10 — Source data Fig. 8 [file 44319_2025_398_MOESM10_ESM.zip › Figure 8/Figure 8 C/Western blot-Stat3.tif]

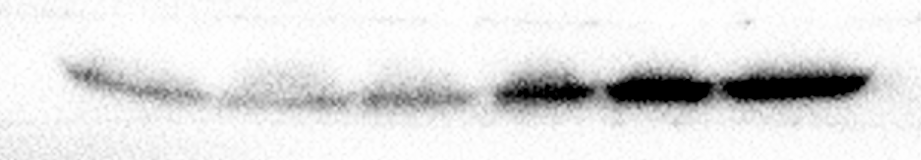

Supplement: Supplementary file 10 — Source data Fig. 8 [file 44319_2025_398_MOESM10_ESM.zip › Figure 8/Figure 8 C/Western blot-Th-1.tif]

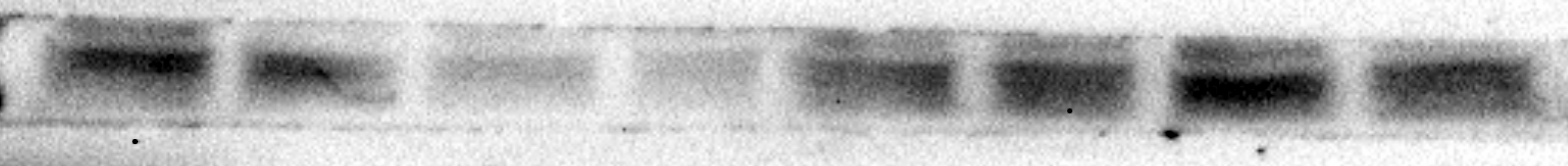

Supplement: Supplementary file 10 — Source data Fig. 8 [file 44319_2025_398_MOESM10_ESM.zip › Figure 8/Figure 8 D/Wb-p-b-catenin.tif]

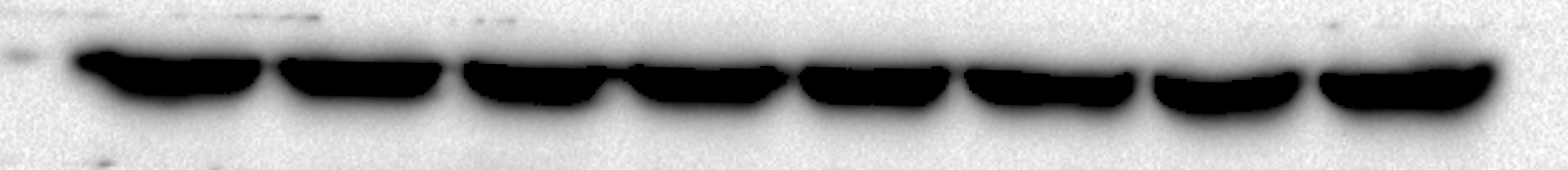

Supplement: Supplementary file 10 — Source data Fig. 8 [file 44319_2025_398_MOESM10_ESM.zip › Figure 8/Figure 8 D/Western blot-Actin.tif]

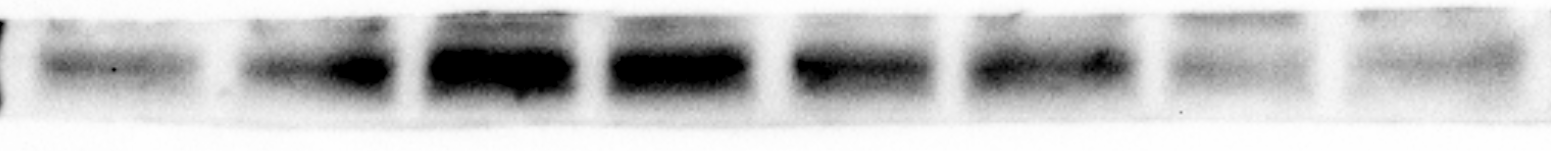

Supplement: Supplementary file 10 — Source data Fig. 8 [file 44319_2025_398_MOESM10_ESM.zip › Figure 8/Figure 8 D/Western blot-b-catenin.tif]

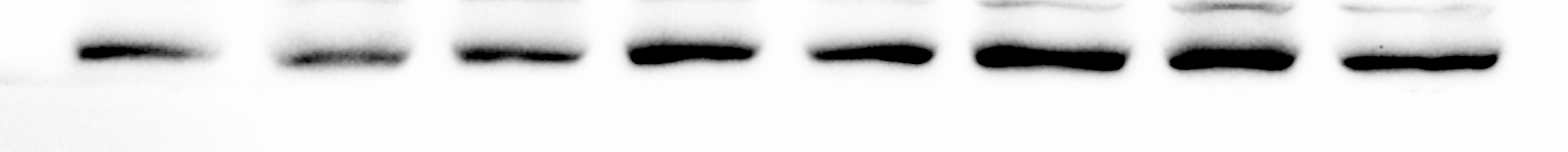

Supplement: Supplementary file 10 — Source data Fig. 8 [file 44319_2025_398_MOESM10_ESM.zip › Figure 8/Figure 8 D/Western blot-Lgr4.tif]

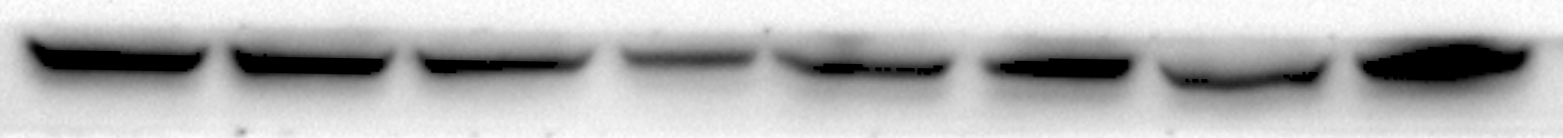

Supplement: Supplementary file 10 — Source data Fig. 8 [file 44319_2025_398_MOESM10_ESM.zip › Figure 8/Figure 8 D/Western blot-p-Stat3.tif]

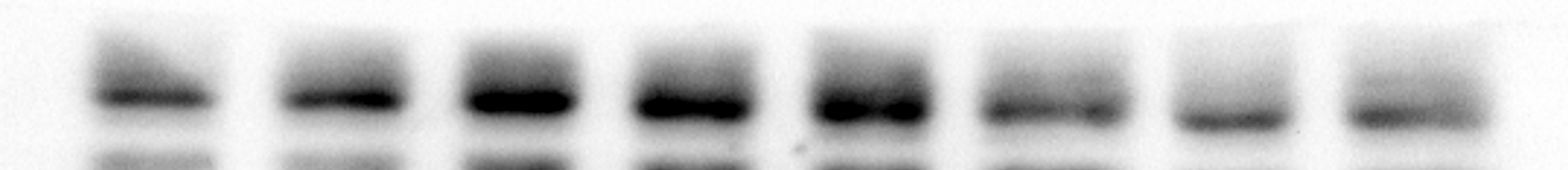

Supplement: Supplementary file 10 — Source data Fig. 8 [file 44319_2025_398_MOESM10_ESM.zip › Figure 8/Figure 8 D/Western blot-Socs-3.tif]

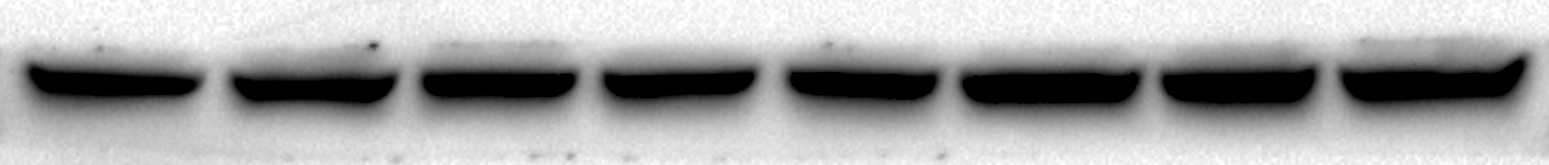

Supplement: Supplementary file 10 — Source data Fig. 8 [file 44319_2025_398_MOESM10_ESM.zip › Figure 8/Figure 8 D/Western blot-Stat3.tif]

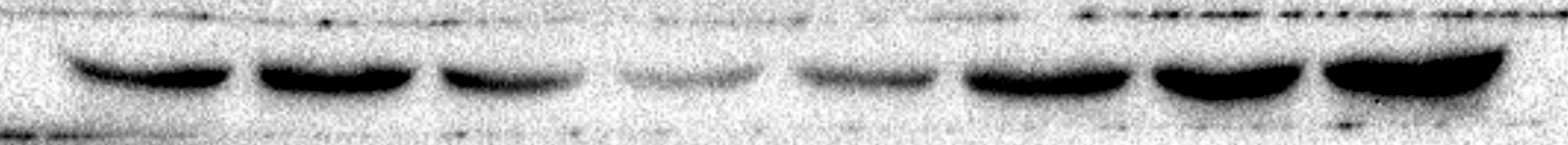

Supplement: Supplementary file 10 — Source data Fig. 8 [file 44319_2025_398_MOESM10_ESM.zip › Figure 8/Figure 8 D/Western blot-Th-1.tif]

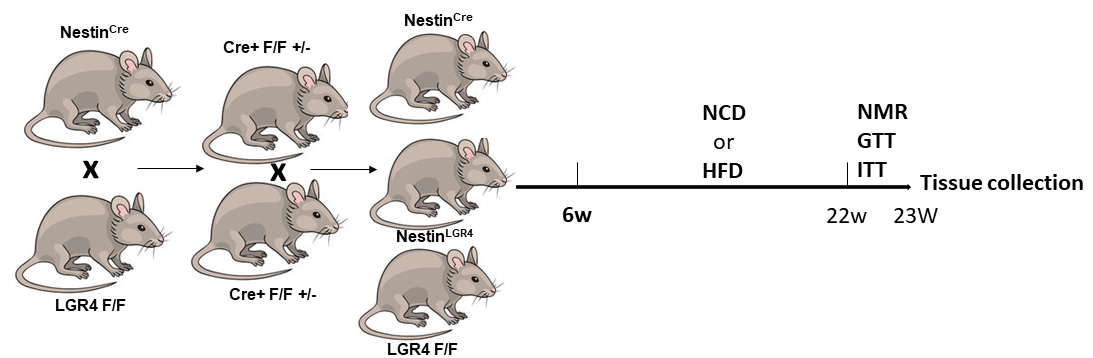

Supplement: Supplementary file 11 — EV and Appendix Figures Source Data [file 44319_2025_398_MOESM11_ESM.zip › Expand View Figures Source Data/Appendix Figures Source Data/Appendix Figure 1/Appendix Figure 1 A/work flow.tif]

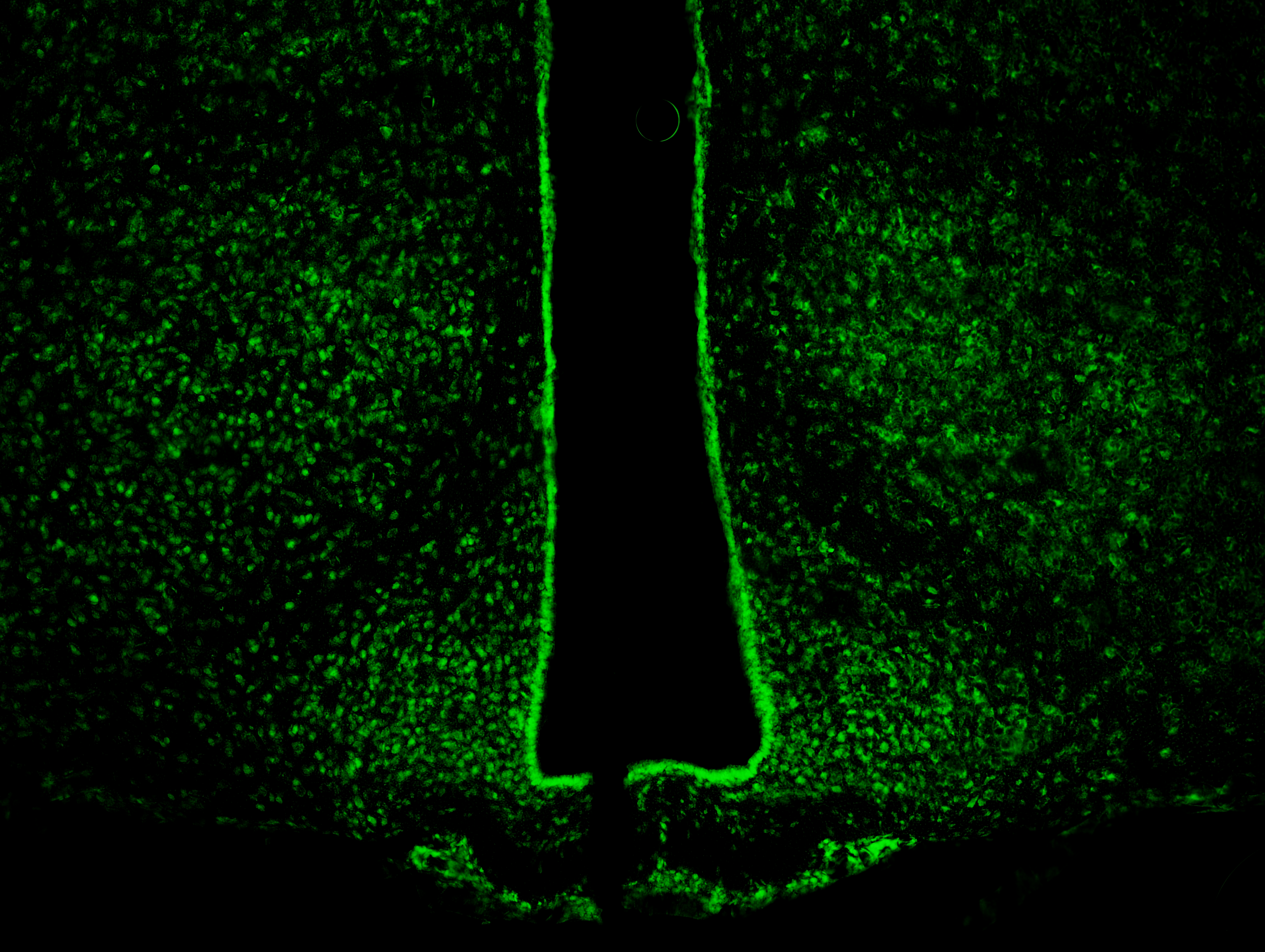

Supplement: Supplementary file 11 — EV and Appendix Figures Source Data [file 44319_2025_398_MOESM11_ESM.zip › Expand View Figures Source Data/Appendix Figures Source Data/Appendix Figure 3/Appendix Figure 3A/FF-ARC VMH.tif]

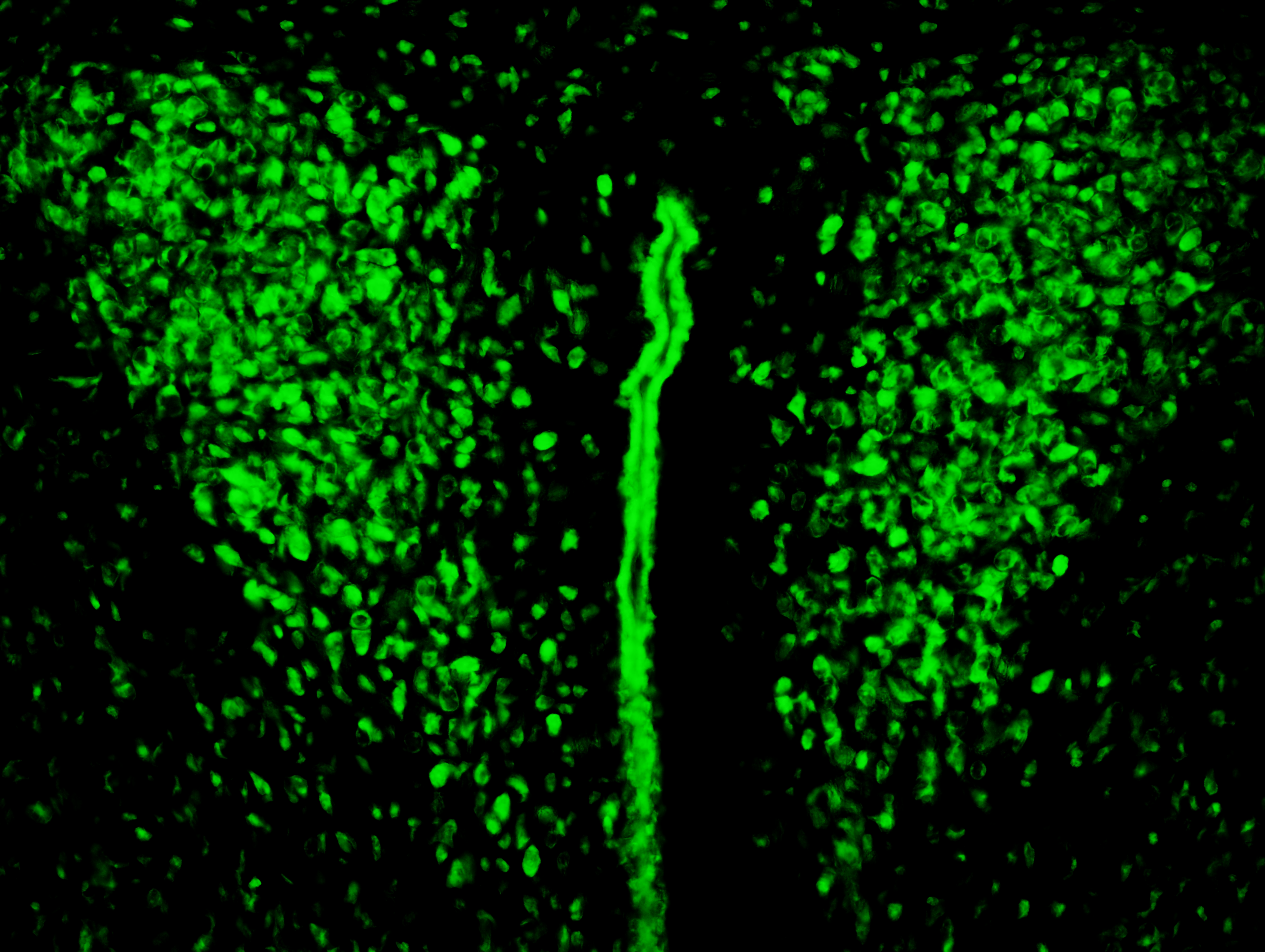

Supplement: Supplementary file 11 — EV and Appendix Figures Source Data [file 44319_2025_398_MOESM11_ESM.zip › Expand View Figures Source Data/Appendix Figures Source Data/Appendix Figure 3/Appendix Figure 3A/FF-PVN.tif]

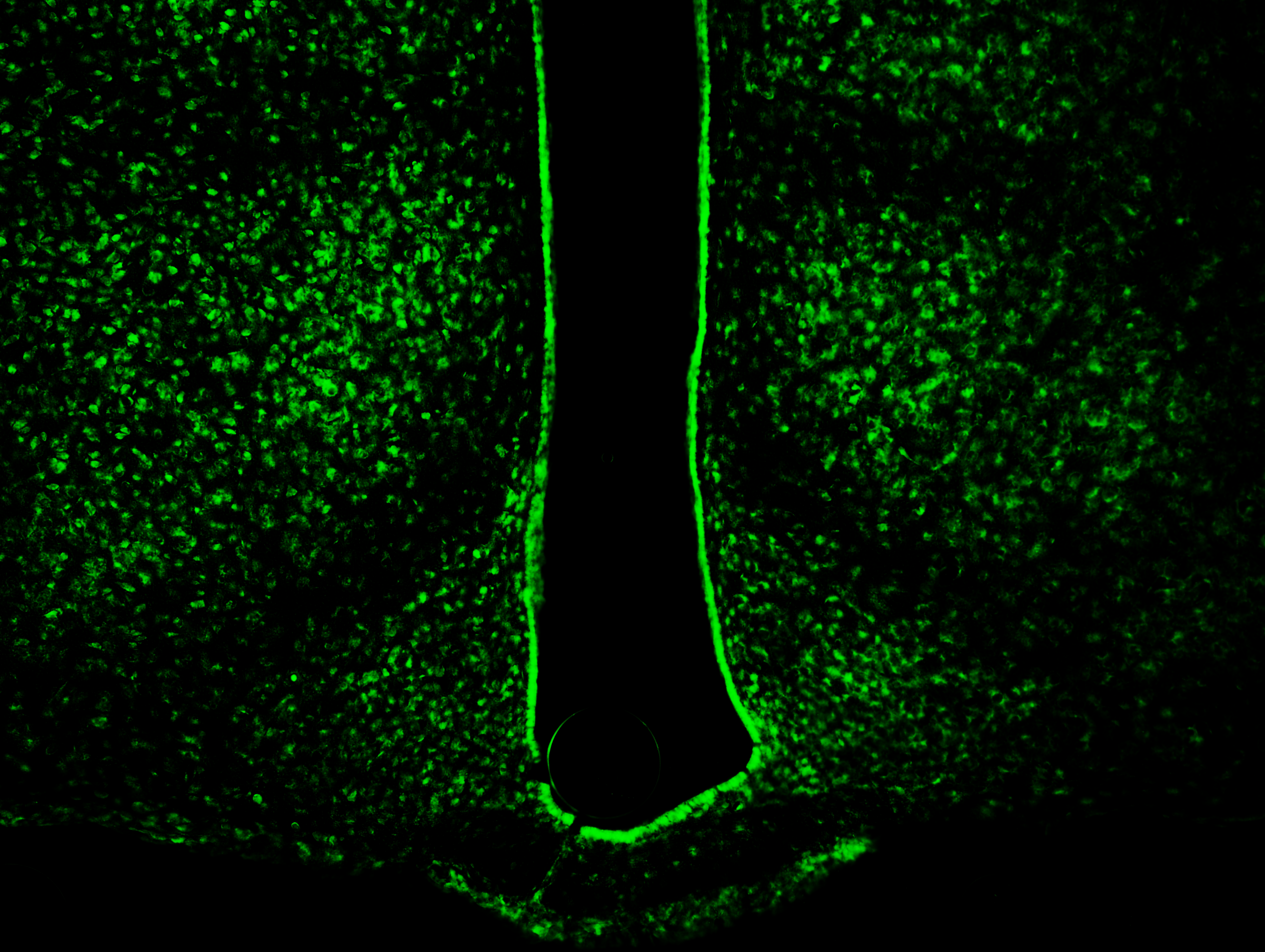

Supplement: Supplementary file 11 — EV and Appendix Figures Source Data [file 44319_2025_398_MOESM11_ESM.zip › Expand View Figures Source Data/Appendix Figures Source Data/Appendix Figure 3/Appendix Figure 3A/NL-ARC VMH.tif]

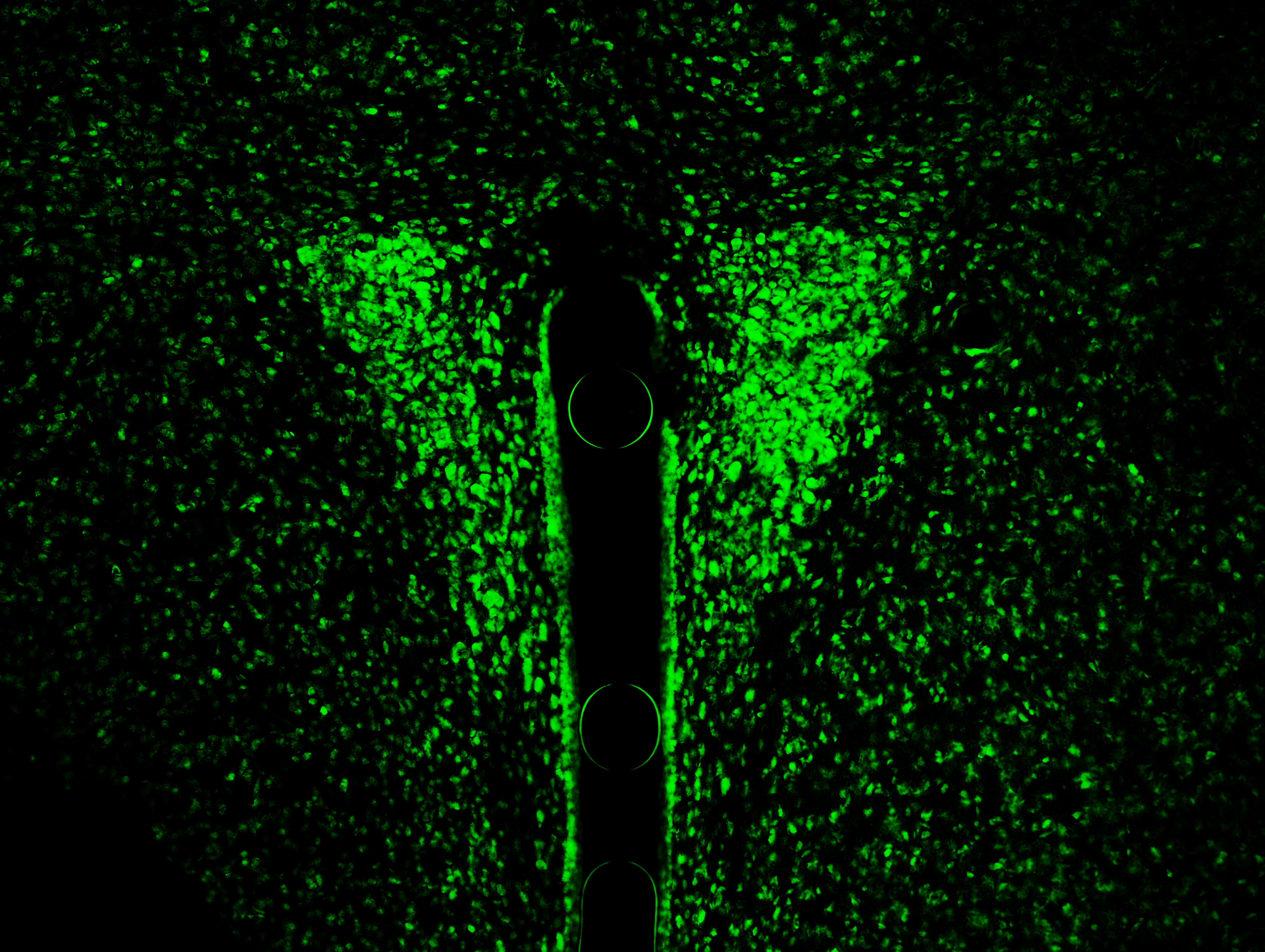

Supplement: Supplementary file 11 — EV and Appendix Figures Source Data [file 44319_2025_398_MOESM11_ESM.zip › Expand View Figures Source Data/Appendix Figures Source Data/Appendix Figure 3/Appendix Figure 3A/NL-PVN.tif]

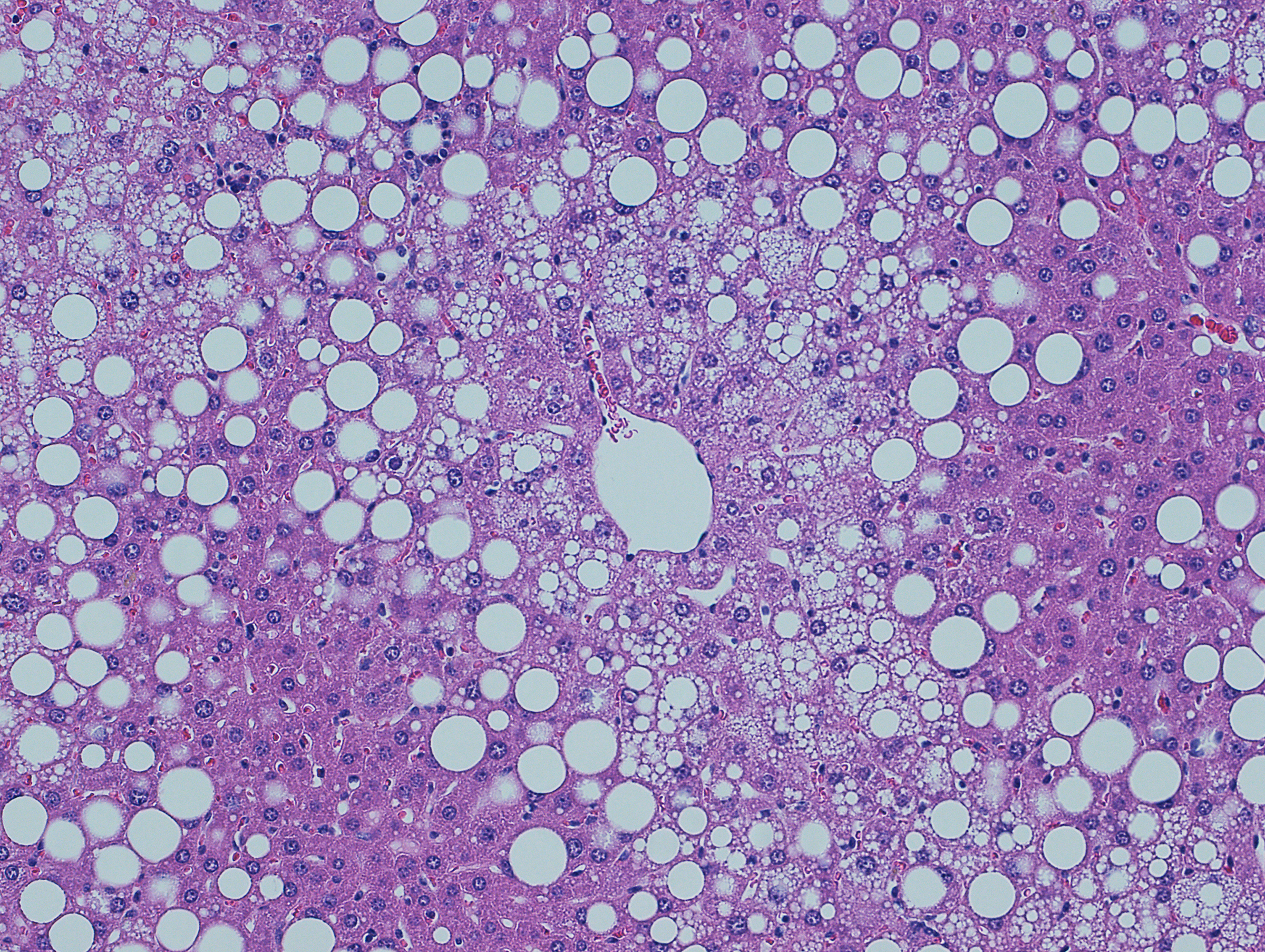

Supplement: Supplementary file 11 — EV and Appendix Figures Source Data [file 44319_2025_398_MOESM11_ESM.zip › Expand View Figures Source Data/Expand View Figures Source Data/Expand View Figure 2/Expand View Figure 2 A/Liver-FF.tif]

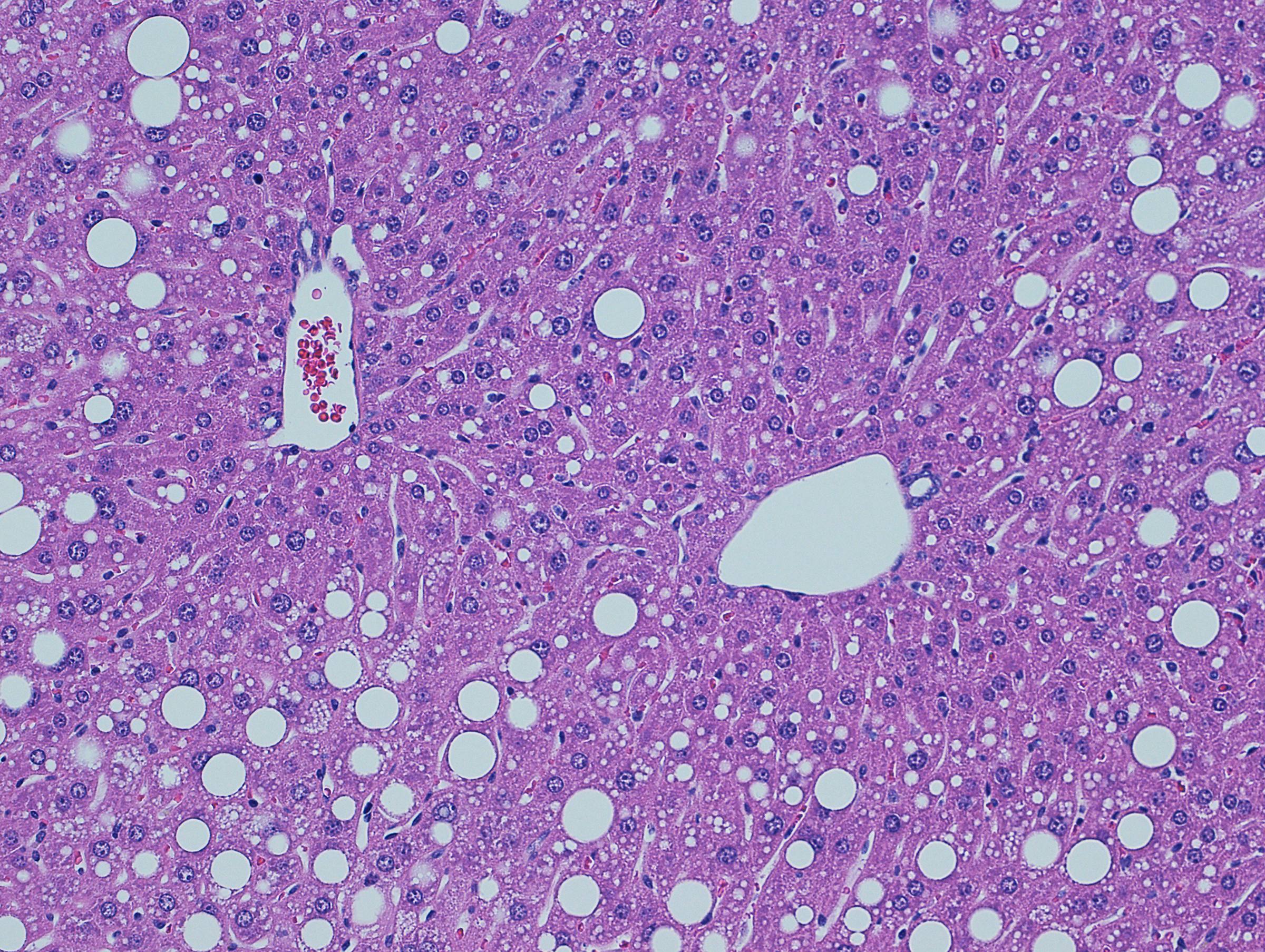

Supplement: Supplementary file 11 — EV and Appendix Figures Source Data [file 44319_2025_398_MOESM11_ESM.zip › Expand View Figures Source Data/Expand View Figures Source Data/Expand View Figure 2/Expand View Figure 2 A/Liver-KO.tif]

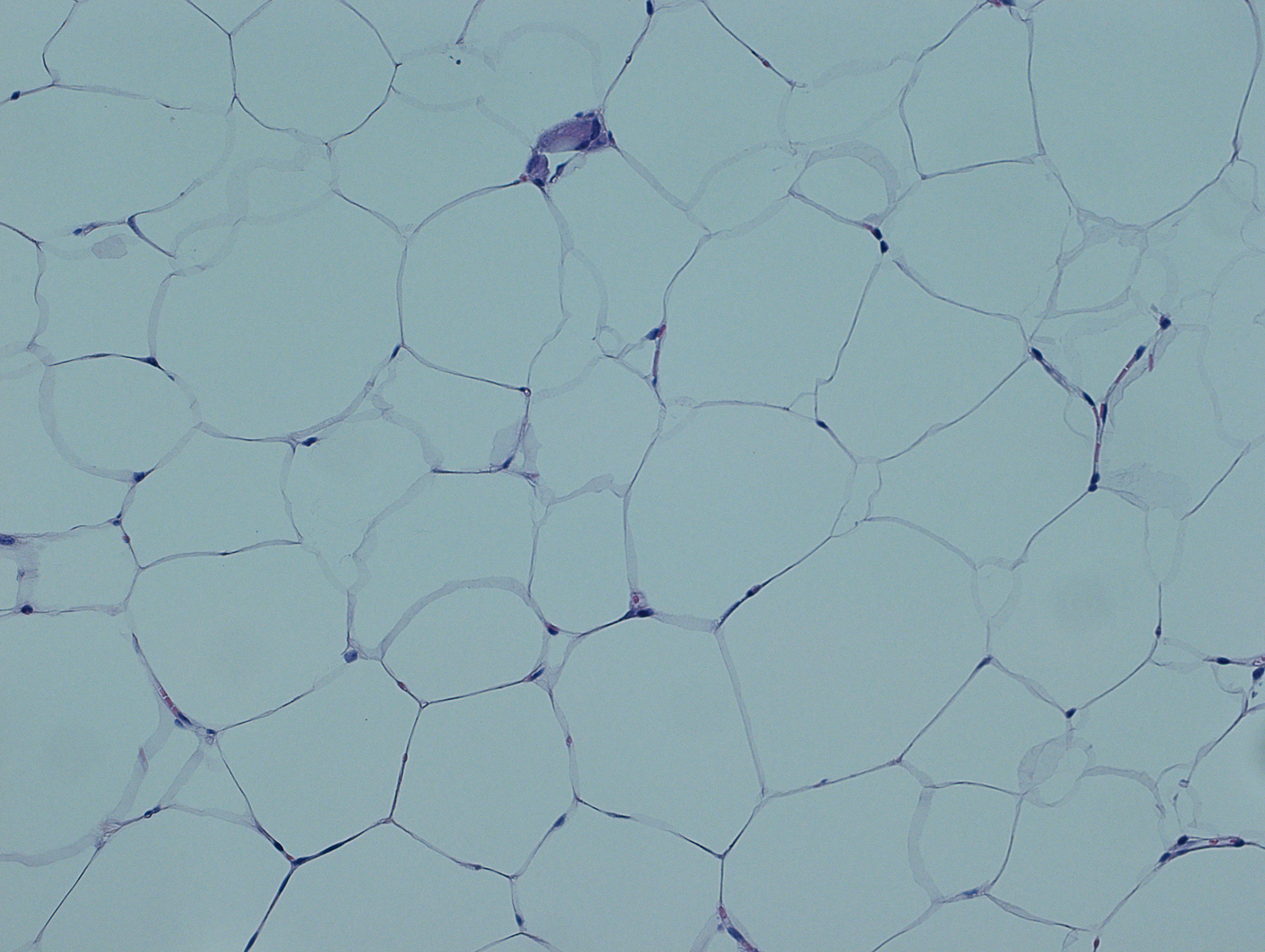

Supplement: Supplementary file 11 — EV and Appendix Figures Source Data [file 44319_2025_398_MOESM11_ESM.zip › Expand View Figures Source Data/Expand View Figures Source Data/Expand View Figure 2/Expand View Figure 2 D/SAT-FF.tif]

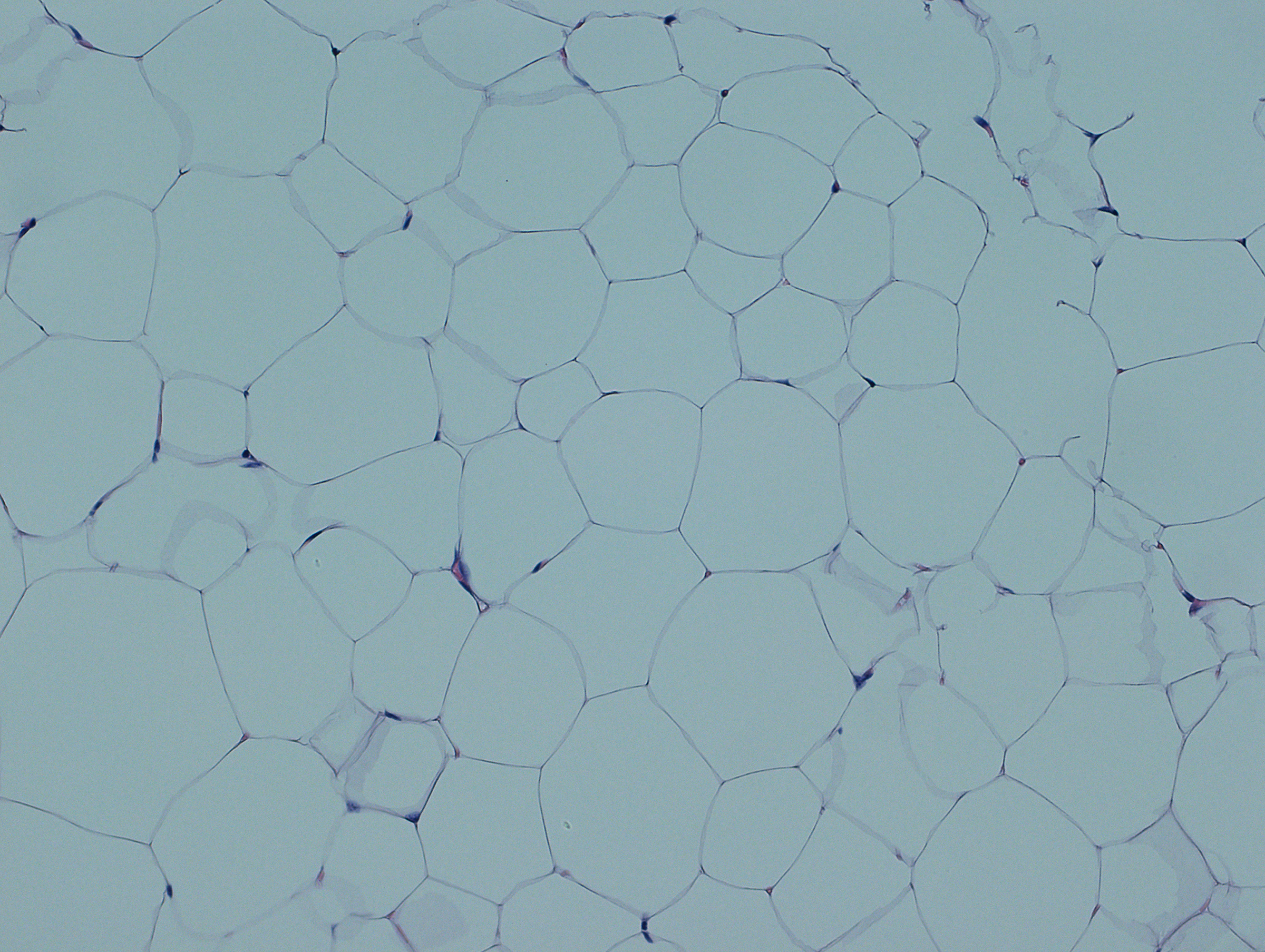

Supplement: Supplementary file 11 — EV and Appendix Figures Source Data [file 44319_2025_398_MOESM11_ESM.zip › Expand View Figures Source Data/Expand View Figures Source Data/Expand View Figure 2/Expand View Figure 2 D/SAT-KO.tif]

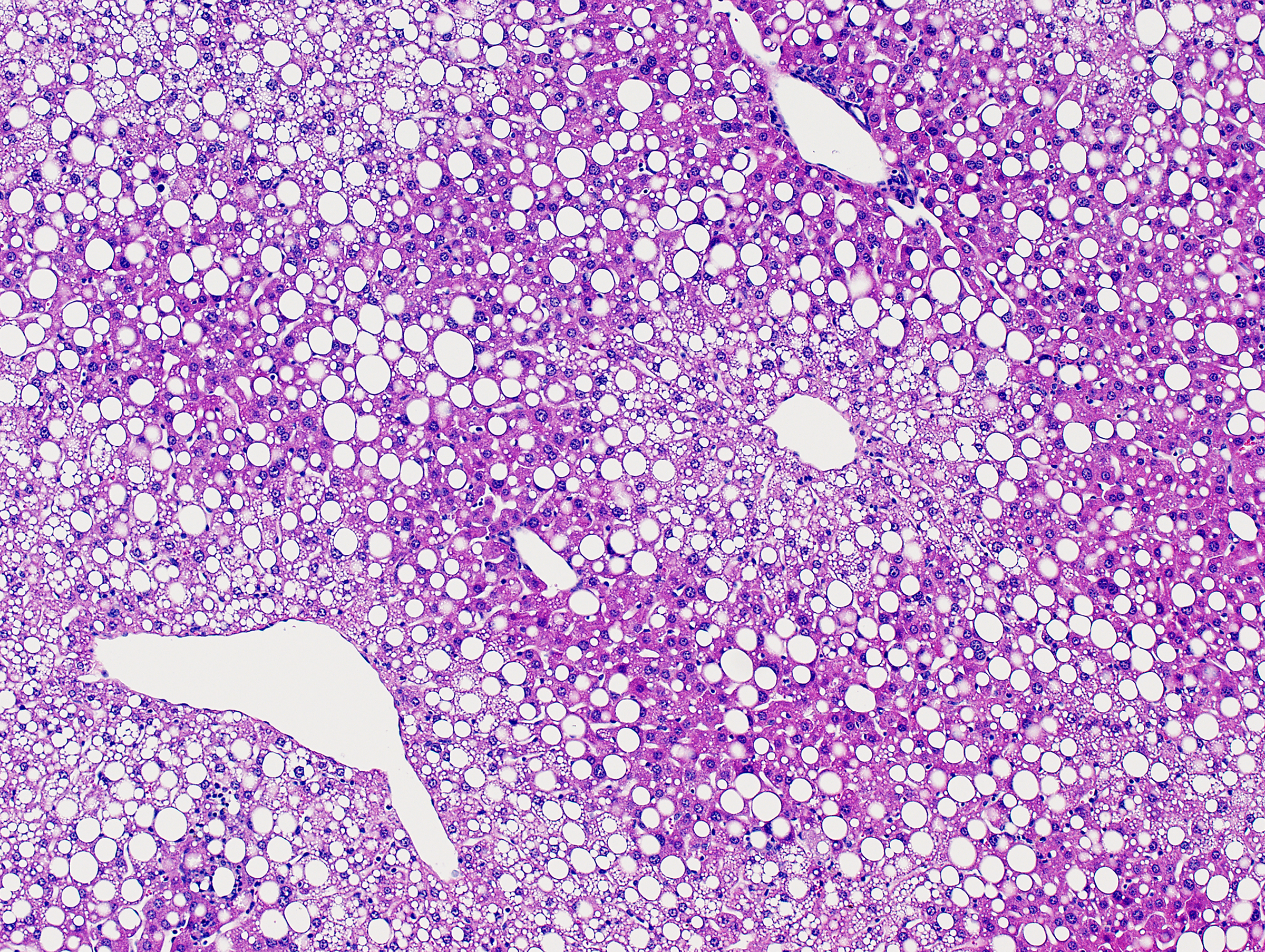

Supplement: Supplementary file 11 — EV and Appendix Figures Source Data [file 44319_2025_398_MOESM11_ESM.zip › Expand View Figures Source Data/Expand View Figures Source Data/Expand View Figure 3/Expand View Figure 3 A/Liver-FF.tif]

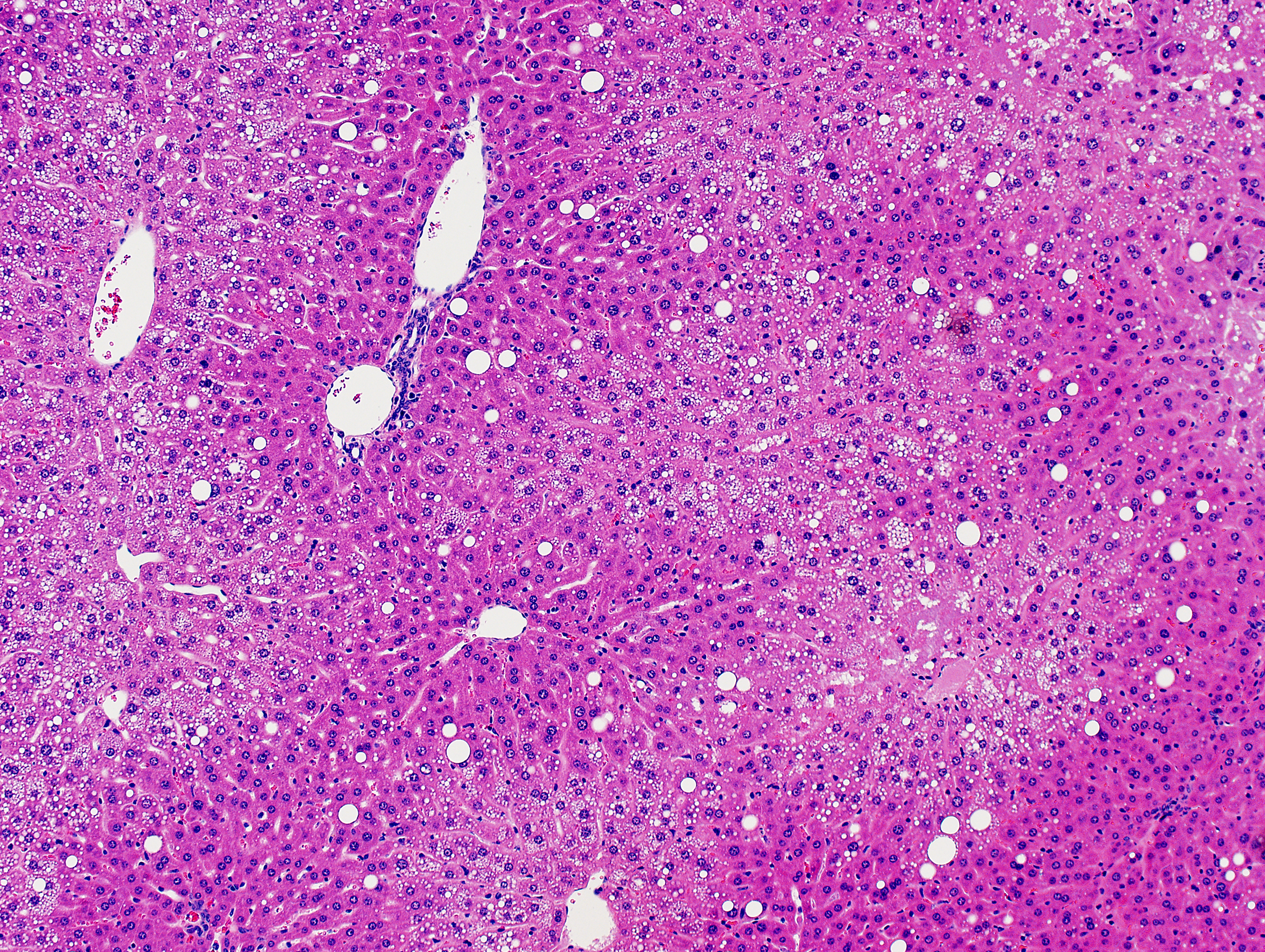

Supplement: Supplementary file 11 — EV and Appendix Figures Source Data [file 44319_2025_398_MOESM11_ESM.zip › Expand View Figures Source Data/Expand View Figures Source Data/Expand View Figure 3/Expand View Figure 3 A/Liver-KO.tif]

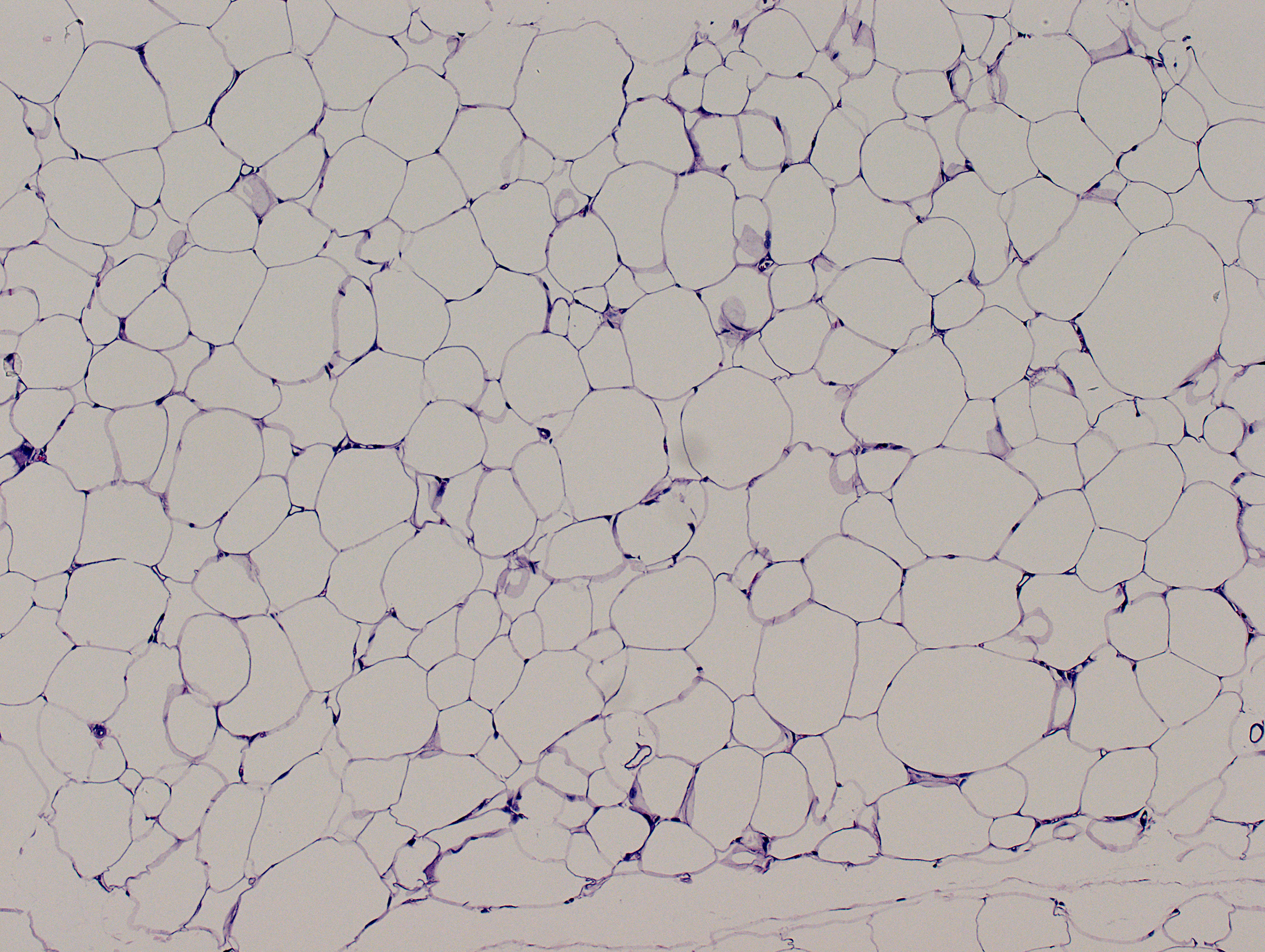

Supplement: Supplementary file 11 — EV and Appendix Figures Source Data [file 44319_2025_398_MOESM11_ESM.zip › Expand View Figures Source Data/Expand View Figures Source Data/Expand View Figure 3/Expand View Figure 3 E/SAT-FF.tif]

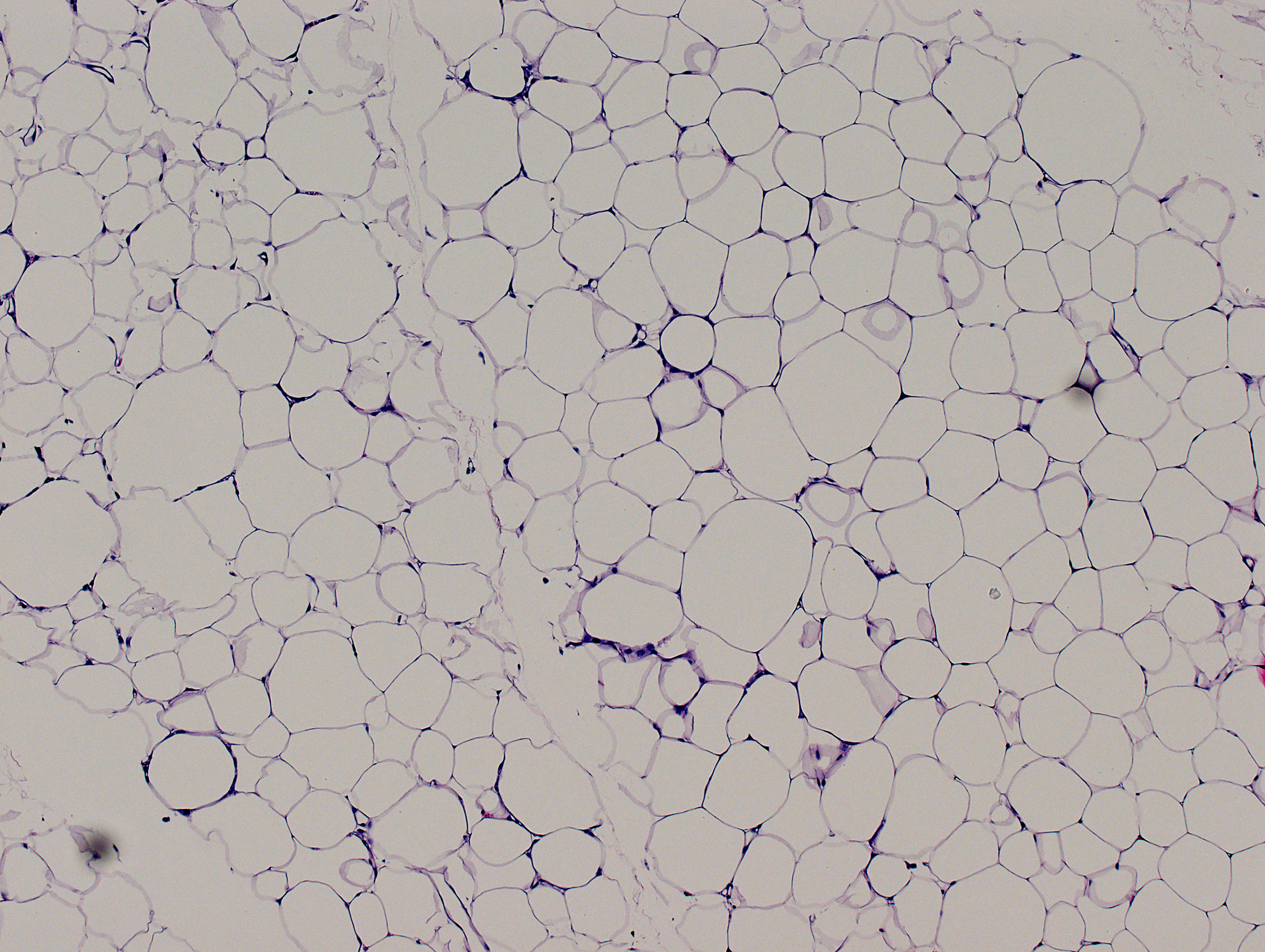

Supplement: Supplementary file 11 — EV and Appendix Figures Source Data [file 44319_2025_398_MOESM11_ESM.zip › Expand View Figures Source Data/Expand View Figures Source Data/Expand View Figure 3/Expand View Figure 3 E/SAT-KO.tif]

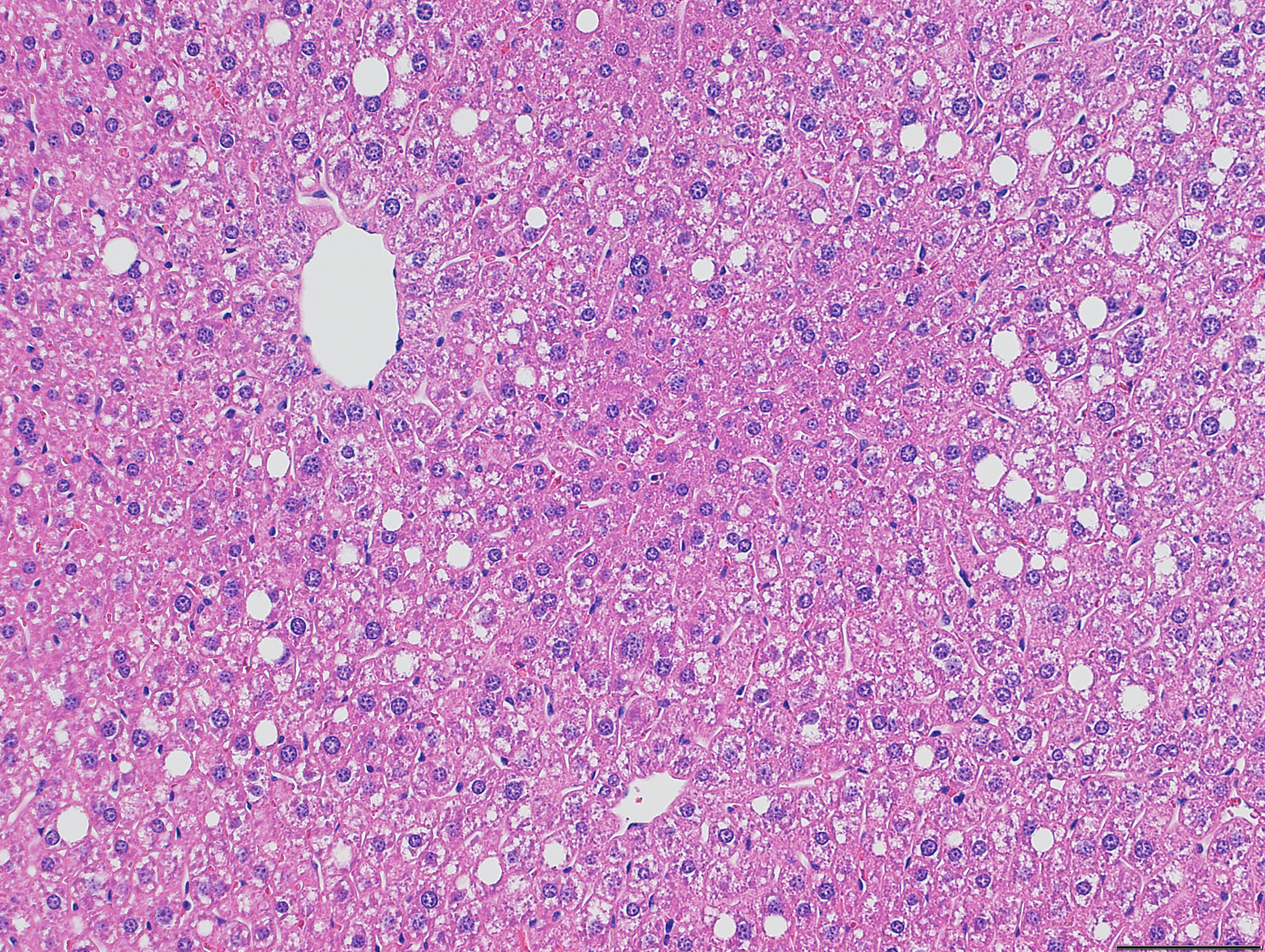

Supplement: Supplementary file 11 — EV and Appendix Figures Source Data [file 44319_2025_398_MOESM11_ESM.zip › Expand View Figures Source Data/Expand View Figures Source Data/Expand View Figure 4/Expand View Figure 4 A/Liver-FF-Sham.tif]

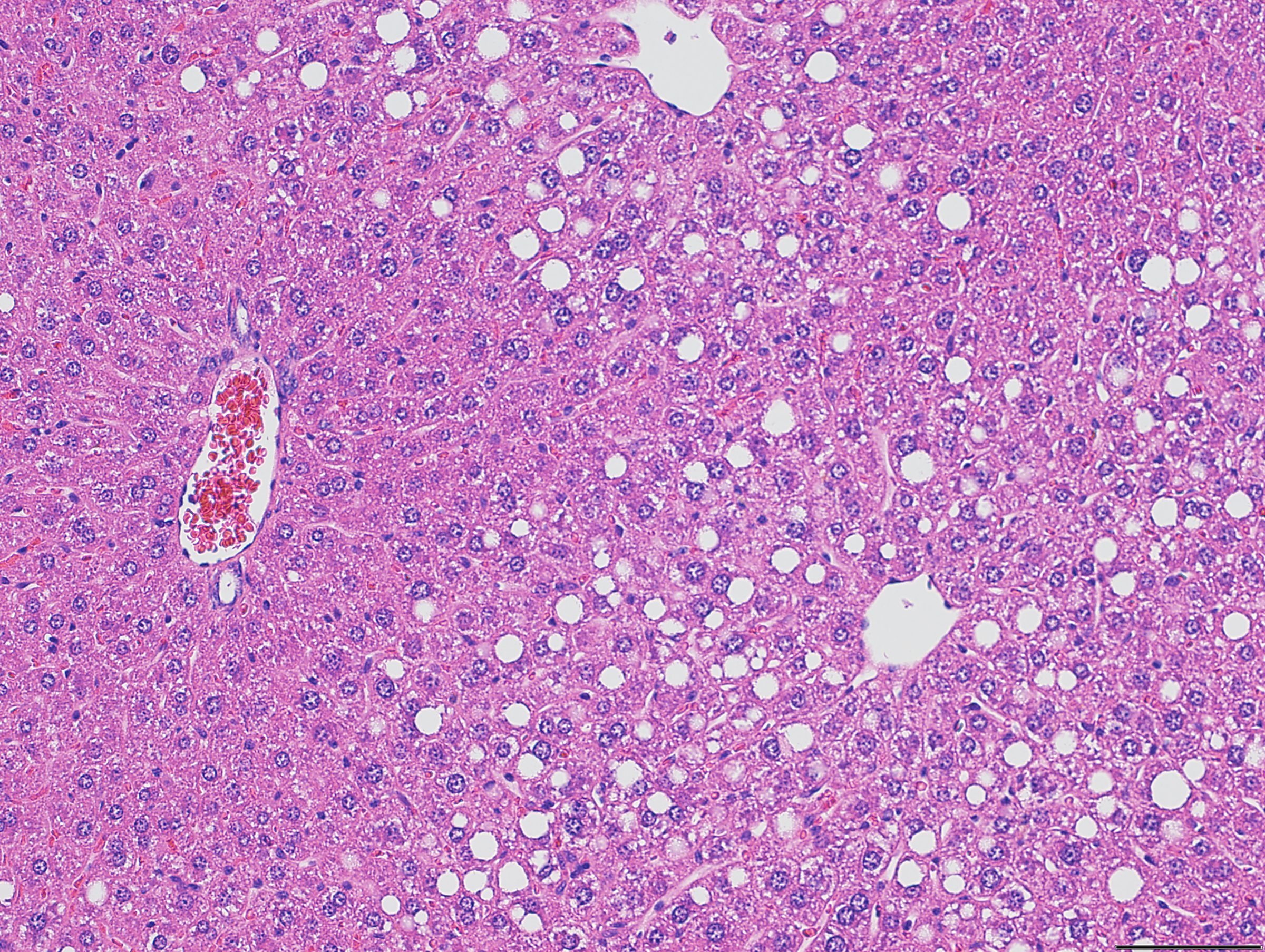

Supplement: Supplementary file 11 — EV and Appendix Figures Source Data [file 44319_2025_398_MOESM11_ESM.zip › Expand View Figures Source Data/Expand View Figures Source Data/Expand View Figure 4/Expand View Figure 4 A/Liver-FF-Surgery.tif]

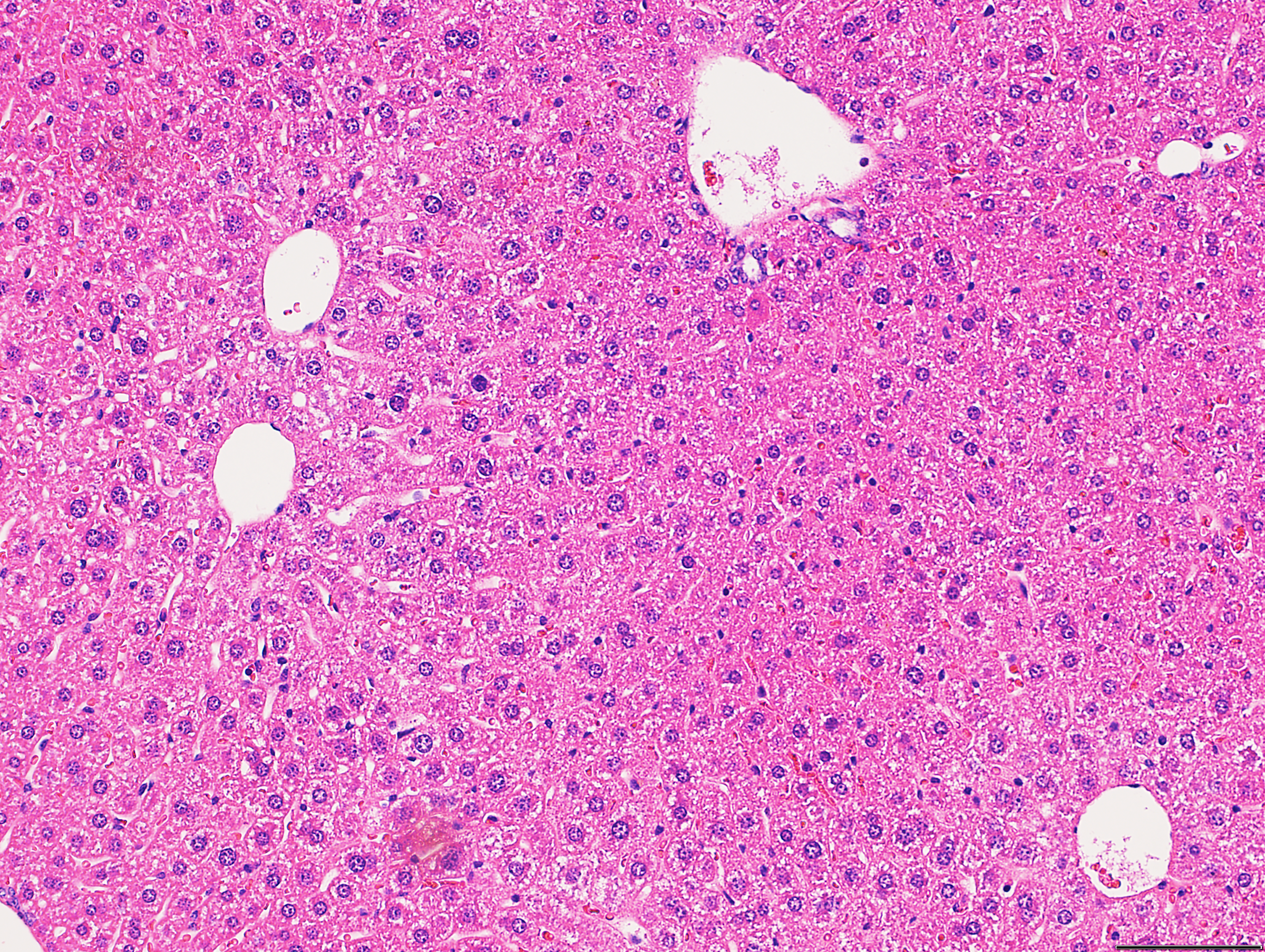

Supplement: Supplementary file 11 — EV and Appendix Figures Source Data [file 44319_2025_398_MOESM11_ESM.zip › Expand View Figures Source Data/Expand View Figures Source Data/Expand View Figure 4/Expand View Figure 4 A/Liver-KO-Sham.tif]

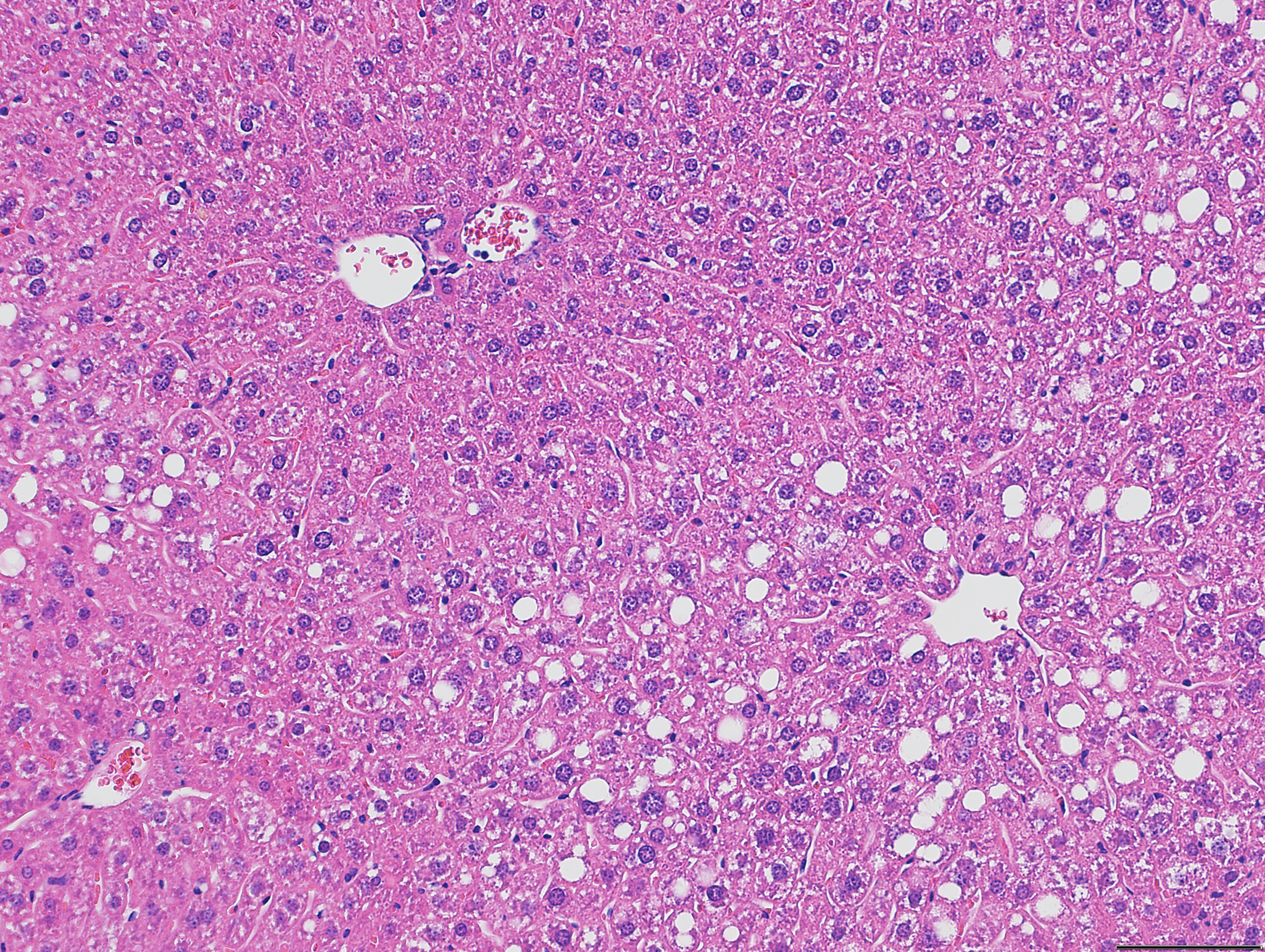

Supplement: Supplementary file 11 — EV and Appendix Figures Source Data [file 44319_2025_398_MOESM11_ESM.zip › Expand View Figures Source Data/Expand View Figures Source Data/Expand View Figure 4/Expand View Figure 4 A/Liver-KO-Surgery.tif]

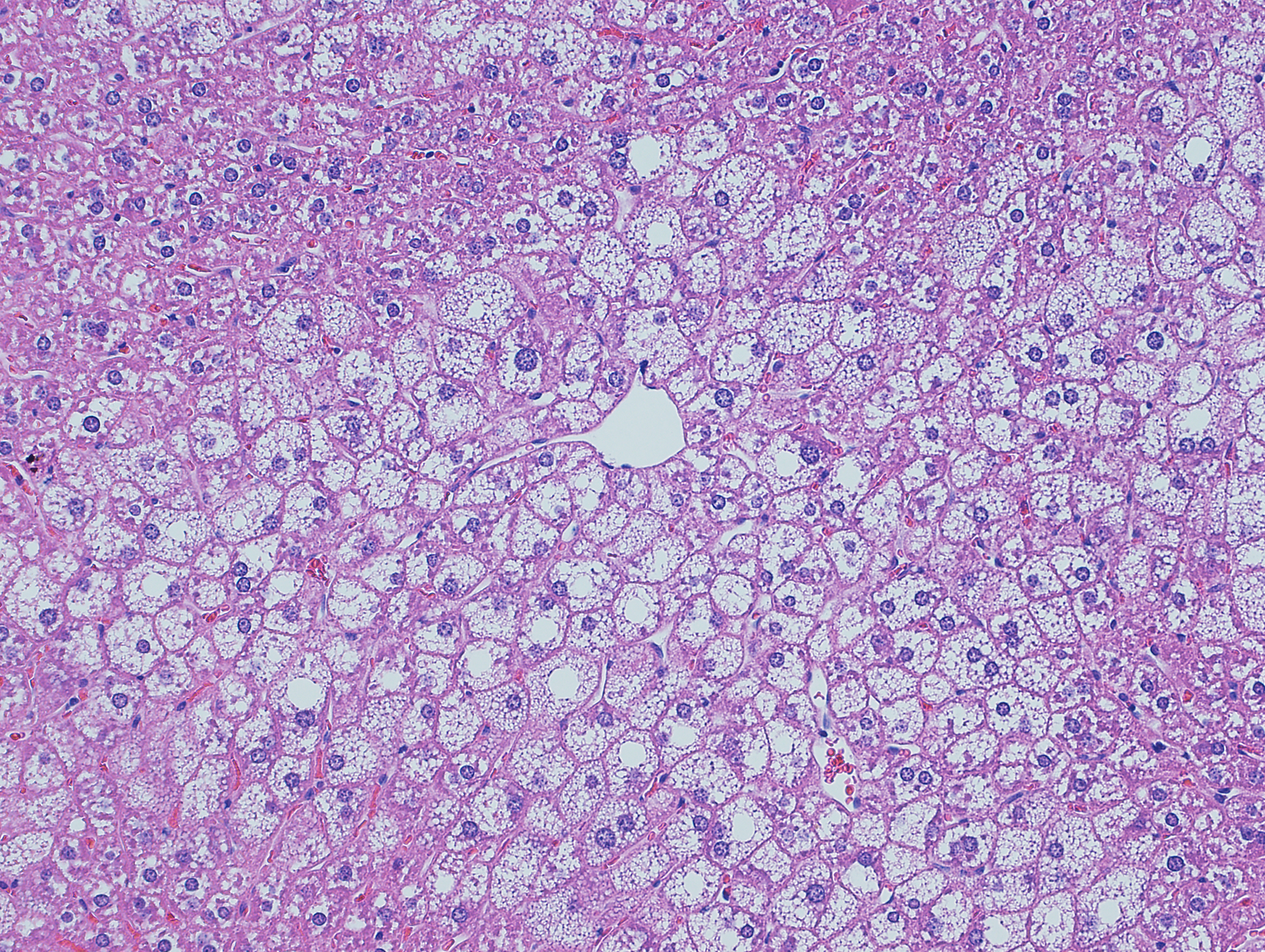

Supplement: Supplementary file 11 — EV and Appendix Figures Source Data [file 44319_2025_398_MOESM11_ESM.zip › Expand View Figures Source Data/Expand View Figures Source Data/Expand View Figure 4/Expand View Figure 4 E/FF-Sham.tif]

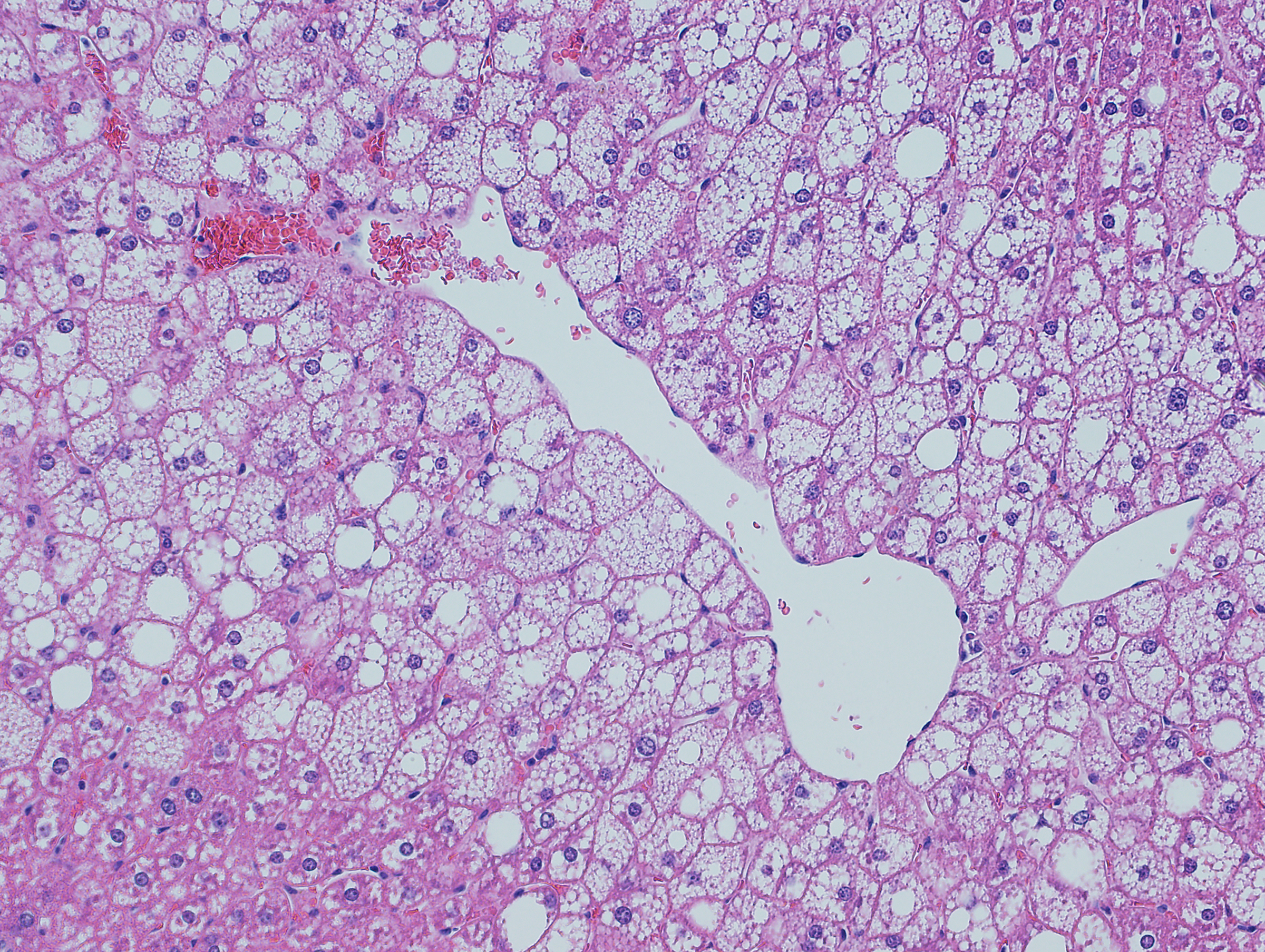

Supplement: Supplementary file 11 — EV and Appendix Figures Source Data [file 44319_2025_398_MOESM11_ESM.zip › Expand View Figures Source Data/Expand View Figures Source Data/Expand View Figure 4/Expand View Figure 4 E/FF-Surgery.tif]

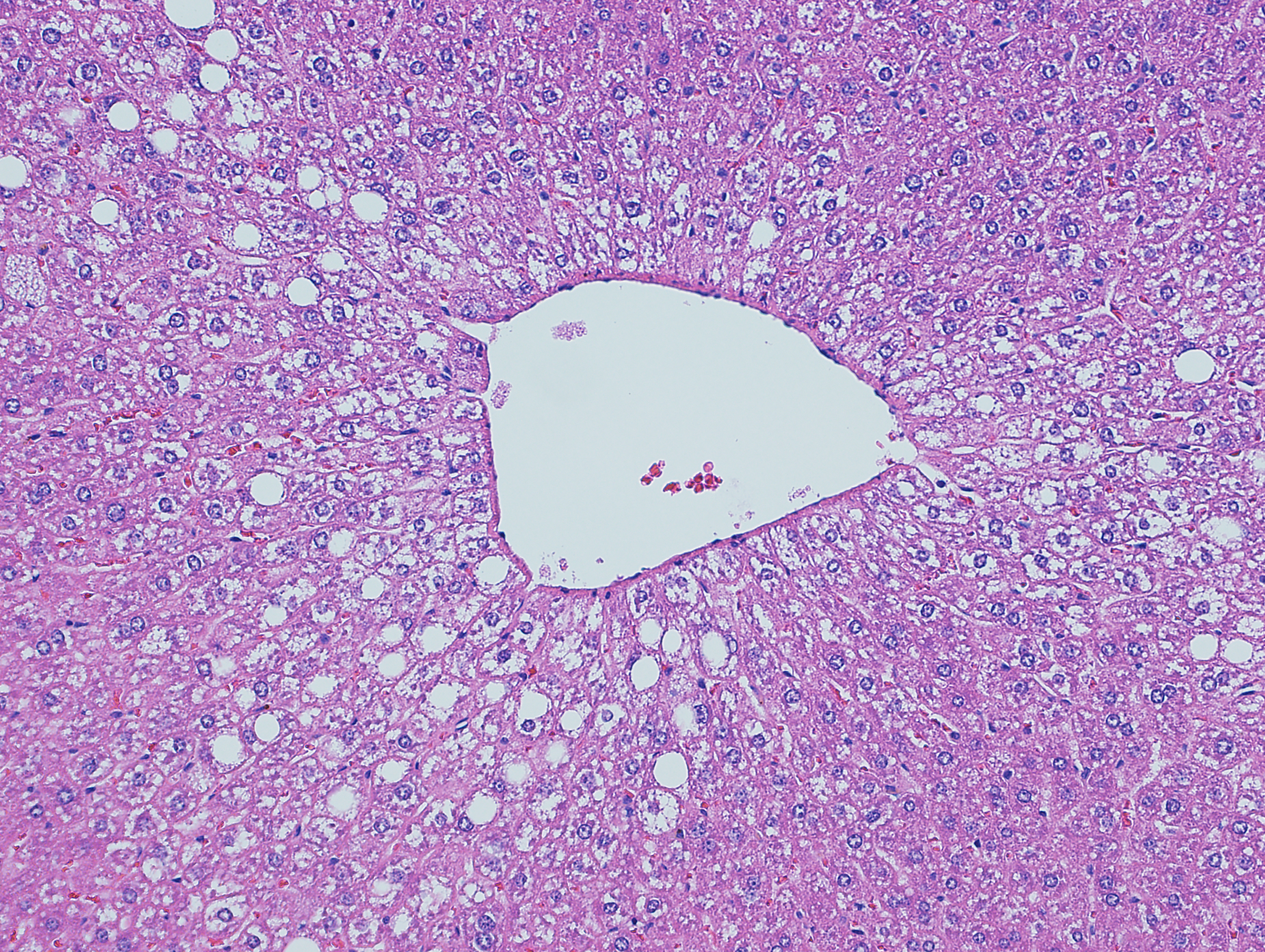

Supplement: Supplementary file 11 — EV and Appendix Figures Source Data [file 44319_2025_398_MOESM11_ESM.zip › Expand View Figures Source Data/Expand View Figures Source Data/Expand View Figure 4/Expand View Figure 4 E/KO-Sham.tif]

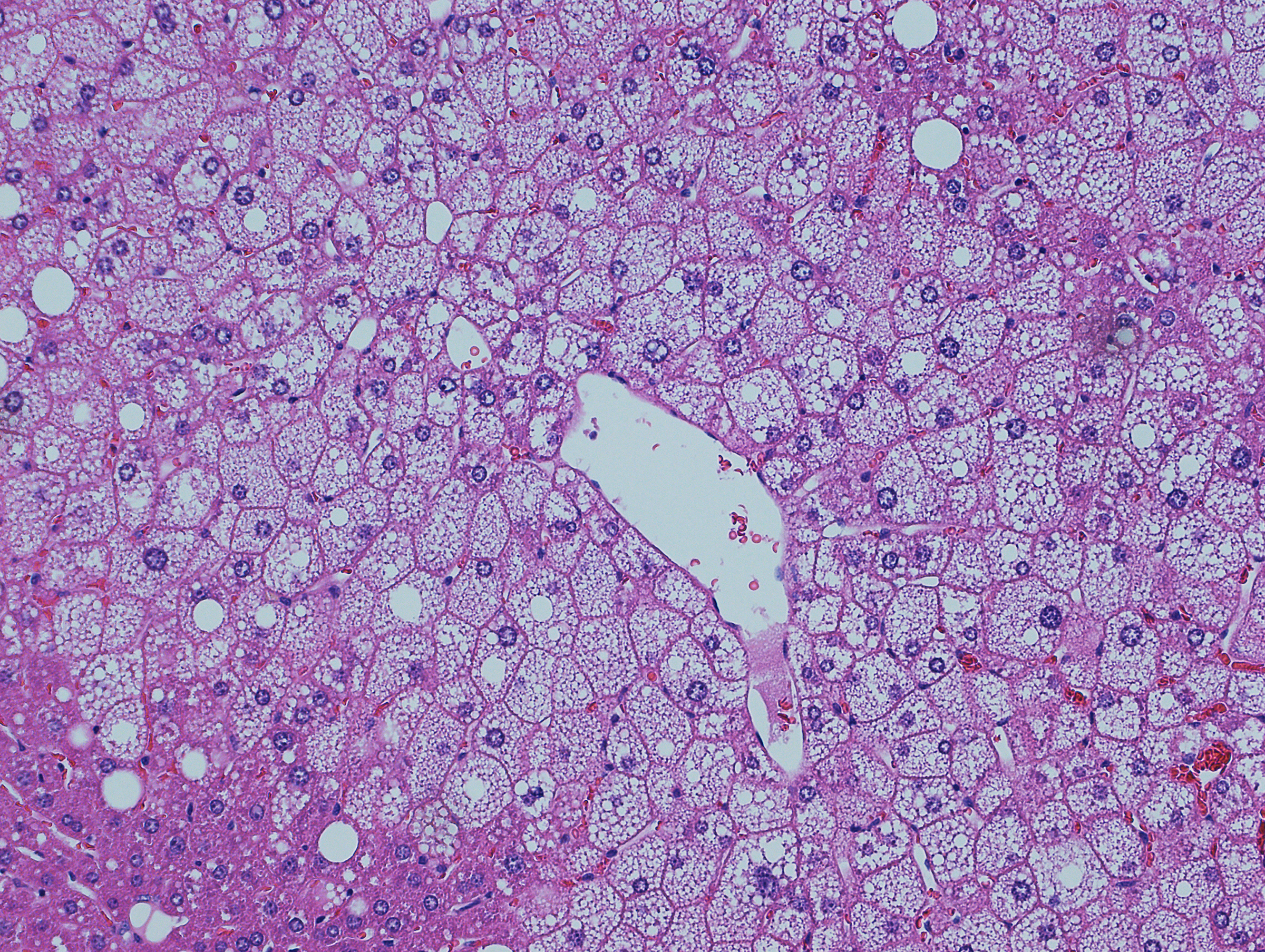

Supplement: Supplementary file 11 — EV and Appendix Figures Source Data [file 44319_2025_398_MOESM11_ESM.zip › Expand View Figures Source Data/Expand View Figures Source Data/Expand View Figure 4/Expand View Figure 4 E/KO-Surgery.tif]

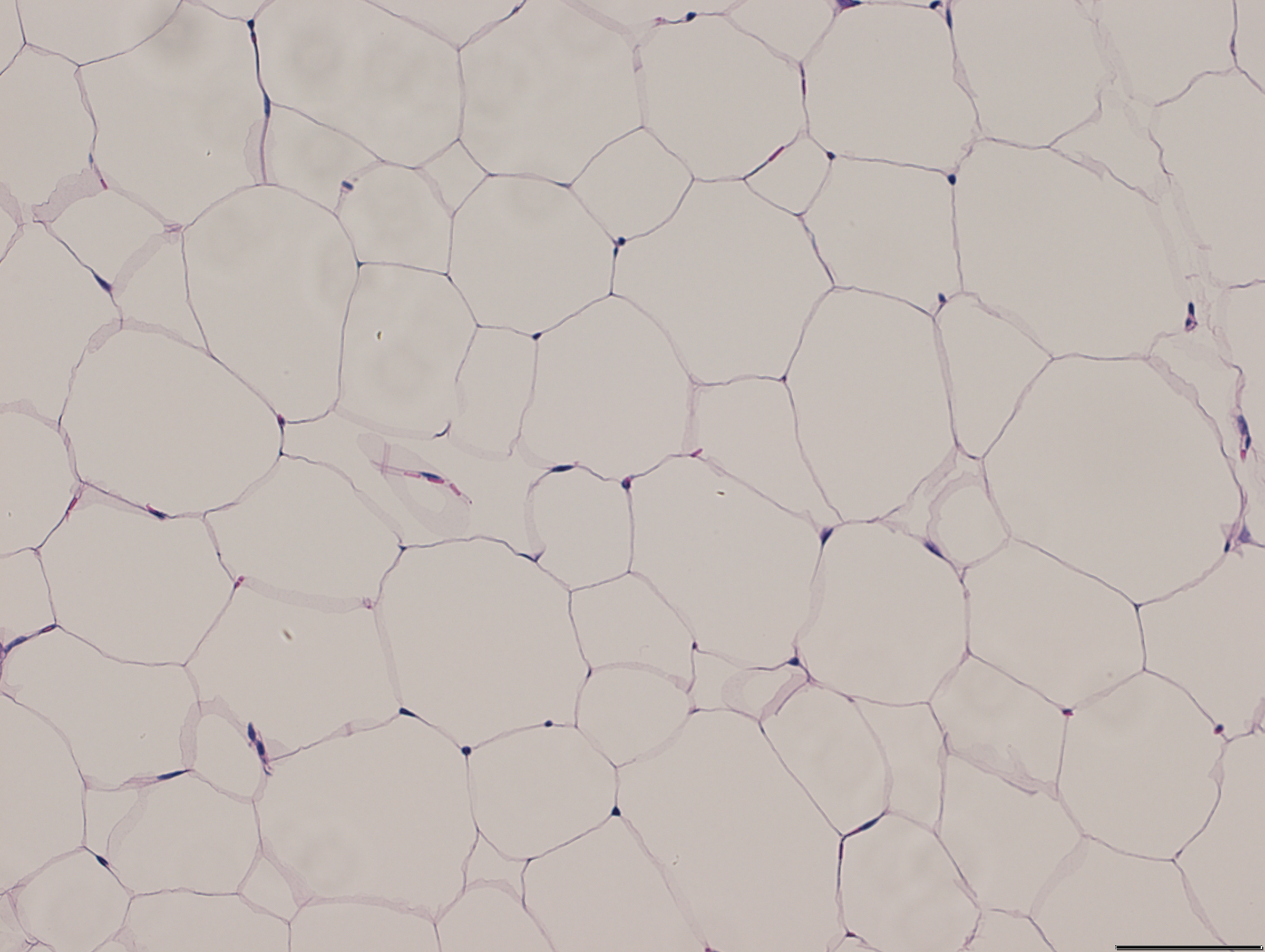

Supplement: Supplementary file 11 — EV and Appendix Figures Source Data [file 44319_2025_398_MOESM11_ESM.zip › Expand View Figures Source Data/Expand View Figures Source Data/Expand View Figure 4/Expand View Figure 4 I/SAT-FF-Sham.tif]

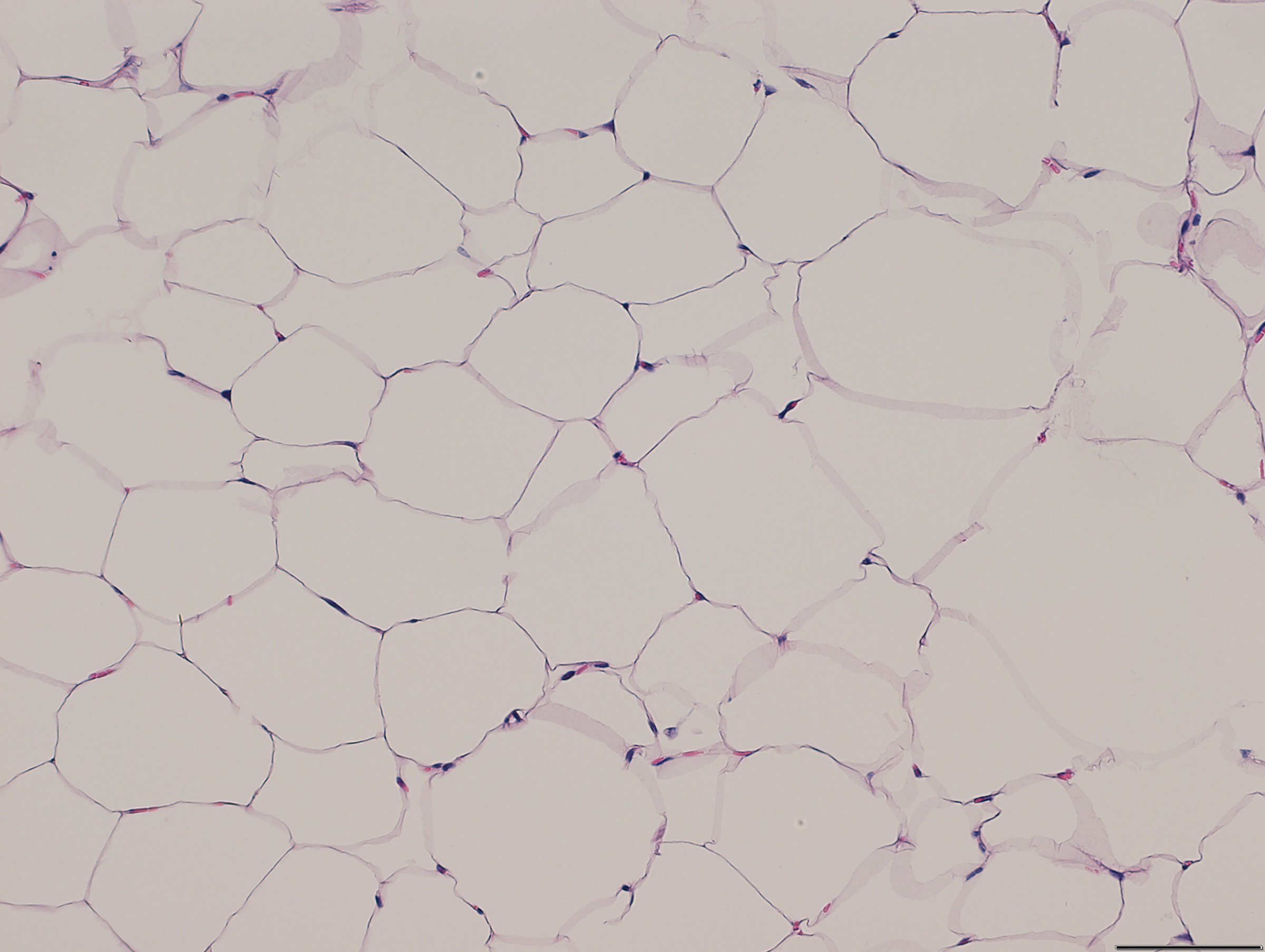

Supplement: Supplementary file 11 — EV and Appendix Figures Source Data [file 44319_2025_398_MOESM11_ESM.zip › Expand View Figures Source Data/Expand View Figures Source Data/Expand View Figure 4/Expand View Figure 4 I/SAT-FF-Surgery.tif]

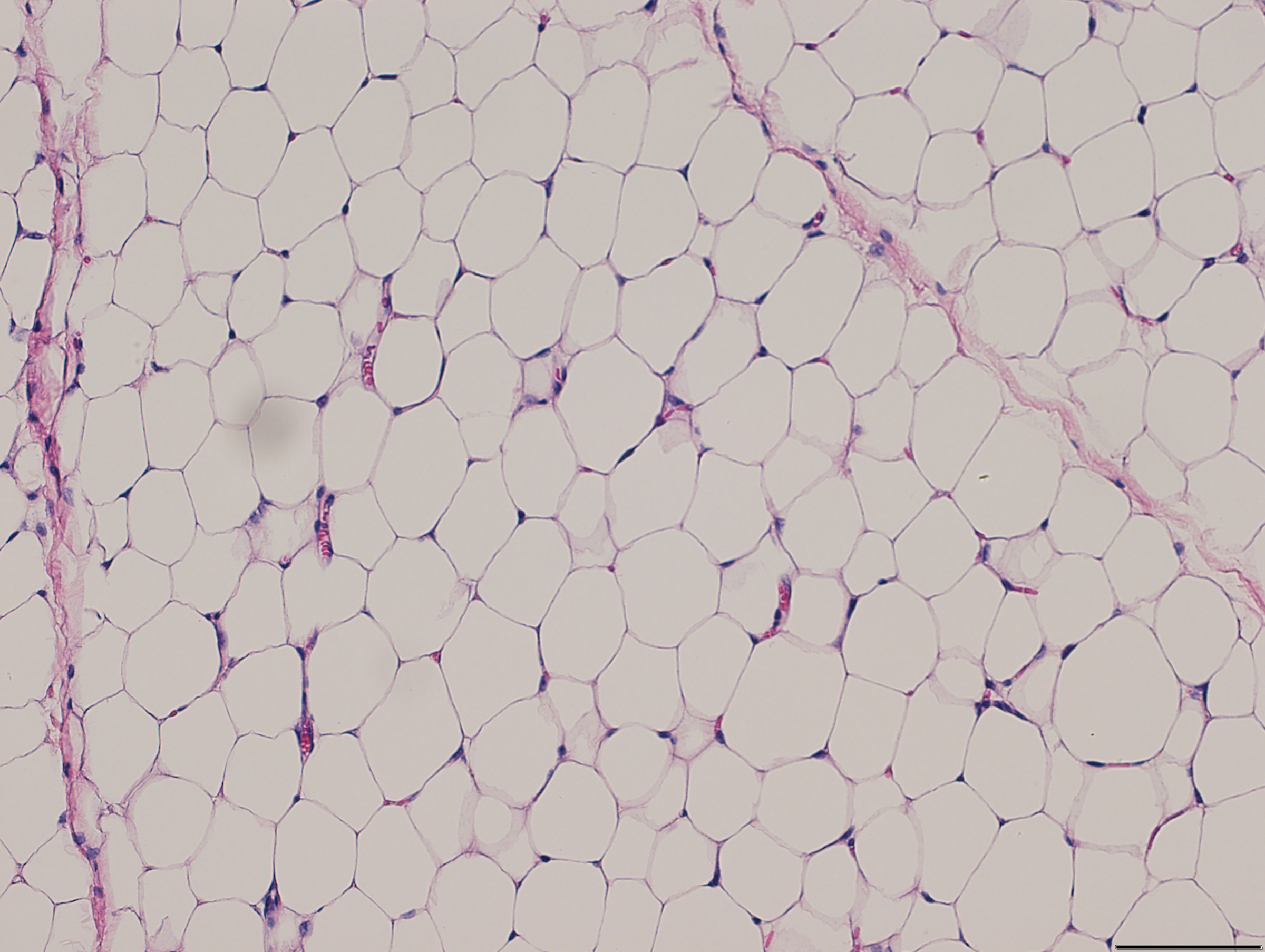

Supplement: Supplementary file 11 — EV and Appendix Figures Source Data [file 44319_2025_398_MOESM11_ESM.zip › Expand View Figures Source Data/Expand View Figures Source Data/Expand View Figure 4/Expand View Figure 4 I/SAT-KO-Sham.tif]

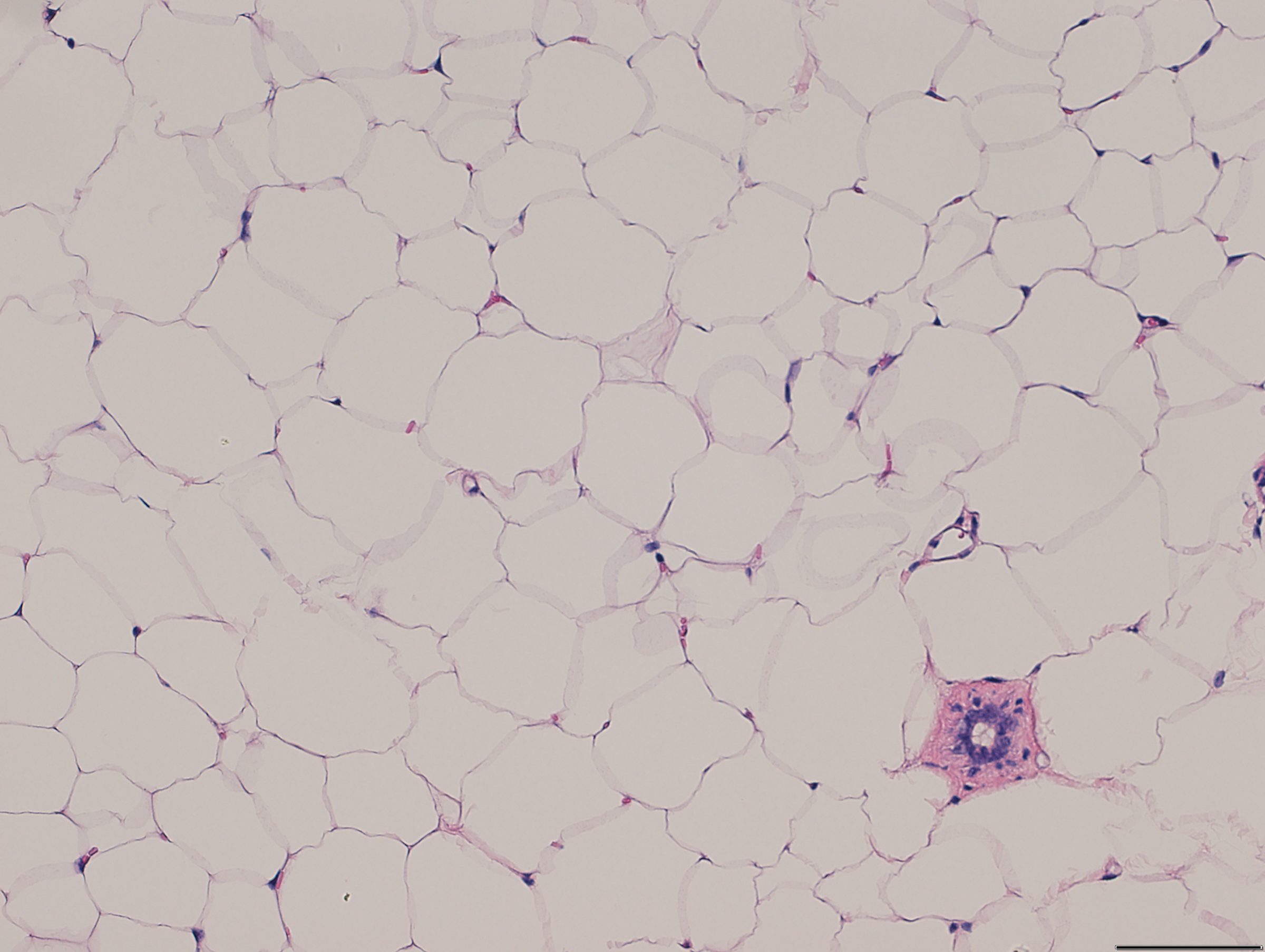

Supplement: Supplementary file 11 — EV and Appendix Figures Source Data [file 44319_2025_398_MOESM11_ESM.zip › Expand View Figures Source Data/Expand View Figures Source Data/Expand View Figure 4/Expand View Figure 4 I/SAT-KO-Surgery.tif]

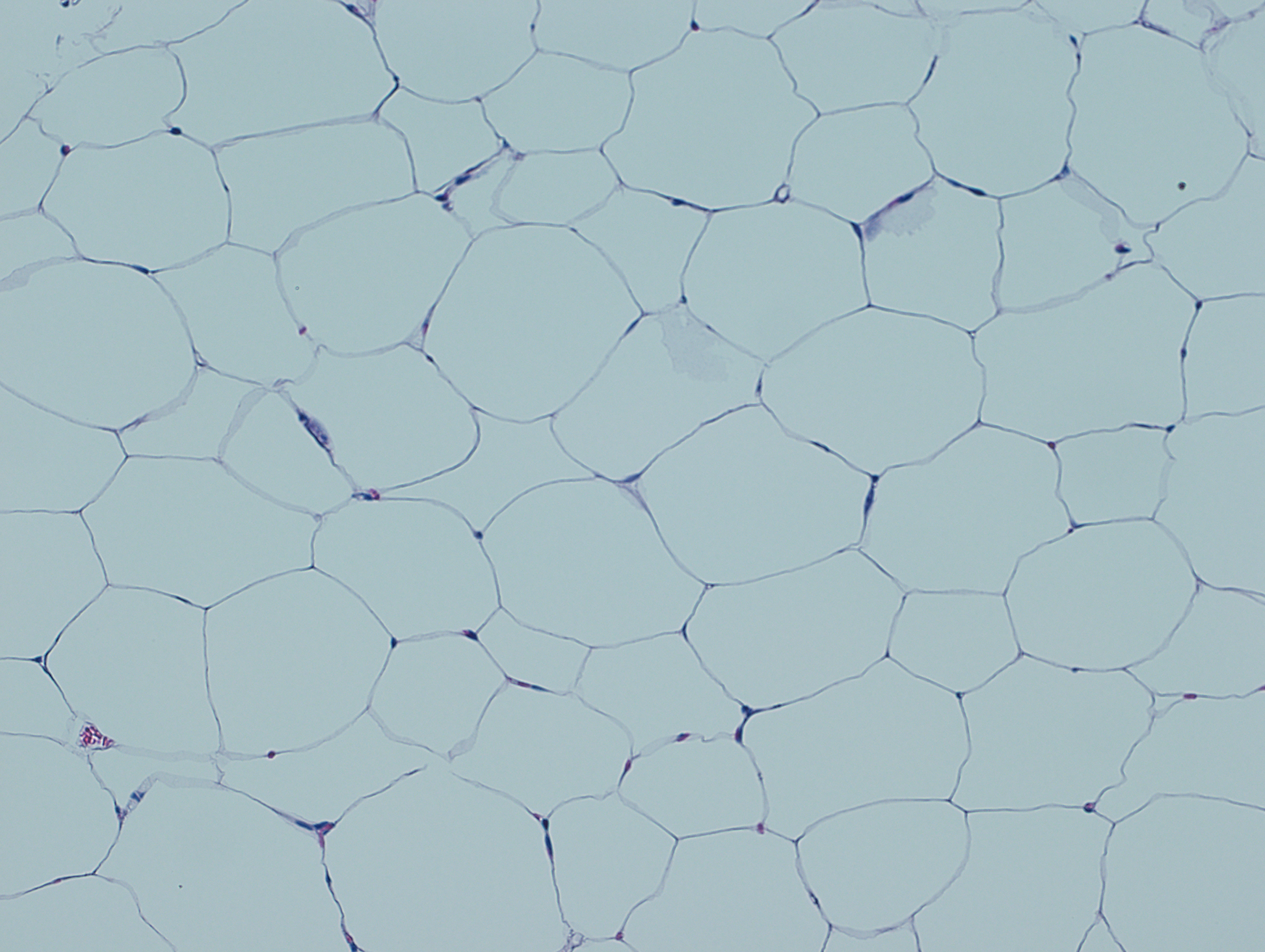

Supplement: Supplementary file 11 — EV and Appendix Figures Source Data [file 44319_2025_398_MOESM11_ESM.zip › Expand View Figures Source Data/Expand View Figures Source Data/Expand View Figure 4/Expand View Figure 4 M/FF-Sham.tif]

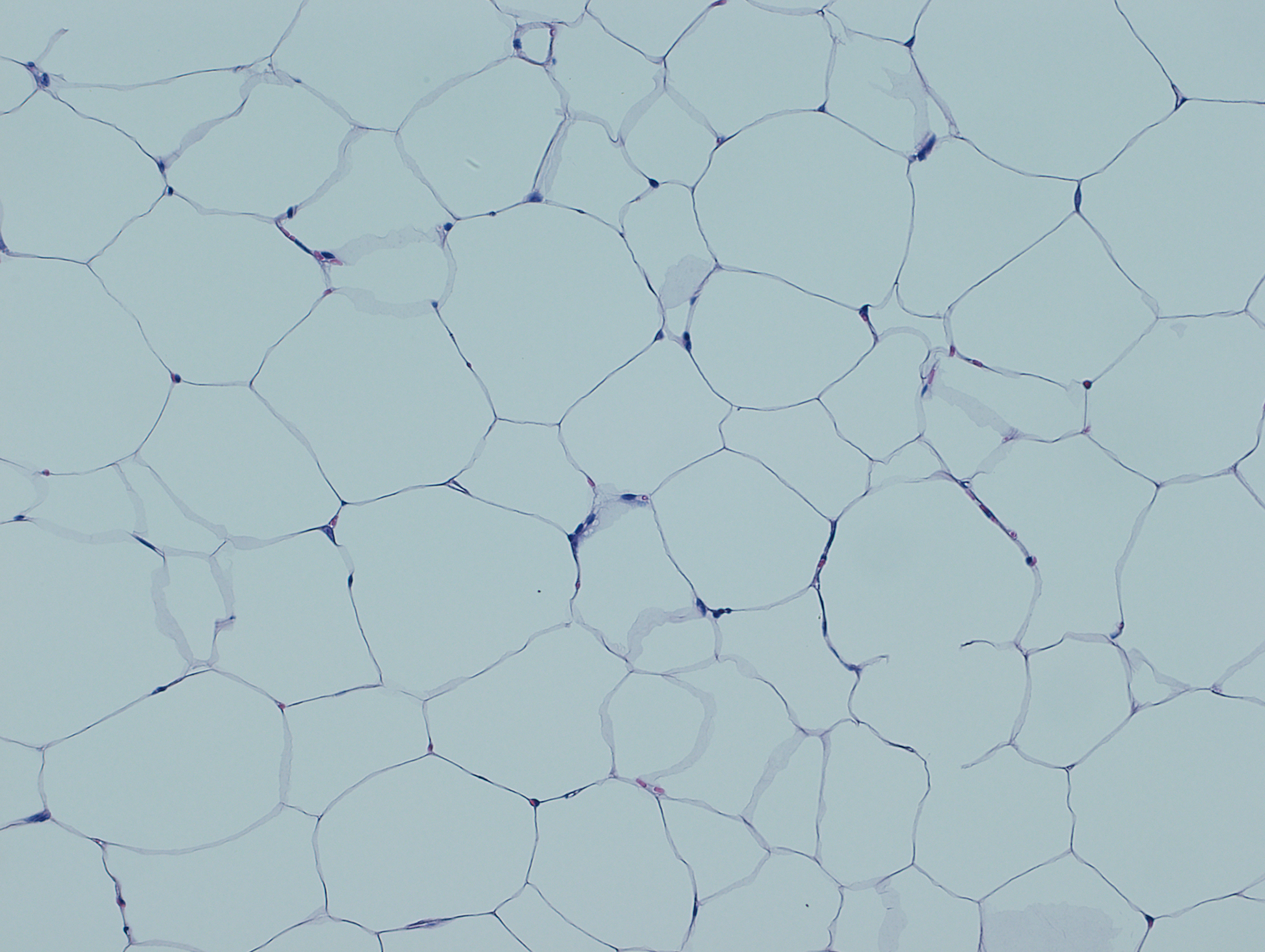

Supplement: Supplementary file 11 — EV and Appendix Figures Source Data [file 44319_2025_398_MOESM11_ESM.zip › Expand View Figures Source Data/Expand View Figures Source Data/Expand View Figure 4/Expand View Figure 4 M/FF-Surgery.tif]

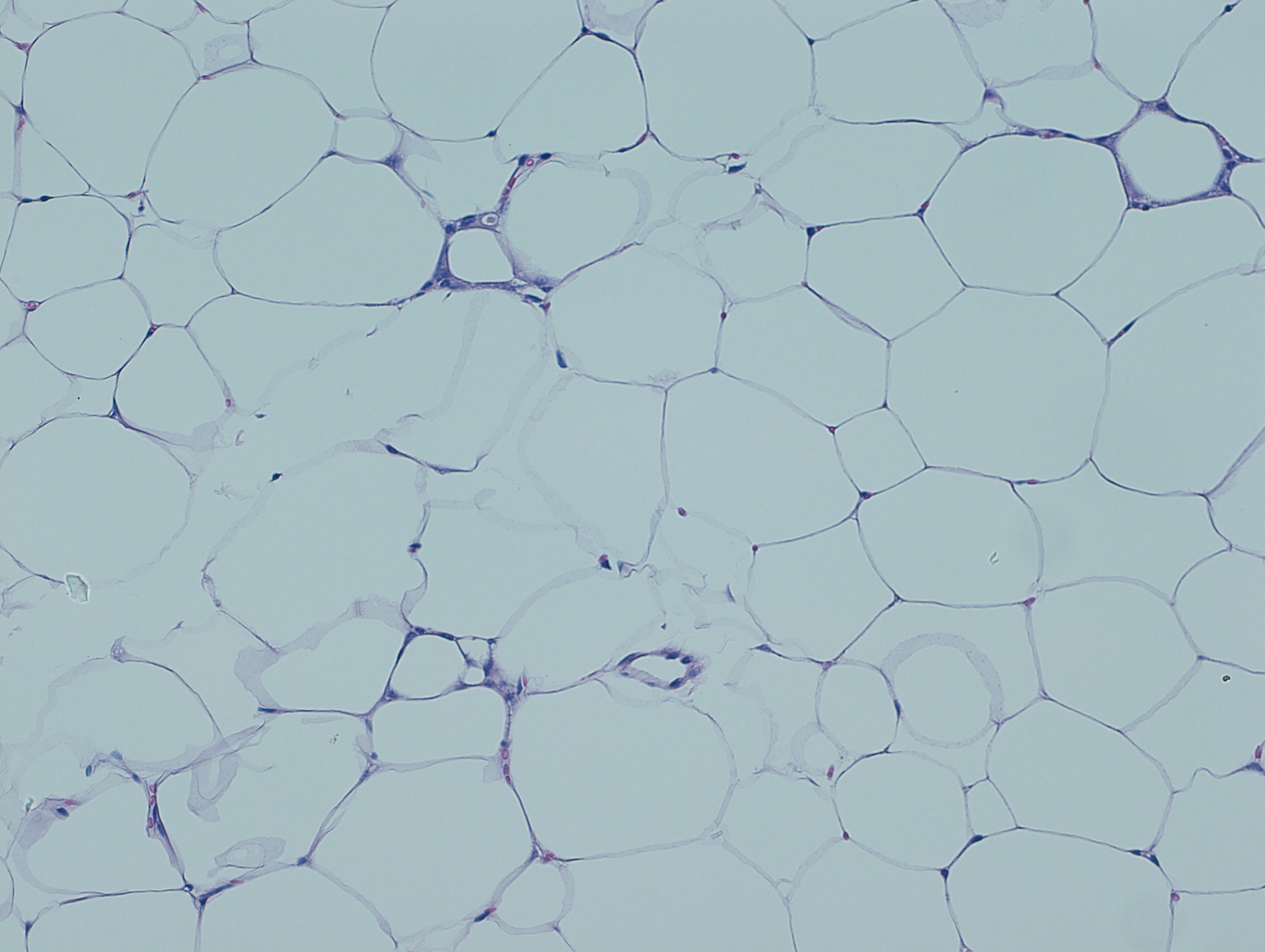

Supplement: Supplementary file 11 — EV and Appendix Figures Source Data [file 44319_2025_398_MOESM11_ESM.zip › Expand View Figures Source Data/Expand View Figures Source Data/Expand View Figure 4/Expand View Figure 4 M/KO-Sham.tif]

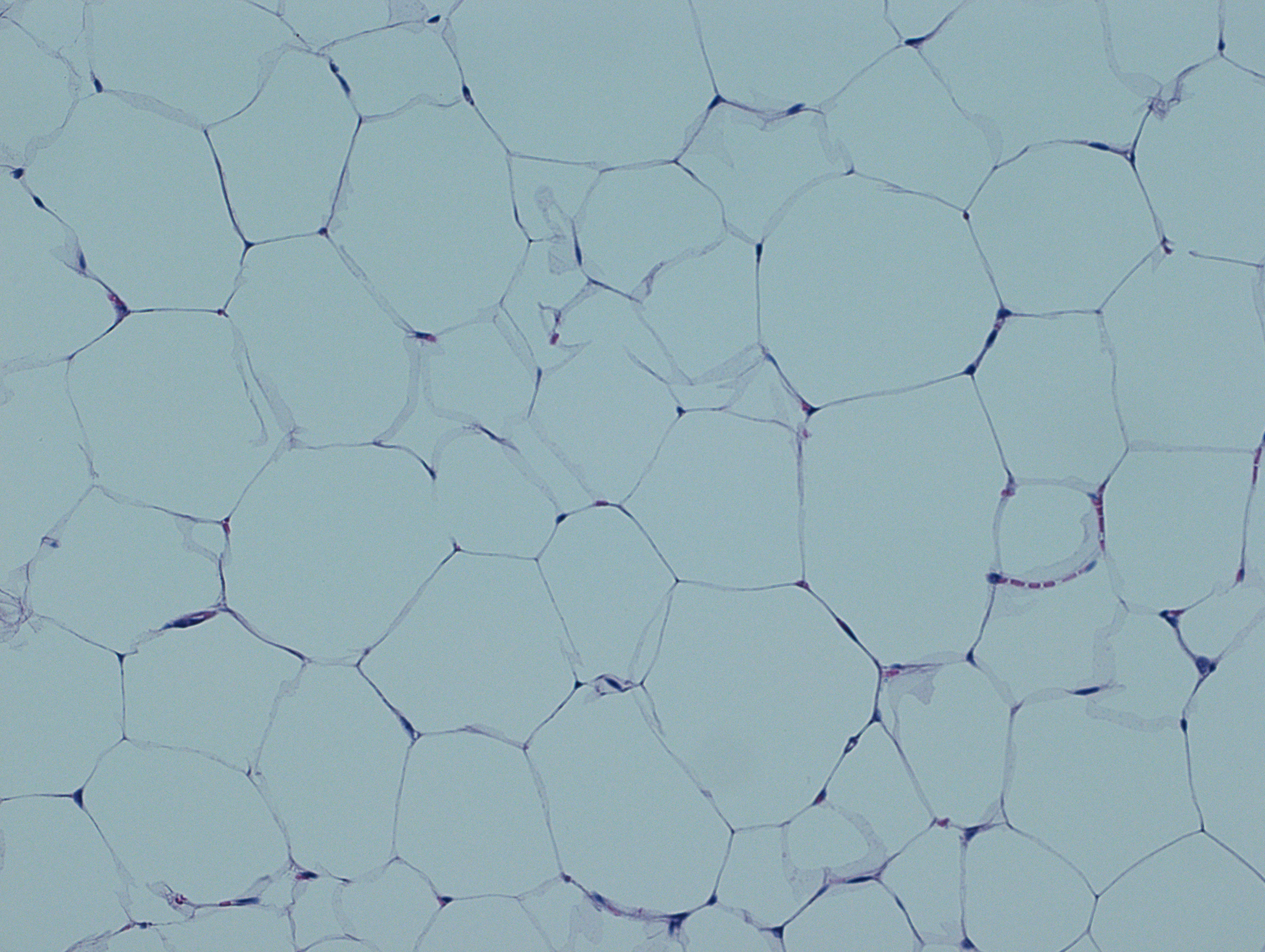

Supplement: Supplementary file 11 — EV and Appendix Figures Source Data [file 44319_2025_398_MOESM11_ESM.zip › Expand View Figures Source Data/Expand View Figures Source Data/Expand View Figure 4/Expand View Figure 4 M/KO-Surgery.tif]
