# Supplementary material for: Deficiency of neuronal LGR4 increases energy expenditure and inhibits food intake via hypothalamic leptin signaling
Source: EMBO Rep. 2025 Mar 11;26(8):2098–120. doi: 10.1038/s44319-025-00398-5 (PMC12018946; doi:10.1038/s44319-025-00398-5)
Supplement: Supplementary file 12 — Expanded View Figures [file 44319_2025_398_MOESM12_ESM.pdf]

Expanded View Figures

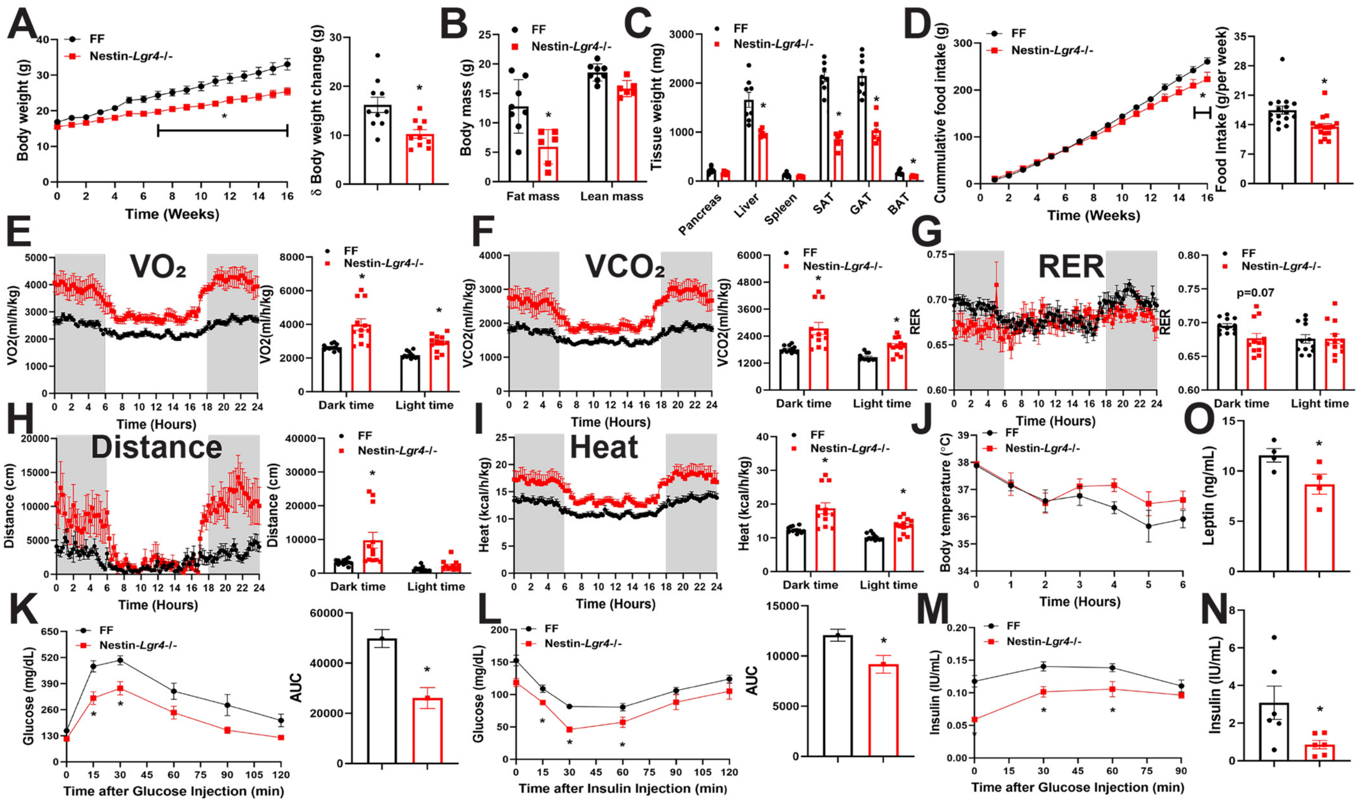

Figure EV1. Metabolic effects of *Lgr4* deficiency in *nestin* neurons of female mice fed HFD.

Female mice at 6 weeks old were fed HFD for 16 weeks. All data were shown as mean±SEM, differences between 2 groups were analyzed by two-tailed Student's *t* test. Comparisons between more than 2 groups or variables were analyzed by one-way or two-way ANOVA and/or Tukey's post hoc test, $*P<0.05$. (**A**) Body weight (left, $P < 0.05$ for week 7 to 16) and body weight change (right, $P = 0.0042$), FF $n = 10$, *Nestin-Lgr4*$^{-/-}$ $n = 10$. (**B**) Body mass (Fat mass: $P = 0.0005$), FF $n = 8$, *Nestin-Lgr4*$^{-/-}$ $n = 6$. (**C**) Tissue weights, FF $n = 8$, *Nestin-Lgr4*$^{-/-}$ $n = 6$ (liver: $P = 0.002698$; SAT: $P < 0.000001$; GAT: $P = 0.000063$; BAT: $P = 0.002363$). (**D**) Cumulative food intake (left, $P < 0.05$ for week 14 to 16) and weekly food intake (right, $P = 0.0024$), FF $n = 8$, *Nestin-Lgr4*$^{-/-}$ $n = 6$. (**E–I**) TSE phenotype data, mice were placed in TSE chamber for 7 days, data from last 3 days were recorded and analyzed, FF $n = 4$, *Nestin-Lgr4*$^{-/-}$ $n = 4$. (**E**) O_2 consumption (dark time: $P < 0.0001$; light time: $P = 0.0187$). (**F**) CO_2 production (dark time: $P = 0.0001$; light time: $P = 0.0446$). (**G**) Respiratory Exchange Ratio (RER). (**H**) locomotor activity (dark time: $P = 0.0009$). (**I**) Energy expenditure (dark time: $P < 0.0001$; light time: $P = 0.0218$). (**J**) Rectal body temperature under 4 °C, FF $n = 6$, *Nestin-Lgr4*$^{-/-}$ $n = 5$. (**K, L**) IPGTT (**K**, $P = 0.0269$) and IPITT (**L**, $P = 0.0155$), FF $n = 8$, *Nestin-Lgr4*$^{-/-}$ $n = 6$. (**M**) Insulin secretion after glucose injection, FF $n = 6$, *Nestin-Lgr4*$^{-/-}$ $n = 6$ (0 min: $P < 0.0001$; 30 min: $P = 0.0044$; 60 min: $P = 0.0200$). (**N**) Plasma Insulin, FF $n = 6$, *Nestin-Lgr4*$^{-/-}$ $n = 6$ ($P = 0.0351$). (**O**) Plasma Leptin, FF $n = 4$, *Nestin-Lgr4*$^{-/-}$ $n = 4$ ($P = 0.0548$) Source data are available online for this figure.

© The Author(s)

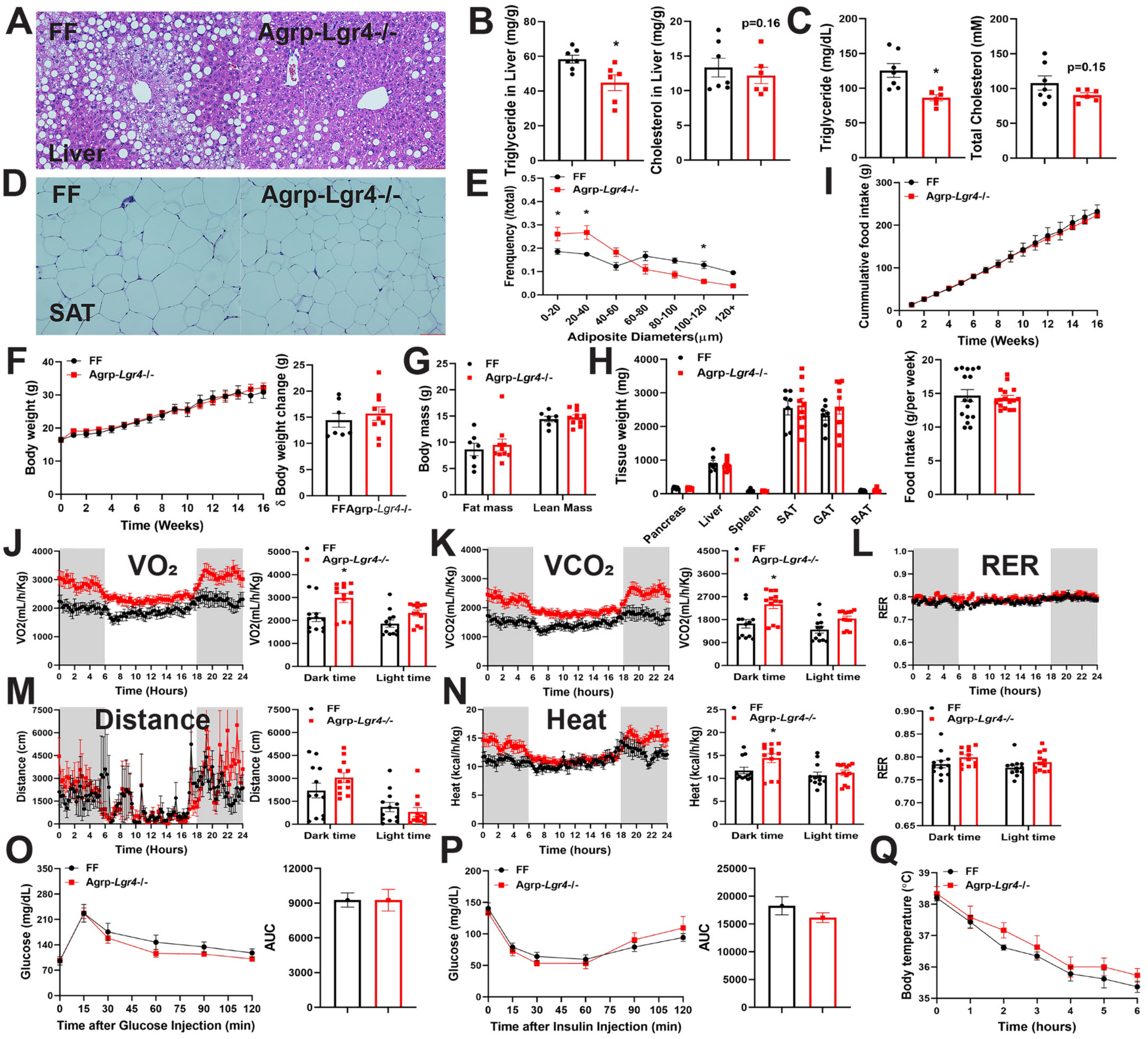

Figure EV2. Metabolic effects of Lgr4 deficiency in AgRP neurons of mice fed HFD.

Male (**A–E**) and female mice (**F–Q**) were fed on HFD at 6 weeks old for 16 weeks. All data were shown as mean±SEM, differences between 2 groups were analyzed by two-tailed Student's *t* test. Comparisons between more than two groups or variables were analyzed by one-way or two-way ANOVA and/or Tukey's post hoc test, $*P < 0.05$. (**A**) Representative H&E staining of liver (scale bar: 500 pixel) sections. (**B**) Triglyceride content (left, $P = 0.0166$) and total cholesterol content (right) in liver, FF $n = 7$, Agrp-Lgr4−/− $n = 6$. (**C**) Triglyceride content (left, $P = 0.0059$) and total cholesterol content (right) in plasma, FF $n = 7$, Agrp-Lgr4−/− $n = 6$. (**D**) Representative H&E staining of SAT (scale bar: 500 pixel) sections. (**E**) Adipocyte size of SAT, FF $n = 6$, Agrp-Lgr4−/− $n = 6$ (0–20 µm: $P = 0.0177$; 20–40 µm: $P = 0.0020$; 100–120 µm: $P = 0.0250$). (**F**) Body weight (left) and body weight change (right), FF $n = 7$, Agrp-Lgr4−/− $n = 10$. (**G**) Body mass, FF $n = 7$, Agrp-Lgr4−/− $n = 10$. (**H**) Tissue weights, FF $n = 7$, Agrp-Lgr4−/− $n = 10$. (**I**) Cumulative food intake (left) and weekly food intake (right), FF $n = 7$, Agrp-Lgr4−/− $n = 10$. (**J–N**) TSE phenotype data, mice were placed in TSE chamber for 7 days, data from last 3 days were recorded and analyzed, FF $n = 4$, Agrp-Lgr4−/− $n = 4$. (**J**) O2 consumption (dark time: $P = 0.0024$). (**K**) CO2 production (dark time: $P = 0.0011$). (**L**) Respiratory Exchange Ratio (RER). (**M**) Locomotor activity. (**N**) Energy expenditure (dark time: $P = 0.0325$). (**O**, **P**) IPGTT (**O**) and IPITT (**P**), FF $n = 5$, Agrp-Lgr4−/− $n = 5$. (**Q**) Rectal body temperature under 4 °C, FF $n = 6$, Agrp-Lgr4−/− $n = 6$. Source data are available online for this figure.

 © The Author(s)

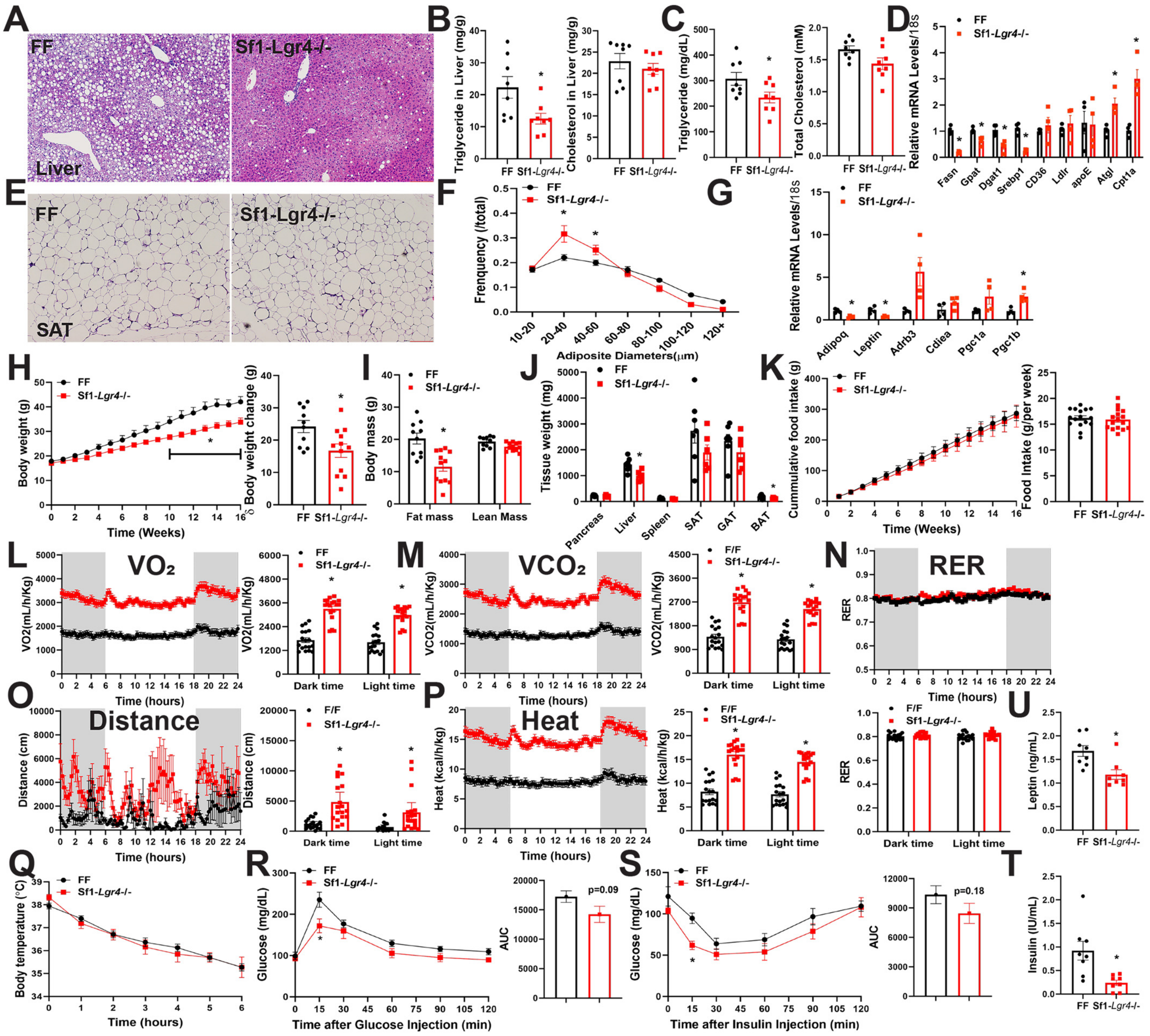

Figure EV3. Metabolic effects of Lgr 4 knockdown in Sf1 neurons of mice fed HFD.

Male mice (**A–G**) and female mice (**H–T**) were fed HFD at 6 weeks old for 16 weeks. All data were shown as mean±SEM, Differences between two groups were analyzed by two-tailed Student's *t* test. Comparisons between more than two groups or variables were analyzed by one-way or two-way ANOVA and/or Tukey's post hoc test, $^*P < 0.05$. (**A**) Representative H&E staining of liver (scale bar: 50 μm) sections. (**B**) Triglyceride content (left, $P = 0.0216$) and total cholesterol content (right) in liver, FF $n = 8$, Sf1-Lgr4−/− $n = 8$. (**C**) Triglyceride content (left, $P = 0.0417$) and total cholesterol content (right) in plasma, FF $n = 8$, Sf1-Lgr4−/− $n = 8$. (**D**) Expression of genes related to lipid genesis, transport and β-oxidation in liver tissue, FF $n = 4$, Sf1-Lgr4−/− $n = 4$ (Fasn: $P = 0.000293$; Gpat: $P = 0.024476$; Dgat1: $P = 0.024476$; Srebp1: $P = 0.001694$; Atgl: $P = 0.024476$; Cpt1α: $P = 0.012742$). (**E**) Representative H&E staining of SAT (scale bar: 50 μm) sections. (**F**) Adipocyte size of SAT, FF $n = 6$, Sf1-Lgr4−/− $n = 5$ (20–40 μm: $P < 0.0001$; 40–60 μm: $P = 0.0499$). (**G**) Expression of genes related to lipolysis and browning in SAT, FF $n = 4$, Sf1-Lgr4−/− $n = 4$ (Adipoq: $P = 0.009246$; Leptin: $P = 0.027954$; Pgc1β: $P = 0.026012$). (**H**) Body weight (left, $P < 0.05$ for week 10 to 16) and body weight change (right, $P = 0.0173$), FF $n = 10$, Sf1-Lgr4−/− $n = 12$. (**I**) Body mass, FF $n = 10$, Sf1-Lgr4−/− $n = 12$ (Fat mass: $P < 0.0001$). (**J**) Tissue weights, FF $n = 7$, Sf1-Lgr4−/− $n = 6$ (liver: $P = 0.005946$; BAT: $P = 0.022265$). (**K**) Cumulative food intake (left) and weekly food intake (right), FF $n = 10$, Sf1-Lgr4−/− $n = 12$. (**L–P**) TSE phenotype data, mice were placed in TSE chamber for 7 days, data from last 3 days were recorded and analyzed, FF $n = 6$, Sf1-Lgr4−/− $n = 6$. (**L**) O_2 consumption (dark time: $P < 0.0001$; light time: $P < 0.0001$). (**M**) CO_2 production (dark time: $P < 0.0001$; light time: $P < 0.0001$). (**N**) Respiratory exchange ratio (RER). (**O**) Locomotor activity (dark time: $P = 0.022250$; light time: $P = 0.010912$). P Energy expenditure (dark time: $P < 0.0001$; light time: $P < 0.0001$). (**Q**) Rectal body temperature under 4 °C, FF $n = 8$, Sf1-Lgr4−/− $n = 7$. (**R–T**) IPGTT (**R**) and IPITT (**S**), FF $n = 8$, Sf1-Lgr4−/− $n = 8$. (**T**) Plasma insulin levels, FF $n = 8$, Sf1-Lgr4−/− $n = 8$ ($P = 0.005422$). (**U**) Plasma leptin levels, FF $n = 8$, Sf1-Lgr4−/− $n = 8$ ($P = 0.0058$). Source data are available online for this figure.

© The Author(s)

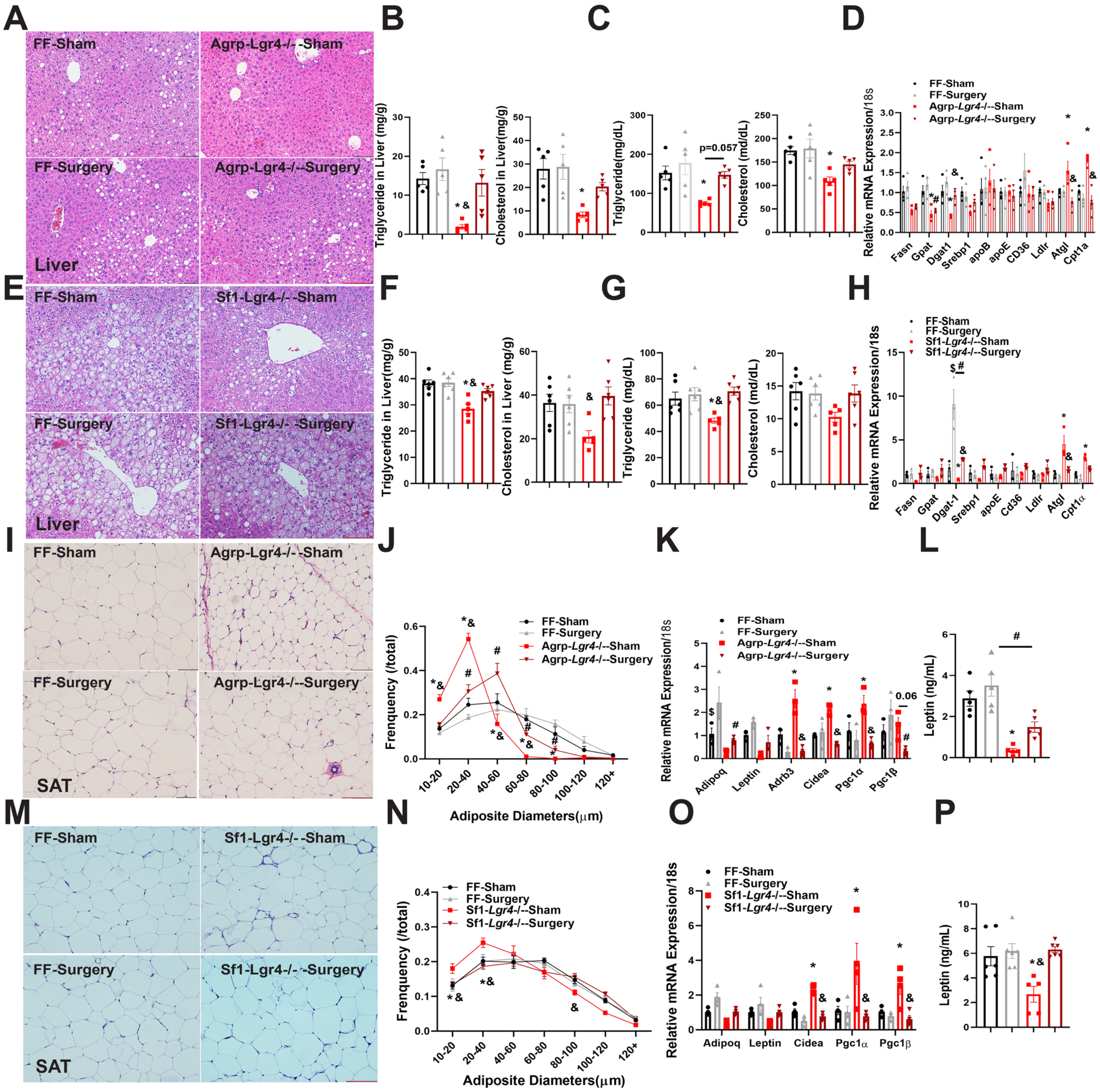
A
FF-Sham
Agrp-Lgr4-/- -Sham
FF-Surgery
Agrp-Lgr4-/--Surgery
Liver
B
Triglyceride in Liver (mg/g)
Cholesterol in Liver(mg/g)
C
Triglyceride(mg/dL)
p=0.057
Cholesterol (md/dL)
D
Relative mRNA Expression/18s
FF-Sham
FF-Surgery
Agrp-Lgr4-/--Sham
Agrp-Lgr4-/--Surgery
Fasn
Gpat
Dgat1
Srebp1
apoB
apoE
CD36
Ldlr
Atgl
Cpt1a
E
FF-Sham
Sf1-Lgr4-/--Sham
FF-Surgery
Sf1-Lgr4-/- -Surgery
Liver
F
Triglyceride in Liver(mg/g)
Cholesterol in Liver (mg/g)
G
Triglyceride (mg/dL)
Cholesterol (md/dL)
H
Relative mRNA Expression/18s
FF-Sham
FF-Surgery
Sf1-Lgr4-/--Sham
Sf1-Lgr4-/--Surgery
Fasn
Gpat
Dgat-1
Srebp1
apoE
Cd36
Ldlr
Atgl
Cpt1α
I
FF-Sham
Agrp-Lgr4-/--Sham
FF-Surgery
Agrp-Lgr4-/--Surgery
SAT
J
Frenquency (/total)
FF-Sham
FF-Surgery
Agrp-Lgr4-/--Sham
Agrp-Lgr4-/--Surgery
10-20
20-40
40-60
60-80
80-100
100-120
120+
Adiposite Diameters(μm)
K
Relative mRNA Expression/18s
FF-Sham
FF-Surgery
Agrp-Lgr4-/--Sham
Agrp-Lgr4-/--Surgery
0.06
Adipoq
Leptin
Adrb3
Cidea
Pgc1α
Pgc1β
L
Leptin (ng/mL)
M
FF-Sham
Sf1-Lgr4-/--Sham
FF-Surgery
Sf1-Lgr4-/- -Surgery
SAT
N
Frenquency (/total)
FF-Sham
FF-Surgery
Sf1-Lgr4-/--Sham
Sf1-Lgr4-/--Surgery
10-20
20-40
40-60
60-80
80-100
100-120
120+
Adiposite Diameters(μm)
O
Relative mRNA Expression/18s
FF-Sham
FF-Surgery
Sf1-Lgr4-/--Sham
Sf1-Lgr4-/--Surgery
Adipoq
Leptin
Cidea
Pgc1α
Pgc1β
P
Leptin (ng/mL)

© The Author(s)

Figure EV4. Intrascapular BAT bilateral sympathectomy reverses the metabolic benefits in *Agrp-Lgr4*$^{-/-}$ and *Sf1-Lgr4*$^{-/-}$ mice.

Intrascapular BAT Bilateral Sympathectomy were performed on male mice fed on HFD for 8 weeks, beginning at 6 weeks. All data were shown as mean±SEM, comparisons between more than two groups or variables were analyzed by one-way or two-way ANOVA and/or Tukey's post hoc test,P<0.05, * FF-sham vs *Agrp* or *Sf1-Lgr4*$^{-/-}$-sham, $^{\$}$ FF-sham vs FF-Surgery, $^{\&}$ *Agrp* or *Sf1-Lgr4*$^{-/-}$-sham vs *Agrp* or *Sf1-Lgr4*$^{-/-}$-Surgery, $^{\#}$ FF-Surgery vs *Agrp* or *Sf1-Lgr4*$^{-/-}$-Surgery. (**A**) Representative H&E staining of liver (scale bar: 500 pixel) sections from FF and *Agrp-Lgr4*$^{-/-}$ mice with or without surgery. (**B**) Triglyceride content (left, $^{*}P = 0.0102$; $^{\&}P = 0.0197$) and total cholesterol content (right, $^{*}P = 0.0076$) in liver, FF-sham $n = 5$, *Agrp-Lgr4*$^{-/-}$-sham $n = 5$, FF-Surgery $n = 5$, *Agrp-Lgr4*$^{-/-}$-Surgery $n = 5$. (**C**) Triglyceride content (left, $^{*}P = 0.0393$) and total cholesterol content (right, $^{*}P = 0.0093$) in plasma, FF-sham $n = 5$, *Agrp-Lgr4*$^{-/-}$-sham $n = 5$, FF-Surgery $n = 5$, *Agrp-Lgr4*$^{-/-}$-Surgery $n = 5$. (**D**) Expression of genes related to lipid genesis, transport and β-oxidation in liver tissue, FF-sham $n = 4$, *Agrp-Lgr4*$^{-/-}$-sham $n = 4$, FF-Surgery $n = 4$, *Agrp-Lgr4*$^{-/-}$-Surgery $n = 4$ (Gpat: $^{*}P = 0.0437$; $^{\&}P = 0.0153$. Dgat1: $^{*}P = 0.0272$; $^{\&}P = 0.0429$. Atgl: $^{*}P = 0.0020$; $^{\&}P < 0.0001$. Cpt1α: $^{*}P = 0.0018$; $^{\&}P < 0.0001$). (**E**) Representative H&E staining of liver (scale bar: 500 pixel) sections from FF and *Sf1-Lgr4*$^{-/-}$ mice with or without surgery. (**F**) Triglyceride content (left, $^{*}P = 0.0007$; $^{\&}P = 0.0176$) and total cholesterol content (right, $^{\&}P = 0.0164$) in liver, FF-Sham $n = 6$, FF-Surgery $n = 6$, *Sf1-Lgr4*$^{-/-}$-Sham $n = 5$, *Sf1-Lgr4*$^{-/-}$-Surgery $n = 6$. (**G**) Triglyceride content (left, $^{*}P = 0.05$; $^{\&}P = 0.0067$) and total cholesterol content (right) in plasma, FF-Sham $n = 6$, FF-Surgery $n = 6$, *Sf1-Lgr4*$^{-/-}$-Sham $n = 5$, *Sf1-Lgr4*$^{-/-}$-Surgery $n = 6$. (**H**) Expression of genes related to lipid genesis, transport and β-oxidation in liver tissue, FF-Sham $n = 3$, FF-Surgery $n = 3$, *Sf1-Lgr4*$^{-/}$-Sham $n = 3$, *Sf1-Lgr4*$^{-/-}$-Surgery $n = 3$ (Dgat1: $^{\$\#}P < 0.0001$; $^{\&}P = 0.0037$. Atgl: $^{\$\&}P < 0.0001$.Cpt1α: $^{*}P = 0.0159$). (**I**) Representative H&E staining of SAT (scale bar: 500 pixel) sections from FF and *Agrp-Lgr4*$^{-/-}$ mice with or without surgery. (**J**) Adipocyte size of SAT, FF-sham $n = 5$, *Agrp-Lgr4*$^{-/-}$-sham $n = 5$, FF-Surgery $n = 5$, *Agrp-Lgr4*$^{-/-}$-Surgery $n = 5$ (1–20 μm: $^{*}P = 0.0007$, $^{\&}P = 0.0034$. 20-40 μm: $^{*\&}P < 0.0001$; $^{\#}P = 0.0019$. 40–60 μm: $^{*}P = 0.0134$. $^{\&\#}P < 0.0001$; 60-80 μm: $^{*}P < 0.0001$; $^{\&}P = 0.0153$; $^{\#}P = 0.0302$. 80–100 μm: $^{*}P = 0.0089$; $^{\&}P = 0.0032$). (**K**) Expression of genes related to lipolysis and browning in SAT, FF-sham $n = 3$, *Agrp-Lgr4*$^{-/-}$-sham $n = 3$, FF-Surgery $n = 3$, *Agrp-Lgr4*$^{-/-}$-Surgery $n = 3$ (Adipoq: $^{\$}P = 0.0137$; $^{\#}P = 0.0024$. Adrb3: $^{*}P = 0.0043$; $^{\&}P < 0.0001$; Cidea: $^{*}P = 0.0464$; $^{\&}P = 0.0046$. Pgc-1α: $^{*}P = 0.0313$; $^{\&}P = 0.0013$. Pgc-1β: $^{\#}P = 0.0039$). (**L**) Plasma leptin levels, FF-sham $n = 5$, *Agrp-Lgr4*$^{-/-}$-sham $n = 5$, FF-Surgery $n = 5$, *Agrp-Lgr4*$^{-/-}$-Surgery $n = 5$ ($^{*}P = 0.0005$; $^{\#}P = 0.0042$). (**M**) Representative H&E staining of SAT (scale bar: 200 pixel) sections from FF and *Sf1-Lgr4*$^{-/-}$ mice with or without surgery. (**N**) Adipocyte size of SAT, FF-Sham $n = 6$, FF-Surgery $n = 6$, *Sf1-Lgr4*$^{-/-}$-Sham $n = 5$, *Sf1-Lgr4*$^{-/-}$-Surgery $n = 6$ (1–20 μm: $^{*}P = 0.0263$; $^{\&}P = 0.0349$. 20–40 μm: $^{*}P = 0.0109$; $^{\&}P = 0.0006$. 100–120 μm: $^{\&}P = 0.0108$). (**O**) Expression of genes related to lipolysis and browning in SAT, FF-Sham $n = 4$, FF-Surgery $n = 4$, *Sf1-Lgr4*$^{-/-}$-Sham $n = 4$, *Sf1-Lgr4*$^{-/-}$-Surgery $n = 4$ (Cidea: $^{*}P = 0.0422$; $^{\&}P = 0.0086$. Pgc-1α: $^{*\&}P < 0.0001$. Pgc-1β: $^{*\&}P < 0.0001$). (**P**) Plasma leptin levels, FF-Sham $n = 6$, FF-Surgery $n = 6$, *Sf1-Lgr4*$^{-/-}$-Sham $n = 5$, *Sf1-Lgr4*$^{-/-}$-Surgery $n = 6$ ($^{*}P = 0.0107$; $^{\&}P = 0.0028$). Source data are available online for this figure.
